# Supplementary material for: Internet-delivered transdiagnostic psychological treatments for individuals with depression, anxiety or both: a systematic review with meta-analysis of randomised controlled trials
Source: BMJ Open. 2024 Apr 3;14(4):e075796. doi: 10.1136/bmjopen-2023-075796 (PMC11015301; doi:10.1136/bmjopen-2023-075796)
Supplement: Supplementary data [file bmjopen-2023-075796supp001.pdf]

Internet-delivered transdiagnostic psychological treatments for individuals with depression, anxiety, or both

Systematic search: 2021 main search

Documentation of search strategies
University Library search consultation group

Date: May 24, 2021

Topic/research question: Systematic review and meta-analysis of transdiagnostic psychological interventions delivered via the internet for individuals suffering from common mental disorders (PROSPERO: CRD42021243172)

Name of researcher(s): Erland Axelsson

Librarian(s): Sabina Gillsund & Emma-Lotta Säätelä

Databases:

1. Medline (Ovid)
2. Cochrane Library (Wiley)
3. Web of Science Core Collection (Clarivate Analytics)
4. PsycInfo (Ovid)

Total number of hits:

- Before deduplication: 7,366
- After deduplication: 3,519

Comments:

Karolinska Institutet

1. Medline

Interface: Ovid MEDLINE(R) and Epub Ahead of Print, In-Process & Other Non-Indexed Citations and Daily<br><br>Date of Search: 24 May 2021<br><br>Number of hits: 1,898<br><br>Comment: In Ovid, two or more words are automatically searched as phrases; i.e. no quotation marks are needed	Field labels<br><br>• exp/ = exploded MeSH term<br>• / = non exploded MeSH term<br>• .ti,ab,kf. = title, abstract and author keywords<br>• adjx = within x words, regardless of order<br>• * = truncation of word for alternate endings

#	Searches	Results
1	exp Psychotherapy/	202021
2	(("acceptance and commitment" or affect-focused or behavio?r* or biofeedback or computeri* or cognit* or digital or "emotional awareness and expression" or educat* or exposure or guided or internet* or interpersonal or mobile or online or positive psychology or psychodynamic or psychologic* or self-guided or self-help or transdiagnost* or web*) adj2 (intervention* or therap* or treat* or program*)).ti,ab,kf.	188596
3	(behavio?ral activation or bias modification or cognitive restructuring or hypno* or meditation or mindfulness or problem solving or psychoeducation* or psychotherap* or relaxation or self-management or unified protocol).ti,ab,kf.	251506
4	or/1-3	538226
5	Anxiety Disorders/	35452
6	Agoraphobia/	2617
7	Neurotic Disorders/	17987
8	Obsessive-Compulsive Disorder/	14943
9	Panic Disorder/	7019
10	exp Phobic Disorders/	11590
11	Stress Disorders, Post-Traumatic/	34732
12	Anxiety/	87458
13	(agoraphobi* or anxi* or obsessive-compulsive or panic or phobi* or post-traumatic stress or posttraumatic stress).ti,ab,kf.	281431
14	or/5-13	336088

15	Mood Disorders/	14821
16	Depressive Disorder/	73869
17	Depressive Disorder, Major/	32106
18	Affective Symptoms/	13300
19	Depression/	127756
20	depress*.ti,ab,kf.	491024
21	((affective or mood) adj2 disorder*).ti,ab,kf.	37052
22	or/15-21	562251
23	14 and 22	140055
24	(emotional adj2 disorder*).ti,ab,kf.	3677
25	(transdiagnost* or unified protocol).ti,ab,kf.	2780
26	24 or 25	6275
27	23 or 26	144265
28	Internet/	75778
29	Internet-Based Intervention/	544
30	Therapy, Computer-Assisted/	6898
31	Telemedicine/	28197
32	Telerehabilitation/	548
33	exp Remote Consultation/	5247
34	Mobile Applications/	7799
35	Videoconferencing/	1919
36	(app or apps or cellphone* or computer* or digital or e mail or email or internet* or mobile application* or online or phone* or smartphone* or technolog* or telephone* or text message* or video* or virtual or web based or web site or website).ti,ab,kf.	1396114
37	(distance counsel?ing or distance consultation* or e consultation* or econsultation* or e counsel?ing or ecounsel?ing or e health* or ehealth* or e therapies or e therapy or etherap* or e visit* or evisit* or m health or mhealth or mobile counsel?ing or mobile consultation* or remote consultation* or remote counsel?ing or tele health* or telehealth* or tele consultation* or teleconsultation* or tele medicine or telemedicine or tele rehabilitation or telerehabilitation).ti,ab,kf.	36032
38	or/28-37	1441976
39	randomized controlled trial.pt.	531357
40	controlled clinical trial.pt.	94168
41	randomi?ed.ab.	622540

42	placebo.ab.	218143
43	clinical trials as topic.sh.	195916
44	randomly.ab.	357785
45	trial.ti.	240304
46	or/39-45	1407342
47	(systematic review or meta-analy*).ti.	219916
48	46 not 47	1340792
49	exp animals/ not humans.sh.	4832148
50	48 not 49	1230987
51	4 and 27 and 38 and 50	1921
52	limit 51 to english language	1905
53	limit 52 to yr="1995 -Current"	1898

Kolaas K, *et al. BMJ Open* 2024; 14:e075796. doi: 10.1136/bmjopen-2023-075796

2. Cochrane Library

Interface: Wiley<br>Date of Search: 24 May 2021<br>Number of hits: 2,337	Field labels<br>• ti,ab,kw = title, abstract and author keywords<br>• NEAR/x = within x words, regardless of order<br>• * = truncation of word for alternate endings

ID	Search	Hits
#1	MeSH descriptor: [Psychotherapy] explode all trees	24621
#2	(("acceptance and commitment" or "affect-focused" or behavio?r* or "biofeedback" or computeri* or cognit* or "digital" or "emotional awareness and expression" or educat* or "exposure" or "guided" or internet* or "interpersonal" or "mobile" or "online" or "positive psychology" or "psychodynamic" or psychologic* or "self-guided" or "self-help" or transdiagnost* or web*) NEAR/2 (intervention* or therap* or treat* or program*)):ti,ab,kw	81443
#3	((behavio?ral NEXT activation) or "bias modification" or "cognitive restructuring" or hypno* or "meditation" or "mindfulness" or "problem solving" or psychoeducation* or psychotherap* or "relaxation" or "self-management" or "unified protocol"):ti,ab,kw	54905
#4	#1 or #2 or #3	121709
#5	MeSH descriptor: [Anxiety Disorders] this term only	3927
#6	MeSH descriptor: [Agoraphobia] this term only	436
#7	MeSH descriptor: [Neurotic Disorders] this term only	300
#8	MeSH descriptor: [Obsessive-Compulsive Disorder] this term only	1050
#9	MeSH descriptor: [Panic Disorder] this term only	955
#10	MeSH descriptor: [Phobic Disorders] explode all trees	1382
#11	MeSH descriptor: [Stress Disorders, Post-Traumatic] this term only	2707
#12	MeSH descriptor: [Anxiety] this term only	7681
#13	(agoraphobi* or anxi* or "obsessive compulsive" or "obsessive-compulsive" or "panic" or phobi* or "post-traumatic stress" or "posttraumatic stress"):ti,ab,kw	63654
#14	#5 or #6 or #7 or #8 or #9 or #10 or #11 or #12 or #13	64273
#15	MeSH descriptor: [Mood Disorders] this term only	839
#16	MeSH descriptor: [Depressive Disorder] this term only	8011
#17	MeSH descriptor: [Depressive Disorder, Major] this term only	5111
#18	MeSH descriptor: [Affective Symptoms] this term only	459
#19	MeSH descriptor: [Depression] this term only	12730
#20	depress*:ti,ab,kw	89514
#21	(("affective" or "mood") NEAR/3 disorder*):ti,ab,kw	5404
#22	#15 or #16 or #17 or #18 or #19 or #20 or #21	91976
#23	#14 and #22	30987
#24	("emotional" NEAR/2 disorder*):ti,ab,kw	862
#25	(transdiagnost* or "unified protocol"):ti,ab,kw	730
#26	#24 or #25	1468
#27	#23 or #26	31780
#28	MeSH descriptor: [Internet] this term only	3893
#29	MeSH descriptor: [Internet-Based Intervention] this term only	182

Kolaas K, et al. BMJ Open 2024; 14:e075796. doi: 10.1136/bmjopen-2023-075796

#30	MeSH descriptor: [Therapy, Computer-Assisted] this term only	1342
#31	MeSH descriptor: [Telemedicine] this term only	2293
#32	MeSH descriptor: [Telerehabilitation] this term only	126
#33	MeSH descriptor: [Remote Consultation] explode all trees	398
#34	MeSH descriptor: [Mobile Applications] this term only	765
#35	MeSH descriptor: [Videoconferencing] this term only	190
#36	("app" or "apps" or cellphone* or computer* or "digital" or "e mail" or "email" or internet* or ("mobile" NEXT application*) or "online" or phone* or smartphone* or technolog* or telephone* or ("text" NEXT message*) or video* or "virtual" or "web based" or "web site" or "website"):ti,ab,kw	150142
#37	(("distance" NEXT "counsel?ing") or ("distance" NEXT consultation*) or ("e" NEXT consultation*) or econsultation* or ("e" NEXT "counsel?ing") or "ecounsel?ing" or ("e" NEXT health*) or ehealth* or "e therapies" or "e therapy" or etherap* or ("e" NEXT visit*) or evisit* or "m health" or "mhealth" or ("mobile" NEXT "counsel?ing") or ("mobile" NEXT consultation*) or ("remote" NEXT consultation*) or ("remote" NEXT "counsel?ing") or ("tele" NEXT health*) or telehealth* or ("tele" NEXT consultation*) or teleconsultation* or "tele medicine" or "telemedicine" or "tele rehabilitation" or "telerehabilitation"):ti,ab,kw	9310
#38	#28 or #29 or #30 or #31 or #32 or #33 or #34 or #35 or #36 or #37	152807
#39	#4 and #27 and #38	4365
#40	(clinicaltrials or trialsearch):so	364015
#41	#39 NOT #40	2416
	Limit to publication date 1995-2021 in Trials	2337

Kolaas K, *et al. BMJ Open* 2024; 14:e075796. doi: 10.1136/bmjopen-2023-075796

Karolinska Institutet

3. Web of Science Core Collection

Interface: Clarivate Analytics<br><br>Date of Search: 24 May 2021<br><br>Number of hits: 2,233	Field labels<br><br>• TS/Topic = title, abstract, author keywords and Keywords Plus<br>• NEAR/x = within x words, regardless of order<br>• * = truncation of word for alternate endings<br><br>Note: sometimes “quotation marks” are needed for single search terms to avoid automatic term mapping (lemmatization).

18 2,233

#14 AND #11 AND #8 AND #3

Refined by: LANGUAGES: (ENGLISH) AND [excluding] PUBLICATION YEARS: (1994 OR 1993) AND DOCUMENT TYPES: (ARTICLE OR CORRECTION OR EARLY ACCESS)

17 2,366

#14 AND #11 AND #8 AND #3

Refined by: LANGUAGES: (ENGLISH) AND [excluding] PUBLICATION YEARS: (1994 OR 1993)

16 2,370

#14 AND #11 AND #8 AND #3

Refined by: LANGUAGES: (ENGLISH)

15 2,395

#14 AND #11 AND #8 AND #3

14 916,173

#12 NOT #13

13 259,971

TI=(“systematic review” or “meta-analy*”)

12 982,134

TI=(“randomi$ed” OR “randomi$ed” OR “randomi$ation” OR “randomi$ation” OR placebo* OR (random* AND (allocat* OR assign*)) OR (blind* AND (“single” OR “double” OR “treble” OR “triple”))) OR AB=(“randomi$ed” OR “randomi$ed” OR “randomi$ation” OR “randomi$ation” OR placebo* OR (random* AND (allocat* OR assign*)) OR (blind* AND (“single” OR “double” OR “treble” OR “triple”)))

Karolinska Institutet

11 4,745,692
#10 OR #9

# 10 51,707
TS=(“distance counsel$ing” or “distance consultation*” or “e consultation*” or econsultation* or “e counsel$ing” or “ecounsel$ing” or “e health*” or ehealth* or “e therapies” or “e therapy” or etherap* or “e visit*” or evisit* or “m health” or “mhealth” or “mobile counsel$ing” or “mobile consultation*” or “remote consultation*” or “remote counsel$ing” or “tele health*” or telehealth* or “tele consultation*” or teleconsultation* or “tele medicine” or “telemedicine” or “tele rehabilitation” or “telerehabilitation")

# 9 4,726,717
TS=(“app” or “apps” or cellphone* or computer* or “digital” or “e mail” or “email” or internet* or “mobile application*” or “online” or phone* or smartphone* or technolog* or telephone* or “text message*” or video* or “virtual” or “web based” or “web site” or “website”)

8 176,232
#7 OR #6

# 7 7,752
TS=((”emotional” NEAR/1 disorder*) or transdiagnost* or “unified protocol”)

6 171,279
#5 AND #4

# 5 691,430
TS=(depress* or ((“affective” or “mood”) NEAR/1 disorder*))

# 4 401,339
TS=(agoraphobi* or anxi* or “obsessive-compulsive” or “panic” or phobi* or “post-traumatic stress” or “posttraumatic stress”)

3 871,462
#2 OR #1

2 644,089

TS=((“behavio$ral activation” or “bias modification” or “cognitive restructuring” or hypno* or “meditation” or “mindfulness” or “problem solving” or psychoeducation* or psychotherap* or “relaxation” or “self-management” or “unified protocol”))

1 263,093

TS=((("acceptance and commitment" or “affect-focused” or behavio$r* or “biofeedback” or computeri* or cognit* or “digital” or "emotional awareness and expression" or educat* or “exposure” or “guided” or internet* or “interpersonal” or “mobile” or “online” or “positive psychology” or “psychodynamic” or psychologic* or “self-guided” or “self-help” or transdiagnost* or web*) NEAR/1 (intervention* or therap* or treat* or program*)))

Kolaas K, *et al. BMJ Open* 2024; 14:e075796. doi: 10.1136/bmjopen-2023-075796

4. Psycinfo

Interface: Ovid<br><br>Date of Search: 24 May 2021<br><br>Number of hits: 898<br><br>Comment: In Ovid, two or more words are automatically searched as phrases; i.e. no quotation marks are needed	Field labels<br><br>• exp/ = exploded controlled term<br>• / = non exploded controlled term<br>• .ti,ab,id. = title, abstract and author keywords<br>• adjx = within x words, regardless of order<br>• * = truncation of word for alternate endings

#	Searches	Results
1	exp Psychotherapy/	207730
2	exp Cognitive Therapy/	13639
3	exp Behavior Therapy/	21289
4	exp Cognitive Behavior Therapy/	23144
5	(("acceptance and commitment" or affect-focused or behavio?r* or biofeedback or computeri* or cognit* or digital or "emotional awareness and expression" or educat* or exposure or guided or internet* or interpersonal or mobile or online or positive psychology or psychodynamic or psychologic* or self-guided or self-help or transdiagnost* or web*) adj2 (intervention* or therap* or treat* or program*)).ti,ab,id.	175860
6	(behavio?ral activation or bias modification or cognitive restructuring or hypno* or meditation or mindfulness or problem solving or psychoeducation* or psychotherap* or relaxation or self-management or unified protocol).ti,ab,id.	240370
7	or/1-6	495594
8	Anxiety Disorders/	19140
9	exp Agoraphobia/	2916
10	Neurosis/	7043
11	Obsessive Compulsive Disorder/	14683
12	Panic Disorder/	7760
13	exp Phobias/	13434
14	Posttraumatic Stress Disorder/	34688
15	Anxiety/	65640
16	(agoraphobi* or anxi* or obsessive-compulsive or panic or phobi* or post-traumatic stress or posttraumatic stress).ti,ab,id.	283726

Kolaas K, *et al. BMJ Open* 2024; 14:e075796. doi: 10.1136/bmjopen-2023-075796

17	or/8-16	296637
18	Affective Disorders/	14669
19	exp Major Depression/	138828
20	"Depression (Emotion)"/	26043
21	depress*.ti,ab,id.	321600
22	((affective or mood) adj2 disorder*).ti,ab,id.	35856
23	or/18-22	343842
24	17 and 23	109245
25	(emotional adj2 disorder*).ti,ab,id.	5905
26	(transdiagnost* or unified protocol).ti,ab,id.	3036
27	25 or 26	8661
28	24 or 27	115823
29	Internet/	29614
30	Digital Interventions/	593
31	Computer Assisted Therapy/	1140
32	exp Telemedicine/	9675
33	Digital Technology/	719
34	Videoconferencing/	645
35	Mobile Applications/	1193
36	(app or apps or cellphone* or computer* or digital or e mail or email or internet* or mobile application* or online or phone* or smartphone* or technolog* or telephone* or text message* or video* or virtual or web based or web site or website).ti,ab,id.	436545
37	(distance counsel?ing or distance consultation* or e consultation* or econsultation* or e counsel?ing or ecounsel?ing or e health* or ehealth* or e therapies or e therapy or etherap* or e visit* or evisit* or m health or mhealth or mobile counsel?ing or mobile consultation* or remote consultation* or remote counsel?ing or tele health* or telehealth* or tele consultation* or teleconsultation* or tele medicine or telemedicine or tele rehabilitation or telerehabilitation).ti,ab,id.	7477
38	or/29-37	440321
39	exp Randomized Controlled Trials/	954
40	randomi?ed.ab.	83970
41	placebo.ab.	40594
42	randomly.ab.	76127
43	trial.ti.	32809

44	or/39-43	183930
45	(systematic review or meta-analy*).ti.	40744
46	44 not 45	176278
47	7 and 28 and 38 and 46	1037
48	limit 47 to english language	983
49	limit 48 to yr="1995 -Current"	981
50	limit 49 to "0100 journal"	898

Kolaas K, *et al. BMJ Open* 2024; 14:e075796. doi: 10.1136/bmjopen-2023-075796

Systematic search: 2023 updated main search

Documentation of search strategies
University Library search consultation group

Date: February 2023

Topic/research question: Systematic review and meta-analysis of transdiagnostic psychological interventions delivered via the internet for individuals suffering from common mental disorders (PROSPERO: CRD42021243172)

Name of researcher(s): Erland Axelsson

Librarian(s): Sabina Gillsund & Emma-Lotta Säätelä

Databases:

1. Medline (Ovid)
2. Cochrane Library (Wiley)
3. Web of Science Core Collection (Clarivate Analytics)
4. PsycInfo (EBSCO)

Total number of hits:

- Before deduplication: 10,075
- After deduplication: 4,909

1. Medline

Interface: Ovid MEDLINE(R) ALL<br><br>Date of Search: 6 February 2023<br><br>Number of hits: 2,602<br><br>Comment: In Ovid, two or more words are automatically searched as phrases; i.e. no quotation marks are needed	Field labels<br><br>• exp/ = exploded MeSH term<br>• / = non exploded MeSH term<br>• .ti,ab,kf. = title, abstract and author keywords<br>• adjx = within x words, regardless of order<br>• * = truncation of word for alternate endings

Karolinska Institutet

Database(s): **Ovid MEDLINE(R) ALL** 1946 to February 03, 2023
Search Strategy:

#	Searches	Results
1	exp Psychotherapy/	215915
2	(("acceptance and commitment" or affect-focused or behavio?r* or biofeedback or computeri* or cognit* or digital or "emotional awareness and expression" or educat* or exposure or guided or internet* or interpersonal or mobile or online or positive psychology or psychodynamic or psychologic* or self-guided or self-help or transdiagnost* or web*) adj2 (intervention* or therap* or treat* or program*)).ti,ab,kf.	220048
3	(behavio?ral activation or bias modification or cognitive restructuring or hypno* or meditation or mindfulness or problem solving or psychoeducation* or psychotherap* or relaxation or self-management or unified protocol).ti,ab,kf.	277967
4	or/1-3	597258
5	Anxiety Disorders/	40399
6	Agoraphobia/	2669
7	Neurotic Disorders/	18000
8	Obsessive-Compulsive Disorder/	16224
9	Panic Disorder/	7250
10	exp Phobic Disorders/	12293
11	Stress Disorders, Post-Traumatic/	40198
12	Anxiety/	103449
13	(agoraphobi* or anxi* or obsessive-compulsive or panic or phobi* or post-traumatic stress or posttraumatic stress).ti,ab,kf.	332361
14	or/5-13	389293
15	Mood Disorders/	15751
16	Depressive Disorder/	74889
17	Depressive Disorder, Major/	37299
18	Affective Symptoms/	13816
19	Depression/	146908
20	depress*.ti,ab,kf.	552903
21	((affective or mood) adj2 disorder*).ti,ab,kf.	40988
22	or/15-21	627024
23	14 and 22	168605

Kolaas K, *et al. BMJ Open* 2024; 14:e075796. doi: 10.1136/bmjopen-2023-075796

Karolinska Institutet

24	(emotional adj2 disorder*).ti,ab,kf.	4243
25	(transdiagnost* or unified protocol).ti,ab,kf.	4104
26	24 or 25	8093
27	23 or 26	174000
28	Internet/	80600
29	Internet-Based Intervention/	1071
30	Therapy, Computer-Assisted/	6968
31	Telemedicine/	36073
32	Telerehabilitation/	900
33	exp Remote Consultation/	5715
34	Mobile Applications/	10971
35	Videoconferencing/	2295
36	(app or apps or cellphone* or computer* or digital or e mail or email or internet* or mobile application* or online or phone* or smartphone* or technolog* or telephone* or text message* or video* or virtual or web based or web site or website).ti,ab,kf.	1678151
37	(distance counsel?ing or distance consultation* or e consultation* or econsultation* or e counsel?ing or ecounsel?ing or e health* or ehealth* or e therapies or e therapy or etherap* or e visit* or evisit* or m health or mhealth or mobile counsel?ing or mobile consultation* or remote consultation* or remote counsel?ing or tele health* or telehealth* or tele consultation* or teleconsultation* or tele medicine or telemedicine or tele rehabilitation or telerehabilitation).ti,ab,kf.	51873
38	or/28-37	1730206
39	randomized controlled trial.pt.	585953
40	controlled clinical trial.pt.	95177
41	randomi?ed.ab.	708985
42	placebo.ab.	235960
43	clinical trials as topic.sh.	200855
44	randomly.ab.	402423
45	trial.ti.	279911
46	or/39-45	1550232
47	(systematic review or meta-analy*).ti.	290755
48	46 not 47	1464360
49	exp animals/ not humans.sh.	5089811
50	48 not 49	1343292

51	4 and 27 and 38 and 50	2627
52	limit 51 to english language	2609
53	limit 52 to yr="1995 -Current"	2602

Karolinska Institutet

2. Cochrane Library

Interface: Wiley<br><br>Date of Search: 3 February 2023<br><br>Number of hits: 3,068	Field labels<br><br>• ti,ab,kw = title, abstract and author keywords<br>• NEAR/x = within x words, regardless of order<br>• * = truncation of word for alternate endings

ID	Search	Hits
#1	MeSH descriptor: [Psychotherapy] explode all trees	30769
#2	(("acceptance and commitment" or "affect-focused" or behavio?r* or "biofeedback" or computeri* or cognit* or "digital" or "emotional awareness and expression" or educat* or "exposure" or "guided" or internet* or "interpersonal" or "mobile" or "online" or "positive psychology" or "psychodynamic" or psychologic* or "self-guided" or "self-help" or transdiagnost* or web*) NEAR/2 (intervention* or therap* or treat* or program*)):ti,ab,kw	95851
#3	((behavio?ral NEXT activation) or "bias modification" or "cognitive restructuring" or hypno* or "meditation" or mindfulness or "problem solving" or psychoeducation* or psychotherap* or "relaxation" or "self-management" or "unified protocol"):ti,ab,kw	64668
#4	#1 or #2 or #3	143208
#5	MeSH descriptor: [Anxiety Disorders] this term only	5112
#6	MeSH descriptor: [Agoraphobia] this term only	472
#7	MeSH descriptor: [Neurotic Disorders] this term only	316
#8	MeSH descriptor: [Obsessive-Compulsive Disorder] this term only	1250
#9	MeSH descriptor: [Panic Disorder] this term only	1053
#10	MeSH descriptor: [Phobic Disorders] explode all trees	1942
#11	MeSH descriptor: [Stress Disorders, Post-Traumatic] this term only	3561
#12	MeSH descriptor: [Anxiety] this term only	9845
#13	(agoraphobi* or anxi* or "obsessive compulsive" or "obsessive-compulsive" or "panic" or phobi* or "post-traumatic stress" or "posttraumatic stress"):ti,ab,kw	76993
#14	#5 or #6 or #7 or #8 or #9 or #10 or #11 or #12 or #13	77700
#15	MeSH descriptor: [Mood Disorders] this term only	1004
#16	MeSH descriptor: [Depressive Disorder] this term only	9053
#17	MeSH descriptor: [Depressive Disorder, Major] this term only	6360
#18	MeSH descriptor: [Affective Symptoms] this term only	514
#19	MeSH descriptor: [Depression] this term only	15880
#20	depress*:ti,ab,kw	103690
#21	(("affective" or "mood") NEAR/3 disorder*):ti,ab,kw	6072
#22	#15 or #16 or #17 or #18 or #19 or #20 or #21	106436
#23	#14 and #22	38854
#24	("emotional" NEAR/2 disorder*):ti,ab,kw	1098
#25	(transdiagnost* or "unified protocol"):ti,ab,kw	1061
#26	#24 or #25	1974
#27	#23 or #26	39878

Kolaas K, et al. BMJ Open 2024; 14:e075796. doi: 10.1136/bmjopen-2023-075796

#28	MeSH descriptor: [Internet] this term only	4780
#29	MeSH descriptor: [Internet-Based Intervention] this term only	527
#30	MeSH descriptor: [Therapy, Computer-Assisted] this term only	1476
#31	MeSH descriptor: [Telemedicine] this term only	3338
#32	MeSH descriptor: [Telerehabilitation] this term only	229
#33	MeSH descriptor: [Remote Consultation] 3 tree(s) exploded	440
#34	MeSH descriptor: [Mobile Applications] this term only	1476
#35	MeSH descriptor: [Videoconferencing] this term only	259
#36	("app" or "apps" or cellphone* or computer* or "digital" or "e mail" or "email" or internet* or (mobile NEXT application*) or "online" or phone* or smartphone* or technolog* or telephone* or (text NEXT message*) or video* or "virtual" or "web based" or "web site" or "website"):ti,ab,kw	184801
#37	(("distance" NEXT counsel?ing) or ("distance" NEXT consultation*) or ("e" NEXT consultation*) or econsultation* or ("e" NEXT counsel?ing) or ecounsel?ing or ("e" NEXT health*) or ehealth* or "e therapies" or "e therapy" or etherap* or ("e" NEXT visit*) or evisit* or "m health" or "mhealth" or ("mobile" NEXT counsel?ing) or ("mobile" NEXT consultation*) or ("remote" NEXT consultation*) or ("remote" NEXT counsel?ing) or ("tele" NEXT health*) or telehealth* or ("tele" NEXT consultation*) or teleconsultation* or "tele medicine" or "telemedicine" or "tele rehabilitation" or "telerehabilitation"):ti,ab,kw	12716
#38	#28 or #29 or #30 or #31 or #32 or #33 or #34 or #35 or #36 or #37	188389
#39	#4 and #27 and #38	6085
#40	(clinicaltrials or trialsearch):so	444610
#41	#39 NOT #40	3156
	Limit to publication date 1995-2023 in Trials	3068

3. Web of Science Core Collection

Interface: Clarivate Analytics<br><br>Date of Search: 3 February 2023<br><br>Number of hits: 3,010	Field labels<br><br>• TS/Topic = title, abstract, author keywords and Keywords Plus<br>• NEAR/x = within x words, regardless of order<br>• * = truncation of word for alternate endings<br><br>Note: sometimes “quotation marks” are needed for single search terms to avoid automatic term mapping (lemmatization).

18 #14 AND #11 AND #8 AND #3

Refined by: LANGUAGES: (ENGLISH) AND [excluding] PUBLICATION YEARS: (1994 OR 1993) AND DOCUMENT TYPES: (ARTICLE OR CORRECTION OR EARLY ACCESS)

17 #14 AND #11 AND #8 AND #3

Refined by: LANGUAGES: (ENGLISH) AND [excluding] PUBLICATION YEARS: (1994 OR 1993)

16 #14 AND #11 AND #8 AND #3

Refined by: LANGUAGES: (ENGLISH)

15 #14 AND #11 AND #8 AND #3

14 #12 NOT #13

13 TI=(“systematic review” or “meta-analy*”)

12 TI=(“randomi$ed” OR “randomi$ed” OR “randomi$ation” OR “randomi$ation” OR placebo* OR (random* AND (allocat* OR assign*)) OR (blind* AND (“single” OR “double” OR “treble” OR “triple”))) OR AB=(“randomi$ed” OR “randomi$ed” OR “randomi$ation” OR “randomi$ation” OR placebo* OR (random* AND (allocat* OR assign*)) OR (blind* AND (“single” OR “double” OR “treble” OR “triple”)))

11 #10 OR #9

10 TS=(“distance counsel$ing” or “distance consultation*” or “e consultation*” or econsultation* or “e counsel$ing” or “ecounsel$ing” or “e health*” or ehealth* or “e therapies” or “e therapy” or etherap* or “e visit*” or evisit* or “m health” or “mhealth” or “mobile counsel$ing” or “mobile consultation*” or “remote consultation*” or “remote counsel$ing” or “tele health*” or telehealth* or “tele consultation*” or teleconsultation* or “tele medicine” or “telemedicine” or “tele rehabilitation” or “telerehabilitation")

9 TS=(“app” or “apps” or cellphone* or computer* or “digital” or “e mail” or “email” or internet* or “mobile application*” or “online” or phone* or smartphone* or technolog* or telephone* or “text message*” or video* or “virtual” or “web based” or “web site” or “website”)

8 #7 OR #6

7 TS=((”emotional” NEAR/1 disorder*) or transdiagnost* or “unified protocol”)

6 #5 AND #4

5 TS=(depress* or ((“affective” or “mood”) NEAR/1 disorder*))

4 TS=(agoraphobi* or anxi* or “obsessive-compulsive” or “panic” or phobi* or “post-traumatic stress” or “posttraumatic stress”)

3 #2 OR #1

2 TS=((“behavio$ral activation” or “bias modification” or “cognitive restructuring” or hypno* or “meditation” or “mindfulness” or “problem solving” or psychoeducation* or psychotherap* or “relaxation” or “self-management” or “unified protocol”))

1 TS=((("acceptance and commitment" or “affect-focused” or behavio$r* or “biofeedback” or computeri* or cognit* or “digital” or "emotional awareness and expression" or educat* or “exposure” or “guided” or internet* or “interpersonal” or “mobile” or “online” or “positive psychology” or “psychodynamic” or psychologic* or “self-guided” or “self-help” or transdiagnost* or web*) NEAR/1 (intervention* or therap* or treat* or program*)))

4. Psycinfo

Interface: EBSCO<br><br>Date of Search: 6 february 2023<br><br>Number of hits: 1,395	Field labels<br><br>• DE = subject heading<br>• TI = title<br>• AB = abstract<br>• KW = author keywords<br>• Nx = within x words, regardless of order<br>• * = truncation of word for alternate endings

S47	S45 NOT S36<br><br>Publication year: 1995-2023<br>Language: - english	1,395
S45	S44 NOT S36	1,418
S44	S7 AND S32 AND S35 AND S43	1,572
S43	S39 OR S42	135,063
S42	S40 AND S41	125,758
S41	S17 OR S18 OR S19 OR S20 OR S21	375,079
S40	S8 OR S9 OR S10 OR S11 OR S12 OR S13 OR S14 OR S15 OR S16	332,986
S39	S37 OR S38	12,089
S38	TI (transdiagnost* or "unified protocol") OR AB (transdiagnost* or "unified protocol") OR KW (transdiagnost* or "unified protocol")	4,261
S37	TI emotional N2 disorder* OR AB emotional N2 disorder* OR KW emotional N2 disorder*	8,225
S36	TI "systematic review" or meta-analy*	50,479
S35	S33 OR S34	206,233
S34	AB randomi?ed OR AB placebo OR AB randomly OR TI trial	206,178
S33	DE "Randomized Controlled Trials" OR DE "Randomized Clinical Trials"	1,387
S32	S23 OR S24 OR S25 OR S26 OR S27 OR S28 OR S29 OR S30 OR S31	504,430
S31	TI (“distance counsel#ing” or “distance consultation*” or “e consultation*” or econsultation* or “e counsel#ing” or “ecounsel#ing” or “e health*” or ehealth* or “e therapies” or “e therapy” or etherap* or “e visit*” or evisit* or “m health” or “mhealth” or “mobile counsel#ing” or “mobile consultation*” or “remote consultation*” or “remote counsel#ing” or “tele health*” or telehealth* or “tele consultation*” or teleconsultation* or “tele medicine” or “telemedicine” or “tele rehabilitation” or “telerehabilitation") OR AB (“distance counsel#ing” or “distance consultation*” or “e consultation*” or econsultation* or “e counsel#ing” or “ecounsel#ing” or “e health*” or ehealth* or “e therapies” or “e therapy” or etherap* or “e visit*” or evisit* or “m health” or “mhealth” or “mobile counsel#ing” or “mobile consultation*” or “remote consultation*” or “remote counsel#ing” or “tele health*” or telehealth* or “tele consultation*” or teleconsultation* or “tele medicine” or “telemedicine” or “tele rehabilitation” or “telerehabilitation") OR KW (“distance counsel#ing” or “distance consultation*” or “e consultation*” or econsultation* or “e counsel#ing” or “ecounsel#ing” or “e health*” or ehealth* or “e therapies” or “e therapy” or etherap* or “e visit*” or evisit* or “m health” or “mhealth” or “mobile counsel#ing” or “mobile consultation*” or “remote consultation*” or “remote counsel#ing” or “tele health*” or telehealth* or “tele consultation*” or teleconsultation* or “tele medicine” or “telemedicine” or “tele rehabilitation” or “telerehabilitation")	10,426
S30	TI (app or apps or cellphone* or computer* or digital or “e mail” or email or internet* or “mobile application*” or online or phone* or smartphone* or technolog* or telephone* or “text message*” or video* or virtual or “web based” or “web site” or website) OR AB (app or apps	498,437

	or cellphone* or computer* or digital or “e mail” or email or internet* or “mobile application*” or online or phone* or smartphone* or technolog* or telephone* or “text message*” or video* or virtual or “web based” or “web site” or website) OR KW (app or apps or cellphone* or computer* or digital or “e mail” or email or internet* or “mobile application*” or online or phone* or smartphone* or technolog* or telephone* or “text message*” or video* or virtual or “web based” or “web site” or website)	
S29	DE "Mobile Applications"	2,881
S28	DE "Videoconferencing"	1,138
S27	DE "Digital Technology"	1,488
S26	DE "Telemedicine" OR DE "Online Therapy" OR DE "Teleconferencing" OR DE "Teleconsultation" OR DE "Telepsychiatry" OR DE "Telepsychology" OR DE "Telerehabilitation" OR DE "Teleconferencing" OR DE "Videoconferencing"	13,791
S25	DE "Computer Assisted Therapy"	1,211
S24	DE "Digital Interventions"	1,160
S23	DE "Internet"	38,434
S22	S17 OR S18 OR S19 OR S20 OR S21	375,079
S21	TI (((affective or mood) N2 disorder*)) OR AB (((affective or mood) N2 disorder*)) OR KW (((affective or mood) N2 disorder*))	41,246
S20	TI depress* OR AB depress* OR KW depress*	349,996
S19	DE "Depression (Emotion)"	26,821
S18	DE "Major Depression" OR DE "Anaclitic Depression" OR DE "Dysthymic Disorder" OR DE "Endogenous Depression" OR DE "Late Life Depression" OR DE "Postpartum Depression" OR DE "Reactive Depression" OR DE "Recurrent Depression" OR DE "Treatment Resistant Depression"	154,908
S17	DE "Affective Disorders"	15,668
S16	TI (agoraphobi* or anxi* or obsessive-compulsive or panic or phobi* or “post-traumatic stress” or “posttraumatic stress”)) OR AB (agoraphobi* or anxi* or obsessive-compulsive or panic or phobi* or “post-traumatic stress” or “posttraumatic stress”)) OR KW (agoraphobi* or anxi* or obsessive-compulsive or panic or phobi* or “post-traumatic stress” or “posttraumatic stress”))	310,917
S15	DE "Anxiety"	94,782
S14	DE "Posttraumatic Stress Disorder"	38,806
S13	DE "Phobias" OR DE "Acrophobia" OR DE "Agoraphobia" OR DE "Claustrophobia" OR DE "Ophidiophobia" OR DE "School Phobia" OR DE "Social Phobia"	14,824
S12	DE "Panic Disorder"	9,312
S11	DE "Obsessive Compulsive Disorder"	15,724
S10	DE "Neurosis"	7,143
S9	DE "Agoraphobia"	3,756
S8	DE "Anxiety Disorders"	34,816
S7	S1 OR S2 OR S3 OR S4 OR S5 OR S6	579,216
S6	TI (“behavio#ral activation” or “bias modification” or “cognitive restructuring” or hypno* or meditation or mindfulness or “problem solving” or psychoeducation* or psychotherap* or relaxation or self-management or “unified protocol”) OR AB (“behavio#ral activation” or “bias modification” or “cognitive restructuring” or hypno* or meditation or mindfulness or “problem solving” or psychoeducation* or psychotherap* or relaxation or self-management or “unified protocol”) OR KW (“behavio#ral activation” or “bias modification” or “cognitive restructuring” or hypno* or meditation or mindfulness or “problem solving” or psychoeducation* or psychotherap* or relaxation or self-management or “unified protocol”)	256,300
S5	TI ((("acceptance and commitment" or affect-focused or behavio#r* or biofeedback or computeri* or cognit* or digital or "emotional awareness and expression" or educat* or exposure or guided or internet* or interpersonal or mobile or online or positive psychology or psychodynamic or psychologic* or self-guided or self-help or transdiagnost* or web*) N2	229,986

	(intervention* or therap* or treat* or program*))) OR AB ((("acceptance and commitment" or affect-focused or behavio#r* or biofeedback or computeri* or cognit* or digital or "emotional awareness and expression" or educat* or exposure or guided or internet* or interpersonal or mobile or online or positive psychology or psychodynamic or psychologic* or self-guided or self-help or transdiagnost* or web*) N2 (intervention* or therap* or treat* or program*))) OR KW ((("acceptance and commitment" or affect-focused or behavio#r* or biofeedback or computeri* or cognit* or digital or "emotional awareness and expression" or educat* or exposure or guided or internet* or interpersonal or mobile or online or positive psychology or psychodynamic or psychologic* or self-guided or self-help or transdiagnost* or web*) N2 (intervention* or therap* or treat* or program*)))	
S4	DE "Cognitive Behavior Therapy" OR DE "Acceptance and Commitment Therapy" OR DE "Cognitive Processing Therapy" OR DE "Prolonged Exposure Therapy"	26,182
S3	DE "Behavior Therapy" OR DE "Aversion Therapy" OR DE "Conversion Therapy" OR DE "Dialectical Behavior Therapy" OR DE "Exposure Therapy" OR DE "Implosive Therapy" OR DE "Reciprocal Inhibition Therapy" OR DE "Response Cost" OR DE "Systematic Desensitization Therapy" OR DE "Aversion Therapy" OR DE "Covert Sensitization" OR DE "Exposure Therapy" OR DE "Imaginal Exposure" OR DE "Implosive Therapy" OR DE "In Vivo Exposure" OR DE "Prolonged Exposure Therapy" OR DE "Systematic Desensitization Therapy" OR DE "Virtual Reality Exposure Therapy"	34,636
S2	DE "Cognitive Therapy"	24,241
S1	DE "Psychotherapy" OR DE "Adlerian Psychotherapy" OR DE "Adolescent Psychotherapy" OR DE "Affirmative Therapy" OR DE "Analytical Psychotherapy" OR DE "Autogenic Training" OR DE "Brief Psychotherapy" OR DE "Brief Relational Therapy" OR DE "Child Psychotherapy" OR DE "Client Centered Therapy" OR DE "Conversion Therapy" OR DE "Couples Therapy" OR DE "Eclectic Psychotherapy" OR DE "Emotion Focused Therapy" OR DE "Existential Therapy" OR DE "Experiential Psychotherapy" OR DE "Expressive Psychotherapy" OR DE "Eye Movement Desensitization Therapy" OR DE "Feminist Therapy" OR DE "Geriatric Psychotherapy" OR DE "Gestalt Therapy" OR DE "Group Psychotherapy" OR DE "Guided Imagery" OR DE "Humanistic Psychotherapy" OR DE "Hypnotherapy" OR DE "Individual Psychotherapy" OR DE "Insight Therapy" OR DE "Integrative Psychotherapy" OR DE "Interpersonal Psychotherapy" OR DE "Logotherapy" OR DE "Narrative Therapy" OR DE "Network Therapy" OR DE "Persuasion Therapy" OR DE "Primal Therapy" OR DE "Psychoanalysis" OR DE "Psychodrama" OR DE "Psychodynamic Psychotherapy" OR DE "Psychotherapeutic Counseling" OR DE "Psychotherapeutic Techniques" OR DE "Rational Emotive Behavior Therapy" OR DE "Reality Therapy" OR DE "Relationship Therapy" OR DE "Solution Focused Therapy" OR DE "Strategic Therapy" OR DE "Supportive Psychotherapy" OR DE "Transactional Analysis" OR DE "Adolescent Psychotherapy" OR DE "Multisystemic Therapy" OR DE "Child Psychotherapy" OR DE "Play Therapy" OR DE "Gestalt Therapy" OR DE "Empty Chair Technique" OR DE "Group Psychotherapy" OR DE "Encounter Group Therapy" OR DE "Therapeutic Community" OR DE "Humanistic Psychotherapy" OR DE "Client Centered Therapy" OR DE "Hypnotherapy" OR DE "Age Regression (Hypnotic)" OR DE "Ericksonian Psychotherapy" OR DE "Posthypnotic Suggestions" OR DE "Integrative Psychotherapy" OR DE "Schema Therapy" OR DE "Psychoanalysis" OR DE "Adlerian Psychotherapy" OR DE "Brief Relational Therapy" OR DE "Dream Analysis" OR DE "Self-Analysis" OR DE "Psychotherapeutic Counseling" OR DE "Family Therapy" OR DE "Psychotherapeutic Techniques" OR DE "Active Listening" OR DE "Animal Assisted Therapy" OR DE "Autogenic Training" OR DE "Brief Relational Therapy" OR DE "Centering" OR DE "Cotherapy" OR DE "Dream Analysis" OR DE "Empty Chair Technique" OR DE "Ericksonian Psychotherapy" OR DE "Free Association" OR DE "Guided Imagery" OR DE "Life Review" OR DE "Mirroring" OR DE "Morita Therapy" OR DE "Motivational Interviewing" OR DE "Mutual Storytelling Technique" OR DE "Network Therapy" OR DE "Paradoxical Techniques" OR DE "Psychodrama" OR DE "Strategic Therapy" OR DE "Strategic Family Therapy"	237,062

Kolaas K, *et al. BMJ Open* 2024; 14:e075796. doi: 10.1136/bmjopen-2023-075796
