# Supplementary material for: Internet-delivered transdiagnostic psychological treatments for individuals with depression, anxiety or both: a systematic review with meta-analysis of randomised controlled trials
Source: BMJ Open. 2024 Apr 3;14(4):e075796. doi: 10.1136/bmjopen-2023-075796 (PMC11015301; doi:10.1136/bmjopen-2023-075796)
Supplement: Supplementary data [file bmjopen-2023-075796supp002.pdf]

Internet-delivered transdiagnostic psychological treatments for individuals with depression, anxiety, or both: a systematic review with meta-analysis of randomized controlled trials

Randomized controlled trials per year of publication

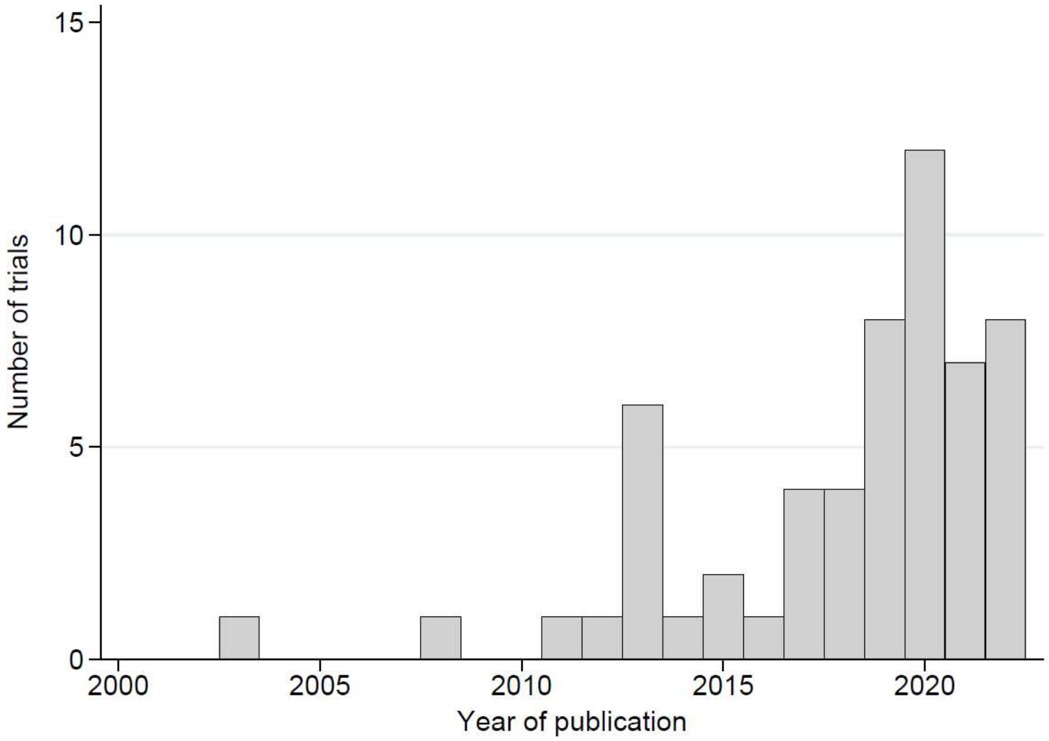

Internet-delivered transdiagnostic psychological treatments for individuals with depression, anxiety, or both: a systematic review with meta-analysis of randomized controlled trials

Exclusion criteria discussed with regard to disagreements

There were 45 disagreements:

- 18 concerned criterion d, i.e., whether the study recruited a mix of participants suffering from clinically significant depression and anxiety
- 15 concerned criterion c, i.e., whether the study evaluated the efficacy of an internet-delivered transdiagnostic psychological treatment for anxiety and depression
- 6 concerned criterion e, i.e., whether the sample composition was such that it was unreasonable to generalize to typical patients with depression and anxiety disorders
- 4 concerned criterion b, i.e., whether the text under consideration was a primary publication from a randomized controlled trial
- 1 concerned criterion a, i.e., whether the text under consideration was a peer reviewed article written in English
- 1 concerned criterion f, i.e., whether there was at least 10 participants in at least one internet-delivered transdiagnostic psychological treatment arm

Internet-delivered transdiagnostic psychological treatments for individuals with depression, anxiety, or both: a systematic review with meta-analysis of randomized controlled trials

Trial characteristics

Table S.B.1. Trial characteristics including references

Reference	Year	Country	Age, M	Women, %	N	Duration	Format	Treatment type/school	Missing, %
Al-Refae, et al. [1]	2021	Canada	25	79	245	< 6 weeks	app	mixed CBT/mindfulness	41
Batterham, et al. [2]	2021	Australia		85	1986	< 6 weeks	mixed/other	CBT	66
Beshai, et al. [3]	2020	Canada	35	44	456	< 6 weeks	mixed/other	mindfulness based	65
Borjalilu, et al. [4]	2019	Iran	24	71	68	≥ 6 weeks	app	mindfulness based	
Carolan, et al. [5]	2017	United Kingdom	41	85	84	≥ 6 weeks	website	CBT	26
Dear, et al. [6]	2018	Australia	21	82	217	< 6 weeks	website	CBT	28
Diaz-Garcia, et al. [7]	2021	Spain	34	72	216	≥ 6 weeks	mixed/other	CBT	32
Farrer, et al. [8]	2019	Australia	22	78	200	≥ 6 weeks	mixed/other	mixed CBT/mindfulness	28
Fitzpatrick, et al. [9]	2017	United States	22	67	70	< 6 weeks	mixed/other	CBT	20
Gonzalez-Robles, et al. [10]	2020	Spain	38	69	214	≥ 6 weeks	website	CBT	39
Graham, et al. [11]	2020	United States	42	82	146	≥ 6 weeks	app	CBT	4
Gregoire, et al. [12]	2022	Canada	26	77	107	< 6 weeks	video	CBT	31
Hadjistavropoulos, et al. [13]	2017	Canada	38	79	182	≥ 6 weeks	website	CBT	25
Hadjistavropoulos, et al. [14]	2020a	Canada	37	76	744	≥ 6 weeks	website	CBT	31
Hadjistavropoulos, et al. [15]	2020b	Canada	37	72	631	≥ 6 weeks	website	CBT	35
Hadjistavropoulos, et al. [16]	2022	Canada	36	76	469	≥ 6 weeks	website	CBT	27
Hensel, et al. [17]	2019	Canada	41	73, 78	812	≥ 6 weeks	website	CBT	45
Hirsch, et al. [18]	2020	United Kingdom	29	83	178	< 6 weeks	website	other	12
Hoek, et al. [19]	2012	The Netherlands	16	76	45	< 6 weeks	website	other	38
Hosseinzadeh Asl [20]	2022	Turkey	33	55	59	< 6 weeks	video	mindfulness based	17
Isbăşoiu, et al. [21]	2021	Romania	33	85	284	≥ 6 weeks	website	CBT	45

Internet-delivered transdiagnostic psychological treatments for individuals with depression, anxiety, or both: a systematic review with meta-analysis of randomized controlled trials

Johansson, et al. [22]	2013	Sweden	45	82	100	≥ 6 weeks	website	other	0
Kang, et al. [23]	2022	China	37	53	100	< 6 weeks	mixed/other	mindfulness based	0
Kladnitski, et al. [24]	2020	Australia	39	86	158	≥ 6 weeks	website	mixed CBT/mindfulness	27
Kleiboer, et al. [25]	2015	The Netherlands	45	65	537	< 6 weeks	website	other	22
Krafft, et al. [26]	2019	United States	25	66	35	< 6 weeks	app	CBT	60
Levin, et al. [27]	2022	United States	20	100	23	< 6 weeks	app	mindfulness based	30
Lindegaard, et al. [28]	2021	Sweden	38	42	59	≥ 6 weeks	website	CBT	39
McCloud, et al. [29]	2020	United Kingdom	24	83	168	≥ 6 weeks	app	CBT	42
Mohr, et al. [30]	2019	United States	36	76	301	≥ 6 weeks	app	CBT	3
Mullin, et al. [31]	2015	Australia	28	64	55	≥ 6 weeks	website	CBT	24
Newby, et al. [32]	2013	Australia	44	78	109	≥ 6 weeks	website	CBT	12
Nilsson, et al. [33]	2019	Sweden			837	≥ 6 weeks	website	other	67
Osborn, et al. [34]	2020	Kenya	15	64	103	< 6 weeks	website	other	0
Peynenburg, et al. [35]	2022	Canada	24	81	308	< 6 weeks	website	CBT	29
Powell, et al. [36]	2012	United Kingdom	41	78	3070	≥ 6 weeks	website	CBT	42
Proudfoot, et al. [37]	2013	Australia	39	70	720	≥ 6 weeks	website	other	28
Robichaud, et al. [38]	2020	Canada	35	87	63	≥ 6 weeks	website	CBT	32
Rogers and Sicouri [39]	2022	Australia	20	55	45	< 6 weeks	website	other	3
Rollman, et al. [40]	2018	United States	43	80	704	≥ 6 weeks	website	CBT	14
Schaeuffele, et al. [41]	2022	Germany	37	68	132	≥ 6 weeks	website	CBT	25
Sethi [42]	2013	Australia	20	58	89	< 6 weeks	website	CBT	0
Soucy, et al. [43]	2021	Canada	38	75	480	≥ 6 weeks	website	CBT	30
Southwell and Gould [44]	2016	Australia	34	88	120	< 6 weeks	website	other	52
Taylor, et al. [45]	2022	United Kingdom	41	83	2182	< 6 weeks	app	mindfulness based	35
Terides, et al. [46]	2018	Australia	45	88, 75	148	≥ 6 weeks	website	CBT	18
Titov, et al. [47]	2011	Australia	44	73	77	≥ 6 weeks	website	CBT	10

Internet-delivered transdiagnostic psychological treatments for individuals with depression, anxiety, or both: a systematic review with meta-analysis of randomized controlled trials

Titov, et al. [48]	2013	Australia	41	74	274	≥ 6 weeks	website	CBT	20
Titov, et al. [49]	2016	Australia	66	64	459	≥ 6 weeks	website	CBT	9
Tönnies, et al. [50]	2021	Germany	49	70	50	≥ 6 weeks	video	other	10
Tulbure, et al. [51]	2018	Romania	34	81	105	≥ 6 weeks	website	CBT	8
Twomey, et al. [52]	2014	Ireland	35	74	201	< 6 weeks	website	CBT	67
van Straten, et al. [53]	2008	The Netherlands	45	71	213	< 6 weeks	website	other	17
Villemaire-Krajden and Myhr [54]	2019	Canada	38	67	67	≥ 6 weeks	mixed/other	CBT	31
Viravan, et al. [55]	2022	Thailand	34	32	80	< 6 weeks	mixed/other	mindfulness based	4
Viskovich and Pakenham [56]	2020	Australia	27	68	2110	< 6 weeks	mixed/other	CBT	68
Zetterqvist, et al. [57]	2003	Sweden	39	57, 65	100	≥ 6 weeks	website	CBT	37

Internet-delivered transdiagnostic psychological treatments for individuals with depression, anxiety, or both: a systematic review with meta-analysis of randomized controlled trials

Publication bias

We explored indicators of publication bias in the meta-analysis of effects versus rudimentary passive controls. For depression, the funnel plot (see below) appeared to indicate that small- to medium-sized trials with non-beneficial effects of internet-delivered psychological treatments were underreported. This asymmetry was confirmed by Egger's test ($z = 2.24$, $p = 0.025$). The trim-and-fill procedure resulted in different outcomes depending on the choice of estimator. With the R0 estimator ($p < 0.001$), 19 studies were imputed, and the between-group effect dropped from 0.52 to 0.29 (95% CI 0.16-0.42). In contrast, the L0 and Q0 estimators resulted in no imputation. Visual inspection of the funnel plot for anxiety (see below) was also indicative of possible asymmetry in the same direction, but this was more ambiguous. Egger's test was significant ($z = 2.64$, $p = 0.008$) but the trim-and-fill procedure did not result in imputation, regardless of estimator. As is recommended in high heterogeneity situations,[58] we repeated all tests of publication bias in a manner informed by the moderator analyses and results were similar (see below).

Internet-delivered transdiagnostic psychological treatments for individuals with depression, anxiety, or both: a systematic review with meta-analysis of randomized controlled trials

Meta-analysis versus rudimentary passive controls, with depression symptoms as outcome

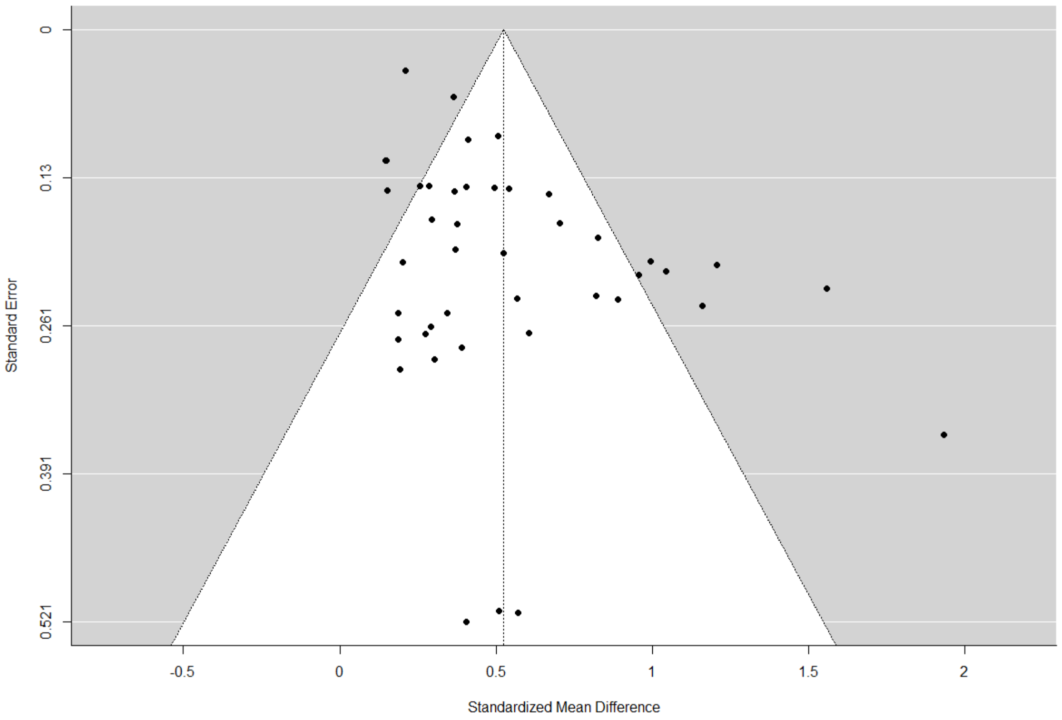

Internet-delivered transdiagnostic psychological treatments for individuals with depression, anxiety, or both: a systematic review with meta-analysis of randomized controlled trials

Meta-analysis versus rudimentary passive controls, with depression symptoms as outcome, with missing trials imputed based on the trim-and-fill procedure and the R0 estimator

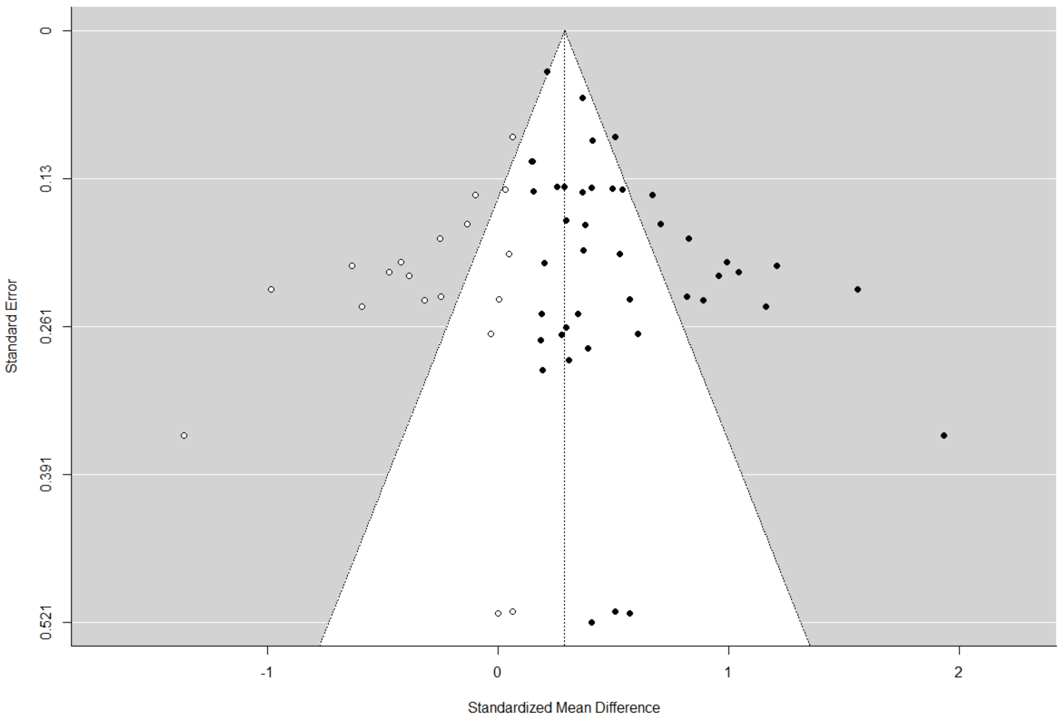

Internet-delivered transdiagnostic psychological treatments for individuals with depression, anxiety, or both: a systematic review with meta-analysis of randomized controlled trials

Meta-analysis versus rudimentary passive controls, with anxiety as outcome

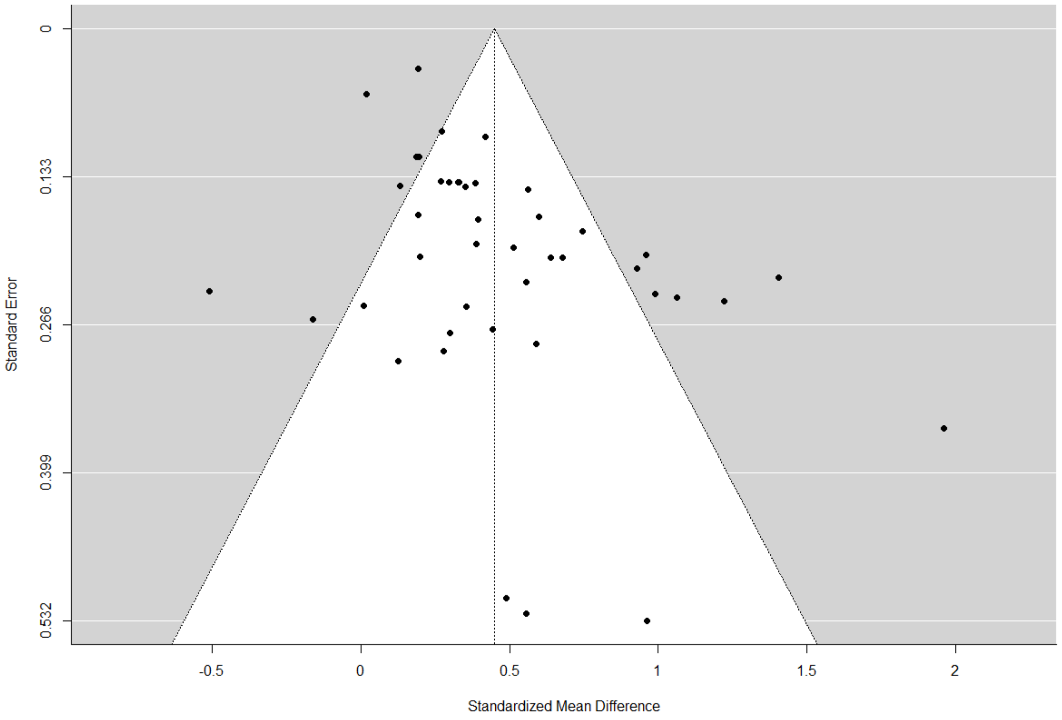

Internet-delivered transdiagnostic psychological treatments for individuals with depression, anxiety, or both: a systematic review with meta-analysis of randomized controlled trials

Risk of bias re-assessed separately based on whether trials required participants to meet full criteria for formal psychiatric diagnosis or not

Asymmetry of the funnel plot may result from several mechanisms. Because heterogeneity in the main analyses was substantial, and the moderator analyses indicated that the only significant moderator of the between-group effect versus rudimentary passive controls was whether trials required all participants to meet full criteria for a formal diagnosis or not, we repeated our investigation of the funnel plot for these substrata of the trials. That is, we first assessed whether publication bias appeared to be present in trials that employed psychiatric diagnoses, and then assessed whether publication bias appeared to be present in trials that did not employ psychiatric diagnoses. The results are tabulated in the table below. Overall, even when the sample of trials was split along this only significant effect moderator (i.e., psychiatric diagnoses or not), the outcome of the analyses of publication bias was still contradictory and similar to the equivalent tests of all trials.

Table S.B.2. Sensitivity analyses pertaining to publication bias

**Subset of trials**	**Outcome**	**Visual inspection**	**Egger's test**	**Trim-and-fill**
Psychiatric diagnosis (k = 12)	Depression	Possibly slight bias, but not clear	z = 3.67, p < 0.001	R0: p = 0.250, but 1 trial imputed<br>L0: no imputation<br>Q0: no imputation
Psychiatric diagnosis (k = 12)	Anxiety	No clear sign of bias	z = 2.08, p = 0.038	R0: p = 0.500, no imputation<br>L0: no imputation<br>Q0: no imputation
Cut-off or symptoms (k = 31)	Depression	Possibly slight bias, but not clear	z = 0.82, p = 0.410	R0: p = 0.004, and 7 trials imputed<br>L0: no imputation<br>Q0: no imputation
Cut-off or symptoms (k = 31)	Anxiety	Possibly slight bias, but not clear	z = 2.31, p = 0.021	R0: p = 0.500, no imputation<br>L0: 9 trials imputed<br>Q0: 12 trials imputed

Internet-delivered transdiagnostic psychological treatments for individuals with depression, anxiety, or both: a systematic review with meta-analysis of randomized controlled trials

Non-planned sensitivity analyses of psychiatric diagnosis as an effect moderator

One moderator was found to be significant for both depression and anxiety symptoms: trials that required all participants to meet full criteria for a psychiatric diagnosis reported a larger pooled between-group effects versus rudimentary passive controls, as compared to trials that did not have this requirement. We conducted a non-planned sensitivity analysis to ensure that this effect was not driven merely by participants in control groups of the routine clinic being more likely to be enrolled other treatments, by testing the moderating effect of diagnosis on the pooled within-group effect. This test illustrated that psychiatric diagnosis remained a significant effect moderator also with regard to within-group effects (depression: 1.10 vs. 0.75, $p = 0.001$; anxiety: 1.14 vs. 0.63, $p < 0.001$). We also reviewed studies that used the most common depression and anxiety outcome respectively (the PHQ-9 and GAD-7) to determine if the effect of diagnosis was driven primarily by a difference in the mean symptom reduction, or by a smaller standard deviation. Though the average standard deviation was slightly smaller in studies that required participants to meet full criteria for a diagnosis, the reduction in the mean was also larger. In summary, requiring participants to meet full criteria for psychiatric diagnosis appeared to be robust effect moderator.

Internet-delivered transdiagnostic psychological treatments for individuals with depression, anxiety, or both: a systematic review with meta-analysis of randomized controlled trials

Randomized controlled trials versus other bona fide treatments

Two trials compared the effects of an internet-delivered transdiagnostic psychological treatment to those of a distinctly different bona fide treatment. One trial compared mindfulness-based stress reduction delivered via a mobile app (n = 28) to a face-to-face equivalent (n = 20) for university students and reported non-significant between-group effects (depression: g = -0.49; anxiety: g = -0.13).[4] One trial compared web-based CBT (n = 23) to a face-to-face equivalent (n = 21) for university students and reported a significant and large between-group effect on depression in favor of the face-to-face treatment (g = -1.39) and a non-significant and small between-group effect on anxiety (g = -0.21).[42] Last, we also identified one trial that was on the verge of being a bona-fide comparison. In this trial, web-based CBT (n = 33) was compared to a condition which was described as a waitlist, but where participants had access to an unguided CBT bibliotherapy which we deemed to be relatively credible (n = 34). There were no significant between-group effects on depression (g = 0.33) or anxiety (g = -0.13).[54]

Internet-delivered transdiagnostic psychological treatments for individuals with depression, anxiety, or both: a systematic review with meta-analysis of randomized controlled trials

Randomized factorial trials

Nine randomized factorial trials explored moderators of the overall effect of internet-delivered transdiagnostic psychological treatments. Seven of these trials evaluated the same treatment protocol: the Wellbeing Course. The factors investigated, and the main outcome of each of the nine randomized factorial trials is tabulated below:

Table S.B.3. Randomized factorial trials of internet-delivered transdiagnostic psychological treatments suitable for both depression and anxiety

Reference	N	Protocol	Factors	Summary of main outcome
Dear *et al.* (2018)[6]	217	Wellbeing Course	Therapist-guided vs. unguided	Mostly no significant mean differences in the clinical outcome, with small between-group effects on depression and anxiety at post-treatment.
Hadjistavropoulos *et al.* (2017)[13]	182	Wellbeing Course	Weekly support vs. optional support	No significant mean differences in the clinical outcome, with small between-group effects on depression and anxiety at post-treatment. Rates of treatment completion were significantly higher with weekly support.
Hadjistavropoulos *et al.* (2020a)[14]	744	Wellbeing Course	Weekly support by ICBT specialist vs. weekly support plus on-demand support by ICBT specialist vs. weekly support by other clinician	Mostly no significant mean differences in the clinical outcome, with small between-group effects on depression and anxiety at post-treatment. Rates of response in depression were lower with support from other clinicians.
Hadjistavropoulos *et al.* (2020b)[15]	631	Wellbeing Course	Use of homework reflection vs. no use of homework reflection<br><br>Weekly support vs. twice-weekly support<br><br>ICBT clinic vs. community mental health clinic	No significant mean differences in the clinical outcome, with small between-group effects on depression and anxiety at post-treatment.
Hadjistavropoulos *et al.* (2022)[16]	469	Wellbeing Course	Optional 4-week extension of support vs. no such option<br><br>Optional booster lesson vs. no such option	No significant mean differences in the clinical outcome, with small between-group effects on depression and anxiety at post-treatment. About 52% opted to make use of the booster lesson.

Internet-delivered transdiagnostic psychological treatments for individuals with depression, anxiety, or both: a systematic review with meta-analysis of randomized controlled trials

Isbăşoiu *et al.* (2021)[21]	284	Unified protocol	Addition of self-enhancement and emotion regulation components vs. no such components	No significant mean differences in the clinical outcome, with small between-group effects on depression and anxiety at post-treatment.
Mohr *et al.* (2019)[30]	301	IntelliCare	Therapist-guided vs. unguided *<br><br>Weekly app recommendations vs. no such recommendations *	Guidance led to a significantly larger mean effect on anxiety (but not depression). Recommendations led to a significantly larger mean effect on depression (but not anxiety). Between-group effects were small.
Peynenburg *et al.* (2022)[35]	308	Wellbeing Course	Internet-delivered exercises based on motivational interviewing before treatment vs. no such exercises *<br><br>Optional booster lesson vs. no such option	Internet-delivered exercises based on motivational interviewing before treatment led to a significantly larger mean effect on depression and anxiety. Having the option of a booster lesson did not lead to significantly larger mean effects. Between-group effects were small. About 32% opted to make use of the booster lesson.
Soucy *et al.* (2021)[43]	480	Wellbeing Course	Internet-delivered exercises based on motivational interviewing before treatment vs. no such exercises	No significant mean differences in the clinical outcome, with small between-group effects on depression and anxiety at post-treatment.

* This factor was a significant moderator of the effect on depression symptoms or anxiety.

Internet-delivered transdiagnostic psychological treatments for individuals with depression, anxiety, or both: a systematic review with meta-analysis of randomized controlled trials

Brief interventions

Two trials evaluated brief interventions (≤2 weeks) for adults. One trial (*N*=70) found that 2 weeks of communication with an automated chatbot trained in active listening and CBT strategies was associated with a small to moderate between-group effect on depression (*g* = 0.37) but no significant between-group effect on anxiety as compared to a waitlist.[9] The second trial (*N*=45) reported null effects of a single session of cognitive bias modification on depression (*g* = -0.04) and anxiety (*g* = 0.42), compared to a psychological placebo.[39]

Internet-delivered transdiagnostic psychological treatments for individuals with depression, anxiety, or both: a systematic review with meta-analysis of randomized controlled trials

References

1. Al-Refae M, Al-Refae A, Munroe M, et al. A Self-Compassion and Mindfulness-Based Cognitive Mobile Intervention (Serene) for Depression, Anxiety, and Stress: Promoting Adaptive Emotional Regulation and Wisdom. *Front Psychol* 2021;12:648087. doi: 10.3389/fpsyg.2021.648087 [published Online First: 2021/04/09]
2. Batterham PJ, Calear AL, Farrer L, et al. Efficacy of a Transdiagnostic Self-Help Internet Intervention for Reducing Depression, Anxiety, and Suicidal Ideation in Adults: Randomized Controlled Trial. *J Med Internet Res* 2021;23(1):e22698. doi: 10.2196/22698 [published Online First: 2021/01/23]
3. Beshai S, Bueno C, Yu M, et al. Examining the effectiveness of an online program to cultivate mindfulness and self-compassion skills (Mind-OP): Randomized controlled trial on Amazon's Mechanical Turk. *Behav Res Ther* 2020;134:103724. doi: 10.1016/j.brat.2020.103724 [published Online First: 2020/09/18]
4. Borjalilu S, Mazaheri MA, Talebpour A. Effectiveness of Mindfulness-Based Stress Management in The Mental Health of Iranian University Students: A Comparison of Blended Therapy, Face-to-Face Sessions, and mHealth App (Aramgar). *Iranian Journal of Psychiatry and Behavioral Sciences* 2019;13(2) doi: 10.5812/ijpbs.84726
5. Carolan S, Harris PR, Greenwood K, et al. Increasing engagement with an occupational digital stress management program through the use of an online facilitated discussion group: Results of a pilot randomised controlled trial. *Internet Interv* 2017;10:1-11. doi: 10.1016/j.invent.2017.08.001 [published Online First: 2018/08/24]
6. Dear BF, Fogliati VJ, Fogliati R, et al. Treating anxiety and depression in young adults: A randomised controlled trial comparing clinician-guided versus self-guided Internet-delivered cognitive behavioural therapy. *Aust N Z J Psychiatry* 2018;52(7):668-79. doi: 10.1177/0004867417738055 [published Online First: 2017/10/25]
7. Diaz-Garcia A, Gonzalez-Robles A, Garcia-Palacios A, et al. Negative and Positive Affect Regulation in a Transdiagnostic Internet-Based Protocol for Emotional Disorders: Randomized Controlled Trial. *J Med Internet Res* 2021;23(2):e21335. doi: 10.2196/21335 [published Online First: 2021/02/02]
8. Farrer LM, Gulliver A, Katruss N, et al. A novel multi-component online intervention to improve the mental health of university students: Randomised controlled trial of the Uni Virtual Clinic. *Internet Interv* 2019;18:100276. doi: 10.1016/j.invent.2019.100276 [published Online First: 2020/01/01]
9. Fitzpatrick KK, Darcy A, Vierhile M. Delivering Cognitive Behavior Therapy to Young Adults With Symptoms of Depression and Anxiety Using a Fully Automated Conversational Agent (Woebot): A Randomized Controlled Trial. *JMIR Ment Health* 2017;4(2):e19. doi: 10.2196/mental.7785 [published Online First: 2017/06/08]
10. Gonzalez-Robles A, Diaz-Garcia A, Garcia-Palacios A, et al. Effectiveness of a Transdiagnostic Guided Internet-Delivered Protocol for Emotional Disorders Versus Treatment as Usual in Specialized Care: Randomized Controlled Trial. *J Med Internet Res* 2020;22(7):e18220. doi: 10.2196/18220 [published Online First: 2020/07/17]
11. Graham AK, Greene CJ, Kwasny MJ, et al. Coached Mobile App Platform for the Treatment of Depression and Anxiety Among Primary Care Patients: A Randomized Clinical Trial. *JAMA Psychiatry* 2020;77(9):906-14. doi: 10.1001/jamapsychiatry.2020.1011 [published Online First: 2020/05/21]
12. Gregoire S, Beaulieu F, Lachance L, et al. An online peer support program to improve mental health among university students: A randomized controlled trial. *J Am Coll*

Internet-delivered transdiagnostic psychological treatments for individuals with depression, anxiety, or both: a systematic review with meta-analysis of randomized controlled trials

Health 2022:1-13. doi: 10.1080/07448481.2022.2099224 [published Online First: 2022/08/10]

13. Hadjistavropoulos HD, Schneider LH, Edmonds M, et al. Randomized controlled trial of internet-delivered cognitive behaviour therapy comparing standard weekly versus optional weekly therapist support. *J Anxiety Disord* 2017;52:15-24. doi: 10.1016/j.janxdis.2017.09.006 [published Online First: 2017/10/02]
14. Hadjistavropoulos HD, Peynenburg V, Nugent M, et al. Transdiagnostic Internet-delivered cognitive behaviour therapy with therapist support offered once-weekly or once-weekly supplemented with therapist support within one-business-day: Pragmatic randomized controlled trial. *Internet Interv* 2020;22:100347. doi: 10.1016/j.invent.2020.100347 [published Online First: 20200831]
15. Hadjistavropoulos HD, Peynenburg V, Thiessen DL, et al. A pragmatic factorial randomized controlled trial of transdiagnostic internet-delivered cognitive behavioural therapy: Exploring benefits of homework reflection questionnaires and twice-weekly therapist support. *Internet Interv* 2020;22:100357. doi: 10.1016/j.invent.2020.100357 [published Online First: 20201201]
16. Hadjistavropoulos HD, Peynenburg V, Thiessen DL, et al. A randomized factorial trial of internet-delivered cognitive behavioural therapy: An 8-week program with or without extended support and booster lesson. *Internet Interv* 2022;27:100499. doi: 10.1016/j.invent.2022.100499 [published Online First: 20220206]
17. Hensel JM, Shaw J, Ivers NM, et al. A Web-Based Mental Health Platform for Individuals Seeking Specialized Mental Health Care Services: Multicenter Pragmatic Randomized Controlled Trial. *J Med Internet Res* 2019;21(6):e10838. doi: 10.2196/10838 [published Online First: 2019/06/06]
18. Hirsch CR, Krahe C, Whyte J, et al. Effects of modifying interpretation bias on transdiagnostic repetitive negative thinking. *J Consult Clin Psychol* 2020;88(3):226-39. doi: 10.1037/ccp0000455 [published Online First: 2020/02/19]
19. Hoek W, Schuurmans J, Koot HM, et al. Effects of Internet-based guided self-help problem-solving therapy for adolescents with depression and anxiety: a randomized controlled trial. *PLoS One* 2012;7(8):e43485. doi: 10.1371/journal.pone.0043485 [published Online First: 2012/09/07]
20. Hosseinzadeh Asl NR. A randomized controlled trial of a mindfulness-based intervention in social workers working during the COVID-19 crisis. *Curr Psychol* 2022;41(11):8192-99. doi: 10.1007/s12144-021-02150-3 [published Online First: 2021/08/17]
21. Isbăşoiu AB, Tulbure BT, Rusu A, et al. Can We Boost Treatment Adherence to an Online Transdiagnostic Intervention by Adding Self-Enhancement Strategies? Results From a Randomized Controlled Non-inferiority Trial. *Frontiers in psychology* 2021;12:752249. doi: 10.3389/fpsyg.2021.752249 [published Online First: 20211202]
22. Johansson R, Bjorklund M, Hornborg C, et al. Affect-focused psychodynamic psychotherapy for depression and anxiety through the Internet: a randomized controlled trial. *PeerJ* 2013;1:e102. doi: 10.7717/peerj.102 [published Online First: 2013/07/19]
23. Kang MY, Nan JKM, Yuan Y. Effectiveness of an online short-term audio-based mindfulness program on negative emotions during the COVID-19 pandemic: Latent growth curve analyses of anxiety and moderated mediation effects of anxiety between mindfulness and negative affect. *Curr Psychol* 2022:1-13. doi: 10.1007/s12144-022-03902-5 [published Online First: 2022/12/06]

Internet-delivered transdiagnostic psychological treatments for individuals with depression, anxiety, or both: a systematic review with meta-analysis of randomized controlled trials

24. Kladnitski N, Smith J, Uppal S, et al. Transdiagnostic internet-delivered CBT and mindfulness-based treatment for depression and anxiety: A randomised controlled trial. *Internet Interv* 2020;20:100310. doi: 10.1016/j.invent.2020.100310 [published Online First: 2020/03/07]
25. Kleiboer A, Donker T, Seekles W, et al. A randomized controlled trial on the role of support in Internet-based problem solving therapy for depression and anxiety. *Behav Res Ther* 2015;72:63-71. doi: 10.1016/j.brat.2015.06.013 [published Online First: 2015/07/21]
26. Krafft J, Potts S, Schoendorff B, et al. A Randomized Controlled Trial of Multiple Versions of an Acceptance and Commitment Therapy Matrix App for Well-Being. *Behav Modif* 2019;43(2):246-72. doi: 10.1177/0145445517748561 [published Online First: 2017/12/22]
27. Levin ME, Hicks ET, Krafft J. Pilot evaluation of the stop, breathe & think mindfulness app for student clients on a college counseling center waitlist. *J Am Coll Health* 2022;70(1):165-73. doi: 10.1080/07448481.2020.1728281 [published Online First: 2020/03/10]
28. Lindegaard T, Seaton F, Halaj A, et al. Internet-based cognitive behavioural therapy for depression and anxiety among Arabic-speaking individuals in Sweden: a pilot randomized controlled trial. *Cogn Behav Ther* 2021;50(1):47-66. doi: 10.1080/16506073.2020.1771414 [published Online First: 2020/07/01]
29. McCloud T, Jones R, Lewis G, et al. Effectiveness of a Mobile App Intervention for Anxiety and Depression Symptoms in University Students: Randomized Controlled Trial. *JMIR Mhealth Uhealth* 2020;8(7):e15418. doi: 10.2196/15418 [published Online First: 2020/08/01]
30. Mohr DC, Schueller SM, Tomasino KN, et al. Comparison of the Effects of Coaching and Receipt of App Recommendations on Depression, Anxiety, and Engagement in the IntelliCare Platform: Factorial Randomized Controlled Trial. *Journal of medical Internet research* 2019;21(8):e13609. doi: 10.2196/13609 [published Online First: 20190828]
31. Mullin A, Dear BF, Karin E, et al. The UniWellbeing course: A randomised controlled trial of a transdiagnostic internet-delivered cognitive behavioural therapy (CBT) programme for university students with symptoms of anxiety and depression. *Internet Interventions* 2015;2(2):128-36. doi: 10.1016/j.invent.2015.02.002
32. Newby JM, Mackenzie A, Williams AD, et al. Internet cognitive behavioural therapy for mixed anxiety and depression: a randomized controlled trial and evidence of effectiveness in primary care. *Psychol Med* 2013;43(12):2635. doi: 10.1017/S0033291713000111
33. Nilsson A, Sorman K, Klingvall J, et al. MyCompass in a Swedish context - lessons learned from the transfer of a self-guided intervention targeting mental health problems. *BMC Psychiatry* 2019;19(1):51. doi: 10.1186/s12888-019-2039-1 [published Online First: 2019/02/02]
34. Osborn TL, Rodriguez M, Wasil AR, et al. Single-session digital intervention for adolescent depression, anxiety, and well-being: Outcomes of a randomized controlled trial with Kenyan adolescents. *J Consult Clin Psychol* 2020;88(7):657-68. doi: 10.1037/ccp0000505 [published Online First: 2020/05/12]
35. Peynenburg V, Hadjistavropoulos H, Thiessen D, et al. Internet-Delivered Cognitive Behavioral Therapy for Postsecondary Students: Randomized Factorial Trial for Examining Motivational Interviewing and Booster Lessons. *Journal of medical*

Internet research 2022;24(9):e40637. doi: 10.2196/40637 [published Online First: 20220907]
36. Powell J, Hamborg T, Stallard N, et al. Effectiveness of a web-based cognitive-behavioral tool to improve mental well-being in the general population: randomized controlled trial. *J Med Internet Res* 2012;15(1):e2. doi: 10.2196/jmir.2240 [published Online First: 2013/01/11]
37. Proudfoot J, Clarke J, Birch MR, et al. Impact of a mobile phone and web program on symptom and functional outcomes for people with mild-to-moderate depression, anxiety and stress: a randomised controlled trial. *BMC Psychiatry* 2013;13:312. doi: 10.1186/1471-244X-13-312 [published Online First: 2013/11/19]
38. Robichaud M, Talbot F, Titov N, et al. Facilitating access to iCBT: a randomized controlled trial assessing a translated version of an empirically validated program using a minimally monitored delivery model. *Behav Cogn Psychother* 2020;48(2):185-202. doi: 10.1017/S135246581900047X [published Online First: 2019/08/17]
39. Rogers J, Sicouri G. A Single-Session Online Cognitive Bias Modification of Interpretations Modified for Adults With Anxiety and Depressive Symptoms. *Behav Ther* 2022;53(5):967-80. doi: 10.1016/j.beth.2022.04.006 [published Online First: 2022/08/21]
40. Rollman BL, Herbeck Belnap B, Abebe KZ, et al. Effectiveness of Online Collaborative Care for Treating Mood and Anxiety Disorders in Primary Care: A Randomized Clinical Trial. *JAMA Psychiatry* 2018;75(1):56-64. doi: 10.1001/jamapsychiatry.2017.3379 [published Online First: 2017/11/09]
41. Schaeuffele C, Homeyer S, Perea L, et al. The unified protocol as an internet-based intervention for emotional disorders: Randomized controlled trial. *PLoS One* 2022;17(7):e0270178. doi: 10.1371/journal.pone.0270178 [published Online First: 2022/07/12]
42. Sethi S. Treating Youth Depression and Anxiety: A Randomised Controlled Trial Examining the Efficacy of Computerised versus Face-to-face Cognitive Behaviour Therapy. *Australian Psychologist* 2013;48(4):249-57. doi: 10.1111/ap.12006
43. Soucy JN, Hadjistavropoulos HD, Karin E, et al. Brief online motivational interviewing pre-treatment intervention for enhancing internet-delivered cognitive behaviour therapy: A randomized controlled trial. *Internet Interv* 2021;25:100394. doi: 10.1016/j.invent.2021.100394 [published Online First: 20210420]
44. Southwell S, Gould E. A randomised wait list-controlled pre–post–follow-up trial of a gratitude diary with a distressed sample. *The Journal of Positive Psychology* 2016;12(6):579-93. doi: 10.1080/17439760.2016.1221127
45. Taylor H, Cavanagh K, Field AP, et al. Health Care Workers' Need for Headspace: Findings From a Multisite Definitive Randomized Controlled Trial of an Unguided Digital Mindfulness-Based Self-help App to Reduce Healthcare Worker Stress. *JMIR Mhealth Uhealth* 2022;10(8):e31744. doi: 10.2196/31744 [published Online First: 2022/08/26]
46. Terides MD, Dear BF, Fogliati VJ, et al. Increased skills usage statistically mediates symptom reduction in self-guided internet-delivered cognitive-behavioural therapy for depression and anxiety: a randomised controlled trial. *Cogn Behav Ther* 2018;47(1):43-61. doi: 10.1080/16506073.2017.1347195 [published Online First: 2017/07/21]

Internet-delivered transdiagnostic psychological treatments for individuals with depression, anxiety, or both: a systematic review with meta-analysis of randomized controlled trials

47. Titov N, Dear BF, Schwencke G, et al. Transdiagnostic internet treatment for anxiety and depression: a randomised controlled trial. *Behav Res Ther* 2011;49(8):441-52. doi: 10.1016/j.brat.2011.03.007 [published Online First: 2011/06/18]
48. Titov N, Dear BF, Johnston L, et al. Improving adherence and clinical outcomes in self-guided internet treatment for anxiety and depression: randomised controlled trial. *PLoS One* 2013;8(7):e62873. doi: 10.1371/journal.pone.0062873 [published Online First: 2013/07/12]
49. Titov N, Fogliati VJ, Staples LG, et al. Treating anxiety and depression in older adults: randomised controlled trial comparing guided v. self-guided internet-delivered cognitive-behavioural therapy. *BJPsych Open* 2016;2(1):50-58. doi: 10.1192/bjpo.bp.115.002139 [published Online First: 2016/10/06]
50. Tönnies J, Hartmann M, Wensing M, et al. Mental Health Specialist Video Consultations Versus Treatment-as-Usual for Patients With Depression or Anxiety Disorders in Primary Care: Randomized Controlled Feasibility Trial. *JMIR Ment Health* 2021;8(3):e22569. doi: 10.2196/22569 [published Online First: 2021/03/13]
51. Tulbure BT, Rusu A, Sava FA, et al. A Web-Based Transdiagnostic Intervention for Affective and Mood Disorders: Randomized Controlled Trial. *JMIR Ment Health* 2018;5(2):e36. doi: 10.2196/mental.8901 [published Online First: 2018/05/26]
52. Twomey C, O'Reilly G, Byrne M, et al. A randomized controlled trial of the computerized CBT programme, MoodGYM, for public mental health service users waiting for interventions. *Br J Clin Psychol* 2014;53(4):433-50. doi: 10.1111/bjc.12055 [published Online First: 2014/05/17]
53. van Straten A, Cuijpers P, Smits N. Effectiveness of a web-based self-help intervention for symptoms of depression, anxiety, and stress: randomized controlled trial. *J Med Internet Res* 2008;10(1):e7. doi: 10.2196/jmir.954 [published Online First: 2008/03/28]
54. Villemaire-Krajden R, Myhr G. Evaluating the Use of a Computerized CBT Program for Outpatients on a Waitlist in a University CBT Unit. *J Psychiatr Pract* 2019;25(4):268-78. doi: 10.1097/PRA.0000000000000396 [published Online First: 2019/07/11]
55. Viravan N, Atsariyasing W, Srifuengfung M, et al. Efficacy of Online Mindfulness Program 'Mindful Senses' for Depression and Anxiety Reduction in Community Samples: A Randomized Controlled Trial. *J Med Assoc Thai* 2022;105(11):1108-19. doi: 10.35755/jmedassocthai.2022.11.13700
56. Viskovich S, Pakenham KI. Randomized controlled trial of a web-based Acceptance and Commitment Therapy (ACT) program to promote mental health in university students. *J Clin Psychol* 2020;76(6):929-51. doi: 10.1002/jclp.22848 [published Online First: 2019/08/31]
57. Zetterqvist K, Maanmies J, Strom L, et al. Randomized controlled trial of internet-based stress management. *Cogn Behav Ther* 2003;32(3):151-60. doi: 10.1080/16506070302316 [published Online First: 2005/11/18]
58. Shi L, Lin L. The trim-and-fill method for publication bias: practical guidelines and recommendations based on a large database of meta-analyses. *Medicine* 2019;98(23)
