# Supplementary material for: Internet-delivered transdiagnostic psychological treatments for individuals with depression, anxiety or both: a systematic review with meta-analysis of randomised controlled trials
Source: BMJ Open. 2024 Apr 3;14(4):e075796. doi: 10.1136/bmjopen-2023-075796 (PMC11015301; doi:10.1136/bmjopen-2023-075796)
Supplement: Supplementary data [file bmjopen-2023-075796supp003.pdf]

**Outcome / reserch question**	**RCT design**	**1. Risk of B**	**2. Inconsis**	**3. Indirect**	**4. Impreci**
Adults vs. TAU/WLC (34 RCTs)	4	-2	-1	0	0
Adults vs. attention/engagement (8 RCTs)	4	-2	-2	0	-1
Adults vs. bona fide (3 RCTs)	4	-2	-2	0	-2
Children/adolescents (2 RCTs)	4	-1	-2	0	-2
Primary care (3 RCTs)	4	0	-2	0	-2

5. Publicat	6*. Large r	7*. Dose R	8*. Effect c	GRADE	Quality
-1	0	0	0	0	Very low
-1	0	0	0	-2	Very low
-1	0	0	0	-3	Very low
-1	0	0	0	-2	Very low
-1	0	0	0	-1	Very low
