# Supplementary material for: Internet-delivered transdiagnostic psychological treatments for individuals with depression, anxiety or both: a systematic review with meta-analysis of randomised controlled trials
Source: BMJ Open. 2024 Apr 3;14(4):e075796. doi: 10.1136/bmjopen-2023-075796 (PMC11015301; doi:10.1136/bmjopen-2023-075796)
Supplement: Author's manuscript [file bmjopen-2023-075796.draft_revisions.pdf]

BMJ Open

BMJ Open is committed to open peer review. As part of this commitment we make the peer review history of every article we publish publicly available.

When an article is published we post the peer reviewers' comments and the authors' responses online. We also post the versions of the paper that were used during peer review. These are the versions that the peer review comments apply to.

The versions of the paper that follow are the versions that were submitted during the peer review process. They are not the versions of record or the final published versions. They should not be cited or distributed as the published version of this manuscript.

BMJ Open is an open access journal and the full, final, typeset and author-corrected version of record of the manuscript is available on our site with no access controls, subscription charges or pay-per-view fees (http://bmjopen.bmj.com).

If you have any questions on BMJ Open's open peer review process please email info.bmjopen@bmj.com

Internet-delivered transdiagnostic psychological treatments for individuals with depression, anxiety, or both: a systematic review and meta-analysis of randomized controlled trials

Journal:	*BMJ Open*
Manuscript ID	bmjopen-2023-075796
Article Type:	Original research
Date Submitted by the Author:	18-May-2023
Complete List of Authors:	Kolaas, Karoline; Karolinska Institutet, Centre for Psychiatry Research, Department of Clinical Neuroscience; Region Stockholm, Liljeholmen Primary Health Care Center<br>Berman, Anne H.; Uppsala Universitet, Department of Psychology; Karolinska Institutet, Centre for Psychiatry Research, Department of Clinical Neuroscience<br>Hedman-Lagerlöf, Erik ; Karolinska Institute, Department of Clinical Neuroscience<br>Lindsäter, Elin; Karolinska Institute, Department of Clinical Neuroscience<br>Hybelius, Jonna; Karolinska Institutet, Division of Family Medicine and Primary Care, Department of Neurobiology, Care Sciences and Society; Region Stockholm, Liljeholmen academic primary care clinic<br>Axelsson, Erland; Karolinska Institutet, Division of Family Medicine and Primary Care, Department of Neurobiology, Care Sciences and Society; Region Stockholm, Liljeholmen academic primary care clinic
Keywords:	MENTAL HEALTH, PSYCHIATRY, Depression & mood disorders < PSYCHIATRY, Anxiety disorders < PSYCHIATRY, Patient Reported Outcome Measures, Systematic Review

SCHOLARONE™
Manuscripts

I, the Submitting Author has the right to grant and does grant on behalf of all authors of the Work (as defined in the below author licence), an exclusive licence and/or a non-exclusive licence for contributions from authors who are: i) UK Crown employees; ii) where BMJ has agreed a CC-BY licence shall apply, and/or iii) in accordance with the terms applicable for US Federal Government officers or employees acting as part of their official duties; on a worldwide, perpetual, irrevocable, royalty-free basis to BMJ Publishing Group Ltd ("BMJ") its licensees and where the relevant Journal is co-owned by BMJ to the co-owners of the Journal, to publish the Work in this journal and any other BMJ products and to exploit all rights, as set out in our licence.

The Submitting Author accepts and understands that any supply made under these terms is made by BMJ to the Submitting Author unless you are acting as an employee on behalf of your employer or a postgraduate student of an affiliated institution which is paying any applicable article publishing charge ("APC") for Open Access articles. Where the Submitting Author wishes to make the Work available on an Open Access basis (and intends to pay the relevant APC), the terms of reuse of such Open Access shall be governed by a Creative Commons licence – details of these licences and which Creative Commons licence will apply to this Work are set out in our licence referred to above.

Other than as permitted in any relevant BMJ Author's Self Archiving Policies, I confirm this Work has not been accepted for publication elsewhere, is not being considered for publication elsewhere and does not duplicate material already published. I confirm all authors consent to publication of this Work and authorise the granting of this licence.

For peer review only

Internet-delivered transdiagnostic psychological treatments for individuals with depression, anxiety, or both: a systematic review and meta-analysis of randomized controlled trials

Karoline Kolaas, MSc[a b c]; Anne H Berman, PhD[a d]; Erik Hedman-Lagerlöf, PhD[e f]; Elin Lindsäter, PhD[a e f]; Jonna Hybelius, MSc[b c g]; and Erland Axelsson, PhD[b c g]

Author Affiliations:[a] Centre for Psychiatry Research, Department of Clinical Neuroscience, Karolinska Institutet & Stockholm Health Care Services, Region Stockholm, Stockholm, Sweden (Ms Kolaas, Dr Berman, and Dr Lindsäter),[b] Liljeholmen Primary Health Care Center, Region Stockholm, Stockholm, Sweden (Ms Kolaas, Ms Hybelius, and Dr Axelsson),[c] Academic Primary Health Care Center, Region Stockholm, Stockholm, Sweden (Ms Kolaas, Ms Hybelius, and Dr Axelsson),[d] Division of Clinical Psychology, Department of Psychology, Uppsala University, Uppsala, Sweden (Dr Berman),[e] Division of Psychology, Department of Clinical Neuroscience, Karolinska Institutet, Stockholm, Sweden (Dr Hedman-Lagerlöf),[f] Gustavsberg Primary Health Care Center, Region Stockholm, Gustavsberg, Sweden (Dr Hedman-Lagerlöf and Dr Lindsäter), and[g] Division of Family Medicine and Primary Care, Department of Neurobiology, Care Sciences and Society, Karolinska Institutet, Stockholm, Sweden (Ms Hybelius and Dr Axelsson).

Corresponding Author: Karoline Kolaas, MSc, Centre for Psychiatry Research, Department of Clinical Neuroscience, Karolinska Institutet, Norra Stationsgatan 69, SE-113 64, Stockholm, Sweden,

Tel: +46 73 655 69 91

For peer review only

For peer review only - http://bmjopen.bmj.com/site/about/guidelines.xhtml

E-mail: karoline.kolaas@ki.se

Word count (abstract): 295; (main text): 4238; Tables: 1; Figures: 4

Page count (main text): 17; Line count (main text): 387

For peer review only

For peer review only - http://bmjopen.bmj.com/site/about/guidelines.xhtml

Abstract

Objective: Depression and anxiety are major public health problems. Online psychological treatments show promise. This study aimed to evaluate the effects of internet-delivered transdiagnostic psychological treatments for individuals with depression, anxiety, or both.

Design: Systematic review and meta-analysis.

Data sources: Medline (Ovid), Cochrane Library (Wiley), the Web of Science Core Collection (Clarivate), and PsycInfo (EBSCO).

Eligibility criteria: Randomized controlled trials of internet-delivered transdiagnostic psychological treatments for individuals with depression, anxiety, or both. Unique contributions of this review include that it concerned all treatment schools, both guided and unguided online formats, and all age groups.

Data extraction and synthesis: We estimated controlled effects on depression symptoms and anxiety in terms of Hedges' *g* with 95% CIs.

Results: We included 57 trials with 21795 participants. Nine of these trials (16%) recruited exclusively from routine care, and three (5%) delivered treatment via video. For adults in treatment, large within-group reductions were seen in symptoms of depression ($g = 0.90$; 95% CI 0.81-0.99) and anxiety ($g = 0.87$; 95% CI 0.78-0.96). Compared to treatment-as-usual and waitlist controls, the added effects were moderate (depression: $g = 0.52$; 95% CI 0.42-0.63; anxiety: $g = 0.45$; 95% CI 0.34-0.56), and larger in trials that required all participants to meet full diagnostic criteria for depression or an anxiety disorder, compared to

For peer review only - http://bmjopen.bmj.com/site/about/guidelines.xhtml

trials without this requirement. Compared to attention/engagement controls, the added effects were small (depression: g = 0.30; 95% CI 0.07-0.53; anxiety: g = 0.21; 95% CI 0.01-0.42). Heterogeneity was substantial. Two trials concerned adolescents and reported mixed results. One trial concerned older adults and reported promising results.

Conclusion: Internet-delivered transdiagnostic treatments for both depression and anxiety show added effects that are small-moderate varying by control condition. Research is needed regarding routine care, the video format, and age groups other than working-age adults.

PROSPERO registration number: CRD42021243172.

For peer review only

<Strengths and limitations of this study>

- The systematic search strategy and the broad eligibility criteria allowed for a comprehensive overview of treatment schools, formats, age groups, and settings including primary health care.
- Several trial, participant, and treatment characteristics were evaluated as potential moderators of the between-group effect versus treatment-as-usual and waitlist controls.
- The majority of trials recruited self-referred participants through advertisement and were not conducted in clinical settings. This makes it more difficult to draw conclusions about effectiveness in routine care.
- Our analyses of moderators were based exclusively on trial-level characteristics. This implies poor power, and often restriction of range.

For peer review only - http://bmjopen.bmj.com/site/about/guidelines.xhtml

<Introduction>

Mental disorders are among the top worldwide causes of disease burden.[1] Psychological treatments for depression and anxiety can be effective[2] including in clinical settings such as primary care[3 4] where the majority of patients are found.[5] However, most patients with depression and anxiety disorders remain untreated,[6 7] illustrating the need for further development and dissemination of effective therapies. It is widely recognized that depression and anxiety disorders often occur together,[8 9] and overlap in terms of genetic, cognitive, and behavioral characteristics.[10-12] This speaks for the use of broader, transdiagnostic, psychological treatments developed to be effective for both depression and anxiety. In addition to beneficial effects for the patient, transdiagnostic treatments can also have organizational advantages, such as streamlined procedures and less time needed for diagnostic assessment.[13] Another method of improving access to psychological treatments can be to deliver these online – for example via websites, mobile apps, or videoconferencing – which saves time and costs for both clinicians and patients. In general, transdiagnostic psychological treatments have been found to have moderate pooled effects on depression and anxiety compared to mostly treatment-as-usual and waitlist control groups.[14] Looking more specifically at internet-delivered transdiagnostic psychological treatments, large within-group symptom reductions have been observed, with mixed outcomes compared to a heterogeneous range of control groups, and comparisons versus diagnosis-specific treatments typically resulting in relatively similar effects on depression and general anxiety.[15-17] However, several fundamental questions about this increasingly common treatment form remain unanswered. First, it remains unclear whether effects generalize to children, adolescents, and older adults. Second, there has been no systematic comparison of treatment schools beyond the cognitive-behavioral therapies. Third, the relative efficacy of methods of delivery such as via websites, mobile apps, and videoconferencing is yet unclear. Fourth, it is not known how well the

For peer review only - http://bmjopen.bmj.com/site/about/guidelines.xhtml

effects of internet-delivered formats translate to routine clinical settings such as primary care. We conducted a systematic review and meta-analysis of randomized controlled trials of internet-delivered transdiagnostic psychological treatments for depression and anxiety. This systematic review included all treatment schools, both guided and unguided formats, and all age groups. Based on previous research, we hypothesized that controlled effects versus treatment-as-usual and waitlist controls would be large, and that the treatment length would be of little importance for the overall effect.

<Method>

<Search strategy>

This systematic review and meta-analysis adhered to the PRISMA 2020 statement[18] and was preregistered with PROSPERO (CRD42021243172) and the Open Science Framework (https://osf.io/dtcey). We searched Medline (Ovid), Cochrane Library (Wiley), the Web of Science Core Collection (Clarivate), and PsycInfo (EBSCO) for randomized controlled trials of internet-delivered transdiagnostic psychological treatments for individuals with depression, anxiety, or both. A search strategy was developed in collaboration with the Karolinska Institutet library service. Four types of search terms were combined: terms for treatment (e.g., "psychotherapy"), terms for online communication (e.g., "internet-delivered"), terms for diagnostic focus (e.g., "transdiagnostic", "depression", "anxiety"), and terms for study design (e.g., "RCT"). Only publications published in 1995 or later were considered. Full search documentation for all databases can be found in the online supplement. An initial search was conducted on 24 May 2021, with an update on 6 February 2023 in accordance with Bramer and Bain.[19]

For peer review only - http://bmjopen.bmj.com/site/about/guidelines.xhtml

<*Selection of studies*>

We employed a systematic de-duplication algorithm in Endnote X9,[20] after which unique search hits were imported into Rayyan.[21] The publications were then assessed by two independent assessors in two phases. In phase 1, all titles and abstracts were screened for eligibility by two authors (of KK, AHB, EHL, and EA), and non-relevant publications were excluded. In phase 2, publications that had been included by at least one assessor in phase 1 were reviewed in full text. One assessor (KK) did this sequentially, i.e., from criterion a to h (see below), for all full texts. Each full text was also reviewed by a second assessor (either AHB, EHL, JH, or EA) who suggested a decision of exclusion based on the first exclusion criterion identified, i.e., in any order. Cases of disagreement were discussed in the research group and a final decision was reached either in consensus or, if necessary, by means of voting. If information about the eligibility criteria was missing, authors were not contacted to provide additional information.

<*Eligibility criteria*>

(a) Studies had to be published in English-language journals and peer-reviewed.

(b) Studies had to be randomized controlled trials where the study participant was the unit of randomization. Only one publication was formally included for each randomized trial.

(c) Studies were required to evaluate the efficacy of an internet-delivered transdiagnostic psychological treatment for anxiety and depression. "Internet-delivered" included treatments delivered via websites, mobile apps, or videoconferencing. Treatments where online components were a mere complement to a face-to-face treatment, or where participants were required to attend a particular physical location, were excluded. "Transdiagnostic" was defined as intending to target depression as well as

For peer review only - http://bmjopen.bmj.com/site/about/guidelines.xhtml

anxiety. "Psychological treatment" was defined as an intervention anchored in psychological theory with a clinical framing (i) where a clinician or facilitator had an active role in moderating the process, or (ii) which was delivered in a structured manner, for example in the form of modules with suggested homework, or with an interactive component such as automatic feedback. For example, a webpage with general information about depression and anxiety would not be considered a psychological treatment, due to the lack of structure and interactivity.

(d) Studies needed to recruit a mix of participants suffering from clinically significant depression and anxiety. We considered depression and anxiety to be clinically significant if (i) all participants were required to meet full diagnostic criteria for either primary depression or a primary anxiety disorder and both populations were included in the trial, or (ii) all participants were required to score above a valid cut-off on an accepted screening measure for either depression or anxiety and both populations were included in the trial, or (iii) all participants were required to score above both a valid cut-off for depression and a valid cut-off for anxiety, or (iv) the baseline sample mean of at least one transdiagnostic treatment arm and at least one other arm were above both a valid cut-off for depression and a valid cut-off for anxiety. Studies that subsumed obsessive-compulsive disorder or post-traumatic stress disorder under the umbrella of anxiety disorders, in accordance with earlier DSM versions, were included. We excluded studies that required all participants to meet full diagnostic criteria for both depression and an anxiety disorder at the same time. Studies that included a substantial proportion of subclinical participants were included only if clinical participants were reported separately.

(e) Studies were required not to have selected participants based on medical diagnoses or characteristics that made it unreasonable to generalize to typical patients with

For peer review only - http://bmjopen.bmj.com/site/about/guidelines.xhtml

depression and anxiety disorders. Studies focusing on specific subgroups such as cancer patients or post-partum depression were thus excluded.

(f) Studies were required to have incorporated at least one arm where at least 10 participants were randomized to an internet-delivered transdiagnostic psychological treatment.

(g) Studies were required to have employed at least one valid self-report questionnaire for depression or anxiety. Domain-specific anxiety scales such as those pertaining to social anxiety, health anxiety, or panic disorder anxiety were not considered adequate.

(h) Studies were required to have incorporated a post-treatment assessment no later than three months from treatment termination.

(i) Studies were required to lend themselves to the tabulation of means and standard deviations, either based on the original publication or information from the author (see below).

<Data extraction>

Pre-specified variables were extracted by the first author (KK), including participant demographics (e.g., age and gender). The following study and sample characteristics were extracted: study setting (primary care, not primary care, or unclear), recruitment path (routine care or not routine care), main inclusion criterion (psychiatric diagnosis or "cut-off or symptoms"), mean depression severity (mild, moderate, or severe), and mean anxiety severity (mild, moderate, or severe). Symptom severity ratings were based on published norm data.[22-25] Treatment characteristics extracted were: delivery format (website, mobile app, video, mixed or other), therapist support (guided or unguided), treatment type (CBT, mindfulness, mixed CBT and mindfulness, or other), specific treatment protocol, treatment length (≥6 weeks or <6 weeks), component flexibility (standardized or tailored), and therapist time

For peer review only - http://bmjopen.bmj.com/site/about/guidelines.xhtml

(minutes per participant in treatment). Each control group was coded as either (a) a treatment-as-usual or waitlist condition (TAU/WL), (b) an attention/engagement control, or (c) as a bona fide treatment. There were two main reasons why we decided to collapse the treatment-as-usual and waitlists into one single category: First, few waitlists require patients to abstain from other treatments, and the setting as well as the proportion of participants in other treatments such as pharmacotherapy was often poorly documented. Considering that treatment-as-usual conditions can also be very rudimentary[26] we found no reliable way of distinguishing between these and the waitlists. Second, controls referred to as “treatment-as-usual” by the original authors that we found to be designed to control for the attention of a clinician or the effects of being enrolled in an intervention were instead subsumed under the category of attention/engagement controls. For each trial, the primary outcome for depression and anxiety was tabulated. If no outcome was defined as primary, or the primary outcome measure was deemed not to be valid, we tabulated the first valid measure that was mentioned. KK contacted the corresponding author when main outcome data was missing. All authors were also asked to provide pre-post correlations for the calculation of within-group effects. Intention-to-treat estimates were tabulated whenever available.

<Risk of bias assessment>

Trial risk of bias was assessed using a modified version of the Cochrane risk-of-bias tool for randomized trials version 2 (RoB 2).[27] The RoB 2 covers five domains: (i) the randomization process, (ii) deviations from intended interventions, (iii) missing outcome data, (iv) measurement of the outcome, and (v) selection of the reported results. Because of the difficulty blinding therapists to the psychological treatment they are delivering, we omitted items related to blinding. Consistent with previous similar research, non-blinding did not therefore lead to an elevated risk of bias.[14 28] Each trial received one rating on each risk of

For peer review only - http://bmjopen.bmj.com/site/about/guidelines.xhtml

bias domain, and this rating concerned both the depression and the anxiety outcome. Reporting one score was sufficient because all trials administered the depression and anxiety measures in parallel, which resulted in identical ratings. Authors KK and EL independently assessed the trials and discussed cases of disagreement to reach consensus. The decision was made that if five articles in a row were rated identically by both assessors, it would no longer be necessary with two assessments per article. This target was achieved after 25 articles, and KK assessed the remaining trials.

<Statistical analysis>

We report studies focusing on children and adolescents, and studies focusing on older adults, separately due to their specific focus. Effects versus other bona fide treatments were too few to be included in meta-analysis. At the end of the tabulation phase, we also decided to report studies with a treatment length of two weeks or shorter separately, a duration which would not typically be regarded as bona fide in routine clinical care. Nine randomized factorial trials were also included in the meta-analysis of within-group effects, but their main results were summarized separately.

We proceeded with meta-analysis using random-effects models in R 4.2.0[29] with the metafor 3.8-1 package.[30] Our overall strategy was to first conduct (i) a meta-analysis of within-group change in treatment, then (ii) a meta-analysis of between-group effects versus treatment-as-usual and waitlist controls, and last (iii) a meta-analysis of between-group effects versus attention/engagement controls. Effects were quantified in terms of Hedges' g, i.e., the bias-corrected standardized mean difference.[31] Absolute values for g of 0.2 are usually regarded as small, 0.5 as moderate, and 0.8 as large.[32] Estimates of heterogeneity were based on the I^2 statistic, which stands for the percentage of the between-sample variance that is due to true study differences as opposed to sampling error. An I^2 of 25% is usually regarded as low, 50%

For peer review only - http://bmjopen.bmj.com/site/about/guidelines.xhtml

as moderate, and 75% as high,[33] though the I^2 also increases with the sample sizes of the original trials. [34] Therefore, we also report the τ^2 which is an absolute measure of heterogeneity. Pertaining to the meta-analysis of trials of transdiagnostic treatment vs. treatment-as-usual and waitlist controls, a series of planned moderator analyses were conducted on the basis of trial, sample, and treatment characteristics whenever strata included at least four studies. One exception was made to this rule where we analyzed primary care setting as a potential moderator based on four arms from three trials because we found this test important. In the analysis of trials with treatment-as-usual and waitlist controls, we also investigated publication bias based on the visual inspection of funnel plots in combination with Egger's test[35] and the Duval and Tweedie trim and fill procedure.[36] In line with recommendations,[37] the latter was repeated using the R0, L0, and Q0 estimators.

For peer review only

<Results>

The inclusion process is presented in in Fig. 1. Out of 435 RCTs reviewed in full text, 55 were suggested for inclusion by both assessors, 335 were immediate exclusions, and 45 were disagreements (90% agreement; κ = 0.65). The reasons for these disagreements are listed in the online supplement. Of the disagreements, five trials were included after discussion. Three trials were then excluded due to insufficient data. This resulted in a final sample of 57 RCTs. Full references to these trials are found in supplement B.

INSERT FIGURE 1 ABOUT HERE

For peer review only - http://bmjopen.bmj.com/site/about/guidelines.xhtml

<Study characteristics>

In the 57 included trials, 21795 participants were randomized. Arms were: 83 internet-delivered transdiagnostic psychological treatments, 36 TAU/WLs, 8 attention/engagement controls, and three other bona fide treatments. With regard to age groups, 53/57 trials (93%) recruited working-age adults, 3/57 (5%) children or adolescents, and 1/57 (2%) older adults. As to delivery formats, 37/57 (65%) of the internet-delivered transdiagnostic psychological treatments were delivered via a website mainly through text, 8/57 (14%) via a mobile app, 3/57 (5%) via video, and (9/57) 16% were mixed or other. In terms of the therapeutic model, 37/57 (65%) evaluated some variation on cognitive behavioral therapy (CBT), 8/57 (14%) mindfulness-based therapy, 3/57 (5%) mixed CBT and mindfulness, and 11/57 (19%) other schools such as problem-solving therapy or affect-focused psychodynamic therapy. As for the recruitment strategy, 39/57 (68%) trials relied on self-referrals, 9/57 (16%) recruited from routine care including student counselling, and 9/57 (16%) used a mixed strategy or other methods such as recruitment via a public health care website. Illustrating the lack of work in the routine clinical setting, 54/57 trials (95%) did not report having been conducted in primary health care, and 3/57 (5%) reported having been conducted in primary health care. The trials were based in Australia 15/57 (26%), Canada 12/57 (21%), United States 6/57 (11%,), United Kingdom 5/57 (9%), Sweden 4/57 (7%), the Netherlands 3/57 (5%), and other countries 12/57 (21%). More trial characteristics are tabulated in the online supplement.

<Study risk of bias>

The distribution of study risk-of-bias ratings is presented in Figure 2. Overall, 9/57 (16%) of the trials were rated as having a low risk of bias, 14/57 (25%) as raising some concerns, and 34/57 (60%) as having a high risk of bias. The most common reason for an overall high risk was missing outcome data, which was seen in 29/59 (51%) of the trials, followed by

For peer review only - http://bmjopen.bmj.com/site/about/guidelines.xhtml

deviations from the intended interventions in 15/57 (26%). Pooled effect sizes did not differ between trials as a function of the overall risk of bias (see Table 1).

INSERT FIGURE 2 ABOUT HERE

<Effects on depression and anxiety>

For working-age adults, the pooled within-group reduction in depression symptoms was large (g = 0.90; 95% CI 0.81-0.99; k = 78) and the heterogeneity was high (I^2 = 95%; τ^2 = 0.14; Q_{77} =1606, p = < 0.001). The pooled within-group reduction in anxiety was also large (g = 0.87; 95% CI 0.78-0.96; k = 78) and the heterogeneity was high (I^2 = 95%; τ^2 = 0.16; Q_{77} = 1882, p = < 0.001). The pooled between-group effect versus the treatment-as-usual and waitlist controls was moderate for depression symptoms (g = 0.52; 95% CI 0.42-0.63; k = 43) with high heterogeneity (I^2 = 80%; τ^2 = 0.08; Q_{42} = 162, p = < 0.001), and moderate for anxiety (g = 0.45; 95% CI 0.34-0.56; k = 43) with high heterogeneity (I^2 = 83%; τ^2 = 0.10; Q_{42}= 176, p = < 0.001). See Fig. 3. The pooled between-group effect versus the attention/engagement controls was also significant but small for depression symptoms (g = 0.30; 95% CI 0.07-0.53; k = 10; I^2 = 91%; τ^2 = 0.12; Q_9 = 48, p = < 0.001) and small for anxiety (g = 0.21; 95% CI 0.01-0.42; k = 10; I^2 = 88%; τ^2 = 0.09; Q_9 = 45, p = < 0.001). See Fig. 4.

INSERT FIGURE 3 AND 4 ABOUT HERE

<Publication bias>

We explored indicators of publication bias in the meta-analysis of effects versus treatment-as-usual and waitlist controls. For depression, there was clear indication of small- to medium-sized trials with non-beneficial effects of internet-delivered psychological treatments being

For peer review only - http://bmjopen.bmj.com/site/about/guidelines.xhtml

underreported, and with imputed studies the between-group effect dropped from 0.52 to 0.29 (95% CI 0.16-0.42). For anxiety, there were also indication of possible asymmetry in the same direction, but this was more ambiguous and the trim-and-fill procedure did not result in any imputed trials. See the online supplement for details.

<Moderators and subgroups>

The results of the planned moderator and subgroup analyses are presented in Table 1. With regard to between-group effects versus the treatment-as-usual and waitlist controls, one moderator was found to be significant for both depression and anxiety symptoms: trials that required all participants to meet full criteria for a psychiatric diagnosis reported a larger pooled between-group effect as compared to trials that did not have this requirement. We also identified 9 randomized factorial trials. These manipulated various aspects of the treatment format and reported mostly null results with small effect sizes. In single trials, small moderating effects were reported for therapist guidance, app recommendations, and pre-treatment motivational interviewing.[38 39] In other trials, null results were however also reported for guidance and motivational interviewing.[40 41]

For peer review only

Table 1. Moderator and subgroup analyses of the relative effect of Internet-delivered transdiagnostic psychological treatment versus treatment-as-usual and waitlist controls.

**DEPRESSION SYMPTOMS**								
**Potential moderator**	***Q (df)***	***p***	**Subgroup**	***k***	***g***	***95% CI***	***I²***	**τ²**
Pooled total				43	0.52	0.42-0.63	80 %	0.08
Study and sample characteristics								
Study setting	2.14 (1)	0.144	Primary health care	4	0.30†	-0.03-0.62	79 %	0.08
			Not or unclear	39	0.55	0.44-0.66		

For peer review only - http://bmjopen.bmj.com/site/about/guidelines.xhtml

Recruitment path	3.32 (1)	0.068	Routine care	8	0.32	0.07-0.57	81%	0.09
			Not routine care	31	0.58	0.45-0.71		
Main inclusion criterion	6.33 (1)	0.012*	Psychiatric diagnosis	12	0.74	0.54-0.94	78%	0.08
			Cut-off or symptoms	31	0.44	0.32-0.56		
Depression severity	2.31 (2)	0.315	Mild	5	0.46	0.18-0.74	68%	0.06
			Moderate	32	0.53	0.42-0.64		
			Severe	4	0.28†	-0.04-0.59		
Anxiety severity	1.29 (1)	0.255	Mild or moderate	30	0.46	0.35-0.56	74%	0.06
			Severe	10	0.59	0.38-0.79		
Treatment characteristics								
Delivery format	1.51 (2)	0.471	Website	29	0.50	0.38-0.63	79%	0.08
			Mobile app	6	0.43	0.09-0.77		
			Mixed or other	8	0.66	0.41-0.90		
Therapist support	0.91 (1)	0.341	Yes, guided	23	0.58	0.43-0.73	80%	0.08
			No, unguided	20	0.47	0.32-0.62		
Treatment type	4.67 (2)	0.097	CBT	29	0.53	0.41-0.66	77%	0.27
			Mindfulness	4	0.88	0.49-1.27		
			Other	10	0.40	0.21-0.60		
Specific protocols	0.06 (1)	0.812	Wellbeing course	6	0.49	0.21-0.78	81%	0.09
			Not wellbeing course	37	0.53	0.41-0.65		
Treatment length	0.02 (1)	0.901	≥6 weeks	28	0.53	0.40-0.66	79%	0.09
			<6 weeks	15	0.52	0.33-0.70		
Component flexibility	1.00 (1)	0.318	Standardized	36	0.56	0.44-0.68	81%	0.09
			Tailored	5	0.39	0.08-0.70		
Therapist time††	0.88 (1)	0.349	Per additional 10 min	28	0.00†	-0.01-0.01	63%	0.03
Overall study risk of bias								
Cochrane tool version 2	0.84 (2)	0.658	High	29	0.49	0.36-0.63	81%	0.09
			Some concerns	8	0.55	0.30-0.80		
			Low	6	0.63	0.35-0.91		
**ANXIETY**								
**Potential moderator**	***Q (df)***	***p***	**Subgroup**	***k***	***g***	***95% CI***	***I²***	**τ²**
Pooled total				43	0.45	0.34-0.56	83%	0.10
Study and sample characteristics								
Study setting	0.91 (1)	0.340	Primary health care	4	0.28†	-0.07-0.64	83%	0.10
			Not or unclear	39	0.47	0.35-0.59		

For peer review only

For peer review only - http://bmjopen.bmj.com/site/about/guidelines.xhtml

Recruitment path	2.19 (1)	0.139	Routine care	8	0.30	0.04-0.55	82 %	0.10
			Not routine care	31	0.52	0.38-0.65		
Main inclusion criterion	7.01 (1)	0.008*	Psychiatric diagnosis	12	0.68	0.48-0.89	81 %	0.09
			Cut-off or symptoms	31	0.36	0.23-0.48		
Depression severity	2.60 (2)	0.272	Mild	5	0.51	0.20-0.81	74 %	0.08
			Moderate	32	0.44	0.32-0.56		
			Severe	4	0.16†	-0.18-0.50		
Anxiety severity	0.85 (1)	0.358	Mild or moderate	30	0.38	0.26-0.50	79 %	0.07
			Severe	10	0.50	0.28-0.72		
Treatment characteristics								
Delivery format	0.02 (2)	0.988	Website	29	0.45	0.31-0.59	83 %	0.11
			Mobile app	6	0.42	0.06-0.79		
			Mixed or other	8	0.46	0.19-0.73		
Therapist support	1.67 (1)	0.196	Yes, guided	23	0.52	0.36-0.68	82 %	0.10
			No, unguided	20	0.38	0.22-0.53		
Treatment type	5.57 (2)	0.062	CBT	29	0.44	0.31-0.57	80 %	0.29
			Mindfulness	4	0.89	0.48-1.30		
			Other	10	0.34	0.13-0.55		
Specific protocols	0.96 (1)	0.328	Wellbeing course	6	0.31	0.00-0.61	84 %	0.10
			Not wellbeing course	37	0.47	0.35-0.60		
Treatment length	0.00 (1)	0.973	≥6 weeks	28	0.45	0.31-0.59	83 %	0.11
			<6 weeks	15	0.45	0.25-0.66		
Component flexibility	2.22 (1)	0.136	Standardized	36	0.49	0.36-0.61	84 %	0.11
			Tailored	5	0.22†	-0.11-0.55		
Therapist time††	0.47 (1)	0.494	Per additional 10 min	28	0.00†	-0.01-0.02	85 %	0.09
Overall study risk of bias								
Cochrane tool version 2	0.01 (2)	0.995	High	29	0.45	0.31-0.60	84 %	0.11
			Some concerns	8	0.44	0.17-0.71		
			Low	6	0.46	0.16-0.76		

In accordance with the study protocol, strata were collapsed or excluded from analysis if $k <$ 4. For anxiety severity, “mild” ($k = 3$) and “moderate” were collapsed. For delivery formats, “video” ($k = 3$) was subsumed under “mixed or other”. For treatment types, “CBT and mindfulness” ($k = 3$) was subsumed under “other”. Q-tests listed in this table are tests of

For peer review only - http://bmjopen.bmj.com/site/about/guidelines.xhtml

moderation, with the intercept included in the statistical model. Heterogeneity is reported as I^2 and τ^2. CBT, cognitive behavior therapy.

* Test of moderation significant at $\alpha = 0.05$.

† Pooled effect not significantly different from zero.

†† Therapist time per participant was tested as a continuous variable.

<Effects versus other bona fide treatments>

Three trials compared the effects of an internet-delivered transdiagnostic psychological treatment to those of a distinctly different bona fide treatment. They showed mixed results and are described in the online supplement.[42 43 44]

<Children, adolescents, and older adults>

Two trials recruited children or adolescents. One trial recruited ages 12-21 (M=16, SD=2; N=45) and reported null effects of 5 weeks of problem-solving therapy on depression (g = 0.04) and anxiety (g = -0.11) as compared to a waitlist.[45] The second trial recruited ages 13-18 (M=16, SD=1; N=103) in a school setting and found that a single session promoting growth, gratitude, and values had significant and moderate added effects on depression (g = 0.50) but not anxiety (g = 0.32) as compared to a control that focused on the promotion of study skills.[46] One trial exclusively recruited older adults, aged 60+ (M=66, SD=5; N=433), and found that 8 weeks of CBT in a structured text-based self-help format was associated with large within-group reductions in depression (gs = 1.19-1.30) and anxiety (gs = 1.21-1.31), but that there was no significant added effect of adding guidance or an initial interview.[47]

For peer review only

<Discussion>

In this systematic review and meta-analysis based on 57 randomized controlled trials we found that, for working-age adults, internet-delivered transdiagnostic psychological treatments led to moderate between-group effects compared to treatment-as-usual and waitlist controls (depression: $g = 0.52$; anxiety: $g = 0.45$) and small between-group effects compared to attention/engagement controls (depression: $g = 0.30$; anxiety: $g = 0.21$). Heterogeneity was substantial. There were also indications of publication bias, which probably inflated the pooled effect on depression versus treatment-as-usual and waitlist controls – perhaps as much as 0.23 units. One significant moderator was found: trials where all participants were required to meet full diagnostic criteria for depression or an anxiety disorder reported larger between-group effects versus treatment-as-usual and waitlist controls, than did trials without this requirement. Pooled within-group symptom reductions were large (depression: $g = 0.90$; anxiety: $g = 0.87$). Strengths of the present study include the systematic search strategy and the broad eligibility criteria, which allowed for an overview of treatment schools, formats, settings including primary health care, and also age groups.

<Comparison with prior research>

Our final sample of 57 RCTs pertaining specifically to internet-delivered transdiagnostic psychological treatments suitable for both primary depression and primary anxiety disorders is large compared to previous systematic reviews.[14] This is probably explained both by the rapid growth of this research field, and our use of exhaustive search terms developed with the specific aim of identifying internet-delivered transdiagnostic psychological treatments. The between-group effect versus treatment-as-usual and waitlist controls was slightly smaller than expected, though still broadly in line with previous meta-analyses.[14 15] Our expectation, based on previous research,[16] that treatment length would be of little importance for the controlled

For peer review only - http://bmjopen.bmj.com/site/about/guidelines.xhtml

effect was also supported in our data. Similarly, the finding that effects on depression are likely to be inflated by publication bias is also in line with previous research and therefore not unexpected.[48]

<Children, adolescents, and older adults>

This exhaustive literature search showed very clearly that the internet-delivered transdiagnostic treatments for children and adolescents, as well as specifically for older adults, have not come very far. Especially for children and adolescents, we found this rather surprising considering that there is a relatively long tradition of transdiagnostic treatments specifically for the anxiety disorders.[49] Conceivably, this may have reduced the incentive to develop newer protocols that can accommodate patients with depression as well. For older adults, we identified a single study which presented promising results. It is widely acknowledged that older adults rarely receive psychological treatment.[50] We wish to encourage further work in this area, especially with increased technical prowess in this population.

<Possible benefit of meeting a diagnosis>

In the exploratory moderator analyses, the only variable that was a significant moderator of the controlled effect of treatment was whether participants were required to meet full criteria for a psychiatric diagnosis. One interpretation is that of a "specificity effect", where larger effects can be expected if patients suffer from clinical depression or an anxiety disorder, as opposed to symptoms caused for example by a somatoform disorder or a somatic disease.

For peer review only - http://bmjopen.bmj.com/site/about/guidelines.xhtml

<*Treatment characteristics*>

Except for the possible benefits of a diagnosis, a general pattern in both the planned tests of potential moderators and the original factorial trials was the lack of significant moderators, not least in terms of treatment characteristics. One interesting finding was for example that therapist-guidance was not found to be a significant moderator in our meta-analyses, and that results from the factorial trials were inconsistent. We found that it is still unusual for trials to investigate the effect of treatments other than CBT or mindfulness-based interventions. There were, however, also examples of other treatments investigated in more than one trial, namely problem-solving therapies[45 51 52] and bias-modification training therapies.[53 54]

<*New formats: Are we at a crossroads?*>

For the past quarter of a century, internet-delivered psychological treatments have mostly been delivered via web platforms, with standardized linear text presentations intended to be accessed via a web browser. This systematic review illustrates that this treatment format is still most widely researched, and that newer formats remain understudied. We were able to pool the mean effects of 6 arms from 5 trials focusing on mobile apps, and 5 arms from 5 trials of tailored treatments, which showed no significant differences compared to the more common standardized web format. Notably, only three trials investigated videoconferencing interventions[55-57], and only one trial investigated the effect of an intervention using artificial intelligence[58]. The vast majority of the literature still pertains to the standardized web-based text format, but the direction of the field appears to be towards automated, tailored, interventions.

For peer review only - http://bmjopen.bmj.com/site/about/guidelines.xhtml

<Limitations>

A clear limitation was that the majority of trials recruited self-referred participants through advertisement and were not conducted in clinical setting. The fact that these samples might have been highly motivated, and not representative of patients in routine care, makes it more difficult to draw conclusions about effectiveness in routine care. Another key limitation is that our analyses of moderators were based exclusively on trial-level characteristics. This implies poor power, and often restriction of range, in many tests such as that of whether pooled effects differ as a function of mean baseline symptom level (potential moderator).

<Clinical implications and recommendations for future research>

This meta-analysis contributes evidence that transdiagnostic psychological treatments are effective for people with elevated depression symptoms or anxiety. However, even though a substantial proportion of patients are treated in routine care settings such as primary care,[5 59] only three (5%) of the 57 included trials were conducted in a primary care setting. Power was limited to quantify the pooled effect of this stratum, and *g* did not reach statistical significance. This means that there is an urgent need to further evaluate internet-delivered transdiagnostic treatments in the routine clinical context. It is also important when planning for future RCTs to reduce currently high levels of missing data. More studies evaluating video-delivered interventions are needed. Adequately powered RCTs comparing internet-delivered transdiagnostic treatments with other bona fide treatments for depression and anxiety are also needed.

<Conclusion>

Internet-delivered transdiagnostic treatments suitable for both depression and anxiety appear to have moderate effects compared to treatment-as-usual and waitlist controls, and significant

For peer review only - http://bmjopen.bmj.com/site/about/guidelines.xhtml

but small effects beyond those of attention/engagement controls. Evidence is lacking with regard to routine care, the video format, and age groups other than working-age adults.

For peer review only

For peer review only - http://bmjopen.bmj.com/site/about/guidelines.xhtml

Acknowledgements: We thank the Karolinska Institutet library service, and in particular Sabina Gillsund and Emma-Lotta Säätelä, for contributing to the systematic database search. We would also like to thank all study authors who responded to our queries and contributed with data on request.

Competing interests: KK, AHB, EHL, JH and EA have taken part in original research on internet-delivered treatment including transdiagnostic treatment.

Contributions of authors: KK and EA led the study design and data collection process, with input from all authors. KK and EA conducted the statistical analysis. KK wrote the initial draft in close collaboration with EA. All authors reviewed the manuscript for intellectual content, made contributions, and approved the final version. KK and EA had full access to all data and take responsibility for the integrity of the data and the accuracy of the presented analysis.

Funding: This work was supported by the Swedish Research Council (EA; identifier: 2021-06496) and the Krica Foundation. Neither of the funders had any role in the design, execution, or publication process.

Availability of data: Tabulated data is available from the authors on request.

Karoline Kolaas, MSc, Karolinska Institutet, Dept of Clin. Neuroscience, Stockholm, Sweden, **Anne H Berman,** PhD, Uppsala University, Dept of Psychology, Uppsala, Sweden, **Erik Hedman-Lagerlöf**, PhD, Karolinska Institutet, Dept of Clin. Neuroscience, Stockholm,

For peer review only

Sweden, **Elin Lindsäter**, PhD, Karolinska Institutet, Dept of Clin. Neuroscience, Stockholm, Sweden, **Jonna Hybelius**, MSc, Karolinska Institutet, Dept of Neurobiology, Care Sciences and Society, Stockholm, Sweden, & **Erland Axelsson**, PhD, Karolinska Institutet, Dept of Neurobiology, Care Sciences and Society, Stockholm, Sweden

For peer review only

References

1. GBD 2019 Mental Disorders Collaborators. Global, regional, and national comparative risk assessment of 84 behavioural, environmental and occupational, and metabolic risks or clusters of risks for 195 countries and territories, 1990-2017: a systematic analysis for the Global Burden of Disease Study 2017. *Lancet* 2018;392(10159):1923-94. doi: 10.1016/s0140-6736(18)32225-6 [published Online First: 20181108]
2. Cuijpers P, Cristea IA, Karyotaki E, et al. How effective are cognitive behavior therapies for major depression and anxiety disorders? A meta-analytic update of the evidence. *World Psychiatry* 2016;15(3):245-58. doi: 10.1002/wps.20346 [published Online First: 2016/10/08]
3. Santoft F, Axelsson E, Öst LG, et al. Cognitive behaviour therapy for depression in primary care: systematic review and meta-analysis. *Psychological medicine* 2019:1-9. doi: 10.1017/S0033291718004208 [published Online First: 2019/01/29]
4. Twomey C, O'Reilly G, Byrne M. Effectiveness of cognitive behavioural therapy for anxiety and depression in primary care: a meta-analysis. *Family practice* 2015;32(1):3-15. doi: 10.1093/fampra/cmu060 [published Online First: 20140922]
5. Alonso J, Angermeyer MC, Bernert S, et al. Use of mental health services in Europe: results from the European Study of the Epidemiology of Mental Disorders (ESEMeD) project. *Acta psychiatrica Scandinavica Supplementum* 2004(420):47-54. doi: 10.1111/j.1600-0047.2004.00330.x [published Online First: 2004/05/07]
6. Thornicroft G, Chatterji S, Evans-Lacko S, et al. Undertreatment of people with major depressive disorder in 21 countries. *Br J Psychiatry* 2017;210(2):119-24. doi: 10.1192/bjp.bp.116.188078 [published Online First: 20161201]

For peer review only - http://bmjopen.bmj.com/site/about/guidelines.xhtml

7. Alonso J, Liu Z, Evans-Lacko S, et al. Treatment gap for anxiety disorders is global: Results of the World Mental Health Surveys in 21 countries. *Depression and anxiety* 2018;35(3):195-208. doi: 10.1002/da.22711 [published Online First: 20180122]

8. McGrath JJ, Lim CCW, Plana-Ripoll O, et al. Comorbidity within mental disorders: a comprehensive analysis based on 145 990 survey respondents from 27 countries. *Epidemiol Psychiatr Sci* 2020;29:e153. doi: 10.1017/s2045796020000633 [published Online First: 20200812]

9. Jacobson NC, Newman MG. Anxiety and depression as bidirectional risk factors for one another: A meta-analysis of longitudinal studies. *Psychol Bull* 2017;143(11):1155-200. doi: 10.1037/bul0000111 [published Online First: 20170814]

10. Sloan E, Hall K, Moulding R, et al. Emotion regulation as a transdiagnostic treatment construct across anxiety, depression, substance, eating and borderline personality disorders: A systematic review. *Clinical psychology review* 2017;57:141-63. doi: 10.1016/j.cpr.2017.09.002 [published Online First: 20170911]

11. Wilamowska ZA, Thompson-Hollands J, Fairholme CP, et al. Conceptual background, development, and preliminary data from the unified protocol for transdiagnostic treatment of emotional disorders. *Depression and anxiety* 2010;27(10):882-90. doi: 10.1002/da.20735

12. Ringwald WR, Forbes MK, Wright AGC. Meta-analysis of structural evidence for the Hierarchical Taxonomy of Psychopathology (HiTOP) model. *Psychological medicine* 2023;53(2):533-46. doi: 10.1017/S0033291721001902 [published Online First: 2021/05/14]

13. Kolaas K, Berman AH, Hedman-Lagerlöf E, et al. Feasibility of a video-delivered mental health course for primary care patients: a single-group prospective cohort study. *BMC*

For peer review only - http://bmjopen.bmj.com/site/about/guidelines.xhtml

Prim Care 2023;24(1):28. doi: 10.1186/s12875-023-01989-8 [published Online First: 20230123]

14. Cuijpers P, Miguel C, Ciharova M, et al. Transdiagnostic treatment of depression and anxiety: a meta-analysis. *Psychological medicine* 2023:1-12.
15. Newby JM, Twomey C, Yuan Li SS, et al. Transdiagnostic computerised cognitive behavioural therapy for depression and anxiety: A systematic review and meta-analysis. *Journal of affective disorders* 2016;199:30-41. doi: https://doi.org/10.1016/j.jad.2016.03.018
16. Păsărelu CR, Andersson G, Bergman Nordgren L, et al. Internet-delivered transdiagnostic and tailored cognitive behavioral therapy for anxiety and depression: a systematic review and meta-analysis of randomized controlled trials. *Cogn Behav Ther* 2017;46(1):1-28. doi: 10.1080/16506073.2016.1231219 [published Online First: 20161007]
17. Thompson EM, Destree L, Albertella L, et al. Internet-based acceptance and commitment therapy: a transdiagnostic systematic review and meta-analysis for mental health outcomes. *Behavior therapy* 2021;52(2):492-507.
18. Page MJ, McKenzie JE, Bossuyt PM, et al. The PRISMA 2020 statement: an updated guideline for reporting systematic reviews. *BMJ (Clinical research ed)* 2021;372:n71. doi: 10.1136/bmj.n71 [published Online First: 20210329]
19. Bramer W, Bain P. Updating search strategies for systematic reviews using EndNote. *J Med Libr Assoc* 2017;105(3):285-89. doi: 10.5195/jmla.2017.183 [published Online First: 20170701]
20. Bramer WM, Giustini D, de Jonge GB, et al. De-duplication of database search results for systematic reviews in EndNote. *J Med Libr Assoc* 2016;104(3):240-3. doi: 10.3163/1536-5050.104.3.014

For peer review only

For peer review only - http://bmjopen.bmj.com/site/about/guidelines.xhtml

21. Ouzzani M, Hammady H, Fedorowicz Z, et al. Rayyan-a web and mobile app for systematic reviews. *Systematic reviews* 2016;5(1):210. doi: 10.1186/s13643-016-0384-4 [published Online First: 20161205]

22. Crawford J, Cayley C, Lovibond PF, et al. Percentile norms and accompanying interval estimates from an Australian general adult population sample for self - report mood scales (BAI, BDI, CRSD, CES - D, DASS, DASS - 21, STAI - X, STAI - Y, SRDS, and SRAS). *Australian Psychologist* 2011;46(1):3-14.

23. Crawford JR, Garthwaite PH, Lawrie CJ, et al. A convenient method of obtaining percentile norms and accompanying interval estimates for self - report mood scales (DASS, DASS - 21, HADS, PANAS, and sAD). *British Journal of Clinical Psychology* 2009;48(2):163-80.

24. Schalet BD, Cook KF, Choi SW, et al. Establishing a common metric for self-reported anxiety: linking the MASQ, PANAS, and GAD-7 to PROMIS Anxiety. *Journal of anxiety disorders* 2014;28(1):88-96.

25. Wahl I, Löwe B, Bjorner JB, et al. Standardization of depression measurement: a common metric was developed for 11 self-report depression measures. *Journal of clinical epidemiology* 2014;67(1):73-86.

26. Watts SE, Turnell A, Kladnitski N, et al. Treatment-as-usual (TAU) is anything but usual: a meta-analysis of CBT versus TAU for anxiety and depression. *Journal of affective disorders* 2015;175:152-67.

27. Sterne JAC, Savović J, Page MJ, et al. RoB 2: a revised tool for assessing risk of bias in randomised trials. *BMJ (Clinical research ed)* 2019;366:l4898. doi: 10.1136/bmj.l4898 [published Online First: 20190828]

For peer review only - http://bmjopen.bmj.com/site/about/guidelines.xhtml

28. Bisby MA, Karin E, Hathway T, et al. A meta-analytic review of randomized clinical trials of online treatments for anxiety: Inclusion/exclusion criteria, uptake, adherence, dropout, and clinical outcomes. *Journal of anxiety disorders* 2022;92:102638. doi: 10.1016/j.janxdis.2022.102638 [published Online First: 20221007]

29. R Core Team. R: A Language and Environment for Statistical Computing. R Foundation for Statistical Computing, Vienna, Austria. 2016

30. Viechtbauer W. Conducting meta-analyses in R with the metafor package. *Journal of statistical software* 2010;36(3):1-48.

31. Hedges LV. Distribution theory for Glass's estimator of effect size and related estimators. *journal of Educational Statistics* 1981;6(2):107-28.

32. Cohen J. Statistical power analysis for the behavioral science. 2 ed: Hillsdale, NJ: Lawrence Erlbaum Associates 1988:24-27.

33. Higgins JP, Thompson SG, Deeks JJ, et al. Measuring inconsistency in meta-analyses. *BMJ* 2003;327(7414):557-60. doi: 10.1136/bmj.327.7414.557 [published Online First: 2003/09/06]

34. Rücker G, Schwarzer G, Carpenter JR, et al. Undue reliance on I 2 in assessing heterogeneity may mislead. *BMC medical research methodology* 2008;8:1-9.

35. Egger M, Davey Smith G, Schneider M, et al. Bias in meta-analysis detected by a simple, graphical test. *BMJ (Clinical research ed)* 1997;315(7109):629-34. doi: 10.1136/bmj.315.7109.629

36. Duval S, Tweedie R. Trim and fill: A simple funnel-plot-based method of testing and adjusting for publication bias in meta-analysis. *Biometrics* 2000;56(2):455-63. doi: 10.1111/j.0006-341x.2000.00455.x

37. Shi L, Lin L. The trim-and-fill method for publication bias: practical guidelines and recommendations based on a large database of meta-analyses. *Medicine* 2019;98(23)

For peer review only - http://bmjopen.bmj.com/site/about/guidelines.xhtml

38. Mohr DC, Schueller SM, Tomasino KN, et al. Comparison of the Effects of Coaching and Receipt of App Recommendations on Depression, Anxiety, and Engagement in the IntelliCare Platform: Factorial Randomized Controlled Trial. *Journal of medical Internet research* 2019;21(8):e13609. doi: 10.2196/13609 [published Online First: 20190828]

39. Peynenburg V, Hadjistavropoulos H, Thiessen D, et al. Internet-Delivered Cognitive Behavioral Therapy for Postsecondary Students: Randomized Factorial Trial for Examining Motivational Interviewing and Booster Lessons. *Journal of medical Internet research* 2022;24(9):e40637. doi: 10.2196/40637 [published Online First: 20220907]

40. Dear BF, Fogliati VJ, Fogliati R, et al. Treating anxiety and depression in young adults: A randomised controlled trial comparing clinician-guided versus self-guided Internet-delivered cognitive behavioural therapy. *Aust N Z J Psychiatry* 2018;52(7):668-79. doi: 10.1177/0004867417738055 [published Online First: 20171024]

41. Soucy JN, Hadjistavropoulos HD, Karin E, et al. Brief online motivational interviewing pre-treatment intervention for enhancing internet-delivered cognitive behaviour therapy: A randomized controlled trial. *Internet Interv* 2021;25:100394. doi: 10.1016/j.invent.2021.100394 [published Online First: 20210420]

42. Borjalilu S, Mazaheri MA, Talebpour A. Effectiveness of mindfulness-based stress management in the mental health of Iranian university students: a comparison of blended therapy, face-to-face sessions, and mHealth app (Aramgar). *Iranian Journal of Psychiatry and Behavioral Sciences* 2019;13(2)

For peer review only - http://bmjopen.bmj.com/site/about/guidelines.xhtml

43. Sethi S. Treating Youth Depression and Anxiety: A Randomised Controlled Trial Examining the Efficacy of Computerised versus Face ‐ to ‐ face Cognitive Behaviour Therapy. *Australian Psychologist* 2013;48(4):249-57. doi: 10.1111/ap.12006

44. Villemaire-Krajden R, Myhr G. Evaluating the use of a computerized CBT program for outpatients on a waitlist in a university CBT unit. *Journal of Psychiatric Practice®* 2019;25(4):268-78.

45. Hoek W, Schuurmans J, Koot HM, et al. Effects of Internet-based guided self-help problem-solving therapy for adolescents with depression and anxiety: a randomized controlled trial. *PLoS One* 2012;7(8):e43485. doi: 10.1371/journal.pone.0043485 [published Online First: 20120831]

46. Osborn TL, Rodriguez M, Wasil AR, et al. Single-session digital intervention for adolescent depression, anxiety, and well-being: Outcomes of a randomized controlled trial with Kenyan adolescents. *Journal of consulting and clinical psychology* 2020;88(7):657-68. doi: 10.1037/ccp0000505 [published Online First: 20200511]

47. Titov N, Fogliati VJ, Staples LG, et al. Treating anxiety and depression in older adults: randomised controlled trial comparing guided v. self-guided internet-delivered cognitive–behavioural therapy. *BJPsych open* 2016;2(1):50-58.

48. Cuijpers P, Miguel C, Harrer M, et al. Cognitive behavior therapy vs. control conditions, other psychotherapies, pharmacotherapies and combined treatment for depression: a comprehensive meta ‐ analysis including 409 trials with 52,702 patients. *World Psychiatry* 2023;22(1):105-15.

49. Ewing DL, Monsen JJ, Thompson EJ, et al. A meta-analysis of transdiagnostic cognitive behavioural therapy in the treatment of child and young person anxiety disorders. *Behavioural and cognitive psychotherapy* 2015;43(5):562-77.

For peer review only

50. Wei W, Sambamoorthi U, Olfson M, et al. Use of psychotherapy for depression in older adults. *American Journal of Psychiatry* 2005;162(4):711-17.

51. Kleiboer A, Donker T, Seekles W, et al. A randomized controlled trial on the role of support in Internet-based problem solving therapy for depression and anxiety. *Behaviour research and therapy* 2015;72:63-71.

52. van Straten A, Cuijpers P, Smits N. Effectiveness of a web-based self-help intervention for symptoms of depression, anxiety, and stress: randomized controlled trial. *Journal of medical Internet research* 2008;10(1):e7.

53. Rogers J, Sicouri G. A Single-Session Online Cognitive Bias Modification of Interpretations Modified for Adults With Anxiety and Depressive Symptoms. *Behavior therapy* 2022;53(5):967-80. doi: 10.1016/j.beth.2022.04.006 [published Online First: 20220419]

54. Hirsch CR, Krahé C, Whyte J, et al. Effects of modifying interpretation bias on transdiagnostic repetitive negative thinking. *Journal of consulting and clinical psychology* 2020;88(3):226.

55. Grégoire S, Beaulieu F, Lachance L, et al. An online peer support program to improve mental health among university students: A randomized controlled trial. *Journal of American College Health* 2022:1-13.

56. Hosseinzadeh Asl NR. A randomized controlled trial of a mindfulness-based intervention in social workers working during the COVID-19 crisis. *Current Psychology* 2022;41(11):8192-99.

57. Tönnies J, Hartmann M, Wensing M, et al. Mental health specialist video consultations versus treatment-as-usual for patients with depression or anxiety disorders in primary care: randomized controlled feasibility trial. *JMIR mental health* 2021;8(3):e22569.

For peer review only - http://bmjopen.bmj.com/site/about/guidelines.xhtml

58. Fitzpatrick KK, Darcy A, Vierhile M. Delivering cognitive behavior therapy to young adults with symptoms of depression and anxiety using a fully automated conversational agent (Woebot): a randomized controlled trial. *JMIR mental health* 2017;4(2):e7785.

59. Wittchen H-U, Mühlig S, Beesdo K. Mental disorders in primary care. *Dialogues in Clinical Neuroscience* 2003;5(2):115-28. doi: 10.31887/DCNS.2003.5.2/huwittchen

For peer review only

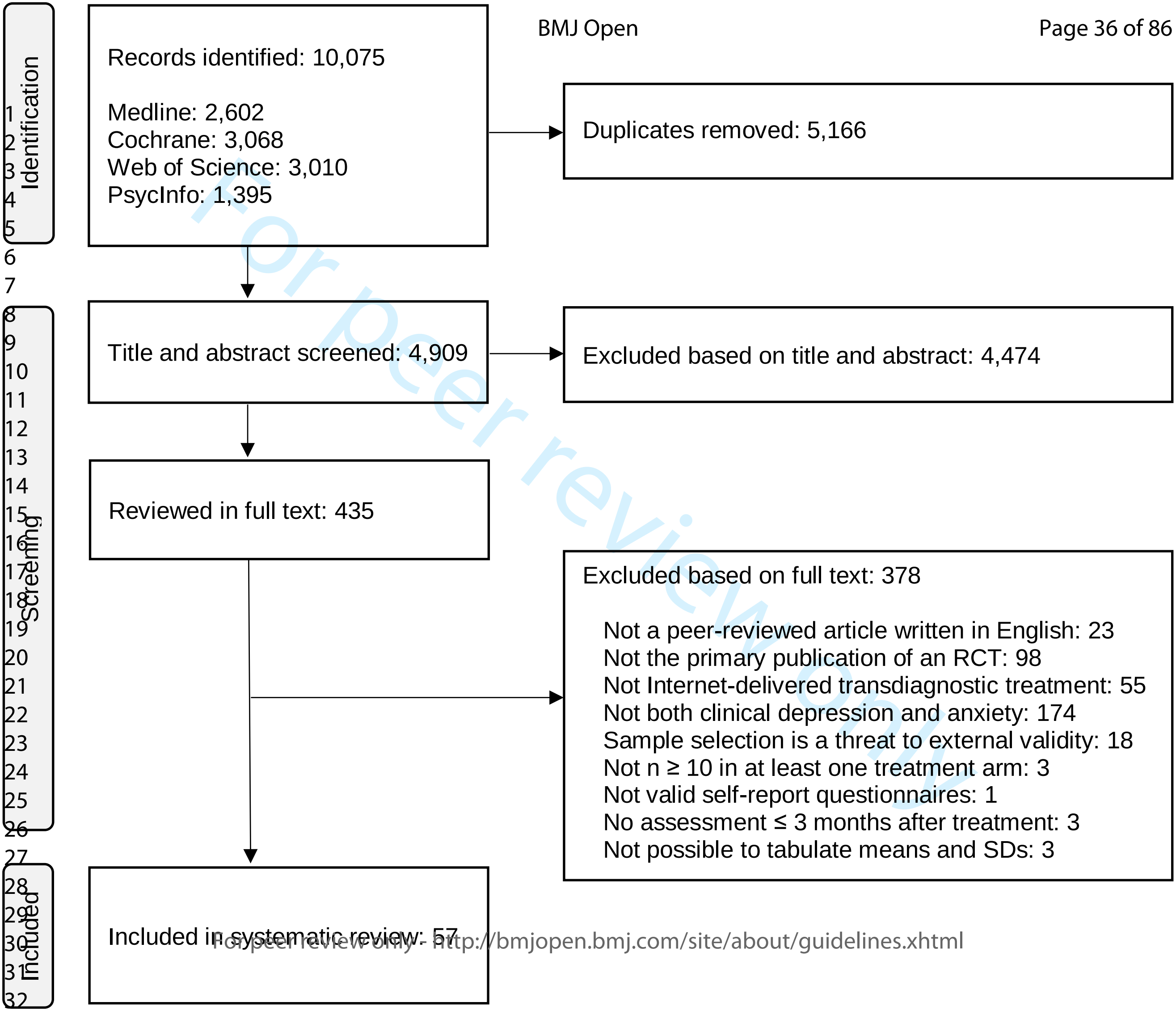

For peer review only - http://bmjopen.bmj.com/site/about/guidelines.xhtml

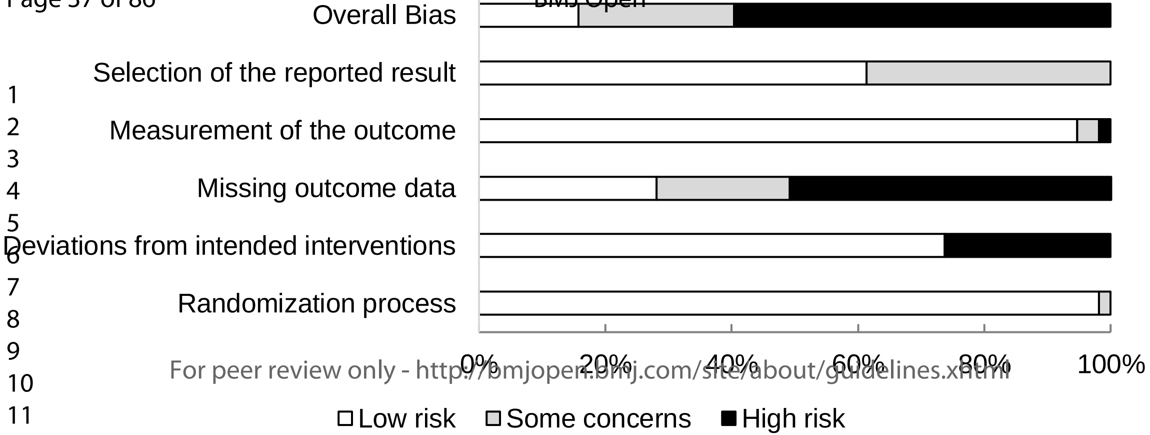

For peer review only - http://bmjopen.bmj.com/site/about/guidelines.xhtml

Standardized effect on depression symptoms

Study	Format	Type	*g* [95% CI]
Al−Refaee (2021)	Mobile app	CBT and mindfulness	0.30 [−0.03, 0.62]
Carolan (2017) I	Web, mainly text	CBT variant	0.19 [−0.35, 0.72]
Carolan (2017) II	Web, mainly text	CBT variant	0.27 [−0.25, 0.80]
Diaz−Garcia (2021) I	Mixed or other	CBT variant	1.21 [0.80, 1.61]
Diaz−Garcia (2021) II	Mixed or other	CBT variant	1.04 [0.63, 1.46]
Farrer (2019)	Mixed or other	CBT and mindfulness	0.15 [−0.12, 0.43]
Gonzalez−Robles (2020)	Web, mainly text	CBT variant	0.37 [0.09, 0.65]
Graham (2020)	Mobile app	CBT variant	0.71 [0.37, 1.04]
Grégoire (2022)	Video format	CBT variant	0.53 [0.14, 0.91]
Hensel (2019)	Web, mainly text	CBT variant	0.41 [0.22, 0.60]
Hosseinzadeh (2021)	Video format	Mindfulness	0.30 [−0.26, 0.87]
Kang (2022)	Mixed or other	Mindfulness	1.56 [1.11, 2.01]
Kladnitski (2020) I	Web, mainly text	CBT variant	0.89 [0.43, 1.36]
Kladnitski (2020) II	Web, mainly text	CBT and mindfulness	1.16 [0.68, 1.64]
Kladnitski (2020) III	Web, mainly text	Mindfulness	0.82 [0.36, 1.28]
Kleiboer (2015) I	Web, mainly text	Problem−solving therapy	0.26 [−0.01, 0.53]
Kleiboer (2015) II	Web, mainly text	Problem−solving therapy	0.29 [0.02, 0.56]
Kleiboer (2015) III	Web, mainly text	Problem−solving therapy	0.40 [0.13, 0.68]
Krafft (2019) I	Mobile app	CBT variant	0.57 [−0.44, 1.58]
Krafft (2019) II	Mobile app	CBT variant	0.51 [−0.49, 1.51]
Levin (2020)	Mobile app	Mindfulness	0.41 [−0.62, 1.43]
Lindegaard (2020)	Web, mainly text	CBT variant	0.29 [−0.22, 0.81]
McCloud (2020)	Mobile app	CBT variant	0.20 [−0.20, 0.60]
Mullin (2015)	Web, mainly text	CBT variant	0.39 [−0.16, 0.94]
Newby (2013)	Web, mainly text	CBT variant	0.99 [0.59, 1.39]
Powell (2013)	Web, mainly text	CBT variant	0.21 [0.14, 0.28]
Proudfoot (2013)	Web, mainly text	Hybrid or other	0.51 [0.32, 0.69]
Robichaud (2020)	Web, mainly text	CBT variant	0.35 [−0.14, 0.84]
Rollman (2018) I	Web, mainly text	CBT variant	0.15 [−0.08, 0.37]
Rollman (2018) II	Web, mainly text	CBT variant	0.15 [−0.07, 0.38]
Schaeuffele (2022)	Web, mainly text	CBT variant	0.82 [0.47, 1.18]
Sethi (2013)	Web, mainly text	CBT variant	1.93 [1.23, 2.63]
Southwell (2017)	Web, mainly text	Gratitude diary	0.37 [−0.01, 0.75]
Terides (2018)	Web, mainly text	CBT variant	0.38 [0.04, 0.71]
Titov (2011)	Web, mainly text	CBT variant	0.57 [0.10, 1.03]
Titov (2013) I	Web, mainly text	CBT variant	0.67 [0.39, 0.96]
Titov (2013) II	Web, mainly text	CBT variant	0.54 [0.27, 0.81]
Twomey (2014)	Web, mainly text	CBT variant	0.19 [−0.30, 0.68]
Tönnies (2021)	Video format	Hybrid or other	0.19 [−0.39, 0.78]
Tullbure (2018)	Web, mainly text	CBT variant	0.96 [0.53, 1.38]
van Staten (2008)	Web, mainly text	Problem−solving therapy	0.50 [0.22, 0.77]
Viskovich (2019)	Mixed or other	CBT variant	0.36 [0.25, 0.48]
Zetterqvist (2003)	Web, mainly text	CBT variant	0.61 [0.08, 1.13]
Pooled total			0.52 [0.42, 0.63]

−2 −1 0 1 2

Higher values favour internet−delivered psychological treatment vs. TAU/WLC

Standardized effect on anxiety

Study	Format	Type	*g* [95% CI]
Al−Refaee (2021)	Mobile app	CBT and mindfulness	0.19 [−0.13, 0.52]
Carolan (2017) I	Web, mainly text	CBT variant	0.30 [−0.24, 0.84]
Carolan (2017) II	Web, mainly text	CBT variant	0.44 [−0.09, 0.97]
Diaz−Garcia (2021) I	Mixed or other	CBT variant	0.64 [0.24, 1.04]
Diaz−Garcia (2021) II	Mixed or other	CBT variant	0.68 [0.28, 1.08]
Farrer (2019)	Mixed or other	CBT and mindfulness	0.13 [−0.14, 0.41]
Gonzalez−Robles (2020)	Web, mainly text	CBT variant	0.35 [0.07, 0.63]
Graham (2020)	Mobile app	CBT variant	0.60 [0.27, 0.93]
Grégoire (2022)	Video format	CBT variant	0.51 [0.13, 0.90]
Hensel (2019)	Web, mainly text	CBT variant	0.42 [0.23, 0.61]
Hosseinzadeh (2021)	Video format	Mindfulness	0.28 [−0.29, 0.85]
Kang (2022)	Mixed or other	Mindfulness	1.41 [0.97, 1.84]
Kladnitski (2020) I	Web, mainly text	CBT variant	1.06 [0.59, 1.54]
Kladnitski (2020) II	Web, mainly text	CBT and mindfulness	1.22 [0.74, 1.70]
Kladnitski (2020) III	Web, mainly text	Mindfulness	0.99 [0.52, 1.46]
Kleiboer (2015) I	Web, mainly text	Problem−solving therapy	0.30 [0.03, 0.57]
Kleiboer (2015) II	Web, mainly text	Problem−solving therapy	0.27 [0.00, 0.54]
Kleiboer (2015) III	Web, mainly text	Problem−solving therapy	0.33 [0.06, 0.60]
Krafft (2019) I	Mobile app	CBT variant	0.96 [−0.08, 2.00]
Krafft (2019) II	Mobile app	CBT variant	0.49 [−0.51, 1.49]
Levin (2020)	Mobile app	Mindfulness	0.56 [−0.47, 1.59]
Lindegaard (2020)	Web, mainly text	CBT variant	−0.16 [−0.67, 0.35]
McCloud (2020)	Mobile app	CBT variant	0.20 [−0.20, 0.60]
Mullin (2015)	Web, mainly text	CBT variant	0.59 [0.04, 1.15]
Newby (2013)	Web, mainly text	CBT variant	0.96 [0.56, 1.36]
Powell (2013)	Web, mainly text	CBT variant	0.19 [0.12, 0.27]
Proudfoot (2013)	Web, mainly text	Hybrid or other	0.27 [0.09, 0.45]
Robichaud (2020)	Web, mainly text	CBT variant	0.35 [−0.14, 0.84]
Rollman (2018) I	Web, mainly text	CBT variant	0.19 [−0.04, 0.41]
Rollman (2018) II	Web, mainly text	CBT variant	0.20 [−0.03, 0.42]
Schaeuffele (2022)	Web, mainly text	CBT variant	0.75 [0.39, 1.10]
Sethi (2013)	Web, mainly text	CBT variant	1.96 [1.26, 2.66]
Southwell (2017)	Web, mainly text	Gratitude diary	0.39 [0.01, 0.77]
Terides (2018)	Web, mainly text	CBT variant	0.40 [0.06, 0.73]
Titov (2011)	Web, mainly text	CBT variant	−0.51 [−0.97, −0.05]
Titov (2013) I	Web, mainly text	CBT variant	0.56 [0.28, 0.85]
Titov (2013) II	Web, mainly text	CBT variant	0.39 [0.11, 0.66]
Twomey (2014)	Web, mainly text	CBT variant	0.01 [−0.48, 0.50]
Tönnies (2021)	Video format	Hybrid or other	0.13 [−0.46, 0.71]
Tullbure (2018)	Web, mainly text	CBT variant	0.93 [0.51, 1.35]
van Staten (2008)	Web, mainly text	Problem−solving therapy	0.33 [0.06, 0.60]
Viskovich (2019)	Mixed or other	CBT variant	0.02 [−0.10, 0.13]
Zetterqvist (2003)	Web, mainly text	CBT variant	0.56 [0.11, 1.00]
Pooled total			0.45 [0.34, 0.56]

−2 −1 0 1 2

Higher values favour internet−delivered psychological treatment vs. TAU/WLC

For peer review only

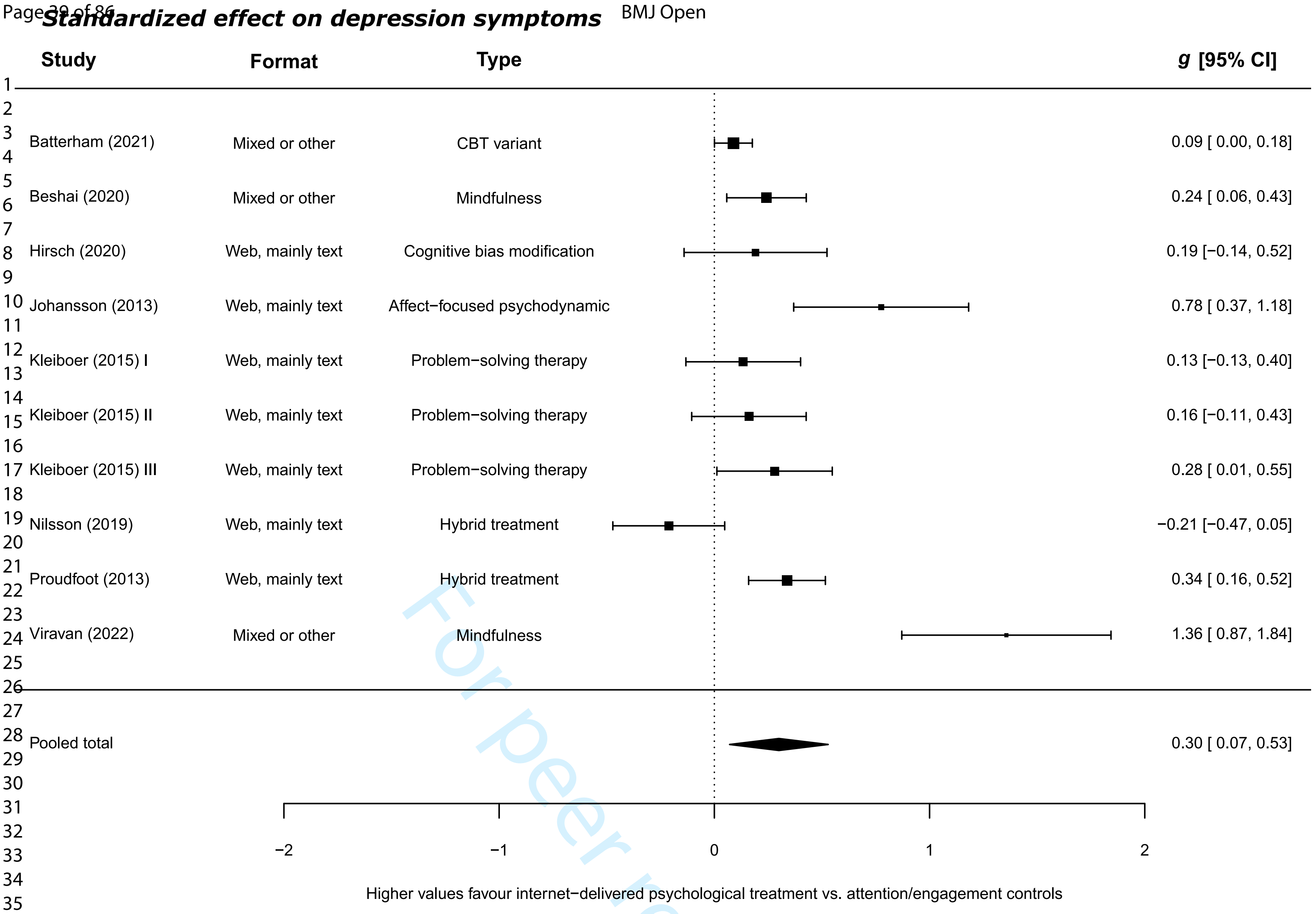

For peer review only

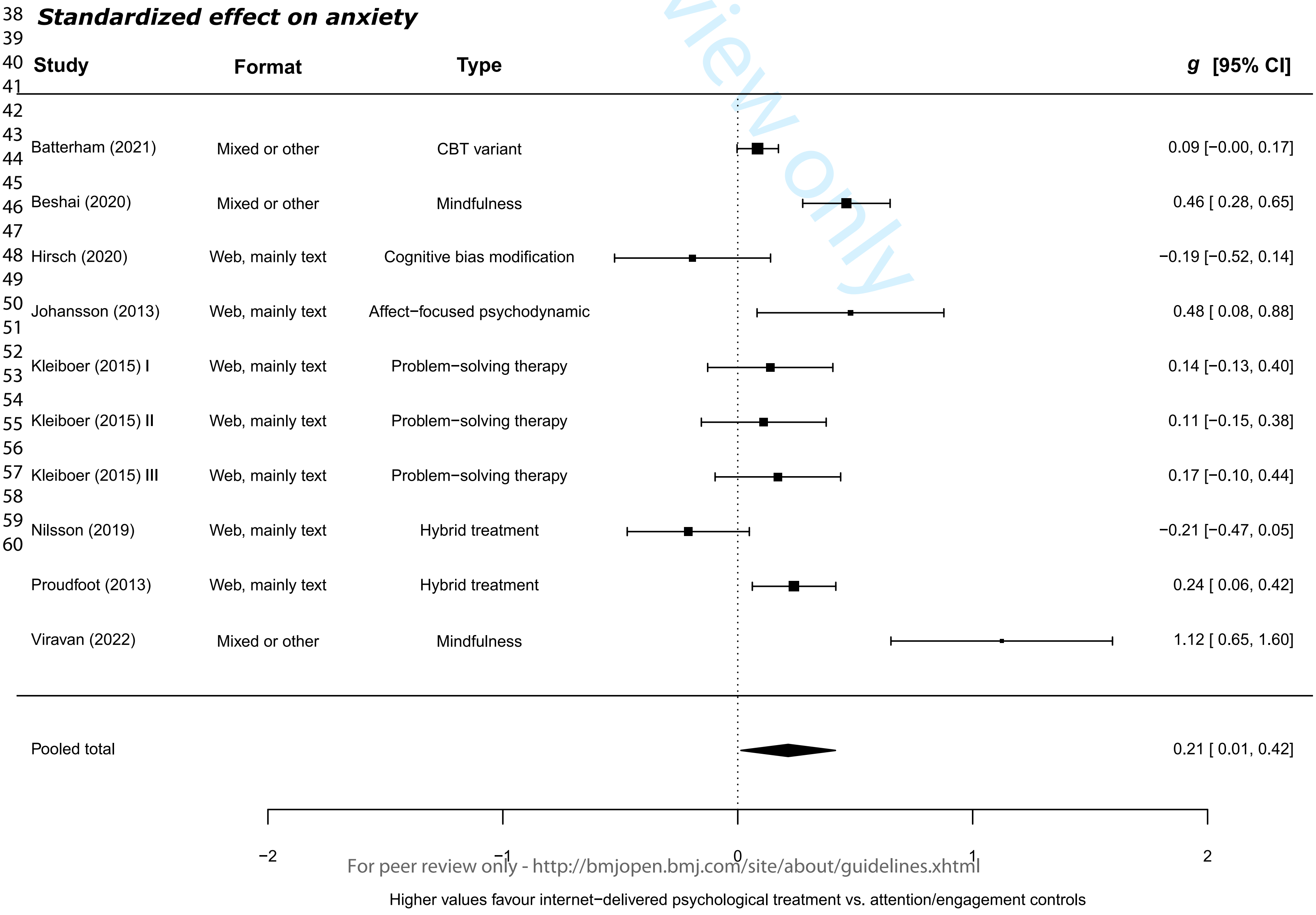

Internet-delivered transdiagnostic psychological treatments for individuals with depression, anxiety, or both

Systematic search: 2021 main search

For peer review only

Karolinska Institutet

Documentation of search strategies
University Library search consultation group

Date: May 24, 2021

Topic/research question: Systematic review and meta-analysis of transdiagnostic psychological interventions delivered via the internet for individuals suffering from common mental disorders (PROSPERO: CRD42021243172)

Name of researcher(s): Erland Axelsson

Librarian(s): Sabina Gillsund & Emma-Lotta Säätelä

Databases:

1. Medline (Ovid)
2. Cochrane Library (Wiley)
3. Web of Science Core Collection (Clarivate Analytics)
4. PsycInfo (Ovid)

Total number of hits:

- Before deduplication: 7,366
- After deduplication: 3,519

Comments:

For peer review only - http://bmjopen.bmj.com/site/about/guidelines.xhtml

Karolinska Institutet

1. Medline

Interface: Ovid MEDLINE(R) and Epub Ahead of Print, In-Process & Other Non-Indexed Citations and Daily<br><br>Date of Search: 24 May 2021<br><br>Number of hits: 1,898<br><br>Comment: In Ovid, two or more words are automatically searched as phrases; i.e. no quotation marks are needed	Field labels<br><br>• exp/ = exploded MeSH term<br>• / = non exploded MeSH term<br>• .ti,ab,kf. = title, abstract and author keywords<br>• adjx = within x words, regardless of order<br>• * = truncation of word for alternate endings

#	Searches	Results
1	exp Psychotherapy/	202021
2	(("acceptance and commitment" or affect-focused or behavio?r* or biofeedback or computeri* or cognit* or digital or "emotional awareness and expression" or educat* or exposure or guided or internet* or interpersonal or mobile or online or positive psychology or psychodynamic or psychologic* or self-guided or self-help or transdiagnost* or web*) adj2 (intervention* or therap* or treat* or program*)).ti,ab,kf.	188596
3	(behavio?ral activation or bias modification or cognitive restructuring or hypno* or meditation or mindfulness or problem solving or psychoeducation* or psychotherap* or relaxation or self-management or unified protocol).ti,ab,kf.	251506
4	or/1-3	538226
5	Anxiety Disorders/	35452
6	Agoraphobia/	2617
7	Neurotic Disorders/	17987
8	Obsessive-Compulsive Disorder/	14943
9	Panic Disorder/	7019
10	exp Phobic Disorders/	11590
11	Stress Disorders, Post-Traumatic/	34732
12	Anxiety/	87458
13	(agoraphobi* or anxi* or obsessive-compulsive or panic or phobi* or post-traumatic stress or posttraumatic stress).ti,ab,kf.	281431
14	or/5-13	336088

For peer review only - http://bmjopen.bmj.com/site/about/guidelines.xhtml

15	Mood Disorders/	14821
16	Depressive Disorder/	73869
17	Depressive Disorder, Major/	32106
18	Affective Symptoms/	13300
19	Depression/	127756
20	depress*.ti,ab,kf.	491024
21	((affective or mood) adj2 disorder*).ti,ab,kf.	37052
22	or/15-21	562251
23	14 and 22	140055
24	(emotional adj2 disorder*).ti,ab,kf.	3677
25	(transdiagnost* or unified protocol).ti,ab,kf.	2780
26	24 or 25	6275
27	23 or 26	144265
28	Internet/	75778
29	Internet-Based Intervention/	544
30	Therapy, Computer-Assisted/	6898
31	Telemedicine/	28197
32	Telerehabilitation/	548
33	exp Remote Consultation/	5247
34	Mobile Applications/	7799
35	Videoconferencing/	1919
36	(app or apps or cellphone* or computer* or digital or e mail or email or internet* or mobile application* or online or phone* or smartphone* or technolog* or telephone* or text message* or video* or virtual or web based or web site or website).ti,ab,kf.	1396114
37	(distance counsel?ing or distance consultation* or e consultation* or econsultation* or e counsel?ing or ecounsel?ing or e health* or ehealth* or e therapies or e therapy or etherap* or e visit* or evisit* or m health or mhealth or mobile counsel?ing or mobile consultation* or remote consultation* or remote counsel?ing or tele health* or telehealth* or tele consultation* or teleconsultation* or tele medicine or telemedicine or tele rehabilitation or telerehabilitation).ti,ab,kf.	36032
38	or/28-37	1441976
39	randomized controlled trial.pt.	531357
40	controlled clinical trial.pt.	94168
41	randomi?ed.ab.	622540

Karolinska Institutet

42	placebo.ab.	218143
43	clinical trials as topic.sh.	195916
44	randomly.ab.	357785
45	trial.ti.	240304
46	or/39-45	1407342
47	(systematic review or meta-analy*).ti.	219916
48	46 not 47	1340792
49	exp animals/ not humans.sh.	4832148
50	48 not 49	1230987
51	4 and 27 and 38 and 50	1921
52	limit 51 to english language	1905
53	limit 52 to yr="1995 -Current"	1898

For peer review only - http://bmjopen.bmj.com/site/about/guidelines.xhtml

Karolinska Institutet

2. Cochrane Library

Interface: Wiley<br><br>Date of Search: 24 May 2021<br><br>Number of hits: 2,337	Field labels<br><br>• ti,ab,kw = title, abstract and author keywords<br>• NEAR/x = within x words, regardless of order<br>• * = truncation of word for alternate endings

ID	Search	Hits
#1	MeSH descriptor: [Psychotherapy] explode all trees	24621
#2	(("acceptance and commitment" or "affect-focused" or behavio?r* or "biofeedback" or computeri* or cognit* or "digital" or "emotional awareness and expression" or educat* or "exposure" or "guided" or internet* or "interpersonal" or "mobile" or "online" or "positive psychology" or "psychodynamic" or psychologic* or "self-guided" or "self-help" or transdiagnost* or web*) NEAR/2 (intervention* or therap* or treat* or program*)):ti,ab,kw	81443
#3	((behavio?ral NEXT activation) or "bias modification" or "cognitive restructuring" or hypno* or "meditation" or "mindfulness" or "problem solving" or psychoeducation* or psychotherap* or "relaxation" or "self-management" or "unified protocol"):ti,ab,kw	54905
#4	#1 or #2 or #3	121709
#5	MeSH descriptor: [Anxiety Disorders] this term only	3927
#6	MeSH descriptor: [Agoraphobia] this term only	436
#7	MeSH descriptor: [Neurotic Disorders] this term only	300
#8	MeSH descriptor: [Obsessive-Compulsive Disorder] this term only	1050
#9	MeSH descriptor: [Panic Disorder] this term only	955
#10	MeSH descriptor: [Phobic Disorders] explode all trees	1382
#11	MeSH descriptor: [Stress Disorders, Post-Traumatic] this term only	2707
#12	MeSH descriptor: [Anxiety] this term only	7681
#13	(agoraphobi* or anxi* or "obsessive compulsive" or "obsessive-compulsive" or "panic" or phobi* or "post-traumatic stress" or "posttraumatic stress"):ti,ab,kw	63654
#14	#5 or #6 or #7 or #8 or #9 or #10 or #11 or #12 or #13	64273
#15	MeSH descriptor: [Mood Disorders] this term only	839
#16	MeSH descriptor: [Depressive Disorder] this term only	8011
#17	MeSH descriptor: [Depressive Disorder, Major] this term only	5111
#18	MeSH descriptor: [Affective Symptoms] this term only	459
#19	MeSH descriptor: [Depression] this term only	12730
#20	depress*:ti,ab,kw	89514
#21	(("affective" or "mood") NEAR/3 disorder*):ti,ab,kw	5404
#22	#15 or #16 or #17 or #18 or #19 or #20 or #21	91976
#23	#14 and #22	30987
#24	("emotional" NEAR/2 disorder*):ti,ab,kw	862
#25	(transdiagnost* or "unified protocol"):ti,ab,kw	730
#26	#24 or #25	1468
#27	#23 or #26	31780
#28	MeSH descriptor: [Internet] this term only	3893
#29	MeSH descriptor: [Internet-Based Intervention] this term only	182

For peer review only

For peer review only - http://bmjopen.bmj.com/site/about/guidelines.xhtml

Karolinska Institutet

#30	MeSH descriptor: [Therapy, Computer-Assisted] this term only	1342
#31	MeSH descriptor: [Telemedicine] this term only	2293
#32	MeSH descriptor: [Telerehabilitation] this term only	126
#33	MeSH descriptor: [Remote Consultation] explode all trees	398
#34	MeSH descriptor: [Mobile Applications] this term only	765
#35	MeSH descriptor: [Videoconferencing] this term only	190
#36	("app" or "apps" or cellphone* or computer* or "digital" or "e mail" or "email" or internet* or ("mobile" NEXT application*) or "online" or phone* or smartphone* or technolog* or telephone* or ("text" NEXT message*) or video* or "virtual" or "web based" or "web site" or "website"):ti,ab,kw	150142
#37	(("distance" NEXT "counsel?ing") or ("distance" NEXT consultation*) or ("e" NEXT consultation*) or econsultation* or ("e" NEXT "counsel?ing") or "ecounsel?ing" or ("e" NEXT health*) or ehealth* or "e therapies" or "e therapy" or etherap* or ("e" NEXT visit*) or evisit* or "m health" or "mhealth" or ("mobile" NEXT "counsel?ing") or ("mobile" NEXT consultation*) or ("remote" NEXT consultation*) or ("remote" NEXT "counsel?ing") or ("tele" NEXT health*) or telehealth* or ("tele" NEXT consultation*) or teleconsultation* or "tele medicine" or "telemedicine" or "tele rehabilitation" or "telerehabilitation"):ti,ab,kw	9310
#38	#28 or #29 or #30 or #31 or #32 or #33 or #34 or #35 or #36 or #37	152807
#39	#4 and #27 and #38	4365
#40	(clinicaltrials or trialsearch):so	364015
#41	#39 NOT #40	2416
	Limit to publication date 1995-2021 in Trials	2337

For peer review only - http://bmjopen.bmj.com/site/about/guidelines.xhtml

3. Web of Science Core Collection

Interface: Clarivate Analytics<br><br>Date of Search: 24 May 2021<br><br>Number of hits: 2,233	Field labels<br><br>• TS/Topic = title, abstract, author keywords and Keywords Plus<br>• NEAR/x = within x words, regardless of order<br>• * = truncation of word for alternate endings<br><br>Note: sometimes “quotation marks” are needed for single search terms to avoid automatic term mapping (lemmatization).

18 2,233

#14 AND #11 AND #8 AND #3

Refined by: LANGUAGES: (ENGLISH) AND [excluding] PUBLICATION YEARS: (1994 OR 1993) AND DOCUMENT TYPES: (ARTICLE OR CORRECTION OR EARLY ACCESS)

17 2,366

#14 AND #11 AND #8 AND #3

Refined by: LANGUAGES: (ENGLISH) AND [excluding] PUBLICATION YEARS: (1994 OR 1993)

16 2,370

#14 AND #11 AND #8 AND #3

Refined by: LANGUAGES: (ENGLISH)

15 2,395

#14 AND #11 AND #8 AND #3

14 916,173

#12 NOT #13

13 259,971

TI=(“systematic review” or “meta-analy*”)

12 982,134

TI=(“randomi$ed” OR “randomi$ed” OR “randomi$ation” OR “randomi$ation” OR placebo* OR (random* AND (allocat* OR assign*)) OR (blind* AND (“single” OR “double” OR “treble” OR “triple”))) OR AB=(“randomi$ed” OR “randomi$ed” OR “randomi$ation” OR “randomi$ation” OR placebo* OR (random* AND (allocat* OR assign*)) OR (blind* AND (“single” OR “double” OR “treble” OR “triple”)))

For peer review only - http://bmjopen.bmj.com/site/about/guidelines.xhtml

Karolinska Institutet

11 4,745,692

#10 OR #9

10 51,707

TS=(“distance counsel$ing” or “distance consultation*” or “e consultation*” or econsultation* or “e counsel$ing” or “ecounsel$ing” or “e health*” or ehealth* or “e therapies” or “e therapy” or etherap* or “e visit*” or evisit* or “m health” or “mhealth” or “mobile counsel$ing” or “mobile consultation*” or “remote consultation*” or “remote counsel$ing” or “tele health*” or telehealth* or “tele consultation*” or teleconsultation* or “tele medicine” or “telemedicine” or “tele rehabilitation” or “telerehabilitation")

9 4,726,717

TS=(“app” or “apps” or cellphone* or computer* or “digital” or “e mail” or “email” or internet* or “mobile application*” or “online” or phone* or smartphone* or technolog* or telephone* or “text message*” or video* or “virtual” or “web based” or “web site” or “website”)

8 176,232

#7 OR #6

7 7,752

TS=((”emotional” NEAR/1 disorder*) or transdiagnost* or “unified protocol”)

6 171,279

#5 AND #4

5 691,430

TS=(depress* or ((“affective” or “mood”) NEAR/1 disorder*))

4 401,339

TS=(agoraphobi* or anxi* or “obsessive-compulsive” or “panic” or phobi* or “post-traumatic stress” or “posttraumatic stress”)

3 871,462

#2 OR #1

2 644,089

For peer review only - http://bmjopen.bmj.com/site/about/guidelines.xhtml

TS=((“behavio$ral activation” or “bias modification” or “cognitive restructuring” or hypno* or “meditation” or “mindfulness” or “problem solving” or psychoeducation* or psychotherap* or “relaxation” or “self-management” or “unified protocol”))

1 263,093

TS=((("acceptance and commitment" or “affect-focused” or behavio$r* or “biofeedback” or computeri* or cognit* or “digital” or "emotional awareness and expression" or educat* or “exposure” or “guided” or internet* or “interpersonal” or “mobile” or “online” or “positive psychology” or “psychodynamic” or psychologic* or “self-guided” or “self-help” or transdiagnost* or web*) NEAR/1 (intervention* or therap* or treat* or program*)))

For peer review only

For peer review only - http://bmjopen.bmj.com/site/about/guidelines.xhtml

Karolinska Institutet

4. Psycinfo

Interface: Ovid<br><br>Date of Search: 24 May 2021<br><br>Number of hits: 898<br><br>Comment: In Ovid, two or more words are automatically searched as phrases; i.e. no quotation marks are needed	Field labels<br><br>• exp/ = exploded controlled term<br>• / = non exploded controlled term<br>• .ti,ab,id. = title, abstract and author keywords<br>• adjx = within x words, regardless of order<br>• * = truncation of word for alternate endings

#	Searches	Results
1	exp Psychotherapy/	207730
2	exp Cognitive Therapy/	13639
3	exp Behavior Therapy/	21289
4	exp Cognitive Behavior Therapy/	23144
5	(("acceptance and commitment" or affect-focused or behavio?r* or biofeedback or computeri* or cognit* or digital or "emotional awareness and expression" or educat* or exposure or guided or internet* or interpersonal or mobile or online or positive psychology or psychodynamic or psychologic* or self-guided or self-help or transdiagnost* or web*) adj2 (intervention* or therap* or treat* or program*)).ti,ab,id.	175860
6	(behavio?ral activation or bias modification or cognitive restructuring or hypno* or meditation or mindfulness or problem solving or psychoeducation* or psychotherap* or relaxation or self-management or unified protocol).ti,ab,id.	240370
7	or/1-6	495594
8	Anxiety Disorders/	19140
9	exp Agoraphobia/	2916
10	Neurosis/	7043
11	Obsessive Compulsive Disorder/	14683
12	Panic Disorder/	7760
13	exp Phobias/	13434
14	Posttraumatic Stress Disorder/	34688
15	Anxiety/	65640
16	(agoraphobi* or anxi* or obsessive-compulsive or panic or phobi* or post-traumatic stress or posttraumatic stress).ti,ab,id.	283726

For peer review only - http://bmjopen.bmj.com/site/about/guidelines.xhtml

Karolinska Institutet

17	or/8-16	296637
18	Affective Disorders/	14669
19	exp Major Depression/	138828
20	"Depression (Emotion)"/	26043
21	depress*.ti,ab,id.	321600
22	((affective or mood) adj2 disorder*).ti,ab,id.	35856
23	or/18-22	343842
24	17 and 23	109245
25	(emotional adj2 disorder*).ti,ab,id.	5905
26	(transdiagnost* or unified protocol).ti,ab,id.	3036
27	25 or 26	8661
28	24 or 27	115823
29	Internet/	29614
30	Digital Interventions/	593
31	Computer Assisted Therapy/	1140
32	exp Telemedicine/	9675
33	Digital Technology/	719
34	Videoconferencing/	645
35	Mobile Applications/	1193
36	(app or apps or cellphone* or computer* or digital or e mail or email or internet* or mobile application* or online or phone* or smartphone* or technolog* or telephone* or text message* or video* or virtual or web based or web site or website).ti,ab,id.	436545
37	(distance counsel?ing or distance consultation* or e consultation* or econsultation* or e counsel?ing or ecounsel?ing or e health* or ehealth* or e therapies or e therapy or etherap* or e visit* or evisit* or m health or mhealth or mobile counsel?ing or mobile consultation* or remote consultation* or remote counsel?ing or tele health* or telehealth* or tele consultation* or teleconsultation* or tele medicine or telemedicine or tele rehabilitation or telerehabilitation).ti,ab,id.	7477
38	or/29-37	440321
39	exp Randomized Controlled Trials/	954
40	randomi?ed.ab.	83970
41	placebo.ab.	40594
42	randomly.ab.	76127
43	trial.ti.	32809

For peer review only - http://bmjopen.bmj.com/site/about/guidelines.xhtml

Karolinska Institutet

44	or/39-43	183930
45	(systematic review or meta-analy*).ti.	40744
46	44 not 45	176278
47	7 and 28 and 38 and 46	1037
48	limit 47 to english language	983
49	limit 48 to yr="1995 -Current"	981
50	limit 49 to "0100 journal"	898

For peer review only

Systematic search: 2023 updated main search

For peer review only

Karolinska Institutet

Documentation of search strategies
University Library search consultation group

Date: February 2023

Topic/research question: Systematic review and meta-analysis of transdiagnostic psychological interventions delivered via the internet for individuals suffering from common mental disorders (PROSPERO: CRD42021243172)

Name of researcher(s): Erland Axelsson

Librarian(s): Sabina Gillsund & Emma-Lotta Säätelä

Databases:

1. Medline (Ovid)
2. Cochrane Library (Wiley)
3. Web of Science Core Collection (Clarivate Analytics)
4. PsycInfo (EBSCO)

Total number of hits:

- Before deduplication: 10,075
- After deduplication: 4,909

---

1. Medline

Interface: Ovid MEDLINE(R) ALL<br><br>Date of Search: 6 February 2023<br><br>Number of hits: 2,602<br><br>Comment: In Ovid, two or more words are automatically searched as phrases; i.e. no quotation marks are needed	Field labels<br><br>• exp/ = exploded MeSH term<br>• / = non exploded MeSH term<br>• .ti,ab,kf. = title, abstract and author keywords<br>• adjx = within x words, regardless of order<br>• * = truncation of word for alternate endings

For peer review only - http://bmjopen.bmj.com/site/about/guidelines.xhtml

For peer review only

Karolinska Institutet

Database(s): **Ovid MEDLINE(R) ALL** 1946 to February 03, 2023
Search Strategy:

#	Searches	Results
1	exp Psychotherapy/	215915
2	(("acceptance and commitment" or affect-focused or behavio?r* or biofeedback or computeri* or cognit* or digital or "emotional awareness and expression" or educat* or exposure or guided or internet* or interpersonal or mobile or online or positive psychology or psychodynamic or psychologic* or self-guided or self-help or transdiagnost* or web*) adj2 (intervention* or therap* or treat* or program*)).ti,ab,kf.	220048
3	(behavio?ral activation or bias modification or cognitive restructuring or hypno* or meditation or mindfulness or problem solving or psychoeducation* or psychotherap* or relaxation or self-management or unified protocol).ti,ab,kf.	277967
4	or/1-3	597258
5	Anxiety Disorders/	40399
6	Agoraphobia/	2669
7	Neurotic Disorders/	18000
8	Obsessive-Compulsive Disorder/	16224
9	Panic Disorder/	7250
10	exp Phobic Disorders/	12293
11	Stress Disorders, Post-Traumatic/	40198
12	Anxiety/	103449
13	(agoraphobi* or anxi* or obsessive-compulsive or panic or phobi* or post-traumatic stress or posttraumatic stress).ti,ab,kf.	332361
14	or/5-13	389293
15	Mood Disorders/	15751
16	Depressive Disorder/	74889
17	Depressive Disorder, Major/	37299
18	Affective Symptoms/	13816
19	Depression/	146908
20	depress*.ti,ab,kf.	552903
21	((affective or mood) adj2 disorder*).ti,ab,kf.	40988
22	or/15-21	627024
23	14 and 22	168605

For peer review only - http://bmjopen.bmj.com/site/about/guidelines.xhtml

Karolinska Institutet

24	(emotional adj2 disorder*).ti,ab,kf.	4243
25	(transdiagnost* or unified protocol).ti,ab,kf.	4104
26	24 or 25	8093
27	23 or 26	174000
28	Internet/	80600
29	Internet-Based Intervention/	1071
30	Therapy, Computer-Assisted/	6968
31	Telemedicine/	36073
32	Telerehabilitation/	900
33	exp Remote Consultation/	5715
34	Mobile Applications/	10971
35	Videoconferencing/	2295
36	(app or apps or cellphone* or computer* or digital or e mail or email or internet* or mobile application* or online or phone* or smartphone* or technolog* or telephone* or text message* or video* or virtual or web based or web site or website).ti,ab,kf.	1678151
37	(distance counsel?ing or distance consultation* or e consultation* or econsultation* or e counsel?ing or ecounsel?ing or e health* or ehealth* or e therapies or e therapy or etherap* or e visit* or evisit* or m health or mhealth or mobile counsel?ing or mobile consultation* or remote consultation* or remote counsel?ing or tele health* or telehealth* or tele consultation* or teleconsultation* or tele medicine or telemedicine or tele rehabilitation or telerehabilitation).ti,ab,kf.	51873
38	or/28-37	1730206
39	randomized controlled trial.pt.	585953
40	controlled clinical trial.pt.	95177
41	randomi?ed.ab.	708985
42	placebo.ab.	235960
43	clinical trials as topic.sh.	200855
44	randomly.ab.	402423
45	trial.ti.	279911
46	or/39-45	1550232
47	(systematic review or meta-analy*).ti.	290755
48	46 not 47	1464360
49	exp animals/ not humans.sh.	5089811
50	48 not 49	1343292

For peer review only - http://bmjopen.bmj.com/site/about/guidelines.xhtml

Karolinska Institutet

51	4 and 27 and 38 and 50	2627
52	limit 51 to english language	2609
53	limit 52 to yr="1995 -Current"	2602

For peer review only

Karolinska Institutet

2. Cochrane Library

Interface: Wiley<br><br>Date of Search: 3 February 2023<br><br>Number of hits: 3,068	Field labels<br><br>• ti,ab,kw = title, abstract and author keywords<br>• NEAR/x = within x words, regardless of order<br>• * = truncation of word for alternate endings

ID	Search	Hits
#1	MeSH descriptor: [Psychotherapy] explode all trees	30769
#2	(("acceptance and commitment" or "affect-focused" or behavio?r* or "biofeedback" or computeri* or cognit* or "digital" or "emotional awareness and expression" or educat* or "exposure" or "guided" or internet* or "interpersonal" or "mobile" or "online" or "positive psychology" or "psychodynamic" or psychologic* or "self-guided" or "self-help" or transdiagnost* or web*) NEAR/2 (intervention* or therap* or treat* or program*)):ti,ab,kw	95851
#3	((behavio?ral NEXT activation) or "bias modification" or "cognitive restructuring" or hypno* or "meditation" or mindfulness or "problem solving" or psychoeducation* or psychotherap* or "relaxation" or "self-management" or "unified protocol"):ti,ab,kw	64668
#4	#1 or #2 or #3	143208
#5	MeSH descriptor: [Anxiety Disorders] this term only	5112
#6	MeSH descriptor: [Agoraphobia] this term only	472
#7	MeSH descriptor: [Neurotic Disorders] this term only	316
#8	MeSH descriptor: [Obsessive-Compulsive Disorder] this term only	1250
#9	MeSH descriptor: [Panic Disorder] this term only	1053
#10	MeSH descriptor: [Phobic Disorders] explode all trees	1942
#11	MeSH descriptor: [Stress Disorders, Post-Traumatic] this term only	3561
#12	MeSH descriptor: [Anxiety] this term only	9845
#13	(agoraphobi* or anxi* or "obsessive compulsive" or "obsessive-compulsive" or "panic" or phobi* or "post-traumatic stress" or "posttraumatic stress"):ti,ab,kw	76993
#14	#5 or #6 or #7 or #8 or #9 or #10 or #11 or #12 or #13	77700
#15	MeSH descriptor: [Mood Disorders] this term only	1004
#16	MeSH descriptor: [Depressive Disorder] this term only	9053
#17	MeSH descriptor: [Depressive Disorder, Major] this term only	6360
#18	MeSH descriptor: [Affective Symptoms] this term only	514
#19	MeSH descriptor: [Depression] this term only	15880
#20	depress*:ti,ab,kw	103690
#21	(("affective" or "mood") NEAR/3 disorder*):ti,ab,kw	6072
#22	#15 or #16 or #17 or #18 or #19 or #20 or #21	106436
#23	#14 and #22	38854
#24	("emotional" NEAR/2 disorder*):ti,ab,kw	1098
#25	(transdiagnost* or "unified protocol"):ti,ab,kw	1061
#26	#24 or #25	1974
#27	#23 or #26	39878

For peer review only - http://bmjopen.bmj.com/site/about/guidelines.xhtml

Karolinska Institutet

#28	MeSH descriptor: [Internet] this term only	4780
#29	MeSH descriptor: [Internet-Based Intervention] this term only	527
#30	MeSH descriptor: [Therapy, Computer-Assisted] this term only	1476
#31	MeSH descriptor: [Telemedicine] this term only	3338
#32	MeSH descriptor: [Telerehabilitation] this term only	229
#33	MeSH descriptor: [Remote Consultation] 3 tree(s) exploded	440
#34	MeSH descriptor: [Mobile Applications] this term only	1476
#35	MeSH descriptor: [Videoconferencing] this term only	259
#36	("app" or "apps" or cellphone* or computer* or "digital" or "e mail" or "email" or internet* or (mobile NEXT application*) or "online" or phone* or smartphone* or technolog* or telephone* or (text NEXT message*) or video* or "virtual" or "web based" or "web site" or "website"):ti,ab,kw	184801
#37	(("distance" NEXT counsel?ing) or ("distance" NEXT consultation*) or ("e" NEXT consultation*) or econsultation* or ("e" NEXT counsel?ing) or ecounsel?ing or ("e" NEXT health*) or ehealth* or "e therapies" or "e therapy" or etherap* or ("e" NEXT visit*) or evisit* or "m health" or "mhealth" or ("mobile" NEXT counsel?ing) or ("mobile" NEXT consultation*) or ("remote" NEXT consultation*) or ("remote" NEXT counsel?ing) or ("tele" NEXT health*) or telehealth* or ("tele" NEXT consultation*) or teleconsultation* or "tele medicine" or "telemedicine" or "tele rehabilitation" or "telerehabilitation"):ti,ab,kw	12716
#38	#28 or #29 or #30 or #31 or #32 or #33 or #34 or #35 or #36 or #37	188389
#39	#4 and #27 and #38	6085
#40	(clinicaltrials or trialsearch):so	444610
#41	#39 NOT #40	3156
	Limit to publication date 1995-2023 in Trials	3068

For peer review only

Karolinska Institutet

3. Web of Science Core Collection

Interface: Clarivate Analytics<br><br>Date of Search: 3 February 2023<br><br>Number of hits: 3,010	Field labels<br><br>• TS/Topic = title, abstract, author keywords and Keywords Plus<br>• NEAR/x = within x words, regardless of order<br>• * = truncation of word for alternate endings<br><br>Note: sometimes “quotation marks” are needed for single search terms to avoid automatic term mapping (lemmatization).

18 #14 AND #11 AND #8 AND #3

Refined by: LANGUAGES: (ENGLISH) AND [excluding] PUBLICATION YEARS: (1994 OR 1993) AND DOCUMENT TYPES: (ARTICLE OR CORRECTION OR EARLY ACCESS)

17 #14 AND #11 AND #8 AND #3

Refined by: LANGUAGES: (ENGLISH) AND [excluding] PUBLICATION YEARS: (1994 OR 1993)

16 #14 AND #11 AND #8 AND #3

Refined by: LANGUAGES: (ENGLISH)

15 #14 AND #11 AND #8 AND #3

14 #12 NOT #13

13 TI=(“systematic review” or “meta-analy*”)

12 TI=(“randomi$ed” OR “randomi$ed” OR “randomi$ation” OR “randomi$ation” OR placebo* OR (random* AND (allocat* OR assign*)) OR (blind* AND (“single” OR “double” OR “treble” OR “triple”))) OR AB=(“randomi$ed” OR “randomi$ed” OR “randomi$ation” OR “randomi$ation” OR placebo* OR (random* AND (allocat* OR assign*)) OR (blind* AND (“single” OR “double” OR “treble” OR “triple”)))

11 #10 OR #9

10 TS=(“distance counsel$ing” or “distance consultation*” or “e consultation*” or econsultation* or “e counsel$ing” or “ecounsel$ing” or “e health*” or ehealth* or “e therapies” or “e therapy” or etherap* or “e visit*” or evisit* or “m health” or “mhealth” or “mobile counsel$ing” or “mobile consultation*” or “remote consultation*” or “remote counsel$ing” or “tele health*” or telehealth* or “tele consultation*” or teleconsultation* or “tele medicine” or “telemedicine” or “tele rehabilitation” or “telerehabilitation")

For peer review only - http://bmjopen.bmj.com/site/about/guidelines.xhtml

9 TS=(“app” or “apps” or cellphone* or computer* or “digital” or “e mail” or “email” or internet* or “mobile application*” or “online” or phone* or smartphone* or technolog* or telephone* or “text message*” or video* or “virtual” or “web based” or “web site” or “website”)

8 #7 OR #6

7 TS=((”emotional” NEAR/1 disorder*) or transdiagnost* or “unified protocol”)

6 #5 AND #4

5 TS=(depress* or ((“affective” or “mood”) NEAR/1 disorder*))

4 TS=(agoraphobi* or anxi* or “obsessive-compulsive” or “panic” or phobi* or “post-traumatic stress” or “posttraumatic stress”)

3 #2 OR #1

2 TS=((“behavio$ral activation” or “bias modification” or “cognitive restructuring” or hypno* or “meditation” or “mindfulness” or “problem solving” or psychoeducation* or psychotherap* or “relaxation” or “self-management” or “unified protocol”))

1 TS=((("acceptance and commitment" or “affect-focused” or behavio$r* or “biofeedback” or computeri* or cognit* or “digital” or "emotional awareness and expression" or educat* or “exposure” or “guided” or internet* or “interpersonal” or “mobile” or “online” or “positive psychology” or “psychodynamic” or psychologic* or “self-guided” or “self-help” or transdiagnost* or web*) NEAR/1 (intervention* or therap* or treat* or program*)))

For peer review only - http://bmjopen.bmj.com/site/about/guidelines.xhtml

Karolinska Institutet

4. Psycinfo

Interface: EBSCO<br><br>Date of Search: 6 february 2023<br><br>Number of hits: 1,395	Field labels<br><br>• DE = subject heading<br>• TI = title<br>• AB = abstract<br>• KW = author keywords<br>• Nx = within x words, regardless of order<br>• * = truncation of word for alternate endings

S47	S45 NOT S36<br><br>Publication year: 1995-2023<br>Language: - english	1,395
S45	S44 NOT S36	1,418
S44	S7 AND S32 AND S35 AND S43	1,572
S43	S39 OR S42	135,063
S42	S40 AND S41	125,758
S41	S17 OR S18 OR S19 OR S20 OR S21	375,079
S40	S8 OR S9 OR S10 OR S11 OR S12 OR S13 OR S14 OR S15 OR S16	332,986
S39	S37 OR S38	12,089
S38	TI (transdiagnost* or "unified protocol") OR AB (transdiagnost* or "unified protocol") OR KW (transdiagnost* or "unified protocol")	4,261
S37	TI emotional N2 disorder* OR AB emotional N2 disorder* OR KW emotional N2 disorder*	8,225
S36	TI "systematic review" or meta-analy*	50,479
S35	S33 OR S34	206,233
S34	AB randomi?ed OR AB placebo OR AB randomly OR TI trial	206,178
S33	DE "Randomized Controlled Trials" OR DE "Randomized Clinical Trials"	1,387
S32	S23 OR S24 OR S25 OR S26 OR S27 OR S28 OR S29 OR S30 OR S31	504,430
S31	TI (“distance counsel#ing” or “distance consultation*” or “e consultation*” or econsultation* or “e counsel#ing” or “ecounsel#ing” or “e health*” or ehealth* or “e therapies” or “e therapy” or etherap* or “e visit*” or evisit* or “m health” or “mhealth” or “mobile counsel#ing” or “mobile consultation*” or “remote consultation*” or “remote counsel#ing” or “tele health*” or telehealth* or “tele consultation*” or teleconsultation* or “tele medicine” or “telemedicine” or “tele rehabilitation” or “telerehabilitation”) OR AB (“distance counsel#ing” or “distance consultation*” or “e consultation*” or econsultation* or “e counsel#ing” or “ecounsel#ing” or “e health*” or ehealth* or “e therapies” or “e therapy” or etherap* or “e visit*” or evisit* or “m health” or “mhealth” or “mobile counsel#ing” or “mobile consultation*” or “remote consultation*” or “remote counsel#ing” or “tele health*” or telehealth* or “tele consultation*” or teleconsultation* or “tele medicine” or “telemedicine” or “tele rehabilitation” or “telerehabilitation”) OR KW (“distance counsel#ing” or “distance consultation*” or “e consultation*” or econsultation* or “e counsel#ing” or “ecounsel#ing” or “e health*” or ehealth* or “e therapies” or “e therapy” or etherap* or “e visit*” or evisit* or “m health” or “mhealth” or “mobile counsel#ing” or “mobile consultation*” or “remote consultation*” or “remote counsel#ing” or “tele health*” or telehealth* or “tele consultation*” or teleconsultation* or “tele medicine” or “telemedicine” or “tele rehabilitation” or “telerehabilitation”)	10,426
S30	TI (app or apps or cellphone* or computer* or digital or “e mail” or email or internet* or “mobile application*” or online or phone* or smartphone* or technolog* or telephone* or “text message*” or video* or virtual or “web based” or “web site” or website) OR AB (app or apps	498,437

For peer review only - http://bmjopen.bmj.com/site/about/guidelines.xhtml

	or cellphone* or computer* or digital or “e mail” or email or internet* or “mobile application*” or online or phone* or smartphone* or technolog* or telephone* or “text message*” or video* or virtual or “web based” or “web site” or website) OR KW (app or apps or cellphone* or computer* or digital or “e mail” or email or internet* or “mobile application*” or online or phone* or smartphone* or technolog* or telephone* or “text message*” or video* or virtual or “web based” or “web site” or website)	
S29	DE "Mobile Applications"	2,881
S28	DE "Videoconferencing"	1,138
S27	DE "Digital Technology"	1,488
S26	DE "Telemedicine" OR DE "Online Therapy" OR DE "Teleconferencing" OR DE "Teleconsultation" OR DE "Telepsychiatry" OR DE "Telepsychology" OR DE "Telerehabilitation" OR DE "Teleconferencing" OR DE "Videoconferencing"	13,791
S25	DE "Computer Assisted Therapy"	1,211
S24	DE "Digital Interventions"	1,160
S23	DE "Internet"	38,434
S22	S17 OR S18 OR S19 OR S20 OR S21	375,079
S21	TI (((affective or mood) N2 disorder*)) OR AB (((affective or mood) N2 disorder*)) OR KW (((affective or mood) N2 disorder*))	41,246
S20	TI depress* OR AB depress* OR KW depress*	349,996
S19	DE "Depression (Emotion)"	26,821
S18	DE "Major Depression" OR DE "Anaclitic Depression" OR DE "Dysthymic Disorder" OR DE "Endogenous Depression" OR DE "Late Life Depression" OR DE "Postpartum Depression" OR DE "Reactive Depression" OR DE "Recurrent Depression" OR DE "Treatment Resistant Depression"	154,908
S17	DE "Affective Disorders"	15,668
S16	TI (agoraphobi* or anxi* or obsessive-compulsive or panic or phobi* or “post-traumatic stress” or “posttraumatic stress”)) OR AB (agoraphobi* or anxi* or obsessive-compulsive or panic or phobi* or “post-traumatic stress” or “posttraumatic stress”)) OR KW (agoraphobi* or anxi* or obsessive-compulsive or panic or phobi* or “post-traumatic stress” or “posttraumatic stress”))	310,917
S15	DE "Anxiety"	94,782
S14	DE "Posttraumatic Stress Disorder"	38,806
S13	DE "Phobias" OR DE "Acrophobia" OR DE "Agoraphobia" OR DE "Claustrophobia" OR DE "Ophidiophobia" OR DE "School Phobia" OR DE "Social Phobia"	14,824
S12	DE "Panic Disorder"	9,312
S11	DE "Obsessive Compulsive Disorder"	15,724
S10	DE "Neurosis"	7,143
S9	DE "Agoraphobia"	3,756
S8	DE "Anxiety Disorders"	34,816
S7	S1 OR S2 OR S3 OR S4 OR S5 OR S6	579,216
S6	TI (“behavio#ral activation” or “bias modification” or “cognitive restructuring” or hypno* or meditation or mindfulness or “problem solving” or psychoeducation* or psychotherap* or relaxation or self-management or “unified protocol”) OR AB (“behavio#ral activation” or “bias modification” or “cognitive restructuring” or hypno* or meditation or mindfulness or “problem solving” or psychoeducation* or psychotherap* or relaxation or self-management or “unified protocol”) OR KW (“behavio#ral activation” or “bias modification” or “cognitive restructuring” or hypno* or meditation or mindfulness or “problem solving” or psychoeducation* or psychotherap* or relaxation or self-management or “unified protocol”)	256,300
S5	TI ((("acceptance and commitment" or affect-focused or behavio#r* or biofeedback or computeri* or cognit* or digital or "emotional awareness and expression" or educat* or exposure or guided or internet* or interpersonal or mobile or online or positive psychology or psychodynamic or psychologic* or self-guided or self-help or transdiagnost* or web*) N2	229,986

For peer review only - http://bmjopen.bmj.com/site/about/guidelines.xhtml

Karolinska Institutet

	(intervention* or therap* or treat* or program*))) OR AB ((("acceptance and commitment" or affect-focused or behavio#r* or biofeedback or computeri* or cognit* or digital or "emotional awareness and expression" or educat* or exposure or guided or internet* or interpersonal or mobile or online or positive psychology or psychodynamic or psychologic* or self-guided or self-help or transdiagnost* or web*) N2 (intervention* or therap* or treat* or program*))) OR KW ((("acceptance and commitment" or affect-focused or behavio#r* or biofeedback or computeri* or cognit* or digital or "emotional awareness and expression" or educat* or exposure or guided or internet* or interpersonal or mobile or online or positive psychology or psychodynamic or psychologic* or self-guided or self-help or transdiagnost* or web*) N2 (intervention* or therap* or treat* or program*)))	
S4	DE "Cognitive Behavior Therapy" OR DE "Acceptance and Commitment Therapy" OR DE "Cognitive Processing Therapy" OR DE "Prolonged Exposure Therapy"	26,182
S3	DE "Behavior Therapy" OR DE "Aversion Therapy" OR DE "Conversion Therapy" OR DE "Dialectical Behavior Therapy" OR DE "Exposure Therapy" OR DE "Implosive Therapy" OR DE "Reciprocal Inhibition Therapy" OR DE "Response Cost" OR DE "Systematic Desensitization Therapy" OR DE "Aversion Therapy" OR DE "Covert Sensitization" OR DE "Exposure Therapy" OR DE "Imaginal Exposure" OR DE "Implosive Therapy" OR DE "In Vivo Exposure" OR DE "Prolonged Exposure Therapy" OR DE "Systematic Desensitization Therapy" OR DE "Virtual Reality Exposure Therapy"	34,636
S2	DE "Cognitive Therapy"	24,241
S1	DE "Psychotherapy" OR DE "Adlerian Psychotherapy" OR DE "Adolescent Psychotherapy" OR DE "Affirmative Therapy" OR DE "Analytical Psychotherapy" OR DE "Autogenic Training" OR DE "Brief Psychotherapy" OR DE "Brief Relational Therapy" OR DE "Child Psychotherapy" OR DE "Client Centered Therapy" OR DE "Conversion Therapy" OR DE "Couples Therapy" OR DE "Eclectic Psychotherapy" OR DE "Emotion Focused Therapy" OR DE "Existential Therapy" OR DE "Experiential Psychotherapy" OR DE "Expressive Psychotherapy" OR DE "Eye Movement Desensitization Therapy" OR DE "Feminist Therapy" OR DE "Geriatric Psychotherapy" OR DE "Gestalt Therapy" OR DE "Group Psychotherapy" OR DE "Guided Imagery" OR DE "Humanistic Psychotherapy" OR DE "Hypnotherapy" OR DE "Individual Psychotherapy" OR DE "Insight Therapy" OR DE "Integrative Psychotherapy" OR DE "Interpersonal Psychotherapy" OR DE "Logotherapy" OR DE "Narrative Therapy" OR DE "Network Therapy" OR DE "Persuasion Therapy" OR DE "Primal Therapy" OR DE "Psychoanalysis" OR DE "Psychodrama" OR DE "Psychodynamic Psychotherapy" OR DE "Psychotherapeutic Counseling" OR DE "Psychotherapeutic Techniques" OR DE "Rational Emotive Behavior Therapy" OR DE "Reality Therapy" OR DE "Relationship Therapy" OR DE "Solution Focused Therapy" OR DE "Strategic Therapy" OR DE "Supportive Psychotherapy" OR DE "Transactional Analysis" OR DE "Adolescent Psychotherapy" OR DE "Multisystemic Therapy" OR DE "Child Psychotherapy" OR DE "Play Therapy" OR DE "Gestalt Therapy" OR DE "Empty Chair Technique" OR DE "Group Psychotherapy" OR DE "Encounter Group Therapy" OR DE "Therapeutic Community" OR DE "Humanistic Psychotherapy" OR DE "Client Centered Therapy" OR DE "Hypnotherapy" OR DE "Age Regression (Hypnotic)" OR DE "Ericksonian Psychotherapy" OR DE "Posthypnotic Suggestions" OR DE "Integrative Psychotherapy" OR DE "Schema Therapy" OR DE "Psychoanalysis" OR DE "Adlerian Psychotherapy" OR DE "Brief Relational Therapy" OR DE "Dream Analysis" OR DE "Self-Analysis" OR DE "Psychotherapeutic Counseling" OR DE "Family Therapy" OR DE "Psychotherapeutic Techniques" OR DE "Active Listening" OR DE "Animal Assisted Therapy" OR DE "Autogenic Training" OR DE "Brief Relational Therapy" OR DE "Centering" OR DE "Cotherapy" OR DE "Dream Analysis" OR DE "Empty Chair Technique" OR DE "Ericksonian Psychotherapy" OR DE "Free Association" OR DE "Guided Imagery" OR DE "Life Review" OR DE "Mirroring" OR DE "Morita Therapy" OR DE "Motivational Interviewing" OR DE "Mutual Storytelling Technique" OR DE "Network Therapy" OR DE "Paradoxical Techniques" OR DE "Psychodrama" OR DE "Strategic Therapy" OR DE "Strategic Family Therapy"	237,062

For peer review only - http://bmjopen.bmj.com/site/about/guidelines.xhtml

Internet-delivered transdiagnostic psychological treatments for individuals with depression, anxiety, or both: a systematic review and meta-analysis of randomized controlled trials

Randomized controlled trials per year of publication

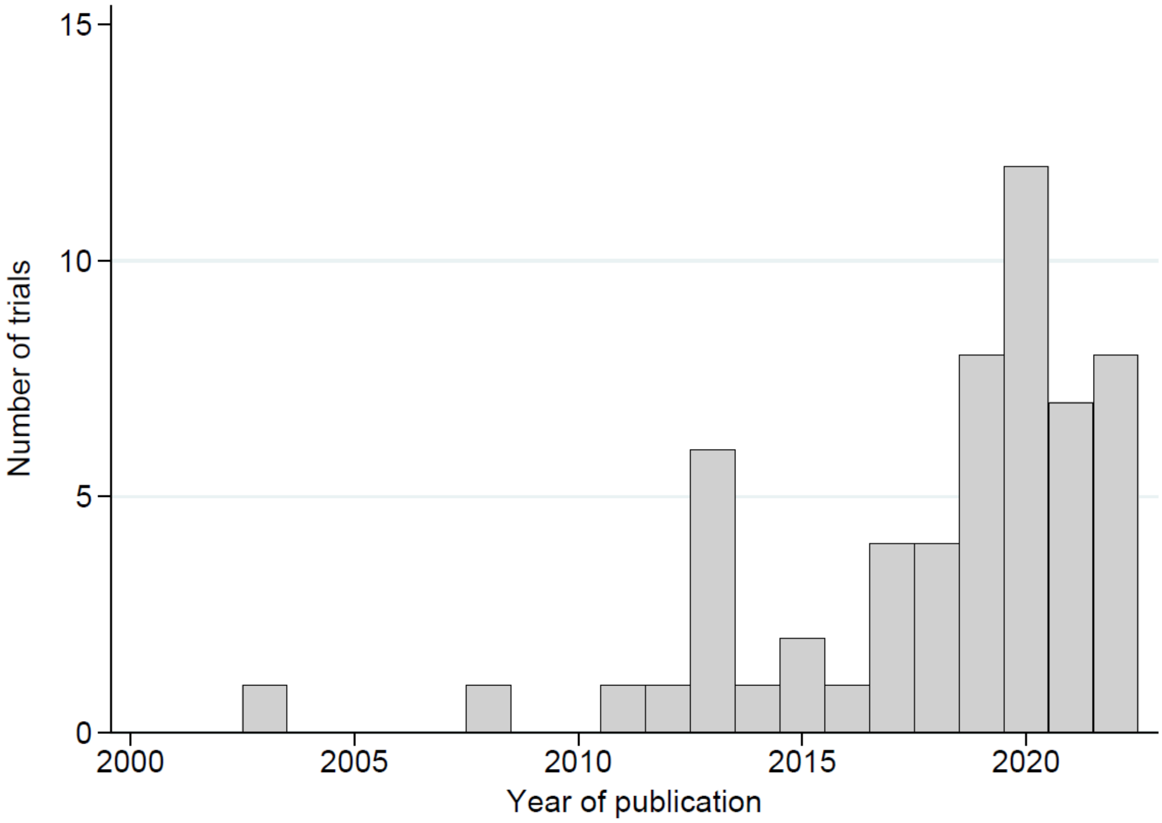

Internet-delivered transdiagnostic psychological treatments for individuals with depression, anxiety, or both: a systematic review and meta-analysis of randomized controlled trials

Exclusion criteria discussed with regard to disagreements

There were 45 disagreements:

- 18 concerned criterion d, i.e., whether the study recruited a mix of participants suffering from clinically significant depression and anxiety
- 15 concerned criterion c, i.e., whether the study evaluated the efficacy of an internet-delivered transdiagnostic psychological treatment for anxiety and depression
- 6 concerned criterion e, i.e., whether the sample composition was such that it was unreasonable to generalize to typical patients with depression and anxiety disorders
- 4 concerned criterion b, i.e., whether the text under consideration was a primary publication from a randomized controlled trial
- 1 concerned criterion a, i.e., whether the text under consideration was a peer reviewed article written in English
- 1 concerned criterion f, i.e., whether there was at least 10 participants in at least one internet-delivered transdiagnostic psychological treatment arm

For peer review only

Internet-delivered transdiagnostic psychological treatments for individuals with depression, anxiety, or both: a systematic review and meta-analysis of randomized controlled trials

Trial characteristics

Table S.B.1. Trial characteristics including references

Reference	Year	Country	Age, M	Women, %	N	Duration	Format	Treatment type/school	Missing, %
Al-Refae, et al. [1]	2021	Canada	25	79	245	< 6 weeks	app	mixed CBT/mindfulness	41
Batterham, et al. [2]	2021	Australia		85	1986	< 6 weeks	mixed/other	CBT	66
Beshai, et al. [3]	2020	Canada	35	44	456	< 6 weeks	mixed/other	mindfulness based	65
Borjalilu, et al. [4]	2019	Iran	24	71	68	≥ 6 weeks	app	mindfulness based	
Carolan, et al. [5]	2017	United Kingdom	41	85	84	≥ 6 weeks	website	CBT	26
Dear, et al. [6]	2018	Australia	21	82	217	< 6 weeks	website	CBT	28
Diaz-Garcia, et al. [7]	2021	Spain	34	72	216	≥ 6 weeks	mixed/other	CBT	32
Farrer, et al. [8]	2019	Australia	22	78	200	≥ 6 weeks	mixed/other	mixed CBT/mindfulness	28
Fitzpatrick, et al. [9]	2017	United States	22	67	70	< 6 weeks	mixed/other	CBT	20
Gonzalez-Robles, et al. [10]	2020	Spain	38	69	214	≥ 6 weeks	website	CBT	39
Graham, et al. [11]	2020	United States	42	82	146	≥ 6 weeks	app	CBT	4
Gregoire, et al. [12]	2022	Canada	26	77	107	< 6 weeks	video	CBT	31
Hadjistavropoulos, et al. [13]	2017	Canada	38	79	182	≥ 6 weeks	website	CBT	25
Hadjistavropoulos, et al. [14]	2020a	Canada	37	76	744	≥ 6 weeks	website	CBT	31
Hadjistavropoulos, et al. [15]	2020b	Canada	37	72	631	≥ 6 weeks	website	CBT	35
Hadjistavropoulos, et al. [16]	2022	Canada	36	76	469	≥ 6 weeks	website	CBT	27
Hensel, et al. [17]	2019	Canada	41	73, 78	812	≥ 6 weeks	website	CBT	45
Hirsch, et al. [18]	2020	United Kingdom	29	83	178	< 6 weeks	website	other	12
Hoek, et al. [19]	2012	The Netherlands	16	76	45	< 6 weeks	website	other	38
Hosseinzadeh Asl [20]	2022	Turkey	33	55	59	< 6 weeks	video	mindfulness based	17
Isbăşoiu, et al. [21]	2021	Romania	33	85	284	≥ 6 weeks	website	CBT	45

For peer review only

Johansson, et al. [22]	2013	Sweden	45	82	100	≥ 6 weeks	website	other	0
Kang, et al. [23]	2022	China	37	53	100	< 6 weeks	mixed/other	mindfulness based	0
Kladnitski, et al. [24]	2020	Australia	39	86	158	≥ 6 weeks	website	mixed CBT/mindfulness	27
Kleiboer, et al. [25]	2015	The Netherlands	45	65	537	< 6 weeks	website	other	22
Krafft, et al. [26]	2019	United States	25	66	35	< 6 weeks	app	CBT	60
Levin, et al. [27]	2022	United States	20	100	23	< 6 weeks	app	mindfulness based	30
Lindegaard, et al. [28]	2021	Sweden	38	42	59	≥ 6 weeks	website	CBT	39
McCloud, et al. [29]	2020	United Kingdom	24	83	168	≥ 6 weeks	app	CBT	42
Mohr, et al. [30]	2019	United States	36	76	301	≥ 6 weeks	app	CBT	3
Mullin, et al. [31]	2015	Australia	28	64	55	≥ 6 weeks	website	CBT	24
Newby, et al. [32]	2013	Australia	44	78	109	≥ 6 weeks	website	CBT	12
Nilsson, et al. [33]	2019	Sweden			837	≥ 6 weeks	website	other	67
Osborn, et al. [34]	2020	Kenya	15	64	103	< 6 weeks	website	other	0
Peynenburg, et al. [35]	2022	Canada	24	81	308	< 6 weeks	website	CBT	29
Powell, et al. [36]	2012	United Kingdom	41	78	3070	≥ 6 weeks	website	CBT	42
Proudfoot, et al. [37]	2013	Australia	39	70	720	≥ 6 weeks	website	other	28
Robichaud, et al. [38]	2020	Canada	35	87	63	≥ 6 weeks	website	CBT	32
Rogers and Sicouri [39]	2022	Australia	20	55	45	< 6 weeks	website	other	3
Rollman, et al. [40]	2018	United States	43	80	704	≥ 6 weeks	website	CBT	14
Schaeuffele, et al. [41]	2022	Germany	37	68	132	≥ 6 weeks	website	CBT	25
Sethi [42]	2013	Australia	20	58	89	< 6 weeks	website	CBT	0
Soucy, et al. [43]	2021	Canada	38	75	480	≥ 6 weeks	website	CBT	30
Southwell and Gould [44]	2016	Australia	34	88	120	< 6 weeks	website	other	52
Taylor, et al. [45]	2022	United Kingdom	41	83	2182	< 6 weeks	app	mindfulness based	35
Terides, et al. [46]	2018	Australia	45	88, 75	148	≥ 6 weeks	website	CBT	18
Titov, et al. [47]	2011	Australia	44	73	77	≥ 6 weeks	website	CBT	10

For peer review only

Internet-delivered transdiagnostic psychological treatments for individuals with depression, anxiety, or both: a systematic review and meta-analysis of randomized controlled trials

Titov, et al. [48]	2013	Australia	41	74	274	≥ 6 weeks	website	CBT	20
Titov, et al. [49]	2016	Australia	66	64	459	≥ 6 weeks	website	CBT	9
Tönnies, et al. [50]	2021	Germany	49	70	50	≥ 6 weeks	video	other	10
Tulbure, et al. [51]	2018	Romania	34	81	105	≥ 6 weeks	website	CBT	8
Twomey, et al. [52]	2014	Ireland	35	74	201	< 6 weeks	website	CBT	67
van Straten, et al. [53]	2008	The Netherlands	45	71	213	< 6 weeks	website	other	17
Villemaire-Krajden and Myhr [54]	2019	Canada	38	67	67	≥ 6 weeks	mixed/other	CBT	31
Viravan, et al. [55]	2022	Thailand	34	32	80	< 6 weeks	mixed/other	mindfulness based	4
Viskovich and Pakenham [56]	2020	Australia	27	68	2110	< 6 weeks	mixed/other	CBT	68
Zetterqvist, et al. [57]	2003	Sweden	39	57, 65	100	≥ 6 weeks	website	CBT	37

For peer review only

Internet-delivered transdiagnostic psychological treatments for individuals with depression, anxiety, or both: a systematic review and meta-analysis of randomized controlled trials

Publication bias

We explored indicators of publication bias in the meta-analysis of effects versus treatment-as-usual and waitlist controls. For depression, the funnel plot (see below) appeared to indicate that small- to medium-sized trials with non-beneficial effects of internet-delivered psychological treatments were underreported. This asymmetry was confirmed by Egger's test ($z = 2.24$, $p = 0.025$). The trim-and-fill procedure resulted in different outcomes depending on the choice of estimator. With the R0 estimator ($p < 0.001$), 19 studies were imputed, and the between-group effect dropped from 0.52 to 0.29 (95% CI 0.16-0.42). In contrast, the L0 and Q0 estimators resulted in no imputation. Visual inspection of the funnel plot for anxiety (see below) was also indicative of possible asymmetry in the same direction, but this was more ambiguous. Egger's test was significant ($z = 2.64$, $p = 0.008$) but the trim-and-fill procedure did not result in imputation, regardless of estimator. As is recommended in high heterogeneity situations,[58] we repeated all tests of publication bias in a manner informed by the moderator analyses and results were similar (see below).

For peer review only

Internet-delivered transdiagnostic psychological treatments for individuals with depression, anxiety, or both: a systematic review and meta-analysis of randomized controlled trials

Meta-analysis versus treatment-as-usual and waitlist controls, with depression symptoms as outcome

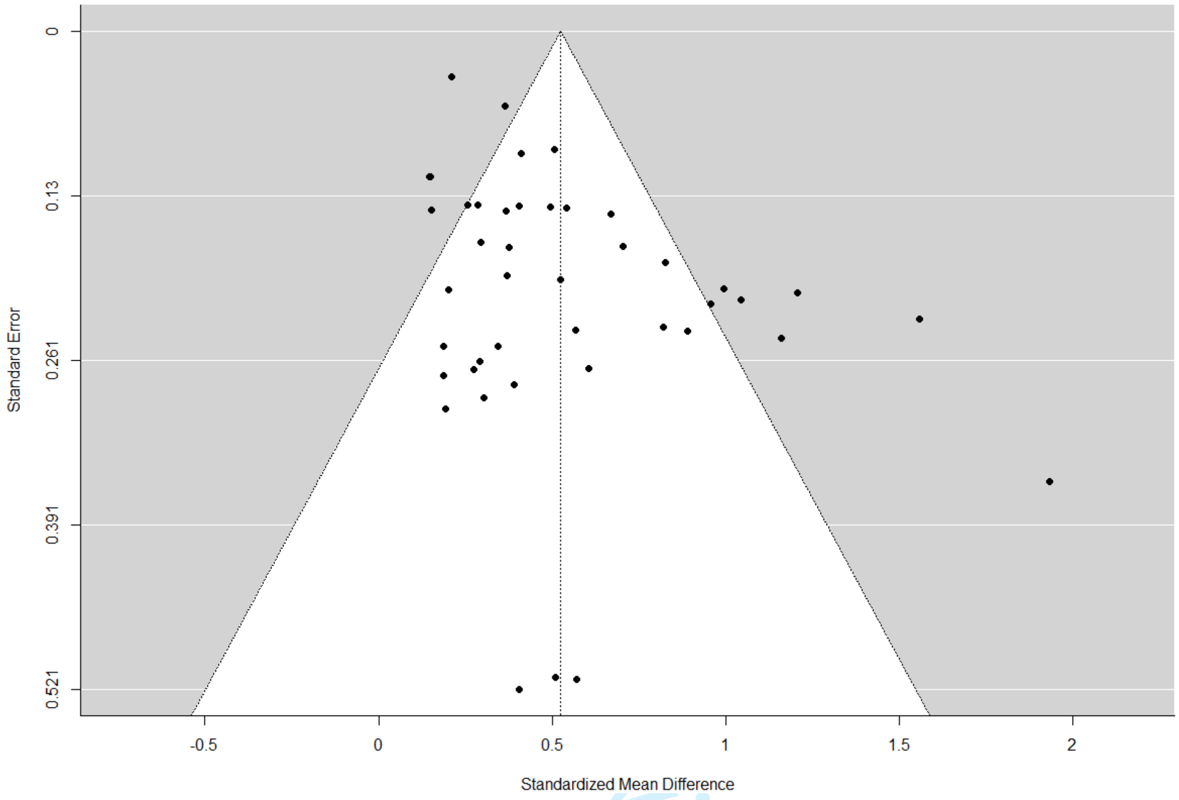

Internet-delivered transdiagnostic psychological treatments for individuals with depression, anxiety, or both: a systematic review and meta-analysis of randomized controlled trials

Meta-analysis versus treatment-as-usual and waitlist controls, with depression symptoms as outcome, with missing trials imputed based on the trim-and-fill procedure and the R0 estimator

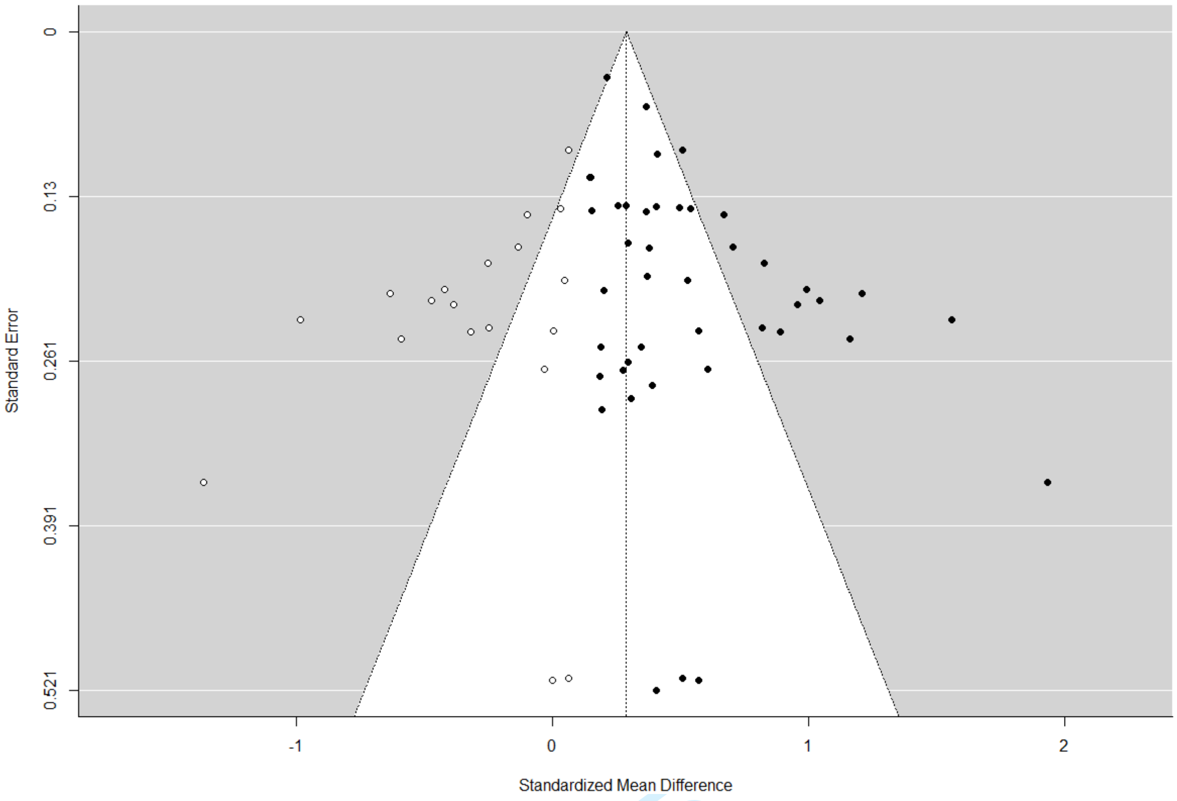

Internet-delivered transdiagnostic psychological treatments for individuals with depression, anxiety, or both: a systematic review and meta-analysis of randomized controlled trials

Meta-analysis versus treatment-as-usual and waitlist controls, with anxiety as outcome

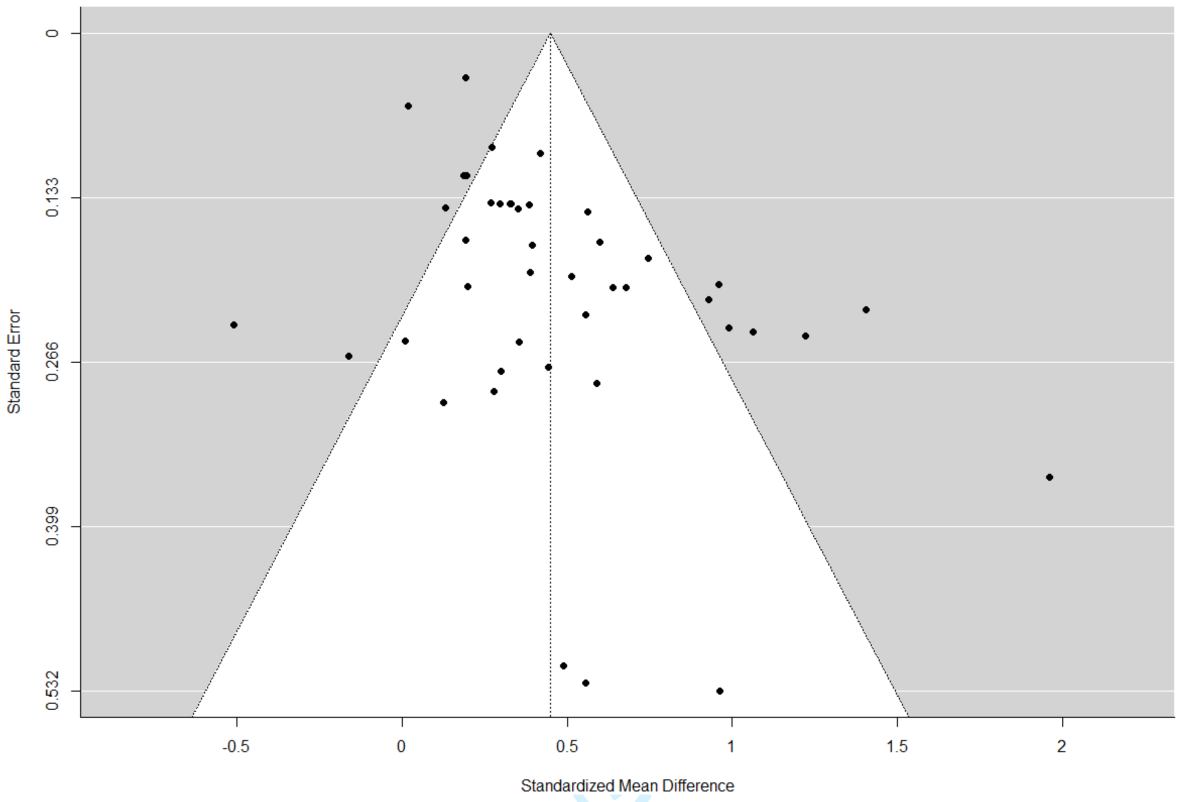

For peer review only - http://bmjopen.bmj.com/site/about/guidelines.xhtml

Internet-delivered transdiagnostic psychological treatments for individuals with depression, anxiety, or both: a systematic review and meta-analysis of randomized controlled trials

Risk of bias re-assessed separately based on whether trials required participants to meet full criteria for formal psychiatric diagnosis or not

Asymmetry of the funnel plot may result from several mechanisms. Because heterogeneity in the main analyses was substantial, and the moderator analyses indicated that the only significant moderator of the between-group effect versus treatment-as-usual and waitlist controls was whether trials required all participants to meet full criteria for a formal diagnosis or not, we repeated our investigation of the funnel plot for these substrata of the trials. That is, we first assessed whether publication bias appeared to be present in trials that employed psychiatric diagnoses, and then assessed whether publication bias appeared to be present in trials that did not employ psychiatric diagnoses. The results are tabulated in the table below. Overall, even when the sample of trials was split along this only significant effect moderator (i.e., psychiatric diagnoses or not), the outcome of the analyses of publication bias was still contradictory and similar to the equivalent tests of all trials.

Table S.B.2. Sensitivity analyses pertaining to publication bias

**Subset of trials**	**Outcome**	**Visual inspection**	**Egger's test**	**Trim-and-fill**
Psychiatric diagnosis (k = 12)	Depression	Possibly slight bias, but not clear	$z = 3.67$, $p < 0.001$	R0: $p = 0.250$, but 1 trial imputed<br>L0: no imputation<br>Q0: no imputation
Psychiatric diagnosis (k = 12)	Anxiety	No clear sign of bias	$z = 2.08$, $p = 0.038$	R0: $p = 0.500$, no imputation<br>L0: no imputation<br>Q0: no imputation
Cut-off or symptoms (k = 31)	Depression	Possibly slight bias, but not clear	$z = 0.82$, $p = 0.410$	R0: $p = 0.004$, and 7 trials imputed<br>L0: no imputation<br>Q0: no imputation
Cut-off or symptoms (k = 31)	Anxiety	Possibly slight bias, but not clear	$z = 2.31$, $p = 0.021$	R0: $p = 0.500$, no imputation<br>L0: 9 trials imputed<br>Q0: 12 trials imputed

For peer review only

Internet-delivered transdiagnostic psychological treatments for individuals with depression, anxiety, or both: a systematic review and meta-analysis of randomized controlled trials

Non-planned sensitivity analyses of psychiatric diagnosis as an effect moderator

One moderator was found to be significant for both depression and anxiety symptoms: trials that required all participants to meet full criteria for a psychiatric diagnosis reported a larger pooled between-group effects versus treatment-as-usual and waitlist controls, as compared to trials that did not have this requirement. We conducted a non-planned sensitivity analysis to ensure that this effect was not driven merely by participants in control groups of the routine clinic being more likely to be enrolled other treatments, by testing the moderating effect of diagnosis on the pooled within-group effect. This test illustrated that psychiatric diagnosis remained a significant effect moderator also with regard to within-group effects (depression: 1.10 vs. 0.75, $p = 0.001$; anxiety: 1.14 vs. 0.63, $p < 0.001$). We also reviewed studies that used the most common depression and anxiety outcome respectively (the PHQ-9 and GAD-7) to determine if the effect of diagnosis was driven primarily by a difference in the mean symptom reduction, or by a smaller standard deviation. Though the average standard deviation was slightly smaller in studies that required participants to meet full criteria for a diagnosis, the reduction in the mean was also larger. In summary, requiring participants to meet full criteria for psychiatric diagnosis appeared to be robust effect moderator.

For peer review only

Internet-delivered transdiagnostic psychological treatments for individuals with depression, anxiety, or both: a systematic review and meta-analysis of randomized controlled trials

Randomized controlled trials versus other bona fide treatments

Two trials compared the effects of an internet-delivered transdiagnostic psychological treatment to those of a distinctly different bona fide treatment. One trial compared mindfulness-based stress reduction delivered via a mobile app (n = 28) to a face-to-face equivalent (n = 20) for university students and reported non-significant between-group effects (depression: g = -0.49; anxiety: g = -0.13).[4] One trial compared web-based CBT (n = 23) to a face-to-face equivalent (n = 21) for university students and reported a significant and large between-group effect on depression in favor of the face-to-face treatment (g = -1.39) and a non-significant and small between-group effect on anxiety (g = -0.21).[42] Last, we also identified one trial that was on the verge of being a bona-fide comparison. In this trial, web-based CBT (n = 33) was compared to a condition which was described as a waitlist, but where participants had access to an unguided CBT bibliotherapy which we deemed to be relatively credible (n = 34). There were no significant between-group effects on depression (g = 0.33) or anxiety (g = -0.13).[54]

For peer review only

Internet-delivered transdiagnostic psychological treatments for individuals with depression, anxiety, or both: a systematic review and meta-analysis of randomized controlled trials

Randomized factorial trials

Nine randomized factorial trials explored moderators of the overall effect of internet-delivered transdiagnostic psychological treatments. Seven of these trials evaluated the same treatment protocol: the Wellbeing Course. The factors investigated, and the main outcome of each of the nine randomized factorial trials is tabulated below:

Table S.B.3. Randomized factorial trials of internet-delivered transdiagnostic psychological treatments suitable for both depression and anxiety

Reference	N	Protocol	Factors	Summary of main outcome
Dear *et al.* (2018)[6]	217	Wellbeing Course	Therapist-guided vs. unguided	Mostly no significant mean differences in the clinical outcome, with small between-group effects on depression and anxiety at post-treatment.
Hadjistavropoulos *et al.* (2017)[13]	182	Wellbeing Course	Weekly support vs. optional support	No significant mean differences in the clinical outcome, with small between-group effects on depression and anxiety at post-treatment. Rates of treatment completion were significantly higher with weekly support.
Hadjistavropoulos *et al.* (2020a)[14]	744	Wellbeing Course	Weekly support by ICBT specialist vs. weekly support plus on-demand support by ICBT specialist vs. weekly support by other clinician	Mostly no significant mean differences in the clinical outcome, with small between-group effects on depression and anxiety at post-treatment. Rates of response in depression were lower with support from other clinicians.
Hadjistavropoulos *et al.* (2020b)[15]	631	Wellbeing Course	Use of homework reflection vs. no use of homework reflection<br><br>Weekly support vs. twice-weekly support<br><br>ICBT clinic vs. community mental health clinic	No significant mean differences in the clinical outcome, with small between-group effects on depression and anxiety at post-treatment.
Hadjistavropoulos *et al.* (2022)[16]	469	Wellbeing Course	Optional 4-week extension of support vs. no such option<br><br>Optional booster lesson vs. no such option	No significant mean differences in the clinical outcome, with small between-group effects on depression and anxiety at post-treatment. About 52% opted to make use of the booster lesson.

For peer review only

Internet-delivered transdiagnostic psychological treatments for individuals with depression, anxiety, or both: a systematic review and meta-analysis of randomized controlled trials

Isbăşoiu *et al.* (2021)[21]	284	Unified protocol	Addition of self-enhancement and emotion regulation components vs. no such components	No significant mean differences in the clinical outcome, with small between-group effects on depression and anxiety at post-treatment.
Mohr *et al.* (2019)[30]	301	IntelliCare	Therapist-guided vs. unguided *<br><br>Weekly app recommendations vs. no such recommendations *	Guidance led to a significantly larger mean effect on anxiety (but not depression). Recommendations led to a significantly larger mean effect on depression (but not anxiety). Between-group effects were small.
Peynenburg *et al.* (2022)[35]	308	Wellbeing Course	Internet-delivered exercises based on motivational interviewing before treatment vs. no such exercises *<br><br>Optional booster lesson vs. no such option	Internet-delivered exercises based on motivational interviewing before treatment led to a significantly larger mean effect on depression and anxiety. Having the option of a booster lesson did not lead to significantly larger mean effects. Between-group effects were small. About 32% opted to make use of the booster lesson.
Soucy *et al.* (2021)[43]	480	Wellbeing Course	Internet-delivered exercises based on motivational interviewing before treatment vs. no such exercises	No significant mean differences in the clinical outcome, with small between-group effects on depression and anxiety at post-treatment.

* This factor was a significant moderator of the effect on depression symptoms or anxiety.

For peer review only

Internet-delivered transdiagnostic psychological treatments for individuals with depression, anxiety, or both: a systematic review and meta-analysis of randomized controlled trials

Brief interventions

Two trials evaluated brief interventions (≤2 weeks) for adults. One trial (*N*=70) found that 2 weeks of communication with an automated chatbot trained in active listening and CBT strategies was associated with a small to moderate between-group effect on depression (g = 0.37) but no significant between-group effect on anxiety as compared to a waitlist.[9] The second trial (*N*=45) reported null effects of a single session of cognitive bias modification on depression (g = -0.04) and anxiety (g = 0.42), compared to a psychological placebo.[39]

For peer review only

Internet-delivered transdiagnostic psychological treatments for individuals with depression, anxiety, or both: a systematic review and meta-analysis of randomized controlled trials

References

1. Al-Refae M, Al-Refae A, Munroe M, et al. A Self-Compassion and Mindfulness-Based Cognitive Mobile Intervention (Serene) for Depression, Anxiety, and Stress: Promoting Adaptive Emotional Regulation and Wisdom. *Front Psychol* 2021;12:648087. doi: 10.3389/fpsyg.2021.648087 [published Online First: 2021/04/09]
2. Batterham PJ, Calear AL, Farrer L, et al. Efficacy of a Transdiagnostic Self-Help Internet Intervention for Reducing Depression, Anxiety, and Suicidal Ideation in Adults: Randomized Controlled Trial. *J Med Internet Res* 2021;23(1):e22698. doi: 10.2196/22698 [published Online First: 2021/01/23]
3. Beshai S, Bueno C, Yu M, et al. Examining the effectiveness of an online program to cultivate mindfulness and self-compassion skills (Mind-OP): Randomized controlled trial on Amazon's Mechanical Turk. *Behav Res Ther* 2020;134:103724. doi: 10.1016/j.brat.2020.103724 [published Online First: 2020/09/18]
4. Borjalilu S, Mazaheri MA, Talebpour A. Effectiveness of Mindfulness-Based Stress Management in The Mental Health of Iranian University Students: A Comparison of Blended Therapy, Face-to-Face Sessions, and mHealth App (Aramgar). *Iranian Journal of Psychiatry and Behavioral Sciences* 2019;13(2) doi: 10.5812/ijpbs.84726
5. Carolan S, Harris PR, Greenwood K, et al. Increasing engagement with an occupational digital stress management program through the use of an online facilitated discussion group: Results of a pilot randomised controlled trial. *Internet Interv* 2017;10:1-11. doi: 10.1016/j.invent.2017.08.001 [published Online First: 2018/08/24]
6. Dear BF, Fogliati VJ, Fogliati R, et al. Treating anxiety and depression in young adults: A randomised controlled trial comparing clinician-guided versus self-guided Internet-delivered cognitive behavioural therapy. *Aust N Z J Psychiatry* 2018;52(7):668-79. doi: 10.1177/0004867417738055 [published Online First: 2017/10/25]
7. Diaz-Garcia A, Gonzalez-Robles A, Garcia-Palacios A, et al. Negative and Positive Affect Regulation in a Transdiagnostic Internet-Based Protocol for Emotional Disorders: Randomized Controlled Trial. *J Med Internet Res* 2021;23(2):e21335. doi: 10.2196/21335 [published Online First: 2021/02/02]
8. Farrer LM, Gulliver A, Katruss N, et al. A novel multi-component online intervention to improve the mental health of university students: Randomised controlled trial of the Uni Virtual Clinic. *Internet Interv* 2019;18:100276. doi: 10.1016/j.invent.2019.100276 [published Online First: 2020/01/01]
9. Fitzpatrick KK, Darcy A, Vierhile M. Delivering Cognitive Behavior Therapy to Young Adults With Symptoms of Depression and Anxiety Using a Fully Automated Conversational Agent (Woebot): A Randomized Controlled Trial. *JMIR Ment Health* 2017;4(2):e19. doi: 10.2196/mental.7785 [published Online First: 2017/06/08]
10. Gonzalez-Robles A, Diaz-Garcia A, Garcia-Palacios A, et al. Effectiveness of a Transdiagnostic Guided Internet-Delivered Protocol for Emotional Disorders Versus Treatment as Usual in Specialized Care: Randomized Controlled Trial. *J Med Internet Res* 2020;22(7):e18220. doi: 10.2196/18220 [published Online First: 2020/07/17]
11. Graham AK, Greene CJ, Kwasny MJ, et al. Coached Mobile App Platform for the Treatment of Depression and Anxiety Among Primary Care Patients: A Randomized Clinical Trial. *JAMA Psychiatry* 2020;77(9):906-14. doi: 10.1001/jamapsychiatry.2020.1011 [published Online First: 2020/05/21]
12. Gregoire S, Beaulieu F, Lachance L, et al. An online peer support program to improve mental health among university students: A randomized controlled trial. *J Am Coll*

For peer review only

Internet-delivered transdiagnostic psychological treatments for individuals with depression, anxiety, or both: a systematic review and meta-analysis of randomized controlled trials

Health 2022:1-13. doi: 10.1080/07448481.2022.2099224 [published Online First: 2022/08/10]

13. Hadjistavropoulos HD, Schneider LH, Edmonds M, et al. Randomized controlled trial of internet-delivered cognitive behaviour therapy comparing standard weekly versus optional weekly therapist support. *J Anxiety Disord* 2017;52:15-24. doi: 10.1016/j.janxdis.2017.09.006 [published Online First: 2017/10/02]
14. Hadjistavropoulos HD, Peynenburg V, Nugent M, et al. Transdiagnostic Internet-delivered cognitive behaviour therapy with therapist support offered once-weekly or once-weekly supplemented with therapist support within one-business-day: Pragmatic randomized controlled trial. *Internet Interv* 2020;22:100347. doi: 10.1016/j.invent.2020.100347 [published Online First: 20200831]
15. Hadjistavropoulos HD, Peynenburg V, Thiessen DL, et al. A pragmatic factorial randomized controlled trial of transdiagnostic internet-delivered cognitive behavioural therapy: Exploring benefits of homework reflection questionnaires and twice-weekly therapist support. *Internet Interv* 2020;22:100357. doi: 10.1016/j.invent.2020.100357 [published Online First: 20201201]
16. Hadjistavropoulos HD, Peynenburg V, Thiessen DL, et al. A randomized factorial trial of internet-delivered cognitive behavioural therapy: An 8-week program with or without extended support and booster lesson. *Internet Interv* 2022;27:100499. doi: 10.1016/j.invent.2022.100499 [published Online First: 20220206]
17. Hensel JM, Shaw J, Ivers NM, et al. A Web-Based Mental Health Platform for Individuals Seeking Specialized Mental Health Care Services: Multicenter Pragmatic Randomized Controlled Trial. *J Med Internet Res* 2019;21(6):e10838. doi: 10.2196/10838 [published Online First: 2019/06/06]
18. Hirsch CR, Krahe C, Whyte J, et al. Effects of modifying interpretation bias on transdiagnostic repetitive negative thinking. *J Consult Clin Psychol* 2020;88(3):226-39. doi: 10.1037/ccp0000455 [published Online First: 2020/02/19]
19. Hoek W, Schuurmans J, Koot HM, et al. Effects of Internet-based guided self-help problem-solving therapy for adolescents with depression and anxiety: a randomized controlled trial. *PLoS One* 2012;7(8):e43485. doi: 10.1371/journal.pone.0043485 [published Online First: 2012/09/07]
20. Hosseinzadeh Asl NR. A randomized controlled trial of a mindfulness-based intervention in social workers working during the COVID-19 crisis. *Curr Psychol* 2022;41(11):8192-99. doi: 10.1007/s12144-021-02150-3 [published Online First: 2021/08/17]
21. Isbăşoiu AB, Tulbure BT, Rusu A, et al. Can We Boost Treatment Adherence to an Online Transdiagnostic Intervention by Adding Self-Enhancement Strategies? Results From a Randomized Controlled Non-inferiority Trial. *Frontiers in psychology* 2021;12:752249. doi: 10.3389/fpsyg.2021.752249 [published Online First: 20211202]
22. Johansson R, Bjorklund M, Hornborg C, et al. Affect-focused psychodynamic psychotherapy for depression and anxiety through the Internet: a randomized controlled trial. *PeerJ* 2013;1:e102. doi: 10.7717/peerj.102 [published Online First: 2013/07/19]
23. Kang MY, Nan JKM, Yuan Y. Effectiveness of an online short-term audio-based mindfulness program on negative emotions during the COVID-19 pandemic: Latent growth curve analyses of anxiety and moderated mediation effects of anxiety between mindfulness and negative affect. *Curr Psychol* 2022:1-13. doi: 10.1007/s12144-022-03902-5 [published Online First: 2022/12/06]

Internet-delivered transdiagnostic psychological treatments for individuals with depression, anxiety, or both: a systematic review and meta-analysis of randomized controlled trials

24. Kladnitski N, Smith J, Uppal S, et al. Transdiagnostic internet-delivered CBT and mindfulness-based treatment for depression and anxiety: A randomised controlled trial. *Internet Interv* 2020;20:100310. doi: 10.1016/j.invent.2020.100310 [published Online First: 2020/03/07]
25. Kleiboer A, Donker T, Seekles W, et al. A randomized controlled trial on the role of support in Internet-based problem solving therapy for depression and anxiety. *Behav Res Ther* 2015;72:63-71. doi: 10.1016/j.brat.2015.06.013 [published Online First: 2015/07/21]
26. Krafft J, Potts S, Schoendorff B, et al. A Randomized Controlled Trial of Multiple Versions of an Acceptance and Commitment Therapy Matrix App for Well-Being. *Behav Modif* 2019;43(2):246-72. doi: 10.1177/0145445517748561 [published Online First: 2017/12/22]
27. Levin ME, Hicks ET, Krafft J. Pilot evaluation of the stop, breathe & think mindfulness app for student clients on a college counseling center waitlist. *J Am Coll Health* 2022;70(1):165-73. doi: 10.1080/07448481.2020.1728281 [published Online First: 2020/03/10]
28. Lindegaard T, Seaton F, Halaj A, et al. Internet-based cognitive behavioural therapy for depression and anxiety among Arabic-speaking individuals in Sweden: a pilot randomized controlled trial. *Cogn Behav Ther* 2021;50(1):47-66. doi: 10.1080/16506073.2020.1771414 [published Online First: 2020/07/01]
29. McCloud T, Jones R, Lewis G, et al. Effectiveness of a Mobile App Intervention for Anxiety and Depression Symptoms in University Students: Randomized Controlled Trial. *JMIR Mhealth Uhealth* 2020;8(7):e15418. doi: 10.2196/15418 [published Online First: 2020/08/01]
30. Mohr DC, Schueller SM, Tomasino KN, et al. Comparison of the Effects of Coaching and Receipt of App Recommendations on Depression, Anxiety, and Engagement in the IntelliCare Platform: Factorial Randomized Controlled Trial. *Journal of medical Internet research* 2019;21(8):e13609. doi: 10.2196/13609 [published Online First: 20190828]
31. Mullin A, Dear BF, Karin E, et al. The UniWellbeing course: A randomised controlled trial of a transdiagnostic internet-delivered cognitive behavioural therapy (CBT) programme for university students with symptoms of anxiety and depression. *Internet Interventions* 2015;2(2):128-36. doi: 10.1016/j.invent.2015.02.002
32. Newby JM, Mackenzie A, Williams AD, et al. Internet cognitive behavioural therapy for mixed anxiety and depression: a randomized controlled trial and evidence of effectiveness in primary care. *Psychol Med* 2013;43(12):2635. doi: 10.1017/S0033291713000111
33. Nilsson A, Sorman K, Klingvall J, et al. MyCompass in a Swedish context - lessons learned from the transfer of a self-guided intervention targeting mental health problems. *BMC Psychiatry* 2019;19(1):51. doi: 10.1186/s12888-019-2039-1 [published Online First: 2019/02/02]
34. Osborn TL, Rodriguez M, Wasil AR, et al. Single-session digital intervention for adolescent depression, anxiety, and well-being: Outcomes of a randomized controlled trial with Kenyan adolescents. *J Consult Clin Psychol* 2020;88(7):657-68. doi: 10.1037/ccp0000505 [published Online First: 2020/05/12]
35. Peynenburg V, Hadjistavropoulos H, Thiessen D, et al. Internet-Delivered Cognitive Behavioral Therapy for Postsecondary Students: Randomized Factorial Trial for Examining Motivational Interviewing and Booster Lessons. *Journal of medical*

For peer review only

Internet research 2022;24(9):e40637. doi: 10.2196/40637 [published Online First: 20220907]

36. Powell J, Hamborg T, Stallard N, et al. Effectiveness of a web-based cognitive-behavioral tool to improve mental well-being in the general population: randomized controlled trial. *J Med Internet Res* 2012;15(1):e2. doi: 10.2196/jmir.2240 [published Online First: 2013/01/11]
37. Proudfoot J, Clarke J, Birch MR, et al. Impact of a mobile phone and web program on symptom and functional outcomes for people with mild-to-moderate depression, anxiety and stress: a randomised controlled trial. *BMC Psychiatry* 2013;13:312. doi: 10.1186/1471-244X-13-312 [published Online First: 2013/11/19]
38. Robichaud M, Talbot F, Titov N, et al. Facilitating access to iCBT: a randomized controlled trial assessing a translated version of an empirically validated program using a minimally monitored delivery model. *Behav Cogn Psychother* 2020;48(2):185-202. doi: 10.1017/S135246581900047X [published Online First: 2019/08/17]
39. Rogers J, Sicouri G. A Single-Session Online Cognitive Bias Modification of Interpretations Modified for Adults With Anxiety and Depressive Symptoms. *Behav Ther* 2022;53(5):967-80. doi: 10.1016/j.beth.2022.04.006 [published Online First: 2022/08/21]
40. Rollman BL, Herbeck Belnap B, Abebe KZ, et al. Effectiveness of Online Collaborative Care for Treating Mood and Anxiety Disorders in Primary Care: A Randomized Clinical Trial. *JAMA Psychiatry* 2018;75(1):56-64. doi: 10.1001/jamapsychiatry.2017.3379 [published Online First: 2017/11/09]
41. Schaeuffele C, Homeyer S, Perea L, et al. The unified protocol as an internet-based intervention for emotional disorders: Randomized controlled trial. *PLoS One* 2022;17(7):e0270178. doi: 10.1371/journal.pone.0270178 [published Online First: 2022/07/12]
42. Sethi S. Treating Youth Depression and Anxiety: A Randomised Controlled Trial Examining the Efficacy of Computerised versus Face-to-face Cognitive Behaviour Therapy. *Australian Psychologist* 2013;48(4):249-57. doi: 10.1111/ap.12006
43. Soucy JN, Hadjistavropoulos HD, Karin E, et al. Brief online motivational interviewing pre-treatment intervention for enhancing internet-delivered cognitive behaviour therapy: A randomized controlled trial. *Internet Interv* 2021;25:100394. doi: 10.1016/j.invent.2021.100394 [published Online First: 20210420]
44. Southwell S, Gould E. A randomised wait list-controlled pre–post–follow-up trial of a gratitude diary with a distressed sample. *The Journal of Positive Psychology* 2016;12(6):579-93. doi: 10.1080/17439760.2016.1221127
45. Taylor H, Cavanagh K, Field AP, et al. Health Care Workers' Need for Headspace: Findings From a Multisite Definitive Randomized Controlled Trial of an Unguided Digital Mindfulness-Based Self-help App to Reduce Healthcare Worker Stress. *JMIR Mhealth Uhealth* 2022;10(8):e31744. doi: 10.2196/31744 [published Online First: 2022/08/26]
46. Terides MD, Dear BF, Fogliati VJ, et al. Increased skills usage statistically mediates symptom reduction in self-guided internet-delivered cognitive-behavioural therapy for depression and anxiety: a randomised controlled trial. *Cogn Behav Ther* 2018;47(1):43-61. doi: 10.1080/16506073.2017.1347195 [published Online First: 2017/07/21]

For peer review only

Internet-delivered transdiagnostic psychological treatments for individuals with depression, anxiety, or both: a systematic review and meta-analysis of randomized controlled trials

47. Titov N, Dear BF, Schwencke G, et al. Transdiagnostic internet treatment for anxiety and depression: a randomised controlled trial. *Behav Res Ther* 2011;49(8):441-52. doi: 10.1016/j.brat.2011.03.007 [published Online First: 2011/06/18]
48. Titov N, Dear BF, Johnston L, et al. Improving adherence and clinical outcomes in self-guided internet treatment for anxiety and depression: randomised controlled trial. *PLoS One* 2013;8(7):e62873. doi: 10.1371/journal.pone.0062873 [published Online First: 2013/07/12]
49. Titov N, Fogliati VJ, Staples LG, et al. Treating anxiety and depression in older adults: randomised controlled trial comparing guided v. self-guided internet-delivered cognitive-behavioural therapy. *BJPsych Open* 2016;2(1):50-58. doi: 10.1192/bjpo.bp.115.002139 [published Online First: 2016/10/06]
50. Tönnies J, Hartmann M, Wensing M, et al. Mental Health Specialist Video Consultations Versus Treatment-as-Usual for Patients With Depression or Anxiety Disorders in Primary Care: Randomized Controlled Feasibility Trial. *JMIR Ment Health* 2021;8(3):e22569. doi: 10.2196/22569 [published Online First: 2021/03/13]
51. Tulbure BT, Rusu A, Sava FA, et al. A Web-Based Transdiagnostic Intervention for Affective and Mood Disorders: Randomized Controlled Trial. *JMIR Ment Health* 2018;5(2):e36. doi: 10.2196/mental.8901 [published Online First: 2018/05/26]
52. Twomey C, O'Reilly G, Byrne M, et al. A randomized controlled trial of the computerized CBT programme, MoodGYM, for public mental health service users waiting for interventions. *Br J Clin Psychol* 2014;53(4):433-50. doi: 10.1111/bjc.12055 [published Online First: 2014/05/17]
53. van Straten A, Cuijpers P, Smits N. Effectiveness of a web-based self-help intervention for symptoms of depression, anxiety, and stress: randomized controlled trial. *J Med Internet Res* 2008;10(1):e7. doi: 10.2196/jmir.954 [published Online First: 2008/03/28]
54. Villemaire-Krajden R, Myhr G. Evaluating the Use of a Computerized CBT Program for Outpatients on a Waitlist in a University CBT Unit. *J Psychiatr Pract* 2019;25(4):268-78. doi: 10.1097/PRA.0000000000000396 [published Online First: 2019/07/11]
55. Viravan N, Atsariyasing W, Srifuengfung M, et al. Efficacy of Online Mindfulness Program ‘Mindful Senses’ for Depression and Anxiety Reduction in Community Samples: A Randomized Controlled Trial. *J Med Assoc Thai* 2022;105(11):1108-19. doi: 10.35755/jmedassocthai.2022.11.13700
56. Viskovich S, Pakenham KI. Randomized controlled trial of a web-based Acceptance and Commitment Therapy (ACT) program to promote mental health in university students. *J Clin Psychol* 2020;76(6):929-51. doi: 10.1002/jclp.22848 [published Online First: 2019/08/31]
57. Zetterqvist K, Maanmies J, Strom L, et al. Randomized controlled trial of internet-based stress management. *Cogn Behav Ther* 2003;32(3):151-60. doi: 10.1080/16506070302316 [published Online First: 2005/11/18]
58. Shi L, Lin L. The trim-and-fill method for publication bias: practical guidelines and recommendations based on a large database of meta-analyses. *Medicine* 2019;98(23)

PRISMA 2020 Checklist

Section and Topic	Item #	Checklist item	Location where item is reported
**TITLE**			Page:
Title	1	Identify the report as a systematic review.	1
**ABSTRACT**			
Abstract	2	See the PRISMA 2020 for Abstracts checklist.	3
**INTRODUCTION**			
Rationale	3	Describe the rationale for the review in the context of existing knowledge.	4
Objectives	4	Provide an explicit statement of the objective(s) or question(s) the review addresses.	5
**METHODS**			
Eligibility criteria	5	Specify the inclusion and exclusion criteria for the review and how studies were grouped for the syntheses.	6,7,8
Information sources	6	Specify all databases, registers, websites, organisations, reference lists and other sources searched or consulted to identify studies. Specify the date when each source was last searched or consulted.	5
Search strategy	7	Present the full search strategies for all databases, registers and websites, including any filters and limits used.	5 + Supplement A
Selection process	8	Specify the methods used to decide whether a study met the inclusion criteria of the review, including how many reviewers screened each record and each report retrieved, whether they worked independently, and if applicable, details of automation tools used in the process.	6
Data collection process	9	Specify the methods used to collect data from reports, including how many reviewers collected data from each report, whether they worked independently, any processes for obtaining or confirming data from study investigators, and if applicable, details of automation tools used in the process.	8,9
Data items	10a	List and define all outcomes for which data were sought. Specify whether all results that were compatible with each outcome domain in each study were sought (e.g. for all measures, time points, analyses), and if not, the methods used to decide which results to collect.	8,9
	10b	List and define all other variables for which data were sought (e.g. participant and intervention characteristics, funding sources). Describe any assumptions made about any missing or unclear information.	8,9
Study risk of bias assessment	11	Specify the methods used to assess risk of bias in the included studies, including details of the tool(s) used, how many reviewers assessed each study and whether they worked independently, and if applicable, details of automation tools used in the process.	10
Effect measures	12	Specify for each outcome the effect measure(s) (e.g. risk ratio, mean difference) used in the synthesis or presentation of results.	10, 11
Synthesis methods	13a	Describe the processes used to decide which studies were eligible for each synthesis (e.g. tabulating the study intervention characteristics and comparing against the planned groups for each synthesis (item #5)).	9 - 11
	13b	Describe any methods required to prepare the data for presentation or synthesis, such as handling of missing summary statistics, or data conversions.	10, 11
	13c	Describe any methods used to tabulate or visually display results of individual studies and syntheses.	10, 11
	13d	Describe any methods used to synthesize results and provide a rationale for the choice(s). If meta-analysis was performed, describe the model(s), method(s) to identify the presence and extent of statistical heterogeneity, and software package(s) used.	10-11
	13e	Describe any methods used to explore possible causes of heterogeneity among study results (e.g. subgroup analysis, meta-regression).	11
	13f	Describe any sensitivity analyses conducted to assess robustness of the synthesized results.	11
Reporting bias assessment	14	Describe any methods used to assess risk of bias due to missing results in a synthesis (arising from reporting biases).	11

For peer review only

For peer review only - http://bmjopen.bmj.com/site/about/guidelines.xhtml

PRISMA 2020 Checklist

Section and Topic	Item #	Checklist item	Location where item is reported
Certainty assessment	15	Describe any methods used to assess certainty (or confidence) in the body of evidence for an outcome.	11
**RESULTS**			
Study selection	16a	Describe the results of the search and selection process, from the number of records identified in the search to the number of studies included in the review, ideally using a flow diagram.	Fig 1
	16b	Cite studies that might appear to meet the inclusion criteria, but which were excluded, and explain why they were excluded.	11
Study characteristics	17	Cite each included study and present its characteristics.	
Risk of bias in studies	18	Present assessments of risk of bias for each included study.	11 + Fig 2
Results of individual studies	19	For all outcomes, present, for each study: (a) summary statistics for each group (where appropriate) and (b) an effect estimate and its precision (e.g. confidence/credible interval), ideally using structured tables or plots.	12, Fig 3, Fig 4
Results of syntheses	20a	For each synthesis, briefly summarise the characteristics and risk of bias among contributing studies.	13
	20b	Present results of all statistical syntheses conducted. If meta-analysis was done, present for each the summary estimate and its precision (e.g. confidence/credible interval) and measures of statistical heterogeneity. If comparing groups, describe the direction of the effect.	13
	20c	Present results of all investigations of possible causes of heterogeneity among study results.	13
	20d	Present results of all sensitivity analyses conducted to assess the robustness of the synthesized results.	14
Reporting biases	21	Present assessments of risk of bias due to missing results (arising from reporting biases) for each synthesis assessed.	14, Supplement 2
Certainty of evidence	22	Present assessments of certainty (or confidence) in the body of evidence for each outcome assessed.	13, 14, Figure 3, Figure 4,
**DISCUSSION**			
Discussion	23a	Provide a general interpretation of the results in the context of other evidence.	16, 17, 18
	23b	Discuss any limitations of the evidence included in the review.	19
	23c	Discuss any limitations of the review processes used.	19
	23d	Discuss implications of the results for practice, policy, and future research.	19
**OTHER INFORMATION**			
Registration and protocol	24a	Provide registration information for the review, including register name and registration number, or state that the review was not registered.	5
	24b	Indicate where the review protocol can be accessed, or state that a protocol was not prepared.	5
	24c	Describe and explain any amendments to information provided at registration or in the protocol.	10
Support	25	Describe sources of financial or non-financial support for the review, and the role of the funders or sponsors in the review.	21
Competing interests	26	Declare any competing interests of review authors.	3
Availability of	27	Report which of the following are publicly available and where they can be found: template data collection forms; data extracted from included	21

For peer review only

PRISMA 2020 Checklist

Section and Topic	Item #	Checklist item	Location where item is reported
data, code and other materials		studies; data used for all analyses; analytic code; any other materials used in the review.	

From: Page MJ, McKenzie JE, Bossuyt PM, Boutron I, Hoffmann TC, Mulrow CD, et al. The PRISMA 2020 statement: an updated guideline for reporting systematic reviews. BMJ 2021;372:n71. doi: 10.1136/bmj.n71

For more information, visit: http://www.prisma-statement.org/

For peer review only

BMJ Open

Internet-delivered transdiagnostic psychological treatments for individuals with depression, anxiety, or both: a systematic review with meta-analysis of randomized controlled trials

Journal:	*BMJ Open*
Manuscript ID	bmjopen-2023-075796.R1
Article Type:	Original research
Date Submitted by the Author:	12-Dec-2023

Complete List of Authors:	Kolaas, Karoline; Karolinska Institute Department of Clinical Neuroscience, Centre for Psychiatry Research; Academic Primary Care Centre, Liljeholmen University Primary Health care Center, Region Stockholm<br>Berman, Anne H.; Uppsala Universitet, Department of Psychology; Karolinska Institute Department of Clinical Neuroscience, Centre for Psychiatry Research<br>Hedman-Lagerlöf, Erik ; Karolinska Institute Department of Clinical Neuroscience, Division of Psychology; Academic Primary Care Centre, Gustavsberg University Primary Health Care Center, Region Stockholm<br>Lindsäter, Elin; Karolinska Institute Department of Clinical Neuroscience, Department of Clinical Neuroscience; Academic Primary Care Centre, Gustavsberg University Primary Health Care Center, Region Stockholm<br>Hybelius, Jonna; Karolinska Institute, Division of Family Medicine and Primary Care, Department of Neurobiology, Care Sciences and Society; Academic Primary Care Centre, Liljeholmen University Primary Health Care Center, Region Stockholm<br>Axelsson, Erland; Karolinska Institutet, Division of Family Medicine and Primary Care, Department of Neurobiology, Care Sciences and Society; Academic Primary Care Centre, Liljeholmen University Primary Health Care Center, Region Stockholm

**Primary Subject Heading**:	Mental health
Secondary Subject Heading:	General practice / Family practice, Evidence based practice, Mental health
Keywords:	MENTAL HEALTH, PSYCHIATRY, Depression & mood disorders < PSYCHIATRY, Anxiety disorders < PSYCHIATRY, Patient Reported Outcome Measures, Systematic Review

SCHOLARONE™
Manuscripts

For peer review only - http://bmjopen.bmj.com/site/about/guidelines.xhtml

I, the Submitting Author has the right to grant and does grant on behalf of all authors of the Work (as defined in the below author licence), an exclusive licence and/or a non-exclusive licence for contributions from authors who are: i) UK Crown employees; ii) where BMJ has agreed a CC-BY licence shall apply, and/or iii) in accordance with the terms applicable for US Federal Government officers or employees acting as part of their official duties; on a worldwide, perpetual, irrevocable, royalty-free basis to BMJ Publishing Group Ltd ("BMJ") its licensees and where the relevant Journal is co-owned by BMJ to the co-owners of the Journal, to publish the Work in this journal and any other BMJ products and to exploit all rights, as set out in our licence.

The Submitting Author accepts and understands that any supply made under these terms is made by BMJ to the Submitting Author unless you are acting as an employee on behalf of your employer or a postgraduate student of an affiliated institution which is paying any applicable article publishing charge ("APC") for Open Access articles. Where the Submitting Author wishes to make the Work available on an Open Access basis (and intends to pay the relevant APC), the terms of reuse of such Open Access shall be governed by a Creative Commons licence – details of these licences and which Creative Commons licence will apply to this Work are set out in our licence referred to above.

Other than as permitted in any relevant BMJ Author's Self Archiving Policies, I confirm this Work has not been accepted for publication elsewhere, is not being considered for publication elsewhere and does not duplicate material already published. I confirm all authors consent to publication of this Work and authorise the granting of this licence.

For peer review only

Internet-delivered transdiagnostic psychological treatments for individuals with depression, anxiety, or both: a systematic review with meta-analysis of randomized controlled trials

Karoline Kolaas, PhD[a b]; Anne H Berman, PhD[a c]; Erik Hedman-Lagerlöf, PhD[d e]; Elin Lindsäter, PhD[a d e]; Jonna Hybelius, MSc[b f]; and Erland Axelsson, PhD[b f]

Author Affiliations:[a] Centre for Psychiatry Research, Department of Clinical Neuroscience, Karolinska Institutet & Stockholm Health Care Services, Region Stockholm, Stockholm, Sweden (Dr Kolaas, Dr Berman, and Dr Lindsäter),[b] Liljeholmen University Primary Health Care Center, Academic Primary Health Care Center, Region Stockholm, Stockholm, Sweden (Dr Kolaas, Ms Hybelius, and Dr Axelsson),[c] Division of Clinical Psychology, Department of Psychology, Uppsala University, Uppsala, Sweden (Dr Berman),[d] Division of Psychology, Department of Clinical Neuroscience, Karolinska Institutet, Stockholm, Sweden (Dr Hedman-Lagerlöf),[e] Gustavsberg University Primary Health Care Center, Academic Primary Health Care Center, Region Stockholm, Gustavsberg, Sweden (Dr Hedman-Lagerlöf and Dr Lindsäter), and [f] Division of Family Medicine and Primary Care, Department of Neurobiology, Care Sciences and Society, Karolinska Institutet, Stockholm, Sweden (Ms Hybelius and Dr Axelsson).

Corresponding Author: Karoline Kolaas, PhD, Centre for Psychiatry Research, Department of Clinical Neuroscience, Karolinska Institutet, Norra Stationsgatan 69, SE-113 64, Stockholm, Sweden,

Tel: +46 73 655 69 91

For peer review only

E-mail: karoline.kolaas@ki.se

Word count (abstract): 296; (main text): 4938; Tables: 1; Figures: 4

Page count (main text): 21; Line count (main text): 441

For peer review only

For peer review only - http://bmjopen.bmj.com/site/about/guidelines.xhtml

Abstract

Objective: Depression and anxiety are major public health problems. This study evaluated the effects of internet-delivered transdiagnostic psychological treatments for individuals with depression, anxiety, or both.

Design: Systematic review with meta-analysis.

Data sources: Medline (Ovid), Cochrane Library (Wiley), the Web of Science Core Collection (Clarivate), and PsycInfo (EBSCO).

Eligibility criteria: Randomized controlled trials of internet-delivered transdiagnostic psychological treatments, open to both participants with primary depression and participants with primary anxiety. This review concerned all treatment frameworks, both guided and unguided formats, and all age groups.

Data extraction and synthesis: In random effects meta-analysis, we estimated pooled effects on depression symptoms and anxiety in terms of Hedges' *g* with 95% CIs. Absolute and relative heterogeneity was quantified as the τ^2 and I^2.

Results: We included 57 trials with 21795 participants. Nine trials (16%) recruited exclusively from routine care, and three (5%) delivered treatment via video. For adults, large within-group reductions were seen in depression (g = 0.90; 95% CI 0.81-0.99) and anxiety (g = 0.87; 95% CI 0.78-0.96). Compared to rudimentary passive controls, the added effects were moderate (depression: g = 0.52; 95% CI 0.42-0.63; anxiety: g = 0.45; 95% CI 0.34-0.56), and larger in trials that required all participants to meet full diagnostic criteria for depression or

For peer review only - http://bmjopen.bmj.com/site/about/guidelines.xhtml

an anxiety disorder. Compared to attention/engagement controls, the added effects were small (depression: g = 0.30; 95% CI 0.07-0.53; anxiety: g = 0.21; 95% CI 0.01-0.42). Heterogeneity was substantial, and the certainty of the evidence was very low. Two trials concerned adolescents and reported mixed results. One trial concerned older adults and reported promising results.

Conclusion: Internet-delivered transdiagnostic treatments for depression and anxiety show small to moderate added effects, varying by control condition. Research is needed regarding routine care, the video format, children and adolescents, and older adults.

PROSPERO registration number: CRD42021243172.

<Strengths and limitations of this study>

- The systematic search strategy and the broad eligibility criteria allowed for a comprehensive overview of treatment frameworks, formats, age groups, and settings including primary health care.
- Several trial, participant, and treatment characteristics were evaluated as potential moderators of the between-group effect versus rudimentary passive controls.
- The majority of trials recruited self-referred participants through advertisement and were not conducted in clinical settings. This makes it more difficult to draw conclusions about effectiveness in routine care.
- Our analyses of moderators were based exclusively on trial-level characteristics, and should preferably be replicated based on individual patient data.

For peer review only - http://bmjopen.bmj.com/site/about/guidelines.xhtml

<Introduction>

Depression and the common anxiety disorders are associated with immense worldwide healthcare costs and disease burden.(1 2) Psychological treatments for depression and anxiety can be effective(3) including in clinical settings such as primary care(4 5) where the majority of patients are found.(6) However, many patients with depression and anxiety disorders remain untreated or receive inadequate treatment,(7 8) illustrating the need for further development and dissemination of effective therapies.

It is widely recognized that depression and anxiety disorders often occur together,(9 10) and overlap in terms of genetic, cognitive, and behavioral characteristics.(11-13) This speaks for the use of broader, transdiagnostic, psychological treatments designed to suit patients suffering from a range of different types of depression and anxiety problems. That is, transdiagnostic treatments are characterized by focusing on components relevant for both depression and anxiety, in contrast to diagnosis-specific treatments targeting one specific mental health conditon. In addition to beneficial effects for the patient, transdiagnostic treatments can also have organizational advantages, such as streamlined procedures and less time needed for diagnostic assessment.(14). In early meta-analysis, transdiagnostic psychological treatments have been found to have moderate pooled effects on depression and anxiety compared to mostly rudumentary treatment-as-usual and waitlist control groups.(15)

Another method of improving access to psychological treatments can be to deliver these online – for example via websites, mobile apps, or videoconferencing – which saves time and costs for both clinicians and patients. For internet-delivered transdiagnostic psychological treatments, large within-group symptom reductions have been observed, with early meta-analyses reporting mixed outcomes compared to a heterogeneous range of control groups,

For peer review only - http://bmjopen.bmj.com/site/about/guidelines.xhtml

For peer review only

and comparisons versus diagnosis-specific treatments typically result in relatively similar effects on depression and general anxiety.(16-18) Internet-delivered therapies appear to be accepted by patients, and have shown promising witin-group effects in pragmatic routine care studies.

Even though important advancements have been made in internet-delivered transdiagnostic psychological treatments for depression and anxiety in the past decades, several fundamental questions remain unanswered in this rapidly growing field. First, it remains unclear whether effects generalize to children, adolescents, and older adults. Second, there has been no systematic comparison of treatment frameworks beyond the cognitive-behavioral therapies. Third, the relative effect of methods of delivery such as via websites, mobile apps, and videoconferencing is yet unclear. Fourth, although acceptability and within-group change has been studied to some extent in the routine care environment(19), based on randomized controlled trials that include a combination of patients with anxiety and depression, it is not known how well the effects of internet-delivered formats translate to routine clinical settings such as primary care.

We conducted a systematic review and meta-analysis of randomized controlled trials of internet-delivered transdiagnostic psychological treatments for depression and anxiety. This systematic review included all treatment frameworks, both guided and unguided formats, and all age groups. Based on previous research, we hypothesized that between-group effects versus rudimentary passive controls would be large, and that the treatment length would be of little importance for the overall effect.

<Method>

For peer review only - http://bmjopen.bmj.com/site/about/guidelines.xhtml

<Search strategy>

This systematic review with meta-analysis adhered to the PRISMA 2020 statement(20) and was registered at PROSPERO (CRD42021243172) on April 16, 2021 and at the Open Science Framework (https://osf.io/dtcey) on May 18, 2021, i.e., before the initial database searches. We searched Medline (Ovid), Cochrane Library (Wiley), the Web of Science Core Collection (Clarivate), and PsycInfo (EBSCO) for randomized controlled trials of internet-delivered transdiagnostic psychological treatments for individuals with depression, anxiety, or both. A search strategy was developed in collaboration with the Karolinska Institutet library service. Candidate search strings were validated against a selection of articles that we were aware of prior to this systematic review, and after discussion within the research group, as well as with an external expert in psychodynamic online therapies. Four types of search terms were combined: terms for treatment (e.g., "psychotherapy"), terms for online communication (e.g., "internet-delivered"), terms for diagnostic focus (e.g., "transdiagnostic", "depression", "anxiety"), and terms for study design (e.g., "RCT"). Only publications published in 1995 or later were considered because there were no RCTs of internet-delivered transdiagnostic psychological treatments before then. Full search documentation for all databases can be found in Supplement A. An initial search was conducted on 24 May 2021, with an update on 6 February 2023 in accordance with Bramer and Bain.(21) We also reviewed the reference lists of previous meta-analyses that we found relevant.(16 17)

<Selection of studies>

We employed a systematic de-duplication algorithm in Endnote X9,(22) after which unique search hits were imported into Rayyan.(23) The publications were then assessed by two independent assessors in two phases. In phase 1, all titles and abstracts were screened for

For peer review only

eligibility by two authors (of KK, AHB, EHL, and EA), and non-relevant publications were excluded. In phase 2, publications that had been included by at least one assessor in phase 1 were reviewed in full text. One assessor (KK) did this sequentially, i.e., from criterion a to h (see below), for all full texts. Each full text was also reviewed by a second assessor (either AHB, EHL, JH, or EA) who suggested a decision of exclusion based on the first exclusion criterion identified, i.e., in any order. Cases of disagreement were discussed in the research group and a final decision was reached either in consensus or, if necessary, by means of voting. If information about the eligibility criteria a-h was missing, authors were not contacted to provide additional information.

<Eligibility criteria>

(a) Studies had to be published in English in a peer-reviewed journal.

(b) Studies had to be randomized controlled trials where the study participant was the unit of randomization. This was because our focus was on conducting meta-analyses, for which cluster randomized trials require distinct analytical methods.(24) Only one publication was formally included for each randomized trial.

(c) Studies were required to evaluate the effect of an internet-delivered transdiagnostic psychological treatment for individuals with anxiety, depression, or both. “Internet-delivered” included treatments delivered via websites, mobile apps, or videoconferencing. Treatments where online components were a mere complement to a face-to-face treatment, or where participants were required to attend a particular physical location, were excluded. “Transdiagnostic” was defined as intending to target depression as well as anxiety. “Psychological treatment” was defined as an intervention anchored in psychological theory with a clinical framing (i) where a clinician or facilitator had an active role in moderating the process, or (ii) which was

For peer review only - http://bmjopen.bmj.com/site/about/guidelines.xhtml

delivered in a structured manner, for example in the form of modules with suggested homework, or with an interactive component such as automatic feedback. For example, a webpage with general information about depression and anxiety would not be considered a psychological treatment, due to the lack of structure and interactivity.

(d) Studies needed to recruit participants suffering from clinically significant depression, anxiety, or both. We considered this criterion to be met if one of the following was true: (i) all participants were required to meet full diagnostic criteria for either primary depression or a primary anxiety disorder and both populations were included in the trial, or (ii) all participants were required to score above a valid cut-off on an accepted screening measure for either depression or anxiety and both populations were included in the trial, or (iii) all participants were required to score above both a valid cut-off for depression and a valid cut-off for anxiety, or (iv) the baseline sample mean of at least one transdiagnostic treatment arm and at least one other arm were above both a valid cut-off for depression and a valid cut-off for anxiety. We excluded RCTs that either required all participants to meet full formal diagnostic criteria for depression, or required all participants to meet full formal diagnostic criteria for an anxiety disorder. This was to ensure a reasonably broad recruitment strategy, and to further reduce the risk that the treamtent had focused overwhealmingly on one specific psychiatric disorder. We also excluded RCTs that required all participants to meet full formal diagnostic criteria for both depression and an anxiety disorder at the same time. This is a very unusual design that requires all participants to have a very substantial level of symptomatology that we suspected could be indicative of a highly specialized care setting unlike primary care which we were primarily interested in. Studies that subsumed obsessive-compulsive disorder or post-traumatic stress disorder under the umbrella of anxiety disorders, in accordance with earlier DSM

For peer review only - http://bmjopen.bmj.com/site/about/guidelines.xhtml

versions, were included. Studies that included a substantial proportion of subclinical participants were included only if clinical participants were reported separately.

(e) Studies were required to evaluate treatments for samples that were representative for a general medical or mental health clinical setting in the sense that the sample had not been heavily selected on any particular clinical characteristic or medical condition other than that of primary interest. This led to the exclusion for example of treatments specifically aimed at cancer patients with depression or anxiety.

(f) Studies were required to have at least 10 participants in one arm enrolled in internet-delivered transdiagnostic psychological treatment. All control groups were included.

(g) Studies were required to have employed at least one valid self-report questionnaire for depression or anxiety. Domain-specific anxiety scales such as those concerning social anxiety, health anxiety, or panic disorder anxiety were not considered adequate.

(h) Post-treatment assessment needed to be completed within three months after treatment termination, so as to not lie closer to a typical follow-up assessment.

(i) Studies were required to have reported means and standard deviations, or provided information that made it possible to derive such estimates. Typically, this was based on the original publication. In certain cases, conversions were applied, for example when the standard deviation could be derived from the sample size and the standard error of the mean. Whenever necessary, the author was also contacted and encouraged to provide estimates via email.

<Data extraction>

Pre-specified variables were extracted by the first author (KK), including participant demographics (e.g., age and gender). Studies were considered to concern children or adolescents if the mean age was below 18, and to concern older adults if the mean age was

For peer review only

above 65. The following study and sample characteristics were extracted: study setting (primary care, not primary care, or unclear), recruitment path (routine care or not routine care), main inclusion criterion (psychiatric diagnosis or "cut-off symptoms"), mean depression severity (mild, moderate, or severe), and mean anxiety severity (mild, moderate, or severe). Symptom severity ratings were based on published norm data.(25-28) Treatment characteristics extracted were: delivery format (website, mobile app, video, mixed or other), therapist support (guided or unguided), treatment type (CBT, mindfulness, mixed CBT and mindfulness, or other), specific treatment protocol, treatment length (≥6 weeks or <6 weeks; intended to contrast what are usually considered brief therapies with therapies of a more conventional length), component flexibility (standardized or tailored), and therapist time (minutes per participant in treatment). In line with recent findings demonstrating that many treatment-as-usual control groups are virtually indistinguishable from waitlist controls,(29) we did not lump all treatment-as-usual controls into the same category but rather made a distinction between (a) rudimentary passive controls that comprised waitlist controls and other controls without a structured intervention, (b) attention/engagement controls where the participants received some sort of standardized intervention that controlled for the attention from a caregiver or engagement in a study but without this being a conventional psychological treament, and (c) bona-fide treatments "delivered by trained therapists and [...] based on psychological principles, [...] offered to the psychotherapy community as viable treatments".(30) For each trial, the primary outcome for depression and anxiety was tabulated. If no outcome was defined as primary, or the primary outcome measure was deemed not to be valid, we tabulated the first valid measure that was mentioned. KK contacted the corresponding author when main outcome data was missing. All authors were also asked to provide pre-post correlations for the calculation of within-group effects. Intention-to-treat estimates were tabulated whenever available.

For peer review only

<*Assessment of risk of bias and quality of the evidence base*>

Trial risk of bias was assessed using a modified version of the Cochrane risk-of-bias tool for randomized trials version 2 (RoB 2).(31) The RoB 2 covers five domains: (i) the randomization process, (ii) deviations from intended interventions, (iii) missing outcome data, (iv) measurement of the outcome, and (v) selection of the reported results. Because of the difficulty blinding therapists to the psychological treatment they are delivering, we omitted items related to blinding. Consistent with previous similar research, non-blinding did not therefore lead to an elevated risk of bias.(15 32) Each trial received one rating on each risk of bias domain, and this rating concerned both the depression and the anxiety outcome. Reporting one score was sufficient because all trials administered the depression and anxiety measures in parallel, which resulted in identical ratings. Authors KK and EL independently assessed the trials and discussed cases of disagreement to reach consensus. The decision was made that if five articles in a row were rated identically by both assessors, it would no longer be necessary with two assessments per article. This target was achieved after 25 articles, and KK assessed the remaining trials. Based partially on the risk of bias ratings, we assessed the overall quality of the evidence base according to GRADE.(33) We assigned separate ratings for the evidence base pertaining to (i) effects versus rudimentary passive controls for adults, (ii) effects versus attention/engagement controls for adults, (iii) effects versus other bona-fide treatments for adults, (iv) effects for children and adolescents, and (v) effects in primary care. Similar to the risk of bias assessment, one common score was given for depression and anxiety.

For peer review only

For peer review only - http://bmjopen.bmj.com/site/about/guidelines.xhtml

<Statistical analysis>

We report studies focusing on children and adolescents, and studies focusing on older adults, separately due to their specific focus. Effects versus other bona fide treatments were too few to be included in meta-analysis. At the end of the tabulation phase, we also decided to report studies with a treatment length of two weeks or shorter separately, a duration which would not typically be regarded as bona fide in routine clinical care. Nine randomized factorial trials were also included in the meta-analysis of within-group effects, but their main results were summarized separately.

We proceeded with meta-analysis using random-effects models fitted with the restricted maximum likelihood estimator using the inverse variance method, in R 4.2.0(34) with the metafor 3.8-1 package.(35) Effects were quantified in terms of Hedges' *g*, i.e., the bias-corrected standardized mean difference.(36) Although not our primary focus, we first conducted a meta-analysis of the within-group change in treatment. The point of this was to ensure that change had been observed per se, to determine if heterogeneity was much reduced without the influence of variance due to different control groups, and to facilitate the interpretation of within-group effects in future studies. For this analysis, we standardized *g* on the pre-treatment standard deviation, and used the pre-post correlation (imputed pooled estimate if missing) to determine the sampling variance (37 38):

$$g = \frac{M_{pre} - M_{post}}{SD_{pre}} \times \sqrt{\frac{2}{(n-1)}}\left(\frac{\Gamma[(n-1)/2]}{\Gamma[(n-2)/2]}\right)$$

$$approx.\,variance(g) = \frac{2(1 - r_{prepost})}{n} + \frac{g^2}{2n}$$

We then conducted a meta-analysis of between-group effects versus rudimentary passive controls, and a meta-analysis of between-group effects versus attention/engagement controls. For these analyses, we standardized *g* on on the pooled post-treatment standard deviation, and employed the large-sample approximation of the sampling variance (36 39):

For peer review only - http://bmjopen.bmj.com/site/about/guidelines.xhtml

$$g = -\frac{M_{tx} - M_{control}}{SD_{pooled}} \times \sqrt{\frac{2}{(n_{tx} + n_{control} - 2)}} \left(\frac{\Gamma[(n_{tx} + n_{control} - 2)/2]}{\Gamma[(n_{tx} + n_{control} - 3)/2]} \right)$$

$$approx.\,variance(g) = \frac{n_{tx} + n_{control}}{n_{tx} n_{control}} + \frac{g^2}{2(n_{tx} + n_{control})}$$

Absolute values for *g* of 0.2 are usually regarded as small, 0.5 as moderate, and 0.8 as large.(40) We quantified absolute heterogeneity in terms of the τ^2, and also report the I^2 which stands for the proportion of the between-sample variance that is due to true study differences as opposed to sampling error. An I^2 of 25% is usually regarded as low, 50% as moderate, and 75% as high,(41) though the I^2 also increases with the precision of the original trials.(42) Pertaining to the meta-analysis of trials of transdiagnostic treatment vs. rudimentary passive controls, a series of planned moderator analyses were conducted on the basis of trial, sample, and treatment characteristics whenever strata included at least four studies. One exception was made to this rule where we analyzed primary care setting as a potential moderator based on four arms from three trials because we found this test important. In the analysis of trials with rudimentary controls, we also investigated publication bias based on the visual inspection of funnel plots in combination with Egger's test(43) and the Duval and Tweedie trim and fill procedure.(44) In line with recommendations,(45) the latter was repeated using the R0, L0, and Q0 estimators.

<Patient and Public Involvement>

None<Results>

The inclusion process is presented in Fig. 1. Out of 435 RCTs reviewed in full text, 55 were suggested for inclusion by both assessors, 335 were immediate exclusions, and 45 were disagreements (90% agreement; κ = 0.65). The reasons for these disagreements are listed in Supplement B. Of the disagreements, five trials were included after discussion. Three trials

For peer review only

For peer review only - http://bmjopen.bmj.com/site/about/guidelines.xhtml

were then excluded due to insufficient data. This resulted in a final sample of 57 RCTs. Full references to these trials are found in Supplement B.

INSERT FIGURE 1 ABOUT HERE

<*Study characteristics*>

In the 57 included trials, 21795 participants were randomized.(46-102)Arms were: 83 internet-delivered transdiagnostic psychological treatments, 36 rudimentary controls, 8 attention/engagement controls, and three other bona fide treatments. With regard to age groups, 53/57 trials (93%) recruited adults, 3/57 (5%) children or adolescents, and 1/57 (2%) specifically older adults. As to delivery formats, 37/57 (65%) of the internet-delivered transdiagnostic psychological treatments were delivered via a website mainly through text, 8/57 (14%) via a mobile app, 3/57 (5%) via video, and 9/57 (16%) were mixed or other. In terms of the therapeutic model, 37/57 (65%) evaluated some variation on cognitive behavioral therapy (CBT), 8/57 (14%) mindfulness-based therapy, 3/57 (5%) mixed CBT and mindfulness, and 11/57 (19%) other frameworks such as problem-solving therapy or affect-focused psychodynamic therapy. As for the recruitment strategy, 39/57 (68%) trials relied on self-referrals, 9/57 (16%) recruited from routine care including student counselling, and 9/57 (16%) used a mixed strategy or other methods such as recruitment via a public health care website. Illustrating the lack of work in the routine clinical setting, 54/57 trials (95%) did not report having been conducted in primary health care, and 3/57 (5%) reported having been conducted in primary health care. The trials were based in Australia 15/57 (26%), Canada 12/57 (21%), United States 6/57 (11%,), United Kingdom 5/57 (9%), Sweden 4/57 (7%), the Netherlands 3/57 (5%), and other countries 12/57 (21%). More trial characteristics are tabulated in the online Supplement B.

For peer review only - http://bmjopen.bmj.com/site/about/guidelines.xhtml

<Study risk of bias, and quality of the evidence base>

The distribution of study risk-of-bias ratings is presented in Figure 2. Overall, 9/57 (16%) of the trials were rated as having a low risk of bias, 14/57 (25%) as raising some concerns, and 34/57 (60%) as having a high risk of bias. The most common reason for an overall high risk was missing outcome data, which was seen in 29/59 (51%) of the trials, followed by deviations from the intended interventions in 15/57 (26%). Pooled effect sizes did not differ between trials as a function of the overall risk of bias (see Table 1). In all outcome domains, the overall quality of the evidence base was very low (see Supplement C for details).

INSERT FIGURE 2 ABOUT HERE

<Effects on depression and anxiety>

For adults, the pooled within-group reduction in depression symptoms was large (g = 0.90; 95% CI 0.81-0.99; k = 78) and the heterogeneity was high (I^2 = 95%; τ^2 = 0.14; Q_{77} =1606, p = < 0.001). The pooled within-group reduction in anxiety was also large (g = 0.87; 95% CI 0.78-0.96; k = 78) and the heterogeneity was high (I^2 = 95%; τ^2 = 0.16; Q_{77} = 1882, p = < 0.001). The pooled between-group effect versus the rudimentary controls was moderate for depression symptoms (g = 0.52; 95% CI 0.42-0.63; k = 43) with high heterogeneity (I^2 = 80%; τ^2 = 0.08; Q_{42} = 162, p = < 0.001), and moderate for anxiety (g = 0.45; 95% CI 0.34-0.56; k = 43) with high heterogeneity (I^2 = 83%; τ^2 = 0.10; Q_{42}= 176, p = < 0.001). See Fig. 3. The pooled between-group effect versus the attention/engagement controls was also significant but small for depression symptoms (g = 0.30; 95% CI 0.07-0.53; k = 10; I^2 = 91%; τ^2 = 0.12; Q_9 = 48, p = < 0.001) and small for anxiety (g = 0.21; 95% CI 0.01-0.42; k = 10; I^2 = 88%; τ^2 = 0.09; Q_9 = 45, p = < 0.001). See Fig. 4.

For peer review only - http://bmjopen.bmj.com/site/about/guidelines.xhtml

INSERT FIGURE 3 AND 4 ABOUT HERE

<*Risk of publication bias*>

We explored indicators of publication bias in the meta-analysis of effects versus rudimentary passive controls. For depression, there was clear indication of small- to medium-sized trials with non-beneficial effects of internet-delivered psychological treatments being underreported, and with imputed studies the between-group effect dropped from 0.52 to 0.29 (95% CI 0.16-0.42). For anxiety, there were also indication of possible asymmetry in the same direction, but this was more ambiguous, and the trim-and-fill procedure did not result in any imputed trials. See tSupplement B for details.

<*Moderators and subgroups*>

The results of the planned moderator and subgroup analyses are presented in Table 1. With regard to between-group effects versus the rudimentary controls, one moderator was found to be significant for both depression and anxiety symptoms: trials that required all participants to meet full criteria for a psychiatric diagnosis reported a larger pooled between-group effect as compared to trials that did not have this requirement. We also identified 9 randomized factorial trials. These manipulated various aspects of the treatment format and reported mostly null results with small effect sizes. In single trials, small moderating effects were reported for therapist guidance, app recommendations, and pre-treatment motivational interviewing.(70 96) In other trials, null results were however also reported for guidance and motivational interviewing.(51 80)

For peer review only

Table 1. Moderator and subgroup analyses of the relative effect of Internet-delivered transdiagnostic psychological treatment versus rudimentary controls.

**DEPRESSION SYMPTOMS**								
**Potential moderator**	***Q (df)***	***p***	**Subgroup**	***k***	***g***	***95% CI***	***I²***	**τ²**
Pooled total				43	0.52	0.42-0.63	80%	0.08
Study and sample characteristics								
Study setting	2.14 (1)	0.144	Primary health care	4	0.30†	-0.03-0.62	79%	0.08
			Not or unclear	39	0.55	0.44-0.66		
Recruitment path	3.32 (1)	0.068	Routine care	8	0.32	0.07-0.57	81%	0.09
			Not routine care	31	0.58	0.45-0.71		
Main inclusion criterion	6.33 (1)	0.012*	Psychiatric diagnosis	12	0.74	0.54-0.94	78%	0.08
			Cut-off or symptoms	31	0.44	0.32-0.56		
Depression severity	2.31 (2)	0.315	Mild	5	0.46	0.18-0.74	68%	0.06
			Moderate	32	0.53	0.42-0.64		
			Severe	4	0.28†	-0.04-0.59		
Anxiety severity	1.29 (1)	0.255	Mild or moderate	30	0.46	0.35-0.56	74%	0.06
			Severe	10	0.59	0.38-0.79		
Treatment characteristics								
Delivery format	1.51 (2)	0.471	Website	29	0.50	0.38-0.63	79%	0.08
			Mobile app	6	0.43	0.09-0.77		
			Mixed or other	8	0.66	0.41-0.90		
Therapist support	0.91 (1)	0.341	Yes, guided	23	0.58	0.43-0.73	80%	0.08
			No, unguided	20	0.47	0.32-0.62		
Treatment type	4.67 (2)	0.097	CBT	29	0.53	0.41-0.66	77%	0.27
			Mindfulness	4	0.88	0.49-1.27		
			Other	10	0.40	0.21-0.60		
Specific protocols	0.06 (1)	0.812	Wellbeing course	6	0.49	0.21-0.78	81%	0.09
			Not wellbeing course	37	0.53	0.41-0.65		
Treatment length	0.02 (1)	0.901	≥6 weeks	28	0.53	0.40-0.66	79%	0.09
			<6 weeks	15	0.52	0.33-0.70		
Component flexibility	1.00 (1)	0.318	Standardized	36	0.56	0.44-0.68	81%	0.09
			Tailored	5	0.39	0.08-0.70		
Therapist time††	0.88 (1)	0.349	Per additional 10 min	28	0.00†	-0.01-0.01	63%	0.03
Overall study risk of bias								

For peer review only

For peer review only - http://bmjopen.bmj.com/site/about/guidelines.xhtml

Cochrane tool version 2	0.84 (2)	0.658	High	29	0.49	0.36-0.63	81%	0.09
			Some concerns	8	0.55	0.30-0.80		
			Low	6	0.63	0.35-0.91		

ANXIETY

**Potential moderator**	***Q (df)***	***p***	**Subgroup**	***k***	***g***	***95% CI***	***I²***	**τ²**
Pooled total				43	0.45	0.34-0.56	83%	0.10
Study and sample characteristics								
Study setting	0.91 (1)	0.340	Primary health care	4	0.28†	-0.07-0.64	83%	0.10
			Not or unclear	39	0.47	0.35-0.59		
Recruitment path	2.19 (1)	0.139	Routine care	8	0.30	0.04-0.55	82%	0.10
			Not routine care	31	0.52	0.38-0.65		
Main inclusion criterion	7.01 (1)	0.008*	Psychiatric diagnosis	12	0.68	0.48-0.89	81%	0.09
			Cut-off or symptoms	31	0.36	0.23-0.48		
Depression severity	2.60 (2)	0.272	Mild	5	0.51	0.20-0.81	74%	0.08
			Moderate	32	0.44	0.32-0.56		
			Severe	4	0.16†	-0.18-0.50		
Anxiety severity	0.85 (1)	0.358	Mild or moderate	30	0.38	0.26-0.50	79%	0.07
			Severe	10	0.50	0.28-0.72		
Treatment characteristics								
Delivery format	0.02 (2)	0.988	Website	29	0.45	0.31-0.59	83%	0.11
			Mobile app	6	0.42	0.06-0.79		
			Mixed or other	8	0.46	0.19-0.73		
Therapist support	1.67 (1)	0.196	Yes, guided	23	0.52	0.36-0.68	82%	0.10
			No, unguided	20	0.38	0.22-0.53		
Treatment type	5.57 (2)	0.062	CBT	29	0.44	0.31-0.57	80%	0.29
			Mindfulness	4	0.89	0.48-1.30		
			Other	10	0.34	0.13-0.55		
Specific protocols	0.96 (1)	0.328	Wellbeing course	6	0.31	0.00-0.61	84%	0.10
			Not wellbeing course	37	0.47	0.35-0.60		
Treatment length	0.00 (1)	0.973	≥6 weeks	28	0.45	0.31-0.59	83%	0.11
			<6 weeks	15	0.45	0.25-0.66		
Component flexibility	2.22 (1)	0.136	Standardized	36	0.49	0.36-0.61	84%	0.11
			Tailored	5	0.22†	-0.11-0.55		
Therapist time††	0.47 (1)	0.494	Per additional 10 min	28	0.00†	-0.01-0.02	85%	0.09

For peer review only - http://bmjopen.bmj.com/site/about/guidelines.xhtml

Overall study risk of bias								
Cochrane tool version 2	0.01 (2)	0.995	High	29	0.45	0.31-0.60	84%	0.11
			Some concerns	8	0.44	0.17-0.71		
			Low	6	0.46	0.16-0.76		

In accordance with the study protocol, strata were collapsed or excluded from analysis if *k* < 4. For anxiety severity, "mild" (*k* = 3) and "moderate" were collapsed. For delivery formats, "video" (*k* = 3) was subsumed under "mixed or other". For treatment types, "CBT and mindfulness" (*k* = 3) was subsumed under "other". Q-tests listed in this table are tests of moderation, with the intercept included in the statistical model. Heterogeneity is reported as I^2 and τ^2. CBT, cognitive behavior therapy.

* Test of moderation significant at $\alpha = 0.05$.

† Pooled effect not significantly different from zero.

†† Therapist time per participant was tested as a continuous variable.

<Effects versus other bona fide treatments>

Three trials compared the effects of an internet-delivered transdiagnostic psychological treatment to those of a distinctly different bona fide treatment. They showed mixed results and are described in Supplement B.(49) (79) (91)

<Children, adolescents, and older adults>

Two trials recruited children or adolescents. One trial recruited ages 12-21 (M=16, SD=2; N=45) and reported null effects of 5 weeks of problem-solving therapy on depression (g = 0.04) and anxiety (g = -0.11) as compared to a waitlist.(62) The second trial recruited ages 13-18 (M=16, SD=1; N=103) in a school setting and found that a single session promoting growth, gratitude, and values had significant and moderate added effects on depression (g = 0.50) but not anxiety (g = 0.32) as compared to a control that focused on the promotion of

For peer review only

For peer review only - http://bmjopen.bmj.com/site/about/guidelines.xhtml

study skills.(74) One trial exclusively recruited older adults, aged 60+ (M=66, SD=5; N=433), and found that 8 weeks of CBT in a structured text-based self-help format was associated with large within-group reductions in depression (*g*s = 1.19-1.30) and anxiety (*g*s = 1.21-1.31), but that there was no significant added effect of adding guidance or an initial interview.(85)

<Discussion>

In this systematic review with meta-analysis based on 57 randomized controlled trials we found that, for adults, internet-delivered transdiagnostic psychological treatments led to moderate between-group effects compared to rudimentary controls (depression: g = 0.52; anxiety: g = 0.45) and small between-group effects compared to attention/engagement controls (depression: g = 0.30; anxiety: g = 0.21). Heterogeneity was substantial. There were also indications of possible publication bias, which probably inflated the pooled effect on depression versus rudimentary controls – perhaps as much as 0.23 units. One significant moderator was found: trials where all participants were required to meet full diagnostic criteria for depression or an anxiety disorder reported larger between-group effects versus rudimentary controls, than did trials without this requirement. Strengths of the present study include the systematic search strategy and the broad eligibility criteria, which allowed for an overview of treatment frameworks, formats, settings including primary health care, and also age groups. On the whole, the overall quality of the evidence base was very low, which implies that there is considerable uncertainty about true effects.

For peer review only - http://bmjopen.bmj.com/site/about/guidelines.xhtml

<Comparison with prior research>

Our final sample of 57 RCTs pertaining specifically to internet-delivered transdiagnostic psychological treatments suitable for both primary depression and primary anxiety disorders is large compared to previous systematic reviews.(15) This is probably explained both by the rapid growth of this research field, and our use of exhaustive search terms developed with the specific aim of identifying internet-delivered transdiagnostic psychological treatments. The between-group effect versus rudimentary controls was slightly smaller than expected, though still broadly in line with previous meta-analyses.(15 16) Our expectation, based on previous research,(17) that treatment length would be of little importance for the between-group effect was also supported in our data. Similarly, the finding that effects on depression are likely to be inflated by publication bias is also in line with previous research and therefore not unexpected.(103)

<Children, adolescents, and older adults>

This exhaustive literature search showed very clearly that the internet-delivered transdiagnostic treatments for children and adolescents, as well as specifically for older adults, have not come very far. Especially for children and adolescents, we found this rather surprising considering the relatively long tradition of treatments intended to suit a wide spectrum of anxiety-, but not necessarily mood, disorders.(104) Conceivably, this may have reduced the incentive to develop newer protocols that can accommodate patients with depression as well. For older adults, we identified a single study which presented promising results. It is widely acknowledged that older adults rarely receive psychological treatment(105 106). We wish to encourage further work in this area, especially with increased technical prowess in this population.

For peer review only - http://bmjopen.bmj.com/site/about/guidelines.xhtml

<Possible benefit of meeting a diagnosis>

In the exploratory moderator analyses, the only variable that was a significant moderator of the between-group effect of treatment was whether participants were required to meet full criteria for a psychiatric diagnosis. One interpretation is that of a "specificity effect", where larger effects can be expected if patients suffer from clinical depression or an anxiety disorder, as opposed to symptoms caused for example by a somatoform disorder or a somatic disease.

<Treatment characteristics>

Except for the possible benefits of a diagnosis, a general pattern in both the planned tests of potential moderators and the original factorial trials was the lack of significant moderators, not least in terms of treatment characteristics. One interesting finding was for example that therapist-guidance was not found to be a significant moderator in our meta-analyses, and that results from the factorial trials were inconsistent. We found that it is still unusual for trials to investigate the effect of treatments other than CBT or mindfulness-based interventions. There were, however, also examples of other treatments investigated in more than one trial, namely problem-solving therapies(62 65 89) and bias-modification training therapies.(61 97)

<New formats: Are we at a crossroads?>

For the past quarter of a century, internet-delivered psychological treatments have mostly been delivered via web platforms, with standardized linear text presentations intended to be accessed via a web browser. This systematic review illustrates that this treatment format is still most widely researched, and that newer formats remain understudied. We were able to pool the mean effects of 6 arms from 5 trials focusing on mobile apps, and 5 arms from 5 trials of tailored treatments, which showed no significant differences compared to the more

For peer review only

common standardized web format. Notably, only three trials investigated videoconferencing interventions(86 93 100), and only one trial investigated the effect of an intervention using artificial intelligence(54). The vast majority of the literature still pertains to the standardized web-based text format, but the direction of the field appears to be towards automated, tailored, interventions.

<Limitations>

A clear limitation of the original studies was that the majority of trials recruited self-referred participants through advertisement and were not conducted in clinical setting. The fact that these samples might have been highly motivated, and not representative of patients in routine care, makes it more difficult to draw conclusions about effectiveness in routine care. Another limitation of this review is that our analyses of moderators were based exclusively on trial-level characteristics. This implies poor power, and often restriction of range, in many tests such as that of whether pooled effects differ as a function of mean baseline symptom level. This review included studies published in English only, which means that RCTs published solely in other languages were systematically overlooked. Another aspect that warranted lowered GRADE ratings was the considerable heterogeneity in reported effects, which remained largely unexplained and often implied poor precision. Last, we remind the reader that being enrolled in a psychological treamtent implies engaging with the content of that treatment, which often includes its theoretical basis. Therefore, therapists and patients cannot be fully blinded to the intervention, and effects may depend partially on expectancy effects, including therapist's and patient's presumptions about "what ought to work".

For peer review only - http://bmjopen.bmj.com/site/about/guidelines.xhtml

<Clinical implications and recommendations for future research>

This meta-analysis contributes tentative evidence that transdiagnostic psychological treatments are effective for people with elevated depression symptoms or anxiety. However, even though a substantial proportion of patients are treated in routine care settings such as primary care,(6 107) only three (5%) of the 57 included trials were conducted in a primary care setting. Power was limited to quantify the pooled effect of this stratum, and *g* did not reach statistical significance. This means that there is an urgent need for RCTs evaluating internet-delivered transdiagnostic treatments based on a broad recruitrment strategies in the routine clinical context. It is also important when planning for future RCTs to reduce currently high levels of missing data. More studies evaluating video-delivered interventions are needed. Adequately powered RCTs comparing internet-delivered transdiagnostic treatments with other bona fide treatments for depression and anxiety are also needed.

<Conclusion>

Internet-delivered transdiagnostic treatments suitable for both depression and anxiety appear to have moderate effects compared to rudimentary controls, and significant but small effects beyond those of attention/engagement controls. Evidence is lacking with regard to routine care, the video format, children and adolescents, and older adults.

For peer review only

Acknowledgements: We thank the Karolinska Institutet library service, and in particular Sabina Gillsund and Emma-Lotta Säätelä, for contributing to the systematic database search. We would also like to thank all study authors who responded to our queries and contributed with data on request.

Competing interests: KK, AHB, EHL, EL, JH, and EA have taken part in original research on internet-delivered treatment including transdiagnostic treatment.

Contributions of authors: KK and EA led the study design and data collection process, with input from all authors. KK and EA conducted the statistical analysis. KK wrote the initial draft in close collaboration with EA. All authors reviewed the manuscript for intellectual content, made contributions, and approved the final version. KK and EA had full access to all data and take responsibility for the integrity of the data and the accuracy of the presented analysis.

Funding: This work was supported by the Swedish Research Council (EA; identifier: 2021-06496) and the Krica Foundation. Neither of the funders had any role in the design, execution, or publication process.

Availability of data: Tabulated data is available from the authors on request.

Ethics Approval: Not applicable

Karoline Kolaas, PhD, Karolinska Institutet, Dept of Clin. Neuroscience, Stockholm, Sweden, **Anne H Berman,** PhD, Uppsala University, Dept of Psychology, Uppsala, Sweden,

For peer review only

For peer review only - http://bmjopen.bmj.com/site/about/guidelines.xhtml

Erik Hedman-Lagerlöf, PhD, Karolinska Institutet, Dept of Clin. Neuroscience, Stockholm, Sweden, **Elin Lindsäter**, PhD, Karolinska Institutet, Dept of Clin. Neuroscience, Stockholm, Sweden, **Jonna Hybelius**, MSc, Karolinska Institutet, Dept of Neurobiology, Care Sciences and Society, Stockholm, Sweden, & **Erland Axelsson**, PhD, Karolinska Institutet, Dept of Neurobiology, Care Sciences and Society, Stockholm, Sweden

For peer review only

For peer review only - http://bmjopen.bmj.com/site/about/guidelines.xhtml

Figure captions:

Figure 1 PRISMA flowchart of the study selection process. RCT, randomized controlled trial; SD, standard deviation.

Figure 2 Study risk of bias based on the Cochrane Risk of Bias Tool version 2. Note that blinding was not assessed, which means that the lack of blinding did not result in an elevated risk score.

Figure 3 Standardized effect on depression and anxiety symptoms vs rudimentary passive controls.

Figure 4 Standardized effect on depressioin and anxiety symptoms vs attention/engagement controls.

For peer review only

For peer review only - http://bmjopen.bmj.com/site/about/guidelines.xhtml

References

1. GBD 2019 Mental Disorders Collaborators. Global, regional, and national comparative risk assessment of 84 behavioural, environmental and occupational, and metabolic risks or clusters of risks for 195 countries and territories, 1990-2017: a systematic analysis for the Global Burden of Disease Study 2017. *Lancet* 2018;392(10159):1923-94. doi: 10.1016/s0140-6736(18)32225-6 [published Online First: 20181108]

2. Bandelow B, Michaelis S. Epidemiology of anxiety disorders in the 21st century. *Dialogues Clin Neurosci* 2015;17(3):327-35. doi: 10.31887/DCNS.2015.17.3/bbandelow

3. Cuijpers P, Cristea IA, Karyotaki E, et al. How effective are cognitive behavior therapies for major depression and anxiety disorders? A meta-analytic update of the evidence. *World Psychiatry* 2016;15(3):245-58. doi: 10.1002/wps.20346 [published Online First: 2016/10/08]

4. Santoft F, Axelsson E, Öst LG, et al. Cognitive behaviour therapy for depression in primary care: systematic review and meta-analysis. *Psychological medicine* 2019:1-9. doi: 10.1017/S0033291718004208 [published Online First: 2019/01/29]

5. Twomey C, O'Reilly G, Byrne M. Effectiveness of cognitive behavioural therapy for anxiety and depression in primary care: a meta-analysis. *Family practice* 2015;32(1):3-15. doi: 10.1093/fampra/cmu060 [published Online First: 20140922]

6. Alonso J, Angermeyer MC, Bernert S, et al. Use of mental health services in Europe: results from the European Study of the Epidemiology of Mental Disorders (ESEMeD) project. *Acta psychiatrica Scandinavica Supplementum* 2004(420):47-54. doi: 10.1111/j.1600-0047.2004.00330.x [published Online First: 2004/05/07]

For peer review only - http://bmjopen.bmj.com/site/about/guidelines.xhtml

7. Thornicroft G, Chatterji S, Evans-Lacko S, et al. Undertreatment of people with major depressive disorder in 21 countries. *Br J Psychiatry* 2017;210(2):119-24. doi: 10.1192/bjp.bp.116.188078 [published Online First: 20161201]

8. Alonso J, Liu Z, Evans-Lacko S, et al. Treatment gap for anxiety disorders is global: Results of the World Mental Health Surveys in 21 countries. *Depression and anxiety* 2018;35(3):195-208. doi: 10.1002/da.22711 [published Online First: 20180122]

9. McGrath JJ, Lim CCW, Plana-Ripoll O, et al. Comorbidity within mental disorders: a comprehensive analysis based on 145 990 survey respondents from 27 countries. *Epidemiol Psychiatr Sci* 2020;29:e153. doi: 10.1017/s2045796020000633 [published Online First: 20200812]

10. Jacobson NC, Newman MG. Anxiety and depression as bidirectional risk factors for one another: A meta-analysis of longitudinal studies. *Psychol Bull* 2017;143(11):1155-200. doi: 10.1037/bul0000111 [published Online First: 20170814]

11. Sloan E, Hall K, Moulding R, et al. Emotion regulation as a transdiagnostic treatment construct across anxiety, depression, substance, eating and borderline personality disorders: A systematic review. *Clinical psychology review* 2017;57:141-63. doi: 10.1016/j.cpr.2017.09.002 [published Online First: 20170911]

12. Wilamowska ZA, Thompson-Hollands J, Fairholme CP, et al. Conceptual background, development, and preliminary data from the unified protocol for transdiagnostic treatment of emotional disorders. *Depression and anxiety* 2010;27(10):882-90. doi: 10.1002/da.20735

13. Ringwald WR, Forbes MK, Wright AGC. Meta-analysis of structural evidence for the Hierarchical Taxonomy of Psychopathology (HiTOP) model. *Psychological medicine* 2023;53(2):533-46. doi: 10.1017/S0033291721001902 [published Online First: 2021/05/14]

For peer review only

For peer review only - http://bmjopen.bmj.com/site/about/guidelines.xhtml

14. Kolaas K, Berman AH, Hedman-Lagerlöf E, et al. Feasibility of a video-delivered mental health course for primary care patients: a single-group prospective cohort study. *BMC Prim Care* 2023;24(1):28. doi: 10.1186/s12875-023-01989-8 [published Online First: 20230123]

15. Cuijpers P, Miguel C, Ciharova M, et al. Transdiagnostic treatment of depression and anxiety: a meta-analysis. *Psychological medicine* 2023:1-12.

16. Newby JM, Twomey C, Yuan Li SS, et al. Transdiagnostic computerised cognitive behavioural therapy for depression and anxiety: A systematic review and meta-analysis. *Journal of affective disorders* 2016;199:30-41. doi: https://doi.org/10.1016/j.jad.2016.03.018

17. Păsărelu CR, Andersson G, Bergman Nordgren L, et al. Internet-delivered transdiagnostic and tailored cognitive behavioral therapy for anxiety and depression: a systematic review and meta-analysis of randomized controlled trials. *Cogn Behav Ther* 2017;46(1):1-28. doi: 10.1080/16506073.2016.1231219 [published Online First: 20161007]

18. Thompson EM, Destree L, Albertella L, et al. Internet-based acceptance and commitment therapy: a transdiagnostic systematic review and meta-analysis for mental health outcomes. *Behavior therapy* 2021;52(2):492-507.

19. Etzelmueller A, Vis C, Karyotaki E, et al. Effects of Internet-Based Cognitive Behavioral Therapy in Routine Care for Adults in Treatment for Depression and Anxiety: Systematic Review and Meta-Analysis. *Journal of medical Internet research* 2020;22(8):e18100. doi: 10.2196/18100 [published Online First: 20200831]

20. Page MJ, McKenzie JE, Bossuyt PM, et al. The PRISMA 2020 statement: an updated guideline for reporting systematic reviews. *BMJ (Clinical research ed)* 2021;372:n71. doi: 10.1136/bmj.n71 [published Online First: 20210329]

For peer review only - http://bmjopen.bmj.com/site/about/guidelines.xhtml

21. Bramer W, Bain P. Updating search strategies for systematic reviews using EndNote. *J Med Libr Assoc* 2017;105(3):285-89. doi: 10.5195/jmla.2017.183 [published Online First: 20170701]

22. Bramer WM, Giustini D, de Jonge GB, et al. De-duplication of database search results for systematic reviews in EndNote. *J Med Libr Assoc* 2016;104(3):240-3. doi: 10.3163/1536-5050.104.3.014

23. Ouzzani M, Hammady H, Fedorowicz Z, et al. Rayyan-a web and mobile app for systematic reviews. *Systematic reviews* 2016;5(1):210. doi: 10.1186/s13643-016-0384-4 [published Online First: 20161205]

24. Bolland MJ, Avenell A, Grey A. Analysis of cluster randomised trials as if they were individually randomised. *Lancet Diabetes Endocrinol* 2023;11(2):75. doi: 10.1016/s2213-8587(22)00363-1 [published Online First: 20221219]

25. Crawford J, Cayley C, Lovibond PF, et al. Percentile norms and accompanying interval estimates from an Australian general adult population sample for self‐report mood scales (BAI, BDI, CRSD, CES‐D, DASS, DASS‐21, STAI‐X, STAI‐Y, SRDS, and SRAS). *Australian Psychologist* 2011;46(1):3-14.

26. Crawford JR, Garthwaite PH, Lawrie CJ, et al. A convenient method of obtaining percentile norms and accompanying interval estimates for self‐report mood scales (DASS, DASS‐21, HADS, PANAS, and sAD). *British Journal of Clinical Psychology* 2009;48(2):163-80.

27. Schalet BD, Cook KF, Choi SW, et al. Establishing a common metric for self-reported anxiety: linking the MASQ, PANAS, and GAD-7 to PROMIS Anxiety. *Journal of anxiety disorders* 2014;28(1):88-96.

For peer review only - http://bmjopen.bmj.com/site/about/guidelines.xhtml

28. Wahl I, Löwe B, Bjorner JB, et al. Standardization of depression measurement: a common metric was developed for 11 self-report depression measures. *Journal of clinical epidemiology* 2014;67(1):73-86.

29. Munder T, Geisshüsler A, Krieger T, et al. Intensity of Treatment as Usual and Its Impact on the Effects of Face-to-Face and Internet-Based Psychotherapy for Depression: A Preregistered Meta-Analysis of Randomized Controlled Trials. *Psychother Psychosom* 2022;91(3):200-09. doi: 10.1159/000521951 [published Online First: 20220214]

30. Wampold BE, Mondin GW, Moody M, et al. A meta-analysis of outcome studies comparing bona fide psychotherapies: Empiricially," all must have prizes.". *Psychological bulletin* 1997;122(3):203.

31. Sterne JAC, Savović J, Page MJ, et al. RoB 2: a revised tool for assessing risk of bias in randomised trials. *BMJ (Clinical research ed)* 2019;366:l4898. doi: 10.1136/bmj.l4898 [published Online First: 20190828]

32. Bisby MA, Karin E, Hathway T, et al. A meta-analytic review of randomized clinical trials of online treatments for anxiety: Inclusion/exclusion criteria, uptake, adherence, dropout, and clinical outcomes. *Journal of anxiety disorders* 2022;92:102638. doi: 10.1016/j.janxdis.2022.102638 [published Online First: 20221007]

33. GRADE. Grading quality of evidence and strength of recommendations. *BMJ (Clinical research ed)* 2004;328(7454):1490.

34. R Core Team. R: A Language and Environment for Statistical Computing. R Foundation for Statistical Computing, Vienna, Austria. 2016

35. Viechtbauer W. Conducting meta-analyses in R with the metafor package. *Journal of statistical software* 2010;36(3):1-48.

For peer review only

For peer review only - http://bmjopen.bmj.com/site/about/guidelines.xhtml

36. Hedges LV. Distribution theory for Glass's estimator of effect size and related estimators. *journal of Educational Statistics* 1981;6(2):107-28.

37. Becker BJ. Synthesizing standardized mean ‐ change measures. *British Journal of Mathematical and Statistical Psychology* 1988;41(2):257-78.

38. Morris SB. Distribution of the standardized mean change effect size for meta ‐ analysis on repeated measures. *British Journal of Mathematical and Statistical Psychology* 2000;53(1):17-29.

39. Hedges LV. Estimation of effect size from a series of independent experiments. *Psychological bulletin* 1982;92(2):490.

40. Cohen J. Statistical power analysis for the behavioral science. 2 ed: Hillsdale, NJ: Lawrence Erlbaum Associates 1988:24-27.

41. Higgins JP, Thompson SG, Deeks JJ, et al. Measuring inconsistency in meta-analyses. *BMJ* 2003;327(7414):557-60. doi: 10.1136/bmj.327.7414.557 [published Online First: 2003/09/06]

42. Rücker G, Schwarzer G, Carpenter JR, et al. Undue reliance on I 2 in assessing heterogeneity may mislead. *BMC medical research methodology* 2008;8:1-9.

43. Egger M, Davey Smith G, Schneider M, et al. Bias in meta-analysis detected by a simple, graphical test. *BMJ (Clinical research ed)* 1997;315(7109):629-34. doi: 10.1136/bmj.315.7109.629

44. Duval S, Tweedie R. Trim and fill: A simple funnel-plot-based method of testing and adjusting for publication bias in meta-analysis. *Biometrics* 2000;56(2):455-63. doi: 10.1111/j.0006-341x.2000.00455.x

45. Shi L, Lin L. The trim-and-fill method for publication bias: practical guidelines and recommendations based on a large database of meta-analyses. *Medicine* 2019;98(23)

For peer review only

For peer review only - http://bmjopen.bmj.com/site/about/guidelines.xhtml

46. Al-Refae M, Al-Refae A, Munroe M, et al. A self-compassion and mindfulness-based cognitive mobile intervention (Serene) for depression, anxiety, and stress: promoting adaptive emotional regulation and wisdom. *Frontiers in psychology* 2021;12:648087.

47. Batterham PJ, Calear AL, Farrer L, et al. Efficacy of a transdiagnostic self-help internet intervention for reducing depression, anxiety, and suicidal ideation in adults: randomized controlled trial. *Journal of medical Internet research* 2021;23(1):e22698.

48. Beshai S, Bueno C, Yu M, et al. Examining the effectiveness of an online program to cultivate mindfulness and self-compassion skills (Mind-OP): Randomized controlled trial on Amazon's Mechanical Turk. *Behaviour research and therapy* 2020;134:103724.

49. Borjalilu S, Mazaheri MA, Talebpour A. Effectiveness of mindfulness-based stress management in the mental health of Iranian university students: a comparison of blended therapy, face-to-face sessions, and mHealth app (Aramgar). *Iranian Journal of Psychiatry and Behavioral Sciences* 2019;13(2)

50. Carolan S, Harris PR, Greenwood K, et al. Increasing engagement with an occupational digital stress management program through the use of an online facilitated discussion group: Results of a pilot randomised controlled trial. *Internet interventions* 2017;10:1-11.

51. Dear BF, Fogliati VJ, Fogliati R, et al. Treating anxiety and depression in young adults: A randomised controlled trial comparing clinician-guided versus self-guided Internet-delivered cognitive behavioural therapy. *Aust N Z J Psychiatry* 2018;52(7):668-79. doi: 10.1177/0004867417738055 [published Online First: 20171024]

52. Díaz-García A, González-Robles A, García-Palacios A, et al. Negative and positive affect regulation in a transdiagnostic Internet-based protocol for emotional disorders: randomized controlled trial. *Journal of medical Internet research* 2021;23(2):e21335.

For peer review only - http://bmjopen.bmj.com/site/about/guidelines.xhtml

53. Farrer LM, Gulliver A, Katruss N, et al. A novel multi-component online intervention to improve the mental health of university students: Randomised controlled trial of the Uni Virtual Clinic. *Internet Interv* 2019;18:100276. doi: 10.1016/j.invent.2019.100276 [published Online First: 20190828]

54. Fitzpatrick KK, Darcy A, Vierhile M. Delivering cognitive behavior therapy to young adults with symptoms of depression and anxiety using a fully automated conversational agent (Woebot): a randomized controlled trial. *JMIR mental health* 2017;4(2):e7785.

55. González-Robles A, Díaz-García A, García-Palacios A, et al. Effectiveness of a transdiagnostic guided internet-delivered protocol for emotional disorders versus treatment as usual in specialized care: randomized controlled trial. *Journal of medical Internet research* 2020;22(7):e18220.

56. Graham AK, Greene CJ, Kwasny MJ, et al. Coached Mobile App Platform for the Treatment of Depression and Anxiety Among Primary Care Patients: A Randomized Clinical Trial. *JAMA psychiatry* 2020;77(9):906-14. doi: 10.1001/jamapsychiatry.2020.1011

57. Hadjistavropoulos HD, Schneider LH, Edmonds M, et al. Randomized controlled trial of internet-delivered cognitive behaviour therapy comparing standard weekly versus optional weekly therapist support. *Journal of anxiety disorders* 2017;52:15-24. doi: 10.1016/j.janxdis.2017.09.006 [published Online First: 20170923]

58. Hadjistavropoulos HD, Peynenburg V, Nugent M, et al. Transdiagnostic Internet-delivered cognitive behaviour therapy with therapist support offered once-weekly or once-weekly supplemented with therapist support within one-business-day: Pragmatic randomized controlled trial. *Internet Interv* 2020;22:100347. doi: 10.1016/j.invent.2020.100347 [published Online First: 20200831]

For peer review only - http://bmjopen.bmj.com/site/about/guidelines.xhtml

59. Hadjistavropoulos HD, Peynenburg V, Thiessen DL, et al. A pragmatic factorial randomized controlled trial of transdiagnostic internet-delivered cognitive behavioural therapy: Exploring benefits of homework reflection questionnaires and twice-weekly therapist support. *Internet Interv* 2020;22:100357. doi: 10.1016/j.invent.2020.100357 [published Online First: 20201201]

60. Hensel JM, Shaw J, Ivers NM, et al. A web-based mental health platform for individuals seeking specialized mental health care services: multicenter pragmatic randomized controlled trial. *Journal of medical Internet research* 2019;21(6):e10838.

61. Hirsch CR, Krahé C, Whyte J, et al. Effects of modifying interpretation bias on transdiagnostic repetitive negative thinking. *Journal of consulting and clinical psychology* 2020;88(3):226.

62. Hoek W, Schuurmans J, Koot HM, et al. Effects of Internet-based guided self-help problem-solving therapy for adolescents with depression and anxiety: a randomized controlled trial. *PLoS One* 2012;7(8):e43485. doi: 10.1371/journal.pone.0043485 [published Online First: 20120831]

63. Johansson R, Björklund M, Hornborg C, et al. Affect-focused psychodynamic psychotherapy for depression and anxiety through the Internet: a randomized controlled trial. *PeerJ* 2013;1:e102.

64. Kladnitski N, Smith J, Uppal S, et al. Transdiagnostic internet-delivered CBT and mindfulness-based treatment for depression and anxiety: A randomised controlled trial. *Internet Interv* 2020;20:14. doi: 10.1016/j.invent.2020.100310

65. Kleiboer A, Donker T, Seekles W, et al. A randomized controlled trial on the role of support in Internet-based problem solving therapy for depression and anxiety. *Behaviour research and therapy* 2015;72:63-71.

For peer review only

For peer review only - http://bmjopen.bmj.com/site/about/guidelines.xhtml

66. Krafft J, Potts S, Schoendorff B, et al. A randomized controlled trial of multiple versions of an acceptance and commitment therapy matrix app for well-being. *Behavior Modification* 2019;43(2):246-72.

67. Levin ME, Hicks ET, Krafft J. Pilot evaluation of the stop, breathe & think mindfulness app for student clients on a college counseling center waitlist. *Journal of American college health* 2022;70(1):165-73.

68. Lindegaard T, Seaton F, Halaj A, et al. Internet-based cognitive behavioural therapy for depression and anxiety among Arabic-speaking individuals in Sweden: a pilot randomized controlled trial. *Cognitive Behaviour Therapy* 2021;50(1):47-66.

69. McCloud T, Jones R, Lewis G, et al. Effectiveness of a mobile app intervention for anxiety and depression symptoms in university students: randomized controlled trial. *JMIR mHealth and uHealth* 2020;8(7):e15418.

70. Mohr DC, Schueller SM, Tomasino KN, et al. Comparison of the Effects of Coaching and Receipt of App Recommendations on Depression, Anxiety, and Engagement in the IntelliCare Platform: Factorial Randomized Controlled Trial. *Journal of medical Internet research* 2019;21(8):e13609. doi: 10.2196/13609 [published Online First: 20190828]

71. Mullin A, Dear BF, Karin E, et al. The UniWellbeing course: A randomised controlled trial of a transdiagnostic internet-delivered cognitive behavioural therapy (CBT) programme for university students with symptoms of anxiety and depression. *Internet Interventions* 2015;2(2):128-36.

72. Newby JM, Mackenzie A, Williams AD, et al. Internet cognitive behavioural therapy for mixed anxiety and depression: a randomized controlled trial and evidence of effectiveness in primary care. *Psychological medicine* 2013;43(12):2635-48.

For peer review only - http://bmjopen.bmj.com/site/about/guidelines.xhtml

73. Nilsson A, Sörman K, Klingvall J, et al. MyCompass in a Swedish context–lessons learned from the transfer of a self-guided intervention targeting mental health problems. *BMC psychiatry* 2019;19:1-11.

74. Osborn TL, Rodriguez M, Wasil AR, et al. Single-session digital intervention for adolescent depression, anxiety, and well-being: Outcomes of a randomized controlled trial with Kenyan adolescents. *Journal of consulting and clinical psychology* 2020;88(7):657-68. doi: 10.1037/ccp0000505 [published Online First: 20200511]

75. Powell J, Hamborg T, Stallard N, et al. Effectiveness of a web-based cognitive-behavioral tool to improve mental well-being in the general population: randomized controlled trial. *Journal of medical Internet research* 2013;15(1):e2.

76. Proudfoot J, Clarke J, Birch M-R, et al. Impact of a mobile phone and web program on symptom and functional outcomes for people with mild-to-moderate depression, anxiety and stress: a randomised controlled trial. *BMC psychiatry* 2013;13(1):1-12.

77. Robichaud M, Talbot F, Titov N, et al. Facilitating access to iCBT: a randomized controlled trial assessing a translated version of an empirically validated program using a minimally monitored delivery model. *Behavioural and Cognitive Psychotherapy* 2020;48(2):185-202.

78. Rollman BL, Belnap BH, Abebe KZ, et al. Effectiveness of online collaborative care for treating mood and anxiety disorders in primary care: a randomized clinical trial. *JAMA psychiatry* 2018;75(1):56-64.

79. Sethi S. Treating Youth Depression and Anxiety: A Randomised Controlled Trial Examining the Efficacy of Computerised versus Face‐to‐face Cognitive Behaviour Therapy. *Australian Psychologist* 2013;48(4):249-57. doi: 10.1111/ap.12006

For peer review only - http://bmjopen.bmj.com/site/about/guidelines.xhtml

80. Soucy JN, Hadjistavropoulos HD, Karin E, et al. Brief online motivational interviewing pre-treatment intervention for enhancing internet-delivered cognitive behaviour therapy: A randomized controlled trial. *Internet Interv* 2021;25:100394. doi: 10.1016/j.invent.2021.100394 [published Online First: 20210420]

81. Southwell S, Gould E. A randomised wait list-controlled pre–post–follow-up trial of a gratitude diary with a distressed sample. *The Journal of Positive Psychology* 2017;12(6):579-93.

82. Terides MD, Dear BF, Fogliati VJ, et al. Increased skills usage statistically mediates symptom reduction in self-guided internet-delivered cognitive–behavioural therapy for depression and anxiety: a randomised controlled trial. *Cognitive behaviour therapy* 2018;47(1):43-61.

83. Titov N, Dear BF, Johnston L, et al. Improving adherence and clinical outcomes in self-guided internet treatment for anxiety and depression: randomised controlled trial. *PloS one* 2013;8(7):e62873.

84. Titov N, Dear BF, Schwencke G, et al. Transdiagnostic internet treatment for anxiety and depression: a randomised controlled trial. *Behaviour research and therapy* 2011;49(8):441-52.

85. Titov N, Fogliati VJ, Staples LG, et al. Treating anxiety and depression in older adults: randomised controlled trial comparing guided v. self-guided internet-delivered cognitive–behavioural therapy. *BJPsych open* 2016;2(1):50-58.

86. Tönnies J, Hartmann M, Wensing M, et al. Mental health specialist video consultations versus treatment-as-usual for patients with depression or anxiety disorders in primary care: randomized controlled feasibility trial. *JMIR mental health* 2021;8(3):e22569.

For peer review only - http://bmjopen.bmj.com/site/about/guidelines.xhtml

87. Tulbure BT, Rusu A, Sava FA, et al. A web-based transdiagnostic intervention for affective and mood disorders: randomized controlled trial. *JMIR mental health* 2018;5(2):e8901.

88. Twomey C, O'Reilly G, Byrne M, et al. A randomized controlled trial of the computerized CBT programme, MoodGYM, for public mental health service users waiting for interventions. *British Journal of Clinical Psychology* 2014;53(4):433-50.

89. van Straten A, Cuijpers P, Smits N. Effectiveness of a web-based self-help intervention for symptoms of depression, anxiety, and stress: randomized controlled trial. *Journal of medical Internet research* 2008;10(1):e7.

90. Viskovich S, Pakenham KI. Randomized controlled trial of a web - based Acceptance and Commitment Therapy (ACT) program to promote mental health in university students. *Journal of clinical psychology* 2020;76(6):929-51.

91. Villemaire-Krajden R, Myhr G. Evaluating the use of a computerized CBT program for outpatients on a waitlist in a university CBT unit. *Journal of Psychiatric Practice®* 2019;25(4):268-78.

92. Zetterqvist K, Maanmies J, Ström L, et al. Randomized controlled trial of internet-based stress management. *Cognitive Behaviour Therapy* 2003;32(3):151-60.

93. Grégoire S, Beaulieu F, Lachance L, et al. An online peer support program to improve mental health among university students: A randomized controlled trial. *Journal of American College Health* 2022:1-13.

94. Hadjistavropoulos HD, Peynenburg V, Thiessen DL, et al. A randomized factorial trial of internet-delivered cognitive behavioural therapy: An 8-week program with or without extended support and booster lesson. *Internet Interv* 2022;27:100499. doi: 10.1016/j.invent.2022.100499 [published Online First: 20220206]

For peer review only - http://bmjopen.bmj.com/site/about/guidelines.xhtml

95. Isbăşoiu AB, Tulbure BT, Rusu A, et al. Can We Boost Treatment Adherence to an Online Transdiagnostic Intervention by Adding Self-Enhancement Strategies? Results From a Randomized Controlled Non-inferiority Trial. *Frontiers in psychology* 2021;12:752249. doi: 10.3389/fpsyg.2021.752249 [published Online First: 20211202]

96. Peynenburg V, Hadjistavropoulos H, Thiessen D, et al. Internet-Delivered Cognitive Behavioral Therapy for Postsecondary Students: Randomized Factorial Trial for Examining Motivational Interviewing and Booster Lessons. *Journal of medical Internet research* 2022;24(9):e40637. doi: 10.2196/40637 [published Online First: 20220907]

97. Rogers J, Sicouri G. A Single-Session Online Cognitive Bias Modification of Interpretations Modified for Adults With Anxiety and Depressive Symptoms. *Behavior therapy* 2022;53(5):967-80. doi: 10.1016/j.beth.2022.04.006 [published Online First: 20220419]

98. Schaeuffele C, Homeyer S, Perea L, et al. The unified protocol as an internet-based intervention for emotional disorders: Randomized controlled trial. *PloS One* 2022;17(7):e0270178.

99. Taylor H, Cavanagh K, Field AP, et al. Health Care Workers' Need for Headspace: Findings From a Multisite Definitive Randomized Controlled Trial of an Unguided Digital Mindfulness-Based Self-help App to Reduce Healthcare Worker Stress. *JMIR mHealth and uHealth* 2022;10(8):e31744.

100. Hosseinzadeh Asl NR. A randomized controlled trial of a mindfulness-based intervention in social workers working during the COVID-19 crisis. *Current Psychology* 2022;41(11):8192-99.

101. Kang MY, Nan JK, Yuan Y. Effectiveness of an online short-term audio-based mindfulness program on negative emotions during the COVID-19 pandemic: Latent

For peer review only - http://bmjopen.bmj.com/site/about/guidelines.xhtml

growth curve analyses of anxiety and moderated mediation effects of anxiety between mindfulness and negative affect. *Current Psychology* 2022:1-13.

102. Viravan N, Atsariyasing W, Srifuengfung M, et al. Efficacy of Online Mindfulness Program'Mindful Senses' for Depression and Anxiety Reduction in Community Samples: A Randomized Controlled Trial. *Journal of the Medical Association of Thailand* 2022;105(11)

103. Cuijpers P, Miguel C, Harrer M, et al. Cognitive behavior therapy vs. control conditions, other psychotherapies, pharmacotherapies and combined treatment for depression: a comprehensive meta‐analysis including 409 trials with 52,702 patients. *World Psychiatry* 2023;22(1):105-15.

104. Ewing DL, Monsen JJ, Thompson EJ, et al. A meta-analysis of transdiagnostic cognitive behavioural therapy in the treatment of child and young person anxiety disorders. *Behavioural and cognitive psychotherapy* 2015;43(5):562-77.

105. Munasinghe S, Page A, Mannan H, et al. Referral patterns to primary mental health services in Western Sydney (Australia): an analysis of routinely collected data (2005-2018). *Int J Ment Health Syst* 2020;14:37. doi: 10.1186/s13033-020-00368-5 [published Online First: 20200526]

106. Prina AM, Marioni RE, Hammond GC, et al. Improving access to psychological therapies and older people: findings from the Eastern Region. *Behaviour research and therapy* 2014;56(100):75-81. doi: 10.1016/j.brat.2014.03.008 [published Online First: 20140328]

107. Wittchen H-U, Mühlig S, Beesdo K. Mental disorders in primary care. *Dialogues in Clinical Neuroscience* 2003;5(2):115-28. doi: 10.31887/DCNS.2003.5.2/huwittchen

For peer review only - http://bmjopen.bmj.com/site/about/guidelines.xhtml

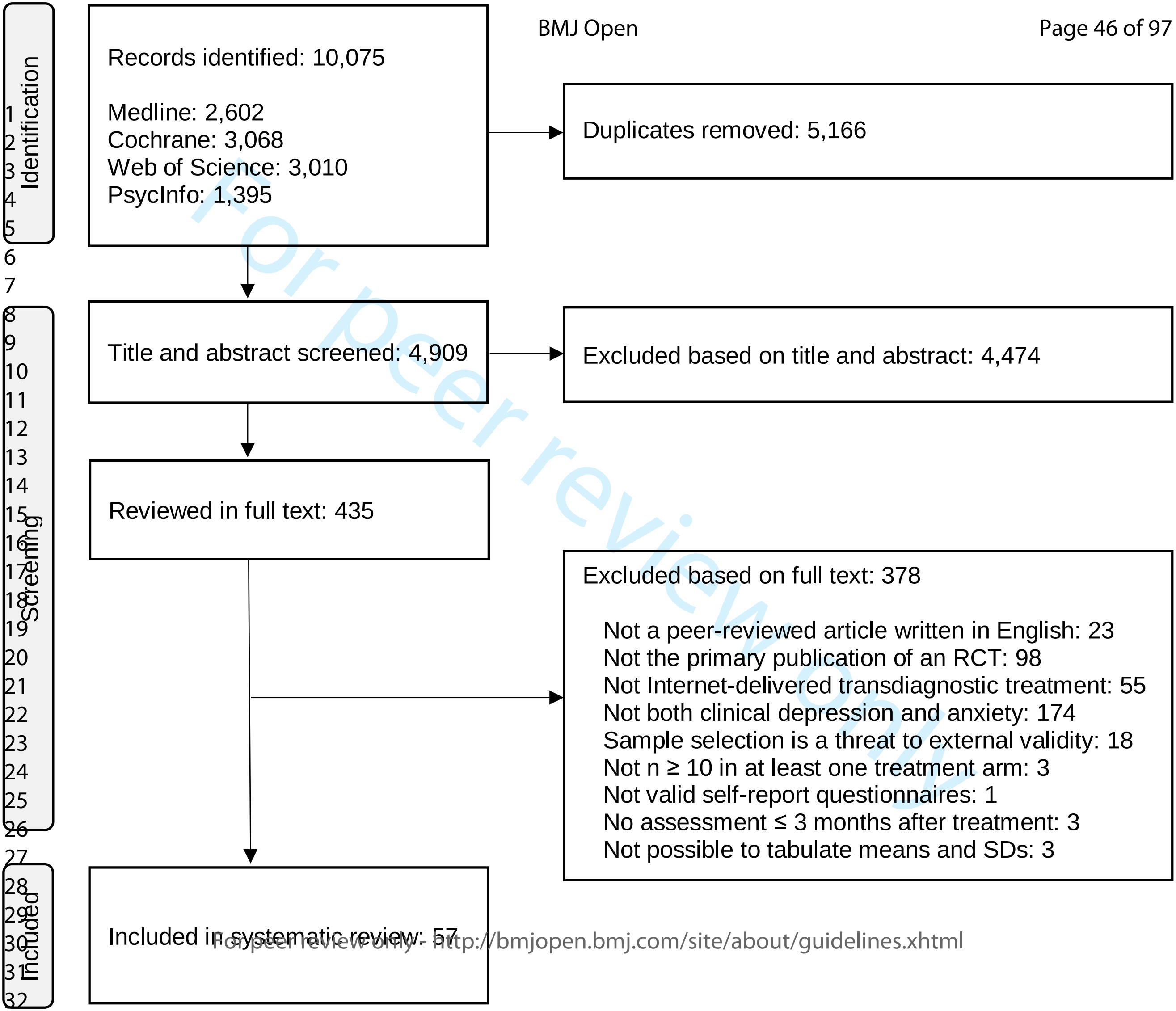

For peer review only

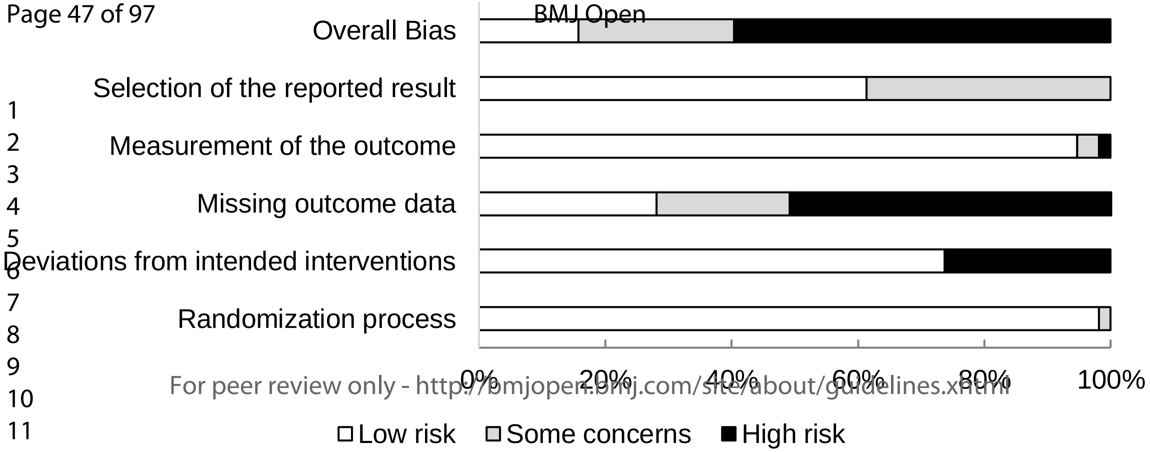

For peer review only - http://bmjopen.bmj.com/site/about/guidelines.xhtml

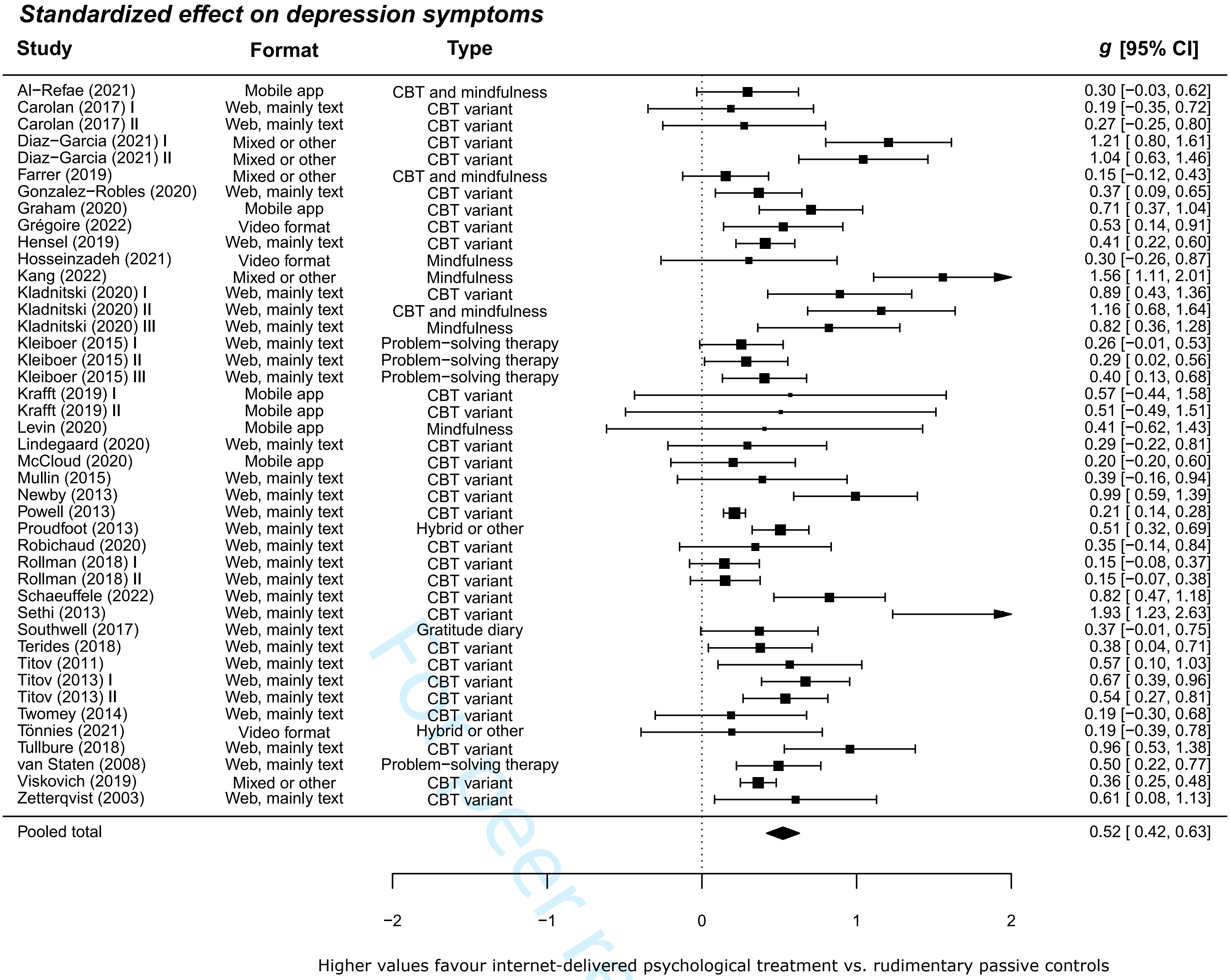

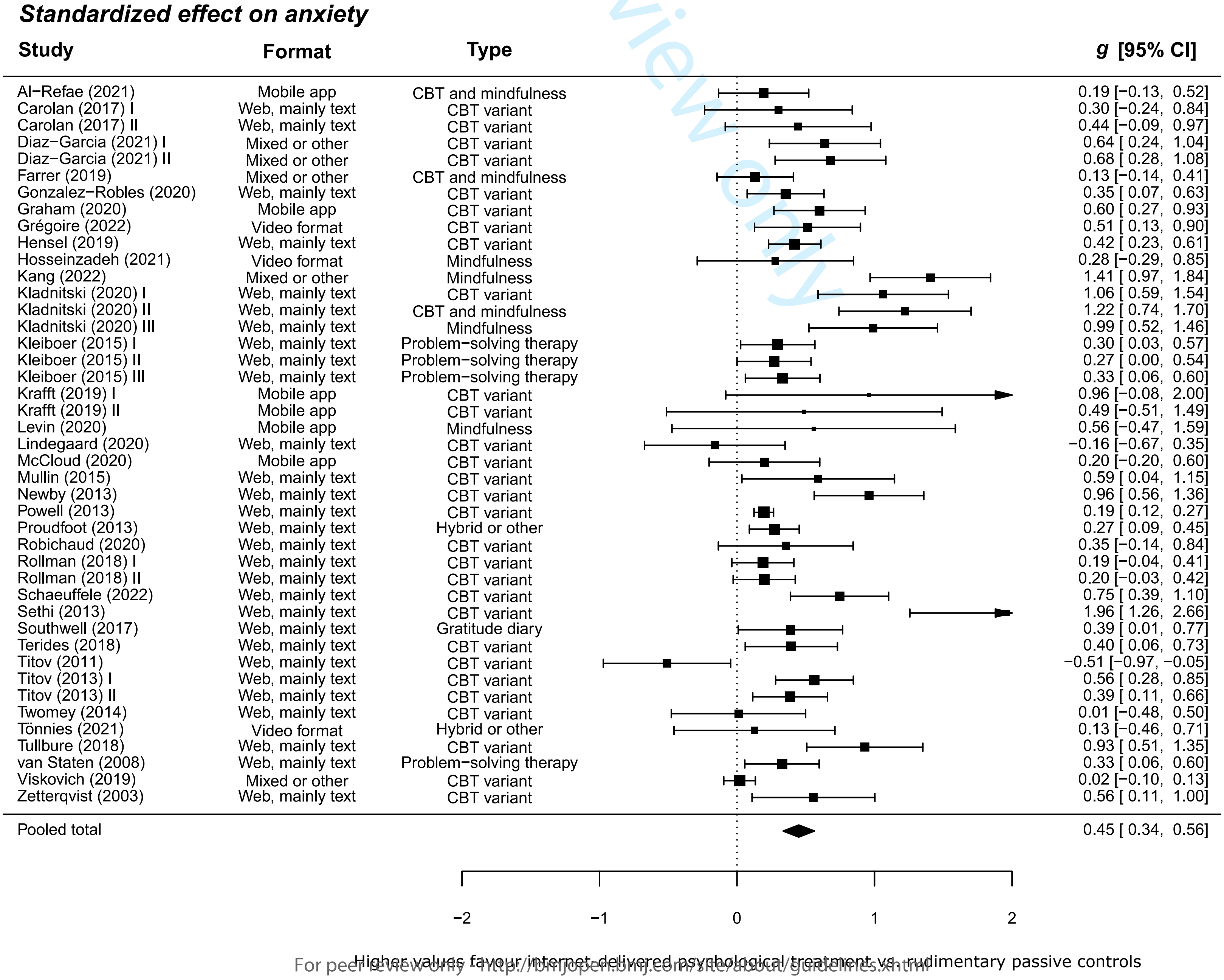

For peer review only - http://bmjopen.bmj.com/site/about/guidelines.xhtml

Standardized effect on depression symptoms

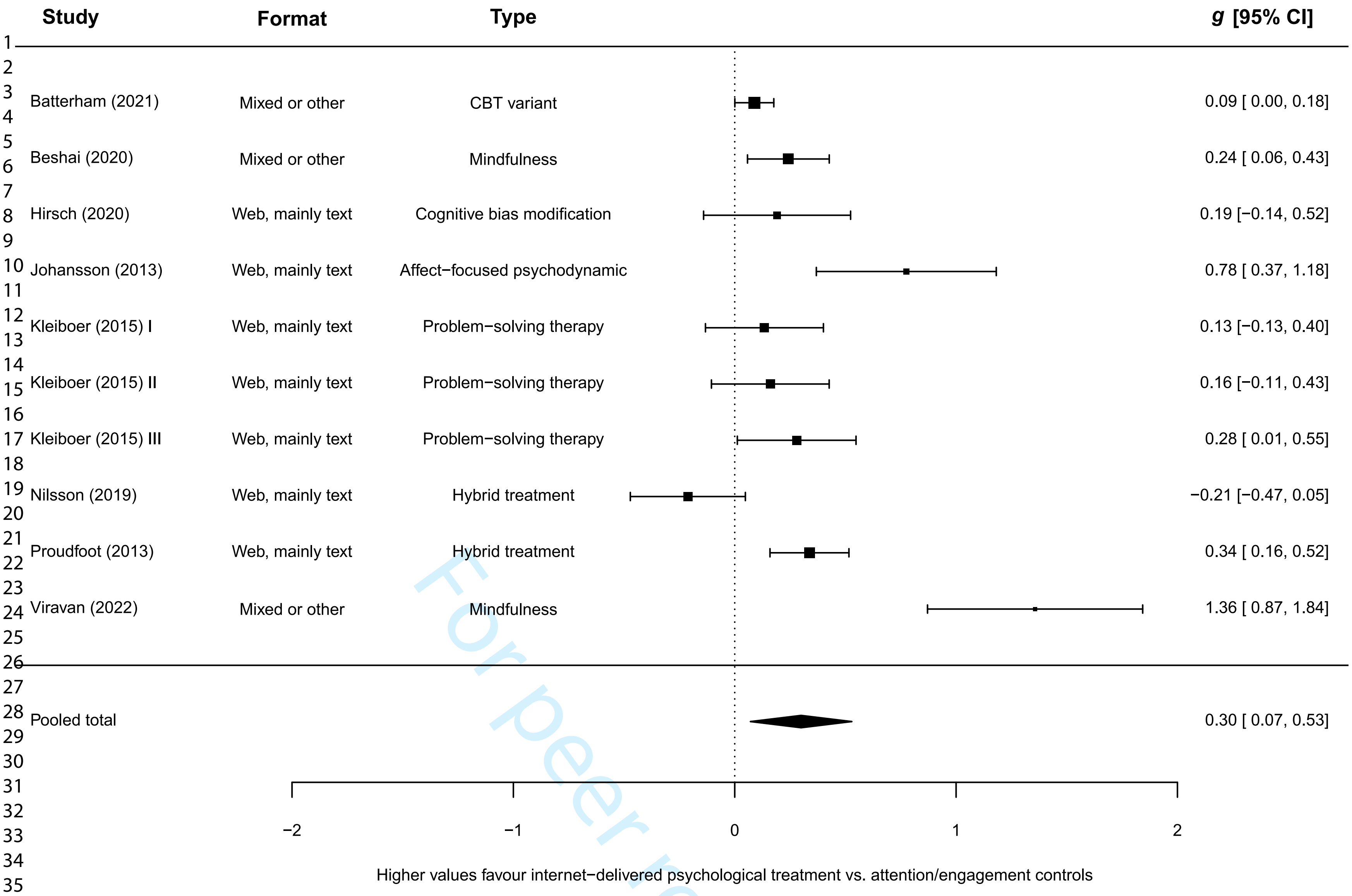

Standardized effect on anxiety

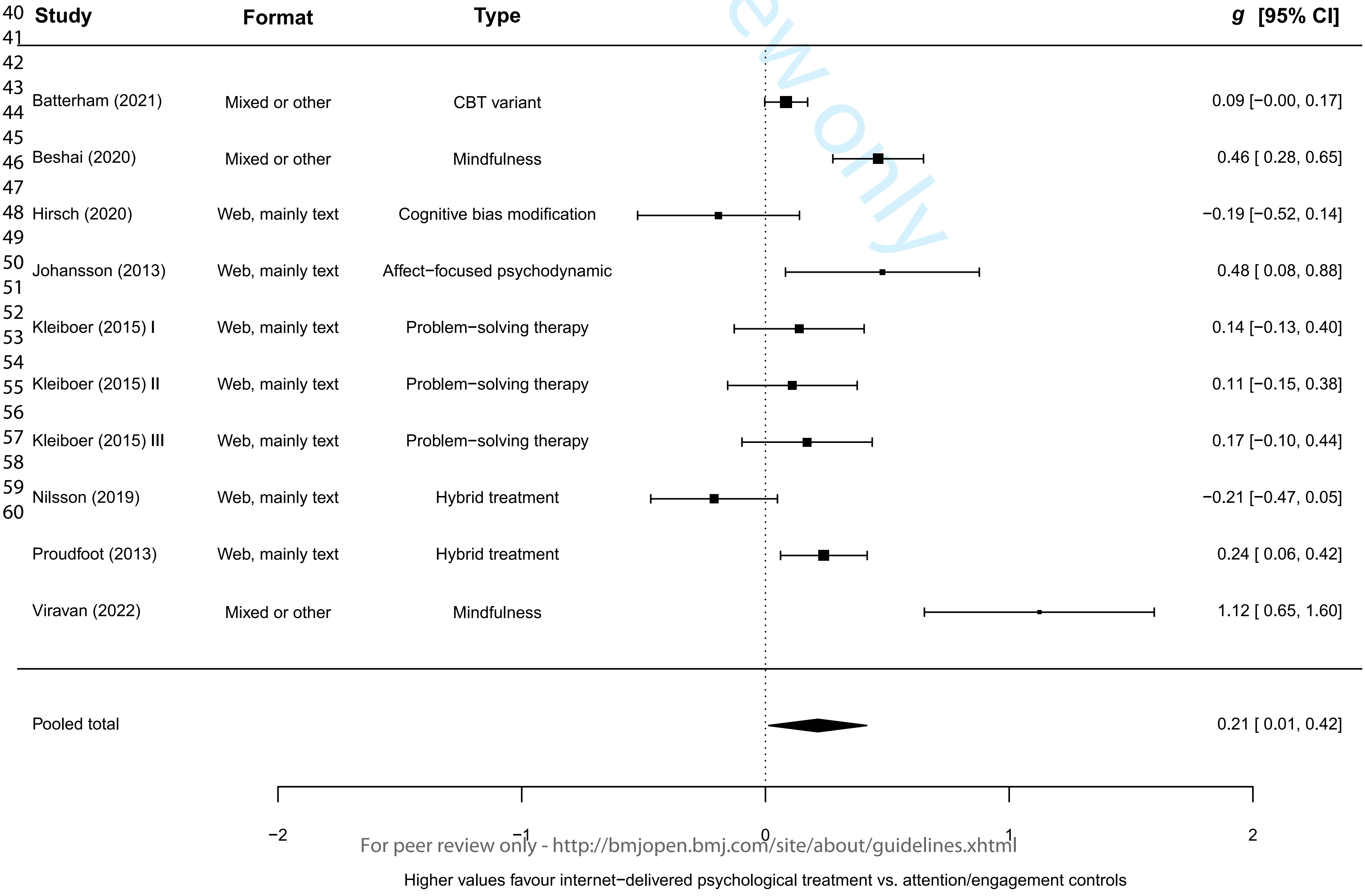

Internet-delivered transdiagnostic psychological treatments for individuals with depression, anxiety, or both

Systematic search: 2021 main search

For peer review only

Karolinska Institutet

Documentation of search strategies
University Library search consultation group

Date: May 24, 2021

Topic/research question: Systematic review and meta-analysis of transdiagnostic psychological interventions delivered via the internet for individuals suffering from common mental disorders (PROSPERO: CRD42021243172)

Name of researcher(s): Erland Axelsson

Librarian(s): Sabina Gillsund & Emma-Lotta Säätelä

Databases:

1. Medline (Ovid)
2. Cochrane Library (Wiley)
3. Web of Science Core Collection (Clarivate Analytics)
4. PsycInfo (Ovid)

Total number of hits:

- Before deduplication: 7,366
- After deduplication: 3,519

Comments:

For peer review only

Karolinska Institutet

1. Medline

Interface: Ovid MEDLINE(R) and Epub Ahead of Print, In-Process & Other Non-Indexed Citations and Daily<br><br>Date of Search: 24 May 2021<br><br>Number of hits: 1,898<br><br>Comment: In Ovid, two or more words are automatically searched as phrases; i.e. no quotation marks are needed	Field labels<br><br>• exp/ = exploded MeSH term<br>• / = non exploded MeSH term<br>• .ti,ab,kf. = title, abstract and author keywords<br>• adjx = within x words, regardless of order<br>• * = truncation of word for alternate endings

#	Searches	Results
1	exp Psychotherapy/	202021
2	(("acceptance and commitment" or affect-focused or behavio?r* or biofeedback or computeri* or cognit* or digital or "emotional awareness and expression" or educat* or exposure or guided or internet* or interpersonal or mobile or online or positive psychology or psychodynamic or psychologic* or self-guided or self-help or transdiagnost* or web*) adj2 (intervention* or therap* or treat* or program*)).ti,ab,kf.	188596
3	(behavio?ral activation or bias modification or cognitive restructuring or hypno* or meditation or mindfulness or problem solving or psychoeducation* or psychotherap* or relaxation or self-management or unified protocol).ti,ab,kf.	251506
4	or/1-3	538226
5	Anxiety Disorders/	35452
6	Agoraphobia/	2617
7	Neurotic Disorders/	17987
8	Obsessive-Compulsive Disorder/	14943
9	Panic Disorder/	7019
10	exp Phobic Disorders/	11590
11	Stress Disorders, Post-Traumatic/	34732
12	Anxiety/	87458
13	(agoraphobi* or anxi* or obsessive-compulsive or panic or phobi* or post-traumatic stress or posttraumatic stress).ti,ab,kf.	281431
14	or/5-13	336088

For peer review only - http://bmjopen.bmj.com/site/about/guidelines.xhtml

Karolinska Institutet

15	Mood Disorders/	14821
16	Depressive Disorder/	73869
17	Depressive Disorder, Major/	32106
18	Affective Symptoms/	13300
19	Depression/	127756
20	depress*.ti,ab,kf.	491024
21	((affective or mood) adj2 disorder*).ti,ab,kf.	37052
22	or/15-21	562251
23	14 and 22	140055
24	(emotional adj2 disorder*).ti,ab,kf.	3677
25	(transdiagnost* or unified protocol).ti,ab,kf.	2780
26	24 or 25	6275
27	23 or 26	144265
28	Internet/	75778
29	Internet-Based Intervention/	544
30	Therapy, Computer-Assisted/	6898
31	Telemedicine/	28197
32	Telerehabilitation/	548
33	exp Remote Consultation/	5247
34	Mobile Applications/	7799
35	Videoconferencing/	1919
36	(app or apps or cellphone* or computer* or digital or e mail or email or internet* or mobile application* or online or phone* or smartphone* or technolog* or telephone* or text message* or video* or virtual or web based or web site or website).ti,ab,kf.	1396114
37	(distance counsel?ing or distance consultation* or e consultation* or econsultation* or e counsel?ing or ecounsel?ing or e health* or ehealth* or e therapies or e therapy or etherap* or e visit* or evisit* or m health or mhealth or mobile counsel?ing or mobile consultation* or remote consultation* or remote counsel?ing or tele health* or telehealth* or tele consultation* or teleconsultation* or tele medicine or telemedicine or tele rehabilitation or telerehabilitation).ti,ab,kf.	36032
38	or/28-37	1441976
39	randomized controlled trial.pt.	531357
40	controlled clinical trial.pt.	94168
41	randomi?ed.ab.	622540

For peer review only - http://bmjopen.bmj.com/site/about/guidelines.xhtml

Karolinska Institutet

42	placebo.ab.	218143
43	clinical trials as topic.sh.	195916
44	randomly.ab.	357785
45	trial.ti.	240304
46	or/39-45	1407342
47	(systematic review or meta-analy*).ti.	219916
48	46 not 47	1340792
49	exp animals/ not humans.sh.	4832148
50	48 not 49	1230987
51	4 and 27 and 38 and 50	1921
52	limit 51 to english language	1905
53	limit 52 to yr="1995 -Current"	1898

For peer review only

2. Cochrane Library

Interface: Wiley<br>Date of Search: 24 May 2021<br>Number of hits: 2,337	Field labels<br>• ti,ab,kw = title, abstract and author keywords<br>• NEAR/x = within x words, regardless of order<br>• * = truncation of word for alternate endings

ID	Search	Hits
#1	MeSH descriptor: [Psychotherapy] explode all trees	24621
#2	(("acceptance and commitment" or "affect-focused" or behavio?r* or "biofeedback" or computeri* or cognit* or "digital" or "emotional awareness and expression" or educat* or "exposure" or "guided" or internet* or "interpersonal" or "mobile" or "online" or "positive psychology" or "psychodynamic" or psychologic* or "self-guided" or "self-help" or transdiagnost* or web*) NEAR/2 (intervention* or therap* or treat* or program*)):ti,ab,kw	81443
#3	((behavio?ral NEXT activation) or "bias modification" or "cognitive restructuring" or hypno* or "meditation" or "mindfulness" or "problem solving" or psychoeducation* or psychotherap* or "relaxation" or "self-management" or "unified protocol"):ti,ab,kw	54905
#4	#1 or #2 or #3	121709
#5	MeSH descriptor: [Anxiety Disorders] this term only	3927
#6	MeSH descriptor: [Agoraphobia] this term only	436
#7	MeSH descriptor: [Neurotic Disorders] this term only	300
#8	MeSH descriptor: [Obsessive-Compulsive Disorder] this term only	1050
#9	MeSH descriptor: [Panic Disorder] this term only	955
#10	MeSH descriptor: [Phobic Disorders] explode all trees	1382
#11	MeSH descriptor: [Stress Disorders, Post-Traumatic] this term only	2707
#12	MeSH descriptor: [Anxiety] this term only	7681
#13	(agoraphobi* or anxi* or "obsessive compulsive" or "obsessive-compulsive" or "panic" or phobi* or "post-traumatic stress" or "posttraumatic stress"):ti,ab,kw	63654
#14	#5 or #6 or #7 or #8 or #9 or #10 or #11 or #12 or #13	64273
#15	MeSH descriptor: [Mood Disorders] this term only	839
#16	MeSH descriptor: [Depressive Disorder] this term only	8011
#17	MeSH descriptor: [Depressive Disorder, Major] this term only	5111
#18	MeSH descriptor: [Affective Symptoms] this term only	459
#19	MeSH descriptor: [Depression] this term only	12730
#20	depress*:ti,ab,kw	89514
#21	(("affective" or "mood") NEAR/3 disorder*):ti,ab,kw	5404
#22	#15 or #16 or #17 or #18 or #19 or #20 or #21	91976
#23	#14 and #22	30987
#24	("emotional" NEAR/2 disorder*):ti,ab,kw	862
#25	(transdiagnost* or "unified protocol"):ti,ab,kw	730
#26	#24 or #25	1468
#27	#23 or #26	31780
#28	MeSH descriptor: [Internet] this term only	3893
#29	MeSH descriptor: [Internet-Based Intervention] this term only	182

For peer review only - http://bmjopen.bmj.com/site/about/guidelines.xhtml

Karolinska Institutet

#30	MeSH descriptor: [Therapy, Computer-Assisted] this term only	1342
#31	MeSH descriptor: [Telemedicine] this term only	2293
#32	MeSH descriptor: [Telerehabilitation] this term only	126
#33	MeSH descriptor: [Remote Consultation] explode all trees	398
#34	MeSH descriptor: [Mobile Applications] this term only	765
#35	MeSH descriptor: [Videoconferencing] this term only	190
#36	("app" or "apps" or cellphone* or computer* or "digital" or "e mail" or "email" or internet* or ("mobile" NEXT application*) or "online" or phone* or smartphone* or technolog* or telephone* or ("text" NEXT message*) or video* or "virtual" or "web based" or "web site" or "website"):ti,ab,kw	150142
#37	(("distance" NEXT "counsel?ing") or ("distance" NEXT consultation*) or ("e" NEXT consultation*) or econsultation* or ("e" NEXT "counsel?ing") or "ecounsel?ing" or ("e" NEXT health*) or ehealth* or "e therapies" or "e therapy" or etherap* or ("e" NEXT visit*) or evisit* or "m health" or "mhealth" or ("mobile" NEXT "counsel?ing") or ("mobile" NEXT consultation*) or ("remote" NEXT consultation*) or ("remote" NEXT "counsel?ing") or ("tele" NEXT health*) or telehealth* or ("tele" NEXT consultation*) or teleconsultation* or "tele medicine" or "telemedicine" or "tele rehabilitation" or "telerehabilitation"):ti,ab,kw	9310
#38	#28 or #29 or #30 or #31 or #32 or #33 or #34 or #35 or #36 or #37	152807
#39	#4 and #27 and #38	4365
#40	(clinicaltrials or trialsearch):so	364015
#41	#39 NOT #40	2416
	Limit to publication date 1995-2021 in Trials	2337

For peer review only

3. Web of Science Core Collection

Interface: Clarivate Analytics<br><br>Date of Search: 24 May 2021<br><br>Number of hits: 2,233	Field labels<br><br>• TS/Topic = title, abstract, author keywords and Keywords Plus<br>• NEAR/x = within x words, regardless of order<br>• * = truncation of word for alternate endings<br><br>Note: sometimes “quotation marks” are needed for single search terms to avoid automatic term mapping (lemmatization).

18 2,233

#14 AND #11 AND #8 AND #3

Refined by: LANGUAGES: (ENGLISH) AND [excluding] PUBLICATION YEARS: (1994 OR 1993) AND DOCUMENT TYPES: (ARTICLE OR CORRECTION OR EARLY ACCESS)

17 2,366

#14 AND #11 AND #8 AND #3

Refined by: LANGUAGES: (ENGLISH) AND [excluding] PUBLICATION YEARS: (1994 OR 1993)

16 2,370

#14 AND #11 AND #8 AND #3

Refined by: LANGUAGES: (ENGLISH)

15 2,395

#14 AND #11 AND #8 AND #3

14 916,173

#12 NOT #13

13 259,971

TI=(“systematic review” or “meta-analy*”)

12 982,134

TI=(“randomi$ed” OR “randomi$ed” OR “randomi$ation” OR “randomi$ation” OR placebo* OR (random* AND (allocat* OR assign*)) OR (blind* AND (“single” OR “double” OR “treble” OR “triple”))) OR AB=(“randomi$ed” OR “randomi$ed” OR “randomi$ation” OR “randomi$ation” OR placebo* OR (random* AND (allocat* OR assign*)) OR (blind* AND (“single” OR “double” OR “treble” OR “triple”)))

For peer review only - http://bmjopen.bmj.com/site/about/guidelines.xhtml

Karolinska Institutet

11 4,745,692

#10 OR #9

10 51,707

TS=("distance counsel$ing" or "distance consultation*" or "e consultation*" or econsultation* or "e counsel$ing" or "ecounsel$ing" or "e health*" or ehealth* or "e therapies" or "e therapy" or etherap* or "e visit*" or evisit* or "m health" or "mhealth" or "mobile counsel$ing" or "mobile consultation*" or "remote consultation*" or "remote counsel$ing" or "tele health*" or telehealth* or "tele consultation*" or teleconsultation* or "tele medicine" or "telemedicine" or "tele rehabilitation" or "telerehabilitation")

9 4,726,717

TS=("app" or "apps" or cellphone* or computer* or "digital" or "e mail" or "email" or internet* or "mobile application*" or "online" or phone* or smartphone* or technolog* or telephone* or "text message*" or video* or "virtual" or "web based" or "web site" or "website")

8 176,232

#7 OR #6

7 7,752

TS=(("emotional" NEAR/1 disorder*) or transdiagnost* or "unified protocol")

6 171,279

#5 AND #4

5 691,430

TS=(depress* or (("affective" or "mood") NEAR/1 disorder*))

4 401,339

TS=(agoraphobi* or anxi* or "obsessive-compulsive" or "panic" or phobi* or "post-traumatic stress" or "posttraumatic stress")

3 871,462

#2 OR #1

2 644,089

For peer review only - http://bmjopen.bmj.com/site/about/guidelines.xhtml

Karolinska Institutet

TS=((“behavio$ral activation” or “bias modification” or “cognitive restructuring” or hypno* or “meditation” or “mindfulness” or “problem solving” or psychoeducation* or psychotherap* or “relaxation” or “self-management” or “unified protocol”))

1 263,093

TS=((("acceptance and commitment" or “affect-focused” or behavio$r* or “biofeedback” or computeri* or cognit* or “digital” or "emotional awareness and expression" or educat* or “exposure” or “guided” or internet* or “interpersonal” or “mobile” or “online” or “positive psychology” or “psychodynamic” or psychologic* or “self-guided” or “self-help” or transdiagnost* or web*) NEAR/1 (intervention* or therap* or treat* or program*)))

For peer review only

4. Psycinfo

Interface: Ovid<br><br>Date of Search: 24 May 2021<br><br>Number of hits: 898<br><br>Comment: In Ovid, two or more words are automatically searched as phrases; i.e. no quotation marks are needed	Field labels<br><br>• exp/ = exploded controlled term<br>• / = non exploded controlled term<br>• .ti,ab,id. = title, abstract and author keywords<br>• adjx = within x words, regardless of order<br>• * = truncation of word for alternate endings

#	Searches	Results
1	exp Psychotherapy/	207730
2	exp Cognitive Therapy/	13639
3	exp Behavior Therapy/	21289
4	exp Cognitive Behavior Therapy/	23144
5	(("acceptance and commitment" or affect-focused or behavio?r* or biofeedback or computeri* or cognit* or digital or "emotional awareness and expression" or educat* or exposure or guided or internet* or interpersonal or mobile or online or positive psychology or psychodynamic or psychologic* or self-guided or self-help or transdiagnost* or web*) adj2 (intervention* or therap* or treat* or program*)).ti,ab,id.	175860
6	(behavio?ral activation or bias modification or cognitive restructuring or hypno* or meditation or mindfulness or problem solving or psychoeducation* or psychotherap* or relaxation or self-management or unified protocol).ti,ab,id.	240370
7	or/1-6	495594
8	Anxiety Disorders/	19140
9	exp Agoraphobia/	2916
10	Neurosis/	7043
11	Obsessive Compulsive Disorder/	14683
12	Panic Disorder/	7760
13	exp Phobias/	13434
14	Posttraumatic Stress Disorder/	34688
15	Anxiety/	65640
16	(agoraphobi* or anxi* or obsessive-compulsive or panic or phobi* or post-traumatic stress or posttraumatic stress).ti,ab,id.	283726

For peer review only - http://bmjopen.bmj.com/site/about/guidelines.xhtml

Karolinska Institutet

17	or/8-16	296637
18	Affective Disorders/	14669
19	exp Major Depression/	138828
20	"Depression (Emotion)"/	26043
21	depress*.ti,ab,id.	321600
22	((affective or mood) adj2 disorder*).ti,ab,id.	35856
23	or/18-22	343842
24	17 and 23	109245
25	(emotional adj2 disorder*).ti,ab,id.	5905
26	(transdiagnost* or unified protocol).ti,ab,id.	3036
27	25 or 26	8661
28	24 or 27	115823
29	Internet/	29614
30	Digital Interventions/	593
31	Computer Assisted Therapy/	1140
32	exp Telemedicine/	9675
33	Digital Technology/	719
34	Videoconferencing/	645
35	Mobile Applications/	1193
36	(app or apps or cellphone* or computer* or digital or e mail or email or internet* or mobile application* or online or phone* or smartphone* or technolog* or telephone* or text message* or video* or virtual or web based or web site or website).ti,ab,id.	436545
37	(distance counsel?ing or distance consultation* or e consultation* or econsultation* or e counsel?ing or ecounsel?ing or e health* or ehealth* or e therapies or e therapy or etherap* or e visit* or evisit* or m health or mhealth or mobile counsel?ing or mobile consultation* or remote consultation* or remote counsel?ing or tele health* or telehealth* or tele consultation* or teleconsultation* or tele medicine or telemedicine or tele rehabilitation or telerehabilitation).ti,ab,id.	7477
38	or/29-37	440321
39	exp Randomized Controlled Trials/	954
40	randomi?ed.ab.	83970
41	placebo.ab.	40594
42	randomly.ab.	76127
43	trial.ti.	32809

For peer review only - http://bmjopen.bmj.com/site/about/guidelines.xhtml

Karolinska Institutet

44	or/39-43	183930
45	(systematic review or meta-analy*).ti.	40744
46	44 not 45	176278
47	7 and 28 and 38 and 46	1037
48	limit 47 to english language	983
49	limit 48 to yr="1995 -Current"	981
50	limit 49 to "0100 journal"	898

For peer review only

For peer review only - http://bmjopen.bmj.com/site/about/guidelines.xhtml

Systematic search: 2023 updated main search

For peer review only

Documentation of search strategies
University Library search consultation group

Date: February 2023

Topic/research question: Systematic review and meta-analysis of transdiagnostic psychological interventions delivered via the internet for individuals suffering from common mental disorders (PROSPERO: CRD42021243172)

Name of researcher(s): Erland Axelsson

Librarian(s): Sabina Gillsund & Emma-Lotta Säätelä

Databases:

1. Medline (Ovid)
2. Cochrane Library (Wiley)
3. Web of Science Core Collection (Clarivate Analytics)
4. PsycInfo (EBSCO)

Total number of hits:

- Before deduplication: 10,075
- After deduplication: 4,909

1. Medline

Interface: Ovid MEDLINE(R) ALL<br><br>Date of Search: 6 February 2023<br><br>Number of hits: 2,602<br><br>Comment: In Ovid, two or more words are automatically searched as phrases; i.e. no quotation marks are needed	Field labels<br><br>• exp/ = exploded MeSH term<br>• / = non exploded MeSH term<br>• .ti,ab,kf. = title, abstract and author keywords<br>• adjx = within x words, regardless of order<br>• * = truncation of word for alternate endings

For peer review only - http://bmjopen.bmj.com/site/about/guidelines.xhtml

Karolinska Institutet

Database(s): **Ovid MEDLINE(R) ALL** 1946 to February 03, 2023
Search Strategy:

#	Searches	Results
1	exp Psychotherapy/	215915
2	(("acceptance and commitment" or affect-focused or behavio?r* or biofeedback or computeri* or cognit* or digital or "emotional awareness and expression" or educat* or exposure or guided or internet* or interpersonal or mobile or online or positive psychology or psychodynamic or psychologic* or self-guided or self-help or transdiagnost* or web*) adj2 (intervention* or therap* or treat* or program*)).ti,ab,kf.	220048
3	(behavio?ral activation or bias modification or cognitive restructuring or hypno* or meditation or mindfulness or problem solving or psychoeducation* or psychotherap* or relaxation or self-management or unified protocol).ti,ab,kf.	277967
4	or/1-3	597258
5	Anxiety Disorders/	40399
6	Agoraphobia/	2669
7	Neurotic Disorders/	18000
8	Obsessive-Compulsive Disorder/	16224
9	Panic Disorder/	7250
10	exp Phobic Disorders/	12293
11	Stress Disorders, Post-Traumatic/	40198
12	Anxiety/	103449
13	(agoraphobi* or anxi* or obsessive-compulsive or panic or phobi* or post-traumatic stress or posttraumatic stress).ti,ab,kf.	332361
14	or/5-13	389293
15	Mood Disorders/	15751
16	Depressive Disorder/	74889
17	Depressive Disorder, Major/	37299
18	Affective Symptoms/	13816
19	Depression/	146908
20	depress*.ti,ab,kf.	552903
21	((affective or mood) adj2 disorder*).ti,ab,kf.	40988
22	or/15-21	627024
23	14 and 22	168605

For peer review only - http://bmjopen.bmj.com/site/about/guidelines.xhtml

Karolinska Institutet

24	(emotional adj2 disorder*).ti,ab,kf.	4243
25	(transdiagnost* or unified protocol).ti,ab,kf.	4104
26	24 or 25	8093
27	23 or 26	174000
28	Internet/	80600
29	Internet-Based Intervention/	1071
30	Therapy, Computer-Assisted/	6968
31	Telemedicine/	36073
32	Telerehabilitation/	900
33	exp Remote Consultation/	5715
34	Mobile Applications/	10971
35	Videoconferencing/	2295
36	(app or apps or cellphone* or computer* or digital or e mail or email or internet* or mobile application* or online or phone* or smartphone* or technolog* or telephone* or text message* or video* or virtual or web based or web site or website).ti,ab,kf.	1678151
37	(distance counsel?ing or distance consultation* or e consultation* or econsultation* or e counsel?ing or ecounsel?ing or e health* or ehealth* or e therapies or e therapy or etherap* or e visit* or evisit* or m health or mhealth or mobile counsel?ing or mobile consultation* or remote consultation* or remote counsel?ing or tele health* or telehealth* or tele consultation* or teleconsultation* or tele medicine or telemedicine or tele rehabilitation or telerehabilitation).ti,ab,kf.	51873
38	or/28-37	1730206
39	randomized controlled trial.pt.	585953
40	controlled clinical trial.pt.	95177
41	randomi?ed.ab.	708985
42	placebo.ab.	235960
43	clinical trials as topic.sh.	200855
44	randomly.ab.	402423
45	trial.ti.	279911
46	or/39-45	1550232
47	(systematic review or meta-analy*).ti.	290755
48	46 not 47	1464360
49	exp animals/ not humans.sh.	5089811
50	48 not 49	1343292

Karolinska Institutet

51	4 and 27 and 38 and 50	2627
52	limit 51 to english language	2609
53	limit 52 to yr="1995 -Current"	2602

For peer review only

For peer review only - http://bmjopen.bmj.com/site/about/guidelines.xhtml

Karolinska Institutet

2. Cochrane Library

Interface: Wiley<br><br>Date of Search: 3 February 2023<br><br>Number of hits: 3,068	Field labels<br><br>• ti,ab,kw = title, abstract and author keywords<br>• NEAR/x = within x words, regardless of order<br>• * = truncation of word for alternate endings

ID	Search	Hits
#1	MeSH descriptor: [Psychotherapy] explode all trees	30769
#2	(("acceptance and commitment" or "affect-focused" or behavio?r* or "biofeedback" or computeri* or cognit* or "digital" or "emotional awareness and expression" or educat* or "exposure" or "guided" or internet* or "interpersonal" or "mobile" or "online" or "positive psychology" or "psychodynamic" or psychologic* or "self-guided" or "self-help" or transdiagnost* or web*) NEAR/2 (intervention* or therap* or treat* or program*)):ti,ab,kw	95851
#3	((behavio?ral NEXT activation) or "bias modification" or "cognitive restructuring" or hypno* or "meditation" or mindfulness or "problem solving" or psychoeducation* or psychotherap* or "relaxation" or "self-management" or "unified protocol"):ti,ab,kw	64668
#4	#1 or #2 or #3	143208
#5	MeSH descriptor: [Anxiety Disorders] this term only	5112
#6	MeSH descriptor: [Agoraphobia] this term only	472
#7	MeSH descriptor: [Neurotic Disorders] this term only	316
#8	MeSH descriptor: [Obsessive-Compulsive Disorder] this term only	1250
#9	MeSH descriptor: [Panic Disorder] this term only	1053
#10	MeSH descriptor: [Phobic Disorders] explode all trees	1942
#11	MeSH descriptor: [Stress Disorders, Post-Traumatic] this term only	3561
#12	MeSH descriptor: [Anxiety] this term only	9845
#13	(agoraphobi* or anxi* or "obsessive compulsive" or "obsessive-compulsive" or "panic" or phobi* or "post-traumatic stress" or "posttraumatic stress"):ti,ab,kw	76993
#14	#5 or #6 or #7 or #8 or #9 or #10 or #11 or #12 or #13	77700
#15	MeSH descriptor: [Mood Disorders] this term only	1004
#16	MeSH descriptor: [Depressive Disorder] this term only	9053
#17	MeSH descriptor: [Depressive Disorder, Major] this term only	6360
#18	MeSH descriptor: [Affective Symptoms] this term only	514
#19	MeSH descriptor: [Depression] this term only	15880
#20	depress*:ti,ab,kw	103690
#21	(("affective" or "mood") NEAR/3 disorder*):ti,ab,kw	6072
#22	#15 or #16 or #17 or #18 or #19 or #20 or #21	106436
#23	#14 and #22	38854
#24	("emotional" NEAR/2 disorder*):ti,ab,kw	1098
#25	(transdiagnost* or "unified protocol"):ti,ab,kw	1061
#26	#24 or #25	1974
#27	#23 or #26	39878

For peer review only - http://bmjopen.bmj.com/site/about/guidelines.xhtml

#28	MeSH descriptor: [Internet] this term only	4780
#29	MeSH descriptor: [Internet-Based Intervention] this term only	527
#30	MeSH descriptor: [Therapy, Computer-Assisted] this term only	1476
#31	MeSH descriptor: [Telemedicine] this term only	3338
#32	MeSH descriptor: [Telerehabilitation] this term only	229
#33	MeSH descriptor: [Remote Consultation] 3 tree(s) exploded	440
#34	MeSH descriptor: [Mobile Applications] this term only	1476
#35	MeSH descriptor: [Videoconferencing] this term only	259
#36	("app" or "apps" or cellphone* or computer* or "digital" or "e mail" or "email" or internet* or (mobile NEXT application*) or "online" or phone* or smartphone* or technolog* or telephone* or (text NEXT message*) or video* or "virtual" or "web based" or "web site" or "website"):ti,ab,kw	184801
#37	(("distance" NEXT counsel?ing) or ("distance" NEXT consultation*) or ("e" NEXT consultation*) or econsultation* or ("e" NEXT counsel?ing) or ecounsel?ing or ("e" NEXT health*) or ehealth* or "e therapies" or "e therapy" or etherap* or ("e" NEXT visit*) or evisit* or "m health" or "mhealth" or ("mobile" NEXT counsel?ing) or ("mobile" NEXT consultation*) or ("remote" NEXT consultation*) or ("remote" NEXT counsel?ing) or ("tele" NEXT health*) or telehealth* or ("tele" NEXT consultation*) or teleconsultation* or "tele medicine" or "telemedicine" or "tele rehabilitation" or "telerehabilitation"):ti,ab,kw	12716
#38	#28 or #29 or #30 or #31 or #32 or #33 or #34 or #35 or #36 or #37	188389
#39	#4 and #27 and #38	6085
#40	(clinicaltrials or trialsearch):so	444610
#41	#39 NOT #40	3156
	Limit to publication date 1995-2023 in Trials	3068

For peer review only - http://bmjopen.bmj.com/site/about/guidelines.xhtml

Karolinska Institutet

3. Web of Science Core Collection

Interface: Clarivate Analytics<br><br>Date of Search: 3 February 2023<br><br>Number of hits: 3,010	Field labels<br><br>• TS/Topic = title, abstract, author keywords and Keywords Plus<br>• NEAR/x = within x words, regardless of order<br>• * = truncation of word for alternate endings<br><br>Note: sometimes “quotation marks” are needed for single search terms to avoid automatic term mapping (lemmatization).

18 #14 AND #11 AND #8 AND #3

Refined by: LANGUAGES: (ENGLISH) AND [excluding] PUBLICATION YEARS: (1994 OR 1993) AND DOCUMENT TYPES: (ARTICLE OR CORRECTION OR EARLY ACCESS)

17 #14 AND #11 AND #8 AND #3

Refined by: LANGUAGES: (ENGLISH) AND [excluding] PUBLICATION YEARS: (1994 OR 1993)

16 #14 AND #11 AND #8 AND #3

Refined by: LANGUAGES: (ENGLISH)

15 #14 AND #11 AND #8 AND #3

14 #12 NOT #13

13 TI=(“systematic review” or “meta-analy*”)

12 TI=(“randomi$ed” OR “randomi$ed” OR “randomi$ation” OR “randomi$ation” OR placebo* OR (random* AND (allocat* OR assign*)) OR (blind* AND (“single” OR “double” OR “treble” OR “triple”))) OR AB=(“randomi$ed” OR “randomi$ed” OR “randomi$ation” OR “randomi$ation” OR placebo* OR (random* AND (allocat* OR assign*)) OR (blind* AND (“single” OR “double” OR “treble” OR “triple”)))

11 #10 OR #9

10 TS=(“distance counsel$ing” or “distance consultation*” or “e consultation*” or econsultation* or “e counsel$ing” or “ecounsel$ing” or “e health*” or ehealth* or “e therapies” or “e therapy” or etherap* or “e visit*” or evisit* or “m health” or “mhealth” or “mobile counsel$ing” or “mobile consultation*” or “remote consultation*” or “remote counsel$ing” or “tele health*” or telehealth* or “tele consultation*” or teleconsultation* or “tele medicine” or “telemedicine” or “tele rehabilitation” or “telerehabilitation")

For peer review only - http://bmjopen.bmj.com/site/about/guidelines.xhtml

9 TS=("app" or "apps" or cellphone* or computer* or "digital" or "e mail" or "email" or internet* or "mobile application*" or "online" or phone* or smartphone* or technolog* or telephone* or "text message*" or video* or "virtual" or "web based" or "web site" or "website")

8 #7 OR #6

7 TS=(("emotional" NEAR/1 disorder*) or transdiagnost* or "unified protocol")

6 #5 AND #4

5 TS=(depress* or (("affective" or "mood") NEAR/1 disorder*))

4 TS=(agoraphobi* or anxi* or "obsessive-compulsive" or "panic" or phobi* or "post-traumatic stress" or "posttraumatic stress")

3 #2 OR #1

2 TS=(("behavio$ral activation" or "bias modification" or "cognitive restructuring" or hypno* or "meditation" or "mindfulness" or "problem solving" or psychoeducation* or psychotherap* or "relaxation" or "self-management" or "unified protocol"))

1 TS=((("acceptance and commitment" or "affect-focused" or behavio$r* or "biofeedback" or computeri* or cognit* or "digital" or "emotional awareness and expression" or educat* or "exposure" or "guided" or internet* or "interpersonal" or "mobile" or "online" or "positive psychology" or "psychodynamic" or psychologic* or "self-guided" or "self-help" or transdiagnost* or web*) NEAR/1 (intervention* or therap* or treat* or program*)))

For peer review only - http://bmjopen.bmj.com/site/about/guidelines.xhtml

Karolinska Institutet

4. Psycinfo

Interface: EBSCO<br><br>Date of Search: 6 february 2023<br><br>Number of hits: 1,395	Field labels<br><br>• DE = subject heading<br>• TI = title<br>• AB = abstract<br>• KW = author keywords<br>• Nx = within x words, regardless of order<br>• * = truncation of word for alternate endings

S47	S45 NOT S36<br><br>Publication year: 1995-2023<br>Language: - english	1,395
S45	S44 NOT S36	1,418
S44	S7 AND S32 AND S35 AND S43	1,572
S43	S39 OR S42	135,063
S42	S40 AND S41	125,758
S41	S17 OR S18 OR S19 OR S20 OR S21	375,079
S40	S8 OR S9 OR S10 OR S11 OR S12 OR S13 OR S14 OR S15 OR S16	332,986
S39	S37 OR S38	12,089
S38	TI (transdiagnost* or "unified protocol") OR AB (transdiagnost* or "unified protocol") OR KW (transdiagnost* or "unified protocol")	4,261
S37	TI emotional N2 disorder* OR AB emotional N2 disorder* OR KW emotional N2 disorder*	8,225
S36	TI "systematic review" or meta-analy*	50,479
S35	S33 OR S34	206,233
S34	AB randomi?ed OR AB placebo OR AB randomly OR TI trial	206,178
S33	DE "Randomized Controlled Trials" OR DE "Randomized Clinical Trials"	1,387
S32	S23 OR S24 OR S25 OR S26 OR S27 OR S28 OR S29 OR S30 OR S31	504,430
S31	TI (“distance counsel#ing” or “distance consultation*” or “e consultation*” or econsultation* or “e counsel#ing” or “ecounsel#ing” or “e health*” or ehealth* or “e therapies” or “e therapy” or etherap* or “e visit*” or evisit* or “m health” or “mhealth” or “mobile counsel#ing” or “mobile consultation*” or “remote consultation*” or “remote counsel#ing” or “tele health*” or telehealth* or “tele consultation*” or teleconsultation* or “tele medicine” or “telemedicine” or “tele rehabilitation” or “telerehabilitation") OR AB (“distance counsel#ing” or “distance consultation*” or “e consultation*” or econsultation* or “e counsel#ing” or “ecounsel#ing” or “e health*” or ehealth* or “e therapies” or “e therapy” or etherap* or “e visit*” or evisit* or “m health” or “mhealth” or “mobile counsel#ing” or “mobile consultation*” or “remote consultation*” or “remote counsel#ing” or “tele health*” or telehealth* or “tele consultation*” or teleconsultation* or “tele medicine” or “telemedicine” or “tele rehabilitation” or “telerehabilitation") OR KW (“distance counsel#ing” or “distance consultation*” or “e consultation*” or econsultation* or “e counsel#ing” or “ecounsel#ing” or “e health*” or ehealth* or “e therapies” or “e therapy” or etherap* or “e visit*” or evisit* or “m health” or “mhealth” or “mobile counsel#ing” or “mobile consultation*” or “remote consultation*” or “remote counsel#ing” or “tele health*” or telehealth* or “tele consultation*” or teleconsultation* or “tele medicine” or “telemedicine” or “tele rehabilitation” or “telerehabilitation")	10,426
S30	TI (app or apps or cellphone* or computer* or digital or “e mail” or email or internet* or “mobile application*” or online or phone* or smartphone* or technolog* or telephone* or “text message*” or video* or virtual or “web based” or “web site” or website) OR AB (app or apps	498,437

For peer review only - http://bmjopen.bmj.com/site/about/guidelines.xhtml

Karolinska Institutet

	or cellphone* or computer* or digital or “e mail” or email or internet* or “mobile application*” or online or phone* or smartphone* or technolog* or telephone* or “text message*” or video* or virtual or “web based” or “web site” or website) OR KW (app or apps or cellphone* or computer* or digital or “e mail” or email or internet* or “mobile application*” or online or phone* or smartphone* or technolog* or telephone* or “text message*” or video* or virtual or “web based” or “web site” or website)	
S29	DE "Mobile Applications"	2,881
S28	DE "Videoconferencing"	1,138
S27	DE "Digital Technology"	1,488
S26	DE "Telemedicine" OR DE "Online Therapy" OR DE "Teleconferencing" OR DE "Teleconsultation" OR DE "Telepsychiatry" OR DE "Telepsychology" OR DE "Telerehabilitation" OR DE "Teleconferencing" OR DE "Videoconferencing"	13,791
S25	DE "Computer Assisted Therapy"	1,211
S24	DE "Digital Interventions"	1,160
S23	DE "Internet"	38,434
S22	S17 OR S18 OR S19 OR S20 OR S21	375,079
S21	TI (((affective or mood) N2 disorder*)) OR AB (((affective or mood) N2 disorder*)) OR KW (((affective or mood) N2 disorder*))	41,246
S20	TI depress* OR AB depress* OR KW depress*	349,996
S19	DE "Depression (Emotion)"	26,821
S18	DE "Major Depression" OR DE "Anaclitic Depression" OR DE "Dysthymic Disorder" OR DE "Endogenous Depression" OR DE "Late Life Depression" OR DE "Postpartum Depression" OR DE "Reactive Depression" OR DE "Recurrent Depression" OR DE "Treatment Resistant Depression"	154,908
S17	DE "Affective Disorders"	15,668
S16	TI (agoraphobi* or anxi* or obsessive-compulsive or panic or phobi* or “post-traumatic stress” or “posttraumatic stress”)) OR AB (agoraphobi* or anxi* or obsessive-compulsive or panic or phobi* or “post-traumatic stress” or “posttraumatic stress”)) OR KW (agoraphobi* or anxi* or obsessive-compulsive or panic or phobi* or “post-traumatic stress” or “posttraumatic stress”))	310,917
S15	DE "Anxiety"	94,782
S14	DE "Posttraumatic Stress Disorder"	38,806
S13	DE "Phobias" OR DE "Acrophobia" OR DE "Agoraphobia" OR DE "Claustrophobia" OR DE "Ophidiophobia" OR DE "School Phobia" OR DE "Social Phobia"	14,824
S12	DE "Panic Disorder"	9,312
S11	DE "Obsessive Compulsive Disorder"	15,724
S10	DE "Neurosis"	7,143
S9	DE "Agoraphobia"	3,756
S8	DE "Anxiety Disorders"	34,816
S7	S1 OR S2 OR S3 OR S4 OR S5 OR S6	579,216
S6	TI (“behavio#ral activation” or “bias modification” or “cognitive restructuring” or hypno* or meditation or mindfulness or “problem solving” or psychoeducation* or psychotherap* or relaxation or self-management or “unified protocol”) OR AB (“behavio#ral activation” or “bias modification” or “cognitive restructuring” or hypno* or meditation or mindfulness or “problem solving” or psychoeducation* or psychotherap* or relaxation or self-management or “unified protocol”) OR KW (“behavio#ral activation” or “bias modification” or “cognitive restructuring” or hypno* or meditation or mindfulness or “problem solving” or psychoeducation* or psychotherap* or relaxation or self-management or “unified protocol”)	256,300
S5	TI ((("acceptance and commitment" or affect-focused or behavio#r* or biofeedback or computeri* or cognit* or digital or "emotional awareness and expression" or educat* or exposure or guided or internet* or interpersonal or mobile or online or positive psychology or psychodynamic or psychologic* or self-guided or self-help or transdiagnost* or web*) N2	229,986

For peer review only

For peer review only - http://bmjopen.bmj.com/site/about/guidelines.xhtml

Karolinska Institutet

	(intervention* or therap* or treat* or program*))) OR AB ((("acceptance and commitment" or affect-focused or behavio#r* or biofeedback or computeri* or cognit* or digital or "emotional awareness and expression" or educat* or exposure or guided or internet* or interpersonal or mobile or online or positive psychology or psychodynamic or psychologic* or self-guided or self-help or transdiagnost* or web*) N2 (intervention* or therap* or treat* or program*))) OR KW ((("acceptance and commitment" or affect-focused or behavio#r* or biofeedback or computeri* or cognit* or digital or "emotional awareness and expression" or educat* or exposure or guided or internet* or interpersonal or mobile or online or positive psychology or psychodynamic or psychologic* or self-guided or self-help or transdiagnost* or web*) N2 (intervention* or therap* or treat* or program*)))	
S4	DE "Cognitive Behavior Therapy" OR DE "Acceptance and Commitment Therapy" OR DE "Cognitive Processing Therapy" OR DE "Prolonged Exposure Therapy"	26,182
S3	DE "Behavior Therapy" OR DE "Aversion Therapy" OR DE "Conversion Therapy" OR DE "Dialectical Behavior Therapy" OR DE "Exposure Therapy" OR DE "Implosive Therapy" OR DE "Reciprocal Inhibition Therapy" OR DE "Response Cost" OR DE "Systematic Desensitization Therapy" OR DE "Aversion Therapy" OR DE "Covert Sensitization" OR DE "Exposure Therapy" OR DE "Imaginal Exposure" OR DE "Implosive Therapy" OR DE "In Vivo Exposure" OR DE "Prolonged Exposure Therapy" OR DE "Systematic Desensitization Therapy" OR DE "Virtual Reality Exposure Therapy"	34,636
S2	DE "Cognitive Therapy"	24,241
S1	DE "Psychotherapy" OR DE "Adlerian Psychotherapy" OR DE "Adolescent Psychotherapy" OR DE "Affirmative Therapy" OR DE "Analytical Psychotherapy" OR DE "Autogenic Training" OR DE "Brief Psychotherapy" OR DE "Brief Relational Therapy" OR DE "Child Psychotherapy" OR DE "Client Centered Therapy" OR DE "Conversion Therapy" OR DE "Couples Therapy" OR DE "Eclectic Psychotherapy" OR DE "Emotion Focused Therapy" OR DE "Existential Therapy" OR DE "Experiential Psychotherapy" OR DE "Expressive Psychotherapy" OR DE "Eye Movement Desensitization Therapy" OR DE "Feminist Therapy" OR DE "Geriatric Psychotherapy" OR DE "Gestalt Therapy" OR DE "Group Psychotherapy" OR DE "Guided Imagery" OR DE "Humanistic Psychotherapy" OR DE "Hypnotherapy" OR DE "Individual Psychotherapy" OR DE "Insight Therapy" OR DE "Integrative Psychotherapy" OR DE "Interpersonal Psychotherapy" OR DE "Logotherapy" OR DE "Narrative Therapy" OR DE "Network Therapy" OR DE "Persuasion Therapy" OR DE "Primal Therapy" OR DE "Psychoanalysis" OR DE "Psychodrama" OR DE "Psychodynamic Psychotherapy" OR DE "Psychotherapeutic Counseling" OR DE "Psychotherapeutic Techniques" OR DE "Rational Emotive Behavior Therapy" OR DE "Reality Therapy" OR DE "Relationship Therapy" OR DE "Solution Focused Therapy" OR DE "Strategic Therapy" OR DE "Supportive Psychotherapy" OR DE "Transactional Analysis" OR DE "Adolescent Psychotherapy" OR DE "Multisystemic Therapy" OR DE "Child Psychotherapy" OR DE "Play Therapy" OR DE "Gestalt Therapy" OR DE "Empty Chair Technique" OR DE "Group Psychotherapy" OR DE "Encounter Group Therapy" OR DE "Therapeutic Community" OR DE "Humanistic Psychotherapy" OR DE "Client Centered Therapy" OR DE "Hypnotherapy" OR DE "Age Regression (Hypnotic)" OR DE "Ericksonian Psychotherapy" OR DE "Posthypnotic Suggestions" OR DE "Integrative Psychotherapy" OR DE "Schema Therapy" OR DE "Psychoanalysis" OR DE "Adlerian Psychotherapy" OR DE "Brief Relational Therapy" OR DE "Dream Analysis" OR DE "Self-Analysis" OR DE "Psychotherapeutic Counseling" OR DE "Family Therapy" OR DE "Psychotherapeutic Techniques" OR DE "Active Listening" OR DE "Animal Assisted Therapy" OR DE "Autogenic Training" OR DE "Brief Relational Therapy" OR DE "Centering" OR DE "Cotherapy" OR DE "Dream Analysis" OR DE "Empty Chair Technique" OR DE "Ericksonian Psychotherapy" OR DE "Free Association" OR DE "Guided Imagery" OR DE "Life Review" OR DE "Mirroring" OR DE "Morita Therapy" OR DE "Motivational Interviewing" OR DE "Mutual Storytelling Technique" OR DE "Network Therapy" OR DE "Paradoxical Techniques" OR DE "Psychodrama" OR DE "Strategic Therapy" OR DE "Strategic Family Therapy"	237,062

For peer review only - http://bmjopen.bmj.com/site/about/guidelines.xhtml

Internet-delivered transdiagnostic psychological treatments for individuals with depression, anxiety, or both: a systematic review with meta-analysis of randomized controlled trials

Randomized controlled trials per year of publication

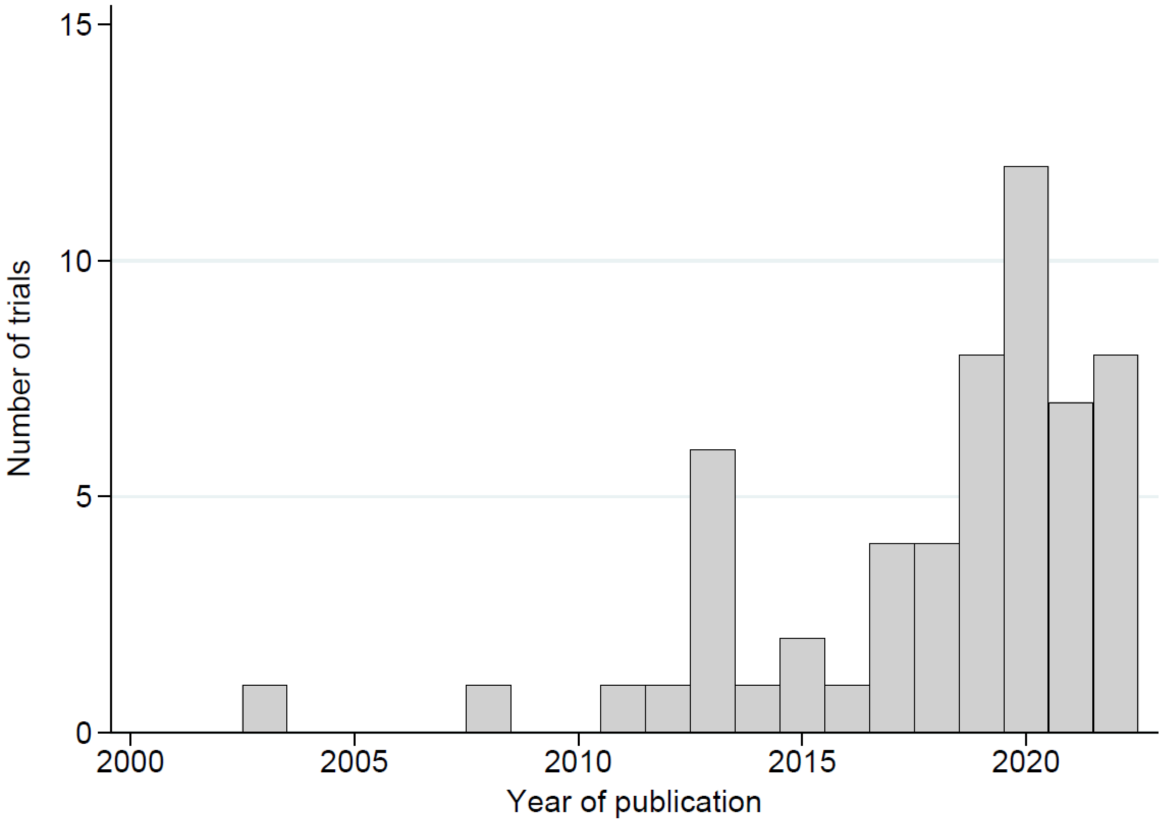

Internet-delivered transdiagnostic psychological treatments for individuals with depression, anxiety, or both: a systematic review with meta-analysis of randomized controlled trials

Exclusion criteria discussed with regard to disagreements

There were 45 disagreements:

- 18 concerned criterion d, i.e., whether the study recruited a mix of participants suffering from clinically significant depression and anxiety
- 15 concerned criterion c, i.e., whether the study evaluated the efficacy of an internet-delivered transdiagnostic psychological treatment for anxiety and depression
- 6 concerned criterion e, i.e., whether the sample composition was such that it was unreasonable to generalize to typical patients with depression and anxiety disorders
- 4 concerned criterion b, i.e., whether the text under consideration was a primary publication from a randomized controlled trial
- 1 concerned criterion a, i.e., whether the text under consideration was a peer reviewed article written in English
- 1 concerned criterion f, i.e., whether there was at least 10 participants in at least one internet-delivered transdiagnostic psychological treatment arm

For peer review only

Internet-delivered transdiagnostic psychological treatments for individuals with depression, anxiety, or both: a systematic review with meta-analysis of randomized controlled trials

Trial characteristics

Table S.B.1. Trial characteristics including references

Reference	Year	Country	Age, M	Women, %	N	Duration	Format	Treatment type/school	Missing, %
Al-Refae, et al. [1]	2021	Canada	25	79	245	< 6 weeks	app	mixed CBT/mindfulness	41
Batterham, et al. [2]	2021	Australia		85	1986	< 6 weeks	mixed/other	CBT	66
Beshai, et al. [3]	2020	Canada	35	44	456	< 6 weeks	mixed/other	mindfulness based	65
Borjalilu, et al. [4]	2019	Iran	24	71	68	≥ 6 weeks	app	mindfulness based	
Carolan, et al. [5]	2017	United Kingdom	41	85	84	≥ 6 weeks	website	CBT	26
Dear, et al. [6]	2018	Australia	21	82	217	< 6 weeks	website	CBT	28
Diaz-Garcia, et al. [7]	2021	Spain	34	72	216	≥ 6 weeks	mixed/other	CBT	32
Farrer, et al. [8]	2019	Australia	22	78	200	≥ 6 weeks	mixed/other	mixed CBT/mindfulness	28
Fitzpatrick, et al. [9]	2017	United States	22	67	70	< 6 weeks	mixed/other	CBT	20
Gonzalez-Robles, et al. [10]	2020	Spain	38	69	214	≥ 6 weeks	website	CBT	39
Graham, et al. [11]	2020	United States	42	82	146	≥ 6 weeks	app	CBT	4
Gregoire, et al. [12]	2022	Canada	26	77	107	< 6 weeks	video	CBT	31
Hadjistavropoulos, et al. [13]	2017	Canada	38	79	182	≥ 6 weeks	website	CBT	25
Hadjistavropoulos, et al. [14]	2020a	Canada	37	76	744	≥ 6 weeks	website	CBT	31
Hadjistavropoulos, et al. [15]	2020b	Canada	37	72	631	≥ 6 weeks	website	CBT	35
Hadjistavropoulos, et al. [16]	2022	Canada	36	76	469	≥ 6 weeks	website	CBT	27
Hensel, et al. [17]	2019	Canada	41	73, 78	812	≥ 6 weeks	website	CBT	45
Hirsch, et al. [18]	2020	United Kingdom	29	83	178	< 6 weeks	website	other	12
Hoek, et al. [19]	2012	The Netherlands	16	76	45	< 6 weeks	website	other	38
Hosseinzadeh Asl [20]	2022	Turkey	33	55	59	< 6 weeks	video	mindfulness based	17
Isbăşoiu, et al. [21]	2021	Romania	33	85	284	≥ 6 weeks	website	CBT	45

For peer review only

Internet-delivered transdiagnostic psychological treatments for individuals with depression, anxiety, or both: a systematic review with meta-analysis of randomized controlled trials

Johansson, et al. [22]	2013	Sweden	45	82	100	≥ 6 weeks	website	other	0
Kang, et al. [23]	2022	China	37	53	100	< 6 weeks	mixed/other	mindfulness based	0
Kladnitski, et al. [24]	2020	Australia	39	86	158	≥ 6 weeks	website	mixed CBT/mindfulness	27
Kleiboer, et al. [25]	2015	The Netherlands	45	65	537	< 6 weeks	website	other	22
Krafft, et al. [26]	2019	United States	25	66	35	< 6 weeks	app	CBT	60
Levin, et al. [27]	2022	United States	20	100	23	< 6 weeks	app	mindfulness based	30
Lindegaard, et al. [28]	2021	Sweden	38	42	59	≥ 6 weeks	website	CBT	39
McCloud, et al. [29]	2020	United Kingdom	24	83	168	≥ 6 weeks	app	CBT	42
Mohr, et al. [30]	2019	United States	36	76	301	≥ 6 weeks	app	CBT	3
Mullin, et al. [31]	2015	Australia	28	64	55	≥ 6 weeks	website	CBT	24
Newby, et al. [32]	2013	Australia	44	78	109	≥ 6 weeks	website	CBT	12
Nilsson, et al. [33]	2019	Sweden			837	≥ 6 weeks	website	other	67
Osborn, et al. [34]	2020	Kenya	15	64	103	< 6 weeks	website	other	0
Peynenburg, et al. [35]	2022	Canada	24	81	308	< 6 weeks	website	CBT	29
Powell, et al. [36]	2012	United Kingdom	41	78	3070	≥ 6 weeks	website	CBT	42
Proudfoot, et al. [37]	2013	Australia	39	70	720	≥ 6 weeks	website	other	28
Robichaud, et al. [38]	2020	Canada	35	87	63	≥ 6 weeks	website	CBT	32
Rogers and Sicouri [39]	2022	Australia	20	55	45	< 6 weeks	website	other	3
Rollman, et al. [40]	2018	United States	43	80	704	≥ 6 weeks	website	CBT	14
Schaeuffele, et al. [41]	2022	Germany	37	68	132	≥ 6 weeks	website	CBT	25
Sethi [42]	2013	Australia	20	58	89	< 6 weeks	website	CBT	0
Soucy, et al. [43]	2021	Canada	38	75	480	≥ 6 weeks	website	CBT	30
Southwell and Gould [44]	2016	Australia	34	88	120	< 6 weeks	website	other	52
Taylor, et al. [45]	2022	United Kingdom	41	83	2182	< 6 weeks	app	mindfulness based	35
Terides, et al. [46]	2018	Australia	45	88, 75	148	≥ 6 weeks	website	CBT	18
Titov, et al. [47]	2011	Australia	44	73	77	≥ 6 weeks	website	CBT	10

For peer review only

Internet-delivered transdiagnostic psychological treatments for individuals with depression, anxiety, or both: a systematic review with meta-analysis of randomized controlled trials

Titov, et al. [48]	2013	Australia	41	74	274	≥ 6 weeks	website	CBT	20
Titov, et al. [49]	2016	Australia	66	64	459	≥ 6 weeks	website	CBT	9
Tönnies, et al. [50]	2021	Germany	49	70	50	≥ 6 weeks	video	other	10
Tulbure, et al. [51]	2018	Romania	34	81	105	≥ 6 weeks	website	CBT	8
Twomey, et al. [52]	2014	Ireland	35	74	201	< 6 weeks	website	CBT	67
van Straten, et al. [53]	2008	The Netherlands	45	71	213	< 6 weeks	website	other	17
Villemaire-Krajden and Myhr [54]	2019	Canada	38	67	67	≥ 6 weeks	mixed/other	CBT	31
Viravan, et al. [55]	2022	Thailand	34	32	80	< 6 weeks	mixed/other	mindfulness based	4
Viskovich and Pakenham [56]	2020	Australia	27	68	2110	< 6 weeks	mixed/other	CBT	68
Zetterqvist, et al. [57]	2003	Sweden	39	57, 65	100	≥ 6 weeks	website	CBT	37

For peer review only

Internet-delivered transdiagnostic psychological treatments for individuals with depression, anxiety, or both: a systematic review with meta-analysis of randomized controlled trials

Publication bias

We explored indicators of publication bias in the meta-analysis of effects versus rudimentary passive controls. For depression, the funnel plot (see below) appeared to indicate that small- to medium-sized trials with non-beneficial effects of internet-delivered psychological treatments were underreported. This asymmetry was confirmed by Egger's test ($z = 2.24$, $p = 0.025$). The trim-and-fill procedure resulted in different outcomes depending on the choice of estimator. With the R0 estimator ($p < 0.001$), 19 studies were imputed, and the between-group effect dropped from 0.52 to 0.29 (95% CI 0.16-0.42). In contrast, the L0 and Q0 estimators resulted in no imputation. Visual inspection of the funnel plot for anxiety (see below) was also indicative of possible asymmetry in the same direction, but this was more ambiguous. Egger's test was significant ($z = 2.64$, $p = 0.008$) but the trim-and-fill procedure did not result in imputation, regardless of estimator. As is recommended in high heterogeneity situations,[58] we repeated all tests of publication bias in a manner informed by the moderator analyses and results were similar (see below).

For peer review only

Internet-delivered transdiagnostic psychological treatments for individuals with depression, anxiety, or both: a systematic review with meta-analysis of randomized controlled trials

Meta-analysis versus rudimentary passive controls, with depression symptoms as outcome

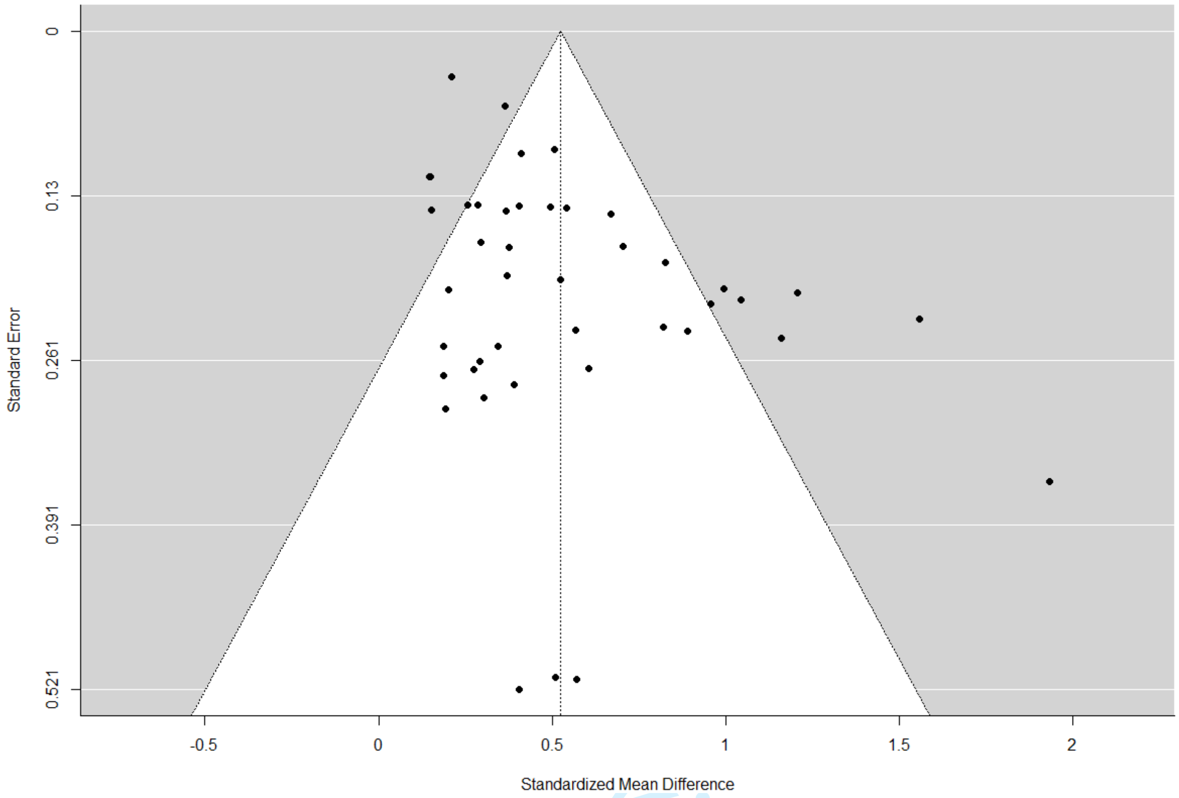

For peer review only

Internet-delivered transdiagnostic psychological treatments for individuals with depression, anxiety, or both: a systematic review with meta-analysis of randomized controlled trials

Meta-analysis versus rudimentary passive controls, with depression symptoms as outcome, with missing trials imputed based on the trim-and-fill procedure and the R0 estimator

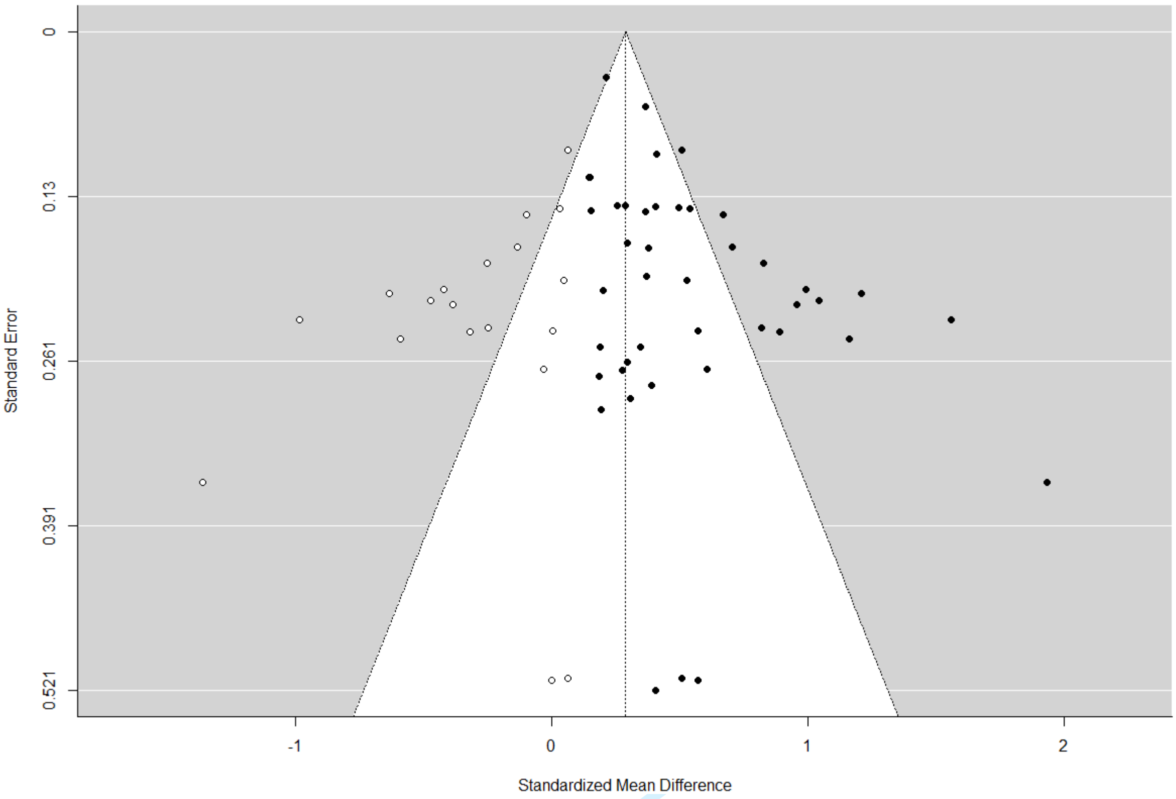

Internet-delivered transdiagnostic psychological treatments for individuals with depression, anxiety, or both: a systematic review with meta-analysis of randomized controlled trials

Meta-analysis versus rudimentary passive controls, with anxiety as outcome

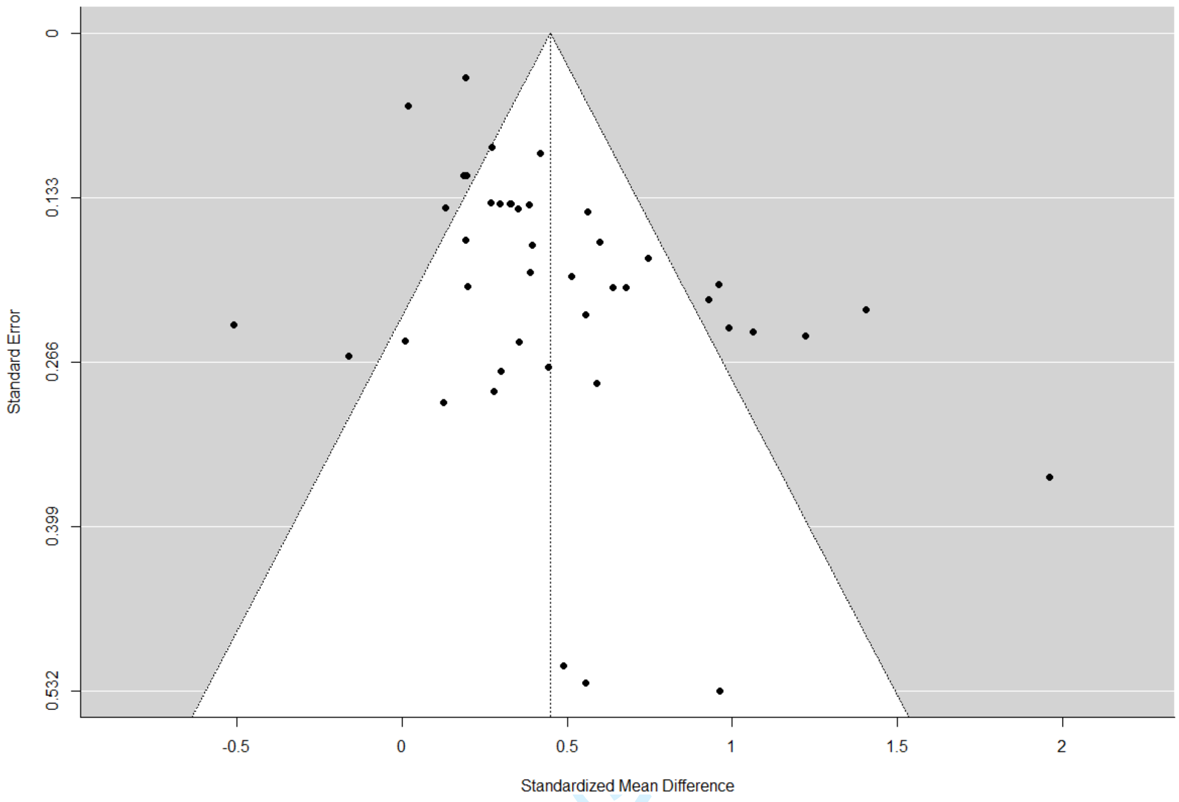

Internet-delivered transdiagnostic psychological treatments for individuals with depression, anxiety, or both: a systematic review with meta-analysis of randomized controlled trials

Risk of bias re-assessed separately based on whether trials required participants to meet full criteria for formal psychiatric diagnosis or not

Asymmetry of the funnel plot may result from several mechanisms. Because heterogeneity in the main analyses was substantial, and the moderator analyses indicated that the only significant moderator of the between-group effect versus rudimentary passive controls was whether trials required all participants to meet full criteria for a formal diagnosis or not, we repeated our investigation of the funnel plot for these substrata of the trials. That is, we first assessed whether publication bias appeared to be present in trials that employed psychiatric diagnoses, and then assessed whether publication bias appeared to be present in trials that did not employ psychiatric diagnoses. The results are tabulated in the table below. Overall, even when the sample of trials was split along this only significant effect moderator (i.e., psychiatric diagnoses or not), the outcome of the analyses of publication bias was still contradictory and similar to the equivalent tests of all trials.

Table S.B.2. Sensitivity analyses pertaining to publication bias

**Subset of trials**	**Outcome**	**Visual inspection**	**Egger's test**	**Trim-and-fill**
Psychiatric diagnosis (k = 12)	Depression	Possibly slight bias, but not clear	z = 3.67, p < 0.001	R0: p = 0.250, but 1 trial imputed<br>L0: no imputation<br>Q0: no imputation
Psychiatric diagnosis (k = 12)	Anxiety	No clear sign of bias	z = 2.08, p = 0.038	R0: p = 0.500, no imputation<br>L0: no imputation<br>Q0: no imputation
Cut-off or symptoms (k = 31)	Depression	Possibly slight bias, but not clear	z = 0.82, p = 0.410	R0: p = 0.004, and 7 trials imputed<br>L0: no imputation<br>Q0: no imputation
Cut-off or symptoms (k = 31)	Anxiety	Possibly slight bias, but not clear	z = 2.31, p = 0.021	R0: p = 0.500, no imputation<br>L0: 9 trials imputed<br>Q0: 12 trials imputed

For peer review only

Internet-delivered transdiagnostic psychological treatments for individuals with depression, anxiety, or both: a systematic review with meta-analysis of randomized controlled trials

Non-planned sensitivity analyses of psychiatric diagnosis as an effect moderator

One moderator was found to be significant for both depression and anxiety symptoms: trials that required all participants to meet full criteria for a psychiatric diagnosis reported a larger pooled between-group effects versus rudimentary passive controls, as compared to trials that did not have this requirement. We conducted a non-planned sensitivity analysis to ensure that this effect was not driven merely by participants in control groups of the routine clinic being more likely to be enrolled other treatments, by testing the moderating effect of diagnosis on the pooled within-group effect. This test illustrated that psychiatric diagnosis remained a significant effect moderator also with regard to within-group effects (depression: 1.10 vs. 0.75, $p = 0.001$; anxiety: 1.14 vs. 0.63, $p < 0.001$). We also reviewed studies that used the most common depression and anxiety outcome respectively (the PHQ-9 and GAD-7) to determine if the effect of diagnosis was driven primarily by a difference in the mean symptom reduction, or by a smaller standard deviation. Though the average standard deviation was slightly smaller in studies that required participants to meet full criteria for a diagnosis, the reduction in the mean was also larger. In summary, requiring participants to meet full criteria for psychiatric diagnosis appeared to be robust effect moderator.

For peer review only

Internet-delivered transdiagnostic psychological treatments for individuals with depression, anxiety, or both: a systematic review with meta-analysis of randomized controlled trials

Randomized controlled trials versus other bona fide treatments

Two trials compared the effects of an internet-delivered transdiagnostic psychological treatment to those of a distinctly different bona fide treatment. One trial compared mindfulness-based stress reduction delivered via a mobile app (n = 28) to a face-to-face equivalent (n = 20) for university students and reported non-significant between-group effects (depression: g = -0.49; anxiety: g = -0.13).[4] One trial compared web-based CBT (n = 23) to a face-to-face equivalent (n = 21) for university students and reported a significant and large between-group effect on depression in favor of the face-to-face treatment (g = -1.39) and a non-significant and small between-group effect on anxiety (g = -0.21).[42] Last, we also identified one trial that was on the verge of being a bona-fide comparison. In this trial, web-based CBT (n = 33) was compared to a condition which was described as a waitlist, but where participants had access to an unguided CBT bibliotherapy which we deemed to be relatively credible (n = 34). There were no significant between-group effects on depression (g = 0.33) or anxiety (g = -0.13).[54]

For peer review only

Internet-delivered transdiagnostic psychological treatments for individuals with depression, anxiety, or both: a systematic review with meta-analysis of randomized controlled trials

Randomized factorial trials

Nine randomized factorial trials explored moderators of the overall effect of internet-delivered transdiagnostic psychological treatments. Seven of these trials evaluated the same treatment protocol: the Wellbeing Course. The factors investigated, and the main outcome of each of the nine randomized factorial trials is tabulated below:

Table S.B.3. Randomized factorial trials of internet-delivered transdiagnostic psychological treatments suitable for both depression and anxiety

Reference	N	Protocol	Factors	Summary of main outcome
Dear *et al.* (2018)[6]	217	Wellbeing Course	Therapist-guided vs. unguided	Mostly no significant mean differences in the clinical outcome, with small between-group effects on depression and anxiety at post-treatment.
Hadjistavropoulos *et al.* (2017)[13]	182	Wellbeing Course	Weekly support vs. optional support	No significant mean differences in the clinical outcome, with small between-group effects on depression and anxiety at post-treatment. Rates of treatment completion were significantly higher with weekly support.
Hadjistavropoulos *et al.* (2020a)[14]	744	Wellbeing Course	Weekly support by ICBT specialist vs. weekly support plus on-demand support by ICBT specialist vs. weekly support by other clinician	Mostly no significant mean differences in the clinical outcome, with small between-group effects on depression and anxiety at post-treatment. Rates of response in depression were lower with support from other clinicians.
Hadjistavropoulos *et al.* (2020b)[15]	631	Wellbeing Course	Use of homework reflection vs. no use of homework reflection<br><br>Weekly support vs. twice-weekly support<br><br>ICBT clinic vs. community mental health clinic	No significant mean differences in the clinical outcome, with small between-group effects on depression and anxiety at post-treatment.
Hadjistavropoulos *et al.* (2022)[16]	469	Wellbeing Course	Optional 4-week extension of support vs. no such option<br><br>Optional booster lesson vs. no such option	No significant mean differences in the clinical outcome, with small between-group effects on depression and anxiety at post-treatment. About 52% opted to make use of the booster lesson.

For peer review only

Internet-delivered transdiagnostic psychological treatments for individuals with depression, anxiety, or both: a systematic review with meta-analysis of randomized controlled trials

Isbăşoiu *et al.* (2021)[21]	284	Unified protocol	Addition of self-enhancement and emotion regulation components vs. no such components	No significant mean differences in the clinical outcome, with small between-group effects on depression and anxiety at post-treatment.
Mohr *et al.* (2019)[30]	301	IntelliCare	Therapist-guided vs. unguided * <br>Weekly app recommendations vs. no such recommendations *	Guidance led to a significantly larger mean effect on anxiety (but not depression). Recommendations led to a significantly larger mean effect on depression (but not anxiety). Between-group effects were small.
Peynenburg *et al.* (2022)[35]	308	Wellbeing Course	Internet-delivered exercises based on motivational interviewing before treatment vs. no such exercises * <br>Optional booster lesson vs. no such option	Internet-delivered exercises based on motivational interviewing before treatment led to a significantly larger mean effect on depression and anxiety. Having the option of a booster lesson did not lead to significantly larger mean effects. Between-group effects were small. About 32% opted to make use of the booster lesson.
Soucy *et al.* (2021)[43]	480	Wellbeing Course	Internet-delivered exercises based on motivational interviewing before treatment vs. no such exercises	No significant mean differences in the clinical outcome, with small between-group effects on depression and anxiety at post-treatment.

* This factor was a significant moderator of the effect on depression symptoms or anxiety.

For peer review only

Internet-delivered transdiagnostic psychological treatments for individuals with depression, anxiety, or both: a systematic review with meta-analysis of randomized controlled trials

Brief interventions

Two trials evaluated brief interventions (≤2 weeks) for adults. One trial (*N*=70) found that 2 weeks of communication with an automated chatbot trained in active listening and CBT strategies was associated with a small to moderate between-group effect on depression (g = 0.37) but no significant between-group effect on anxiety as compared to a waitlist.[9] The second trial (*N*=45) reported null effects of a single session of cognitive bias modification on depression (g = -0.04) and anxiety (g = 0.42), compared to a psychological placebo.[39]

For peer review only

Internet-delivered transdiagnostic psychological treatments for individuals with depression, anxiety, or both: a systematic review with meta-analysis of randomized controlled trials

References

1. Al-Refae M, Al-Refae A, Munroe M, et al. A Self-Compassion and Mindfulness-Based Cognitive Mobile Intervention (Serene) for Depression, Anxiety, and Stress: Promoting Adaptive Emotional Regulation and Wisdom. *Front Psychol* 2021;12:648087. doi: 10.3389/fpsyg.2021.648087 [published Online First: 2021/04/09]
2. Batterham PJ, Calear AL, Farrer L, et al. Efficacy of a Transdiagnostic Self-Help Internet Intervention for Reducing Depression, Anxiety, and Suicidal Ideation in Adults: Randomized Controlled Trial. *J Med Internet Res* 2021;23(1):e22698. doi: 10.2196/22698 [published Online First: 2021/01/23]
3. Beshai S, Bueno C, Yu M, et al. Examining the effectiveness of an online program to cultivate mindfulness and self-compassion skills (Mind-OP): Randomized controlled trial on Amazon's Mechanical Turk. *Behav Res Ther* 2020;134:103724. doi: 10.1016/j.brat.2020.103724 [published Online First: 2020/09/18]
4. Borjalilu S, Mazaheri MA, Talebpour A. Effectiveness of Mindfulness-Based Stress Management in The Mental Health of Iranian University Students: A Comparison of Blended Therapy, Face-to-Face Sessions, and mHealth App (Aramgar). *Iranian Journal of Psychiatry and Behavioral Sciences* 2019;13(2) doi: 10.5812/ijpbs.84726
5. Carolan S, Harris PR, Greenwood K, et al. Increasing engagement with an occupational digital stress management program through the use of an online facilitated discussion group: Results of a pilot randomised controlled trial. *Internet Interv* 2017;10:1-11. doi: 10.1016/j.invent.2017.08.001 [published Online First: 2018/08/24]
6. Dear BF, Fogliati VJ, Fogliati R, et al. Treating anxiety and depression in young adults: A randomised controlled trial comparing clinician-guided versus self-guided Internet-delivered cognitive behavioural therapy. *Aust N Z J Psychiatry* 2018;52(7):668-79. doi: 10.1177/0004867417738055 [published Online First: 2017/10/25]
7. Diaz-Garcia A, Gonzalez-Robles A, Garcia-Palacios A, et al. Negative and Positive Affect Regulation in a Transdiagnostic Internet-Based Protocol for Emotional Disorders: Randomized Controlled Trial. *J Med Internet Res* 2021;23(2):e21335. doi: 10.2196/21335 [published Online First: 2021/02/02]
8. Farrer LM, Gulliver A, Katruss N, et al. A novel multi-component online intervention to improve the mental health of university students: Randomised controlled trial of the Uni Virtual Clinic. *Internet Interv* 2019;18:100276. doi: 10.1016/j.invent.2019.100276 [published Online First: 2020/01/01]
9. Fitzpatrick KK, Darcy A, Vierhile M. Delivering Cognitive Behavior Therapy to Young Adults With Symptoms of Depression and Anxiety Using a Fully Automated Conversational Agent (Woebot): A Randomized Controlled Trial. *JMIR Ment Health* 2017;4(2):e19. doi: 10.2196/mental.7785 [published Online First: 2017/06/08]
10. Gonzalez-Robles A, Diaz-Garcia A, Garcia-Palacios A, et al. Effectiveness of a Transdiagnostic Guided Internet-Delivered Protocol for Emotional Disorders Versus Treatment as Usual in Specialized Care: Randomized Controlled Trial. *J Med Internet Res* 2020;22(7):e18220. doi: 10.2196/18220 [published Online First: 2020/07/17]
11. Graham AK, Greene CJ, Kwasny MJ, et al. Coached Mobile App Platform for the Treatment of Depression and Anxiety Among Primary Care Patients: A Randomized Clinical Trial. *JAMA Psychiatry* 2020;77(9):906-14. doi: 10.1001/jamapsychiatry.2020.1011 [published Online First: 2020/05/21]
12. Gregoire S, Beaulieu F, Lachance L, et al. An online peer support program to improve mental health among university students: A randomized controlled trial. *J Am Coll*

Internet-delivered transdiagnostic psychological treatments for individuals with depression, anxiety, or both: a systematic review with meta-analysis of randomized controlled trials

Health 2022:1-13. doi: 10.1080/07448481.2022.2099224 [published Online First: 2022/08/10]

13. Hadjistavropoulos HD, Schneider LH, Edmonds M, et al. Randomized controlled trial of internet-delivered cognitive behaviour therapy comparing standard weekly versus optional weekly therapist support. *J Anxiety Disord* 2017;52:15-24. doi: 10.1016/j.janxdis.2017.09.006 [published Online First: 2017/10/02]
14. Hadjistavropoulos HD, Peynenburg V, Nugent M, et al. Transdiagnostic Internet-delivered cognitive behaviour therapy with therapist support offered once-weekly or once-weekly supplemented with therapist support within one-business-day: Pragmatic randomized controlled trial. *Internet Interv* 2020;22:100347. doi: 10.1016/j.invent.2020.100347 [published Online First: 20200831]
15. Hadjistavropoulos HD, Peynenburg V, Thiessen DL, et al. A pragmatic factorial randomized controlled trial of transdiagnostic internet-delivered cognitive behavioural therapy: Exploring benefits of homework reflection questionnaires and twice-weekly therapist support. *Internet Interv* 2020;22:100357. doi: 10.1016/j.invent.2020.100357 [published Online First: 20201201]
16. Hadjistavropoulos HD, Peynenburg V, Thiessen DL, et al. A randomized factorial trial of internet-delivered cognitive behavioural therapy: An 8-week program with or without extended support and booster lesson. *Internet Interv* 2022;27:100499. doi: 10.1016/j.invent.2022.100499 [published Online First: 20220206]
17. Hensel JM, Shaw J, Ivers NM, et al. A Web-Based Mental Health Platform for Individuals Seeking Specialized Mental Health Care Services: Multicenter Pragmatic Randomized Controlled Trial. *J Med Internet Res* 2019;21(6):e10838. doi: 10.2196/10838 [published Online First: 2019/06/06]
18. Hirsch CR, Krahe C, Whyte J, et al. Effects of modifying interpretation bias on transdiagnostic repetitive negative thinking. *J Consult Clin Psychol* 2020;88(3):226-39. doi: 10.1037/ccp0000455 [published Online First: 2020/02/19]
19. Hoek W, Schuurmans J, Koot HM, et al. Effects of Internet-based guided self-help problem-solving therapy for adolescents with depression and anxiety: a randomized controlled trial. *PLoS One* 2012;7(8):e43485. doi: 10.1371/journal.pone.0043485 [published Online First: 2012/09/07]
20. Hosseinzadeh Asl NR. A randomized controlled trial of a mindfulness-based intervention in social workers working during the COVID-19 crisis. *Curr Psychol* 2022;41(11):8192-99. doi: 10.1007/s12144-021-02150-3 [published Online First: 2021/08/17]
21. Isbăşoiu AB, Tulbure BT, Rusu A, et al. Can We Boost Treatment Adherence to an Online Transdiagnostic Intervention by Adding Self-Enhancement Strategies? Results From a Randomized Controlled Non-inferiority Trial. *Frontiers in psychology* 2021;12:752249. doi: 10.3389/fpsyg.2021.752249 [published Online First: 20211202]
22. Johansson R, Bjorklund M, Hornborg C, et al. Affect-focused psychodynamic psychotherapy for depression and anxiety through the Internet: a randomized controlled trial. *PeerJ* 2013;1:e102. doi: 10.7717/peerj.102 [published Online First: 2013/07/19]
23. Kang MY, Nan JKM, Yuan Y. Effectiveness of an online short-term audio-based mindfulness program on negative emotions during the COVID-19 pandemic: Latent growth curve analyses of anxiety and moderated mediation effects of anxiety between mindfulness and negative affect. *Curr Psychol* 2022:1-13. doi: 10.1007/s12144-022-03902-5 [published Online First: 2022/12/06]

For peer review only

Internet-delivered transdiagnostic psychological treatments for individuals with depression, anxiety, or both: a systematic review with meta-analysis of randomized controlled trials

24. Kladnitski N, Smith J, Uppal S, et al. Transdiagnostic internet-delivered CBT and mindfulness-based treatment for depression and anxiety: A randomised controlled trial. *Internet Interv* 2020;20:100310. doi: 10.1016/j.invent.2020.100310 [published Online First: 2020/03/07]
25. Kleiboer A, Donker T, Seekles W, et al. A randomized controlled trial on the role of support in Internet-based problem solving therapy for depression and anxiety. *Behav Res Ther* 2015;72:63-71. doi: 10.1016/j.brat.2015.06.013 [published Online First: 2015/07/21]
26. Krafft J, Potts S, Schoendorff B, et al. A Randomized Controlled Trial of Multiple Versions of an Acceptance and Commitment Therapy Matrix App for Well-Being. *Behav Modif* 2019;43(2):246-72. doi: 10.1177/0145445517748561 [published Online First: 2017/12/22]
27. Levin ME, Hicks ET, Krafft J. Pilot evaluation of the stop, breathe & think mindfulness app for student clients on a college counseling center waitlist. *J Am Coll Health* 2022;70(1):165-73. doi: 10.1080/07448481.2020.1728281 [published Online First: 2020/03/10]
28. Lindegaard T, Seaton F, Halaj A, et al. Internet-based cognitive behavioural therapy for depression and anxiety among Arabic-speaking individuals in Sweden: a pilot randomized controlled trial. *Cogn Behav Ther* 2021;50(1):47-66. doi: 10.1080/16506073.2020.1771414 [published Online First: 2020/07/01]
29. McCloud T, Jones R, Lewis G, et al. Effectiveness of a Mobile App Intervention for Anxiety and Depression Symptoms in University Students: Randomized Controlled Trial. *JMIR Mhealth Uhealth* 2020;8(7):e15418. doi: 10.2196/15418 [published Online First: 2020/08/01]
30. Mohr DC, Schueller SM, Tomasino KN, et al. Comparison of the Effects of Coaching and Receipt of App Recommendations on Depression, Anxiety, and Engagement in the IntelliCare Platform: Factorial Randomized Controlled Trial. *Journal of medical Internet research* 2019;21(8):e13609. doi: 10.2196/13609 [published Online First: 20190828]
31. Mullin A, Dear BF, Karin E, et al. The UniWellbeing course: A randomised controlled trial of a transdiagnostic internet-delivered cognitive behavioural therapy (CBT) programme for university students with symptoms of anxiety and depression. *Internet Interventions* 2015;2(2):128-36. doi: 10.1016/j.invent.2015.02.002
32. Newby JM, Mackenzie A, Williams AD, et al. Internet cognitive behavioural therapy for mixed anxiety and depression: a randomized controlled trial and evidence of effectiveness in primary care. *Psychol Med* 2013;43(12):2635. doi: 10.1017/S0033291713000111
33. Nilsson A, Sorman K, Klingvall J, et al. MyCompass in a Swedish context - lessons learned from the transfer of a self-guided intervention targeting mental health problems. *BMC Psychiatry* 2019;19(1):51. doi: 10.1186/s12888-019-2039-1 [published Online First: 2019/02/02]
34. Osborn TL, Rodriguez M, Wasil AR, et al. Single-session digital intervention for adolescent depression, anxiety, and well-being: Outcomes of a randomized controlled trial with Kenyan adolescents. *J Consult Clin Psychol* 2020;88(7):657-68. doi: 10.1037/ccp0000505 [published Online First: 2020/05/12]
35. Peynenburg V, Hadjistavropoulos H, Thiessen D, et al. Internet-Delivered Cognitive Behavioral Therapy for Postsecondary Students: Randomized Factorial Trial for Examining Motivational Interviewing and Booster Lessons. *Journal of medical*

For peer review only

Internet research 2022;24(9):e40637. doi: 10.2196/40637 [published Online First: 20220907]

36. Powell J, Hamborg T, Stallard N, et al. Effectiveness of a web-based cognitive-behavioral tool to improve mental well-being in the general population: randomized controlled trial. *J Med Internet Res* 2012;15(1):e2. doi: 10.2196/jmir.2240 [published Online First: 2013/01/11]
37. Proudfoot J, Clarke J, Birch MR, et al. Impact of a mobile phone and web program on symptom and functional outcomes for people with mild-to-moderate depression, anxiety and stress: a randomised controlled trial. *BMC Psychiatry* 2013;13:312. doi: 10.1186/1471-244X-13-312 [published Online First: 2013/11/19]
38. Robichaud M, Talbot F, Titov N, et al. Facilitating access to iCBT: a randomized controlled trial assessing a translated version of an empirically validated program using a minimally monitored delivery model. *Behav Cogn Psychother* 2020;48(2):185-202. doi: 10.1017/S135246581900047X [published Online First: 2019/08/17]
39. Rogers J, Sicouri G. A Single-Session Online Cognitive Bias Modification of Interpretations Modified for Adults With Anxiety and Depressive Symptoms. *Behav Ther* 2022;53(5):967-80. doi: 10.1016/j.beth.2022.04.006 [published Online First: 2022/08/21]
40. Rollman BL, Herbeck Belnap B, Abebe KZ, et al. Effectiveness of Online Collaborative Care for Treating Mood and Anxiety Disorders in Primary Care: A Randomized Clinical Trial. *JAMA Psychiatry* 2018;75(1):56-64. doi: 10.1001/jamapsychiatry.2017.3379 [published Online First: 2017/11/09]
41. Schaeuffele C, Homeyer S, Perea L, et al. The unified protocol as an internet-based intervention for emotional disorders: Randomized controlled trial. *PLoS One* 2022;17(7):e0270178. doi: 10.1371/journal.pone.0270178 [published Online First: 2022/07/12]
42. Sethi S. Treating Youth Depression and Anxiety: A Randomised Controlled Trial Examining the Efficacy of Computerised versus Face-to-face Cognitive Behaviour Therapy. *Australian Psychologist* 2013;48(4):249-57. doi: 10.1111/ap.12006
43. Soucy JN, Hadjistavropoulos HD, Karin E, et al. Brief online motivational interviewing pre-treatment intervention for enhancing internet-delivered cognitive behaviour therapy: A randomized controlled trial. *Internet Interv* 2021;25:100394. doi: 10.1016/j.invent.2021.100394 [published Online First: 20210420]
44. Southwell S, Gould E. A randomised wait list-controlled pre–post–follow-up trial of a gratitude diary with a distressed sample. *The Journal of Positive Psychology* 2016;12(6):579-93. doi: 10.1080/17439760.2016.1221127
45. Taylor H, Cavanagh K, Field AP, et al. Health Care Workers' Need for Headspace: Findings From a Multisite Definitive Randomized Controlled Trial of an Unguided Digital Mindfulness-Based Self-help App to Reduce Healthcare Worker Stress. *JMIR Mhealth Uhealth* 2022;10(8):e31744. doi: 10.2196/31744 [published Online First: 2022/08/26]
46. Terides MD, Dear BF, Fogliati VJ, et al. Increased skills usage statistically mediates symptom reduction in self-guided internet-delivered cognitive-behavioural therapy for depression and anxiety: a randomised controlled trial. *Cogn Behav Ther* 2018;47(1):43-61. doi: 10.1080/16506073.2017.1347195 [published Online First: 2017/07/21]

For peer review only

Internet-delivered transdiagnostic psychological treatments for individuals with depression, anxiety, or both: a systematic review with meta-analysis of randomized controlled trials

47. Titov N, Dear BF, Schwencke G, et al. Transdiagnostic internet treatment for anxiety and depression: a randomised controlled trial. *Behav Res Ther* 2011;49(8):441-52. doi: 10.1016/j.brat.2011.03.007 [published Online First: 2011/06/18]
48. Titov N, Dear BF, Johnston L, et al. Improving adherence and clinical outcomes in self-guided internet treatment for anxiety and depression: randomised controlled trial. *PLoS One* 2013;8(7):e62873. doi: 10.1371/journal.pone.0062873 [published Online First: 2013/07/12]
49. Titov N, Fogliati VJ, Staples LG, et al. Treating anxiety and depression in older adults: randomised controlled trial comparing guided v. self-guided internet-delivered cognitive-behavioural therapy. *BJPsych Open* 2016;2(1):50-58. doi: 10.1192/bjpo.bp.115.002139 [published Online First: 2016/10/06]
50. Tönnies J, Hartmann M, Wensing M, et al. Mental Health Specialist Video Consultations Versus Treatment-as-Usual for Patients With Depression or Anxiety Disorders in Primary Care: Randomized Controlled Feasibility Trial. *JMIR Ment Health* 2021;8(3):e22569. doi: 10.2196/22569 [published Online First: 2021/03/13]
51. Tulbure BT, Rusu A, Sava FA, et al. A Web-Based Transdiagnostic Intervention for Affective and Mood Disorders: Randomized Controlled Trial. *JMIR Ment Health* 2018;5(2):e36. doi: 10.2196/mental.8901 [published Online First: 2018/05/26]
52. Twomey C, O'Reilly G, Byrne M, et al. A randomized controlled trial of the computerized CBT programme, MoodGYM, for public mental health service users waiting for interventions. *Br J Clin Psychol* 2014;53(4):433-50. doi: 10.1111/bjc.12055 [published Online First: 2014/05/17]
53. van Straten A, Cuijpers P, Smits N. Effectiveness of a web-based self-help intervention for symptoms of depression, anxiety, and stress: randomized controlled trial. *J Med Internet Res* 2008;10(1):e7. doi: 10.2196/jmir.954 [published Online First: 2008/03/28]
54. Villemaire-Krajden R, Myhr G. Evaluating the Use of a Computerized CBT Program for Outpatients on a Waitlist in a University CBT Unit. *J Psychiatr Pract* 2019;25(4):268-78. doi: 10.1097/PRA.0000000000000396 [published Online First: 2019/07/11]
55. Viravan N, Atsariyasing W, Srifuengfung M, et al. Efficacy of Online Mindfulness Program 'Mindful Senses' for Depression and Anxiety Reduction in Community Samples: A Randomized Controlled Trial. *J Med Assoc Thai* 2022;105(11):1108-19. doi: 10.35755/jmedassocthai.2022.11.13700
56. Viskovich S, Pakenham KI. Randomized controlled trial of a web-based Acceptance and Commitment Therapy (ACT) program to promote mental health in university students. *J Clin Psychol* 2020;76(6):929-51. doi: 10.1002/jclp.22848 [published Online First: 2019/08/31]
57. Zetterqvist K, Maanmies J, Strom L, et al. Randomized controlled trial of internet-based stress management. *Cogn Behav Ther* 2003;32(3):151-60. doi: 10.1080/16506070302316 [published Online First: 2005/11/18]
58. Shi L, Lin L. The trim-and-fill method for publication bias: practical guidelines and recommendations based on a large database of meta-analyses. *Medicine* 2019;98(23)

For peer review only

Outcome / reserch question	RCT design	1. Risk of E	2. Inconsis	3. Indirect	4. Imprecis
Adults vs. TAU/WLC (34 RCTs)	4	-2	-1	0	0
Adults vs. attention/engagement (8 RCTs)	4	-2	-2	0	-1
Adults vs. bona fide (3 RCTs)	4	-2	-2	0	-2
Children/adolescents (2 RCTs)	4	-1	-2	0	-2
Primary care (3 RCTs)	4	0	-2	0	-2

For peer review only

5. Publicat	6*. Large r	7*. Dose R	8*. Effect c	GRADE	Quality
-1	0	0	0	0	Very low
-1	0	0	0	-2	Very low
-1	0	0	0	-3	Very low
-1	0	0	0	-2	Very low
-1	0	0	0	-1	Very low

For peer review only

PRISMA 2020 Checklist

Section and Topic	Item #	Checklist item	Location where item is reported
**TITLE**			Page:
Title	1	Identify the report as a systematic review.	1
**ABSTRACT**			
Abstract	2	See the PRISMA 2020 for Abstracts checklist.	3
**INTRODUCTION**			
Rationale	3	Describe the rationale for the review in the context of existing knowledge.	4
Objectives	4	Provide an explicit statement of the objective(s) or question(s) the review addresses.	5
**METHODS**			
Eligibility criteria	5	Specify the inclusion and exclusion criteria for the review and how studies were grouped for the syntheses.	6,7,8
Information sources	6	Specify all databases, registers, websites, organisations, reference lists and other sources searched or consulted to identify studies. Specify the date when each source was last searched or consulted.	5
Search strategy	7	Present the full search strategies for all databases, registers and websites, including any filters and limits used.	5 + Supplement A
Selection process	8	Specify the methods used to decide whether a study met the inclusion criteria of the review, including how many reviewers screened each record and each report retrieved, whether they worked independently, and if applicable, details of automation tools used in the process.	6
Data collection process	9	Specify the methods used to collect data from reports, including how many reviewers collected data from each report, whether they worked independently, any processes for obtaining or confirming data from study investigators, and if applicable, details of automation tools used in the process.	8,9
Data items	10a	List and define all outcomes for which data were sought. Specify whether all results that were compatible with each outcome domain in each study were sought (e.g. for all measures, time points, analyses), and if not, the methods used to decide which results to collect.	8,9
	10b	List and define all other variables for which data were sought (e.g. participant and intervention characteristics, funding sources). Describe any assumptions made about any missing or unclear information.	8,9
Study risk of bias assessment	11	Specify the methods used to assess risk of bias in the included studies, including details of the tool(s) used, how many reviewers assessed each study and whether they worked independently, and if applicable, details of automation tools used in the process.	10
Effect measures	12	Specify for each outcome the effect measure(s) (e.g. risk ratio, mean difference) used in the synthesis or presentation of results.	10, 11
Synthesis methods	13a	Describe the processes used to decide which studies were eligible for each synthesis (e.g. tabulating the study intervention characteristics and comparing against the planned groups for each synthesis (item #5)).	9 - 11
	13b	Describe any methods required to prepare the data for presentation or synthesis, such as handling of missing summary statistics, or data conversions.	10, 11
	13c	Describe any methods used to tabulate or visually display results of individual studies and syntheses.	10, 11
	13d	Describe any methods used to synthesize results and provide a rationale for the choice(s). If meta-analysis was performed, describe the model(s), method(s) to identify the presence and extent of statistical heterogeneity, and software package(s) used.	10-11
	13e	Describe any methods used to explore possible causes of heterogeneity among study results (e.g. subgroup analysis, meta-regression).	11
	13f	Describe any sensitivity analyses conducted to assess robustness of the synthesized results.	11
Reporting bias assessment	14	Describe any methods used to assess risk of bias due to missing results in a synthesis (arising from reporting biases).	11

For peer review only - http://bmjopen.bmj.com/site/about/guidelines.xhtml

PRISMA 2020 Checklist

Section and Topic	Item #	Checklist item	Location where item is reported
Certainty assessment	15	Describe any methods used to assess certainty (or confidence) in the body of evidence for an outcome.	11
**RESULTS**			
Study selection	16a	Describe the results of the search and selection process, from the number of records identified in the search to the number of studies included in the review, ideally using a flow diagram.	Fig 1
	16b	Cite studies that might appear to meet the inclusion criteria, but which were excluded, and explain why they were excluded.	11
Study characteristics	17	Cite each included study and present its characteristics.	
Risk of bias in studies	18	Present assessments of risk of bias for each included study.	11 + Fig 2
Results of individual studies	19	For all outcomes, present, for each study: (a) summary statistics for each group (where appropriate) and (b) an effect estimate and its precision (e.g. confidence/credible interval), ideally using structured tables or plots.	12, Fig 3, Fig 4
Results of syntheses	20a	For each synthesis, briefly summarise the characteristics and risk of bias among contributing studies.	13
	20b	Present results of all statistical syntheses conducted. If meta-analysis was done, present for each the summary estimate and its precision (e.g. confidence/credible interval) and measures of statistical heterogeneity. If comparing groups, describe the direction of the effect.	13
	20c	Present results of all investigations of possible causes of heterogeneity among study results.	13
	20d	Present results of all sensitivity analyses conducted to assess the robustness of the synthesized results.	14
Reporting biases	21	Present assessments of risk of bias due to missing results (arising from reporting biases) for each synthesis assessed.	14, Supplement 2
Certainty of evidence	22	Present assessments of certainty (or confidence) in the body of evidence for each outcome assessed.	13, 14, Figure 3, Figure 4,
**DISCUSSION**			
Discussion	23a	Provide a general interpretation of the results in the context of other evidence.	16, 17, 18
	23b	Discuss any limitations of the evidence included in the review.	19
	23c	Discuss any limitations of the review processes used.	19
	23d	Discuss implications of the results for practice, policy, and future research.	19
**OTHER INFORMATION**			
Registration and protocol	24a	Provide registration information for the review, including register name and registration number, or state that the review was not registered.	5
	24b	Indicate where the review protocol can be accessed, or state that a protocol was not prepared.	5
	24c	Describe and explain any amendments to information provided at registration or in the protocol.	10
Support	25	Describe sources of financial or non-financial support for the review, and the role of the funders or sponsors in the review.	21
Competing interests	26	Declare any competing interests of review authors.	3
Availability of	27	Report which of the following are publicly available and where they can be found: template data collection forms; data extracted from included	21

For peer review only

PRISMA 2020 Checklist

Section and Topic	Item #	Checklist item	Location where item is reported
data, code and other materials		studies; data used for all analyses; analytic code; any other materials used in the review.	

From: Page MJ, McKenzie JE, Bossuyt PM, Boutron I, Hoffmann TC, Mulrow CD, et al. The PRISMA 2020 statement: an updated guideline for reporting systematic reviews. BMJ 2021;372:n71. doi: 10.1136/bmj.n71
For more information, visit: http://www.prisma-statement.org/

For peer review only

BMJ Open

Internet-delivered transdiagnostic psychological treatments for individuals with depression, anxiety, or both: a systematic review with meta-analysis of randomized controlled trials

Journal:	*BMJ Open*
Manuscript ID	bmjopen-2023-075796.R2
Article Type:	Original research
Date Submitted by the Author:	23-Feb-2024

Complete List of Authors:	Kolaas, Karoline; Karolinska Institute Department of Clinical Neuroscience, Centre for Psychiatry Research; Academic Primary Care Centre, Liljeholmen University Primary Health care Center, Region Stockholm<br>Berman, Anne H.; Uppsala Universitet, Department of Psychology; Karolinska Institute Department of Clinical Neuroscience, Centre for Psychiatry Research<br>Hedman-Lagerlöf, Erik ; Karolinska Institute Department of Clinical Neuroscience, Division of Psychology; Academic Primary Care Centre, Gustavsberg University Primary Health Care Center, Region Stockholm<br>Lindsäter, Elin; Karolinska Institute Department of Clinical Neuroscience, Department of Clinical Neuroscience; Academic Primary Care Centre, Gustavsberg University Primary Health Care Center, Region Stockholm<br>Hybelius, Jonna; Karolinska Institute, Division of Family Medicine and Primary Care, Department of Neurobiology, Care Sciences and Society; Academic Primary Care Centre, Liljeholmen University Primary Health Care Center, Region Stockholm<br>Axelsson, Erland; Karolinska Institutet, Division of Family Medicine and Primary Care, Department of Neurobiology, Care Sciences and Society; Academic Primary Care Centre, Liljeholmen University Primary Health Care Center, Region Stockholm

**Primary Subject Heading**:	Mental health
Secondary Subject Heading:	General practice / Family practice, Evidence based practice, Mental health
Keywords:	MENTAL HEALTH, PSYCHIATRY, Depression & mood disorders < PSYCHIATRY, Anxiety disorders < PSYCHIATRY, Patient Reported Outcome Measures, Systematic Review

SCHOLARONE™
Manuscripts

I, the Submitting Author has the right to grant and does grant on behalf of all authors of the Work (as defined in the below author licence), an exclusive licence and/or a non-exclusive licence for contributions from authors who are: i) UK Crown employees; ii) where BMJ has agreed a CC-BY licence shall apply, and/or iii) in accordance with the terms applicable for US Federal Government officers or employees acting as part of their official duties; on a worldwide, perpetual, irrevocable, royalty-free basis to BMJ Publishing Group Ltd ("BMJ") its licensees and where the relevant Journal is co-owned by BMJ to the co-owners of the Journal, to publish the Work in this journal and any other BMJ products and to exploit all rights, as set out in our licence.

The Submitting Author accepts and understands that any supply made under these terms is made by BMJ to the Submitting Author unless you are acting as an employee on behalf of your employer or a postgraduate student of an affiliated institution which is paying any applicable article publishing charge ("APC") for Open Access articles. Where the Submitting Author wishes to make the Work available on an Open Access basis (and intends to pay the relevant APC), the terms of reuse of such Open Access shall be governed by a Creative Commons licence – details of these licences and which Creative Commons licence will apply to this Work are set out in our licence referred to above.

Other than as permitted in any relevant BMJ Author's Self Archiving Policies, I confirm this Work has not been accepted for publication elsewhere, is not being considered for publication elsewhere and does not duplicate material already published. I confirm all authors consent to publication of this Work and authorise the granting of this licence.

For peer review only

Internet-delivered transdiagnostic psychological treatments for individuals with depression, anxiety, or both: a systematic review with meta-analysis of randomized controlled trials

Karoline Kolaas, PhD[a b]; Anne H Berman, PhD[a c]; Erik Hedman-Lagerlöf, PhD[d e]; Elin Lindsäter, PhD[a d e]; Jonna Hybelius, MSc[b f]; and Erland Axelsson, PhD[b f]

Author Affiliations:[a] Centre for Psychiatry Research, Department of Clinical Neuroscience, Karolinska Institutet & Stockholm Health Care Services, Region Stockholm, Stockholm, Sweden (Dr Kolaas, Dr Berman, and Dr Lindsäter),[b] Liljeholmen University Primary Health Care Center, Academic Primary Health Care Center, Region Stockholm, Stockholm, Sweden (Dr Kolaas, Ms Hybelius, and Dr Axelsson),[c] Division of Clinical Psychology, Department of Psychology, Uppsala University, Uppsala, Sweden (Dr Berman),[d] Division of Psychology, Department of Clinical Neuroscience, Karolinska Institutet, Stockholm, Sweden (Dr Hedman-Lagerlöf),[e] Gustavsberg University Primary Health Care Center, Academic Primary Health Care Center, Region Stockholm, Gustavsberg, Sweden (Dr Hedman-Lagerlöf and Dr Lindsäter), and [f] Division of Family Medicine and Primary Care, Department of Neurobiology, Care Sciences and Society, Karolinska Institutet, Stockholm, Sweden (Ms Hybelius and Dr Axelsson).

Corresponding Author: Karoline Kolaas, PhD, Centre for Psychiatry Research, Department of Clinical Neuroscience, Karolinska Institutet, Norra Stationsgatan 69, SE-113 64, Stockholm, Sweden,

Tel: +46 73 655 69 91

For peer review only

For peer review only - http://bmjopen.bmj.com/site/about/guidelines.xhtml

E-mail: karoline.kolaas@ki.se

Word count (abstract): 296; (main text): 4938; Tables: 1; Figures: 4

Page count (main text): 21; Line count (main text): 441

For peer review only

For peer review only - http://bmjopen.bmj.com/site/about/guidelines.xhtml

Abstract

Objective: Depression and anxiety are major public health problems. This study evaluated the effects of internet-delivered transdiagnostic psychological treatments for individuals with depression, anxiety, or both.

Design: Systematic review with meta-analysis.

Data sources: Medline (Ovid), Cochrane Library (Wiley), the Web of Science Core Collection (Clarivate), and PsycInfo (EBSCO) were searched on 24 May 2021, with an update on 6 February 2023.

Eligibility criteria: Randomized controlled trials of internet-delivered transdiagnostic psychological treatments, open to both participants with primary depression and participants with primary anxiety. This review concerned all treatment frameworks, both guided and unguided formats, and all age groups.

Data extraction and synthesis: In random effects meta-analysis, we estimated pooled effects on depression symptoms and anxiety in terms of Hedges' *g* with 95% CIs. Absolute and relative heterogeneity was quantified as the τ^2 and I^2.

Results: We included 57 trials with 21795 participants. Nine trials (16%) recruited exclusively from routine care, and three (5%) delivered treatment via video. For adults, large within-group reductions were seen in depression (*g* = 0.90; 95% CI 0.81-0.99) and anxiety (*g* = 0.87; 95% CI 0.78-0.96). Compared to rudimentary passive controls, the added effects were moderate (depression: *g* = 0.52; 95% CI 0.42-0.63; anxiety: *g* = 0.45; 95% CI 0.34-0.56), and

For peer review only - http://bmjopen.bmj.com/site/about/guidelines.xhtml

larger in trials that required all participants to meet full diagnostic criteria for depression or an anxiety disorder. Compared to attention/engagement controls, the added effects were small (depression: $g = 0.30$; 95% CI 0.07-0.53; anxiety: $g = 0.21$; 95% CI 0.01-0.42). Heterogeneity was substantial, and the certainty of the evidence was very low. Two trials concerned adolescents and reported mixed results. One trial concerned older adults and reported promising results.

Conclusion: Internet-delivered transdiagnostic treatments for depression and anxiety show small to moderate added effects, varying by control condition. Research is needed regarding routine care, the video format, children and adolescents, and older adults.

PROSPERO registration number: CRD42021243172.

<Strengths and limitations of this study>

- The systematic search strategy and the broad eligibility criteria allowed for a comprehensive overview of treatment frameworks, formats, age groups, and settings including primary health care.
- Several trial, participant, and treatment characteristics were evaluated as potential moderators of the between-group effect versus rudimentary passive controls.
- The majority of trials recruited self-referred participants through advertisement and were not conducted in clinical settings. This makes it more difficult to draw conclusions about effectiveness in routine care.

For peer review only

For peer review only - http://bmjopen.bmj.com/site/about/guidelines.xhtml

- Our analyses of moderators were based exclusively on trial-level characteristics, and should preferably be replicated based on individual patient data.

<Introduction>

Depression and the common anxiety disorders are associated with immense worldwide healthcare costs and disease burden.(1 2) Psychological treatments for depression and anxiety can be effective(3) including in clinical settings such as primary care(4 5) where the majority of patients are found.(6) However, many patients with depression and anxiety disorders remain untreated or receive inadequate treatment,(7 8) illustrating the need for further development and dissemination of effective therapies.

It is widely recognized that depression and anxiety disorders often occur together,(9 10) and overlap in terms of genetic, cognitive, and behavioral characteristics.(11-13) This speaks for the use of broader, transdiagnostic, psychological treatments designed to suit patients suffering from a range of different types of depression and anxiety problems. That is, transdiagnostic treatments are characterized by focusing on components relevant for both depression and anxiety, in contrast to diagnosis-specific treatments targeting one specific mental health conditon. In addition to beneficial effects for the patient, transdiagnostic treatments can also have organizational advantages, such as streamlined procedures and less time needed for diagnostic assessment.(14). In early meta-analysis, transdiagnostic psychological treatments have been found to have moderate pooled effects on depression and anxiety compared to mostly rudumentary treatment-as-usual and waitlist control groups.(15)

Another method of improving access to psychological treatments can be to deliver these online – for example via websites, mobile apps, or videoconferencing – which saves time and costs for both clinicians and patients. For internet-delivered transdiagnostic psychological

For peer review only - http://bmjopen.bmj.com/site/about/guidelines.xhtml

treatments, large within-group symptom reductions have been observed, with early meta-analyses reporting mixed outcomes compared to a heterogeneous range of control groups, and comparisons versus diagnosis-specific treatments typically result in relatively similar effects on depression and general anxiety.(16-18) Internet-delivered therapies appear to be accepted by patients, and have shown promising witin-group effects in pragmatic routine care studies.

Even though important advancements have been made in internet-delivered transdiagnostic psychological treatments for depression and anxiety in the past decades, several fundamental questions remain unanswered in this rapidly growing field. First, it remains unclear whether effects generalize to children, adolescents, and older adults. Second, there has been no systematic comparison of treatment frameworks beyond the cognitive-behavioral therapies. Third, the relative effect of methods of delivery such as via websites, mobile apps, and videoconferencing is yet unclear. Fourth, although acceptability and within-group change has been studied to some extent in the routine care environment(19), based on randomized controlled trials that include a combination of patients with anxiety and depression, it is not known how well the effects of internet-delivered formats translate to routine clinical settings such as primary care.

We conducted a systematic review and meta-analysis of randomized controlled trials of internet-delivered transdiagnostic psychological treatments for depression and anxiety. This systematic review included all treatment frameworks, both guided and unguided formats, and all age groups. Based on previous research, we hypothesized that between-group effects versus rudimentary passive controls would be large, and that the treatment length would be of little importance for the overall effect.

For peer review only - http://bmjopen.bmj.com/site/about/guidelines.xhtml

<Method>

<Search strategy>

This systematic review with meta-analysis adhered to the PRISMA 2020 statement(20) and was registered at PROSPERO (CRD42021243172) on April 16, 2021 and at the Open Science Framework (https://osf.io/dtcey) on May 18, 2021, i.e., before the initial database searches. We searched Medline (Ovid), Cochrane Library (Wiley), the Web of Science Core Collection (Clarivate), and PsycInfo (EBSCO) for randomized controlled trials of internet-delivered transdiagnostic psychological treatments for individuals with depression, anxiety, or both. A search strategy was developed in collaboration with the Karolinska Institutet library service. Candidate search strings were validated against a selection of articles that we were aware of prior to this systematic review, and after discussion within the research group, as well as with an external expert in psychodynamic online therapies. Four types of search terms were combined: terms for treatment (e.g., “psychotherapy”), terms for online communication (e.g., “internet-delivered”), terms for diagnostic focus (e.g., “transdiagnostic”, “depression”, “anxiety”), and terms for study design (e.g., “RCT”). Only publications published in 1995 or later were considered because there were no RCTs of internet-delivered transdiagnostic psychological treatments before then. Full search documentation for all databases can be found in Supplement A. An initial search was conducted on 24 May 2021, with an update on 6 February 2023 in accordance with Bramer and Bain.(21) We also reviewed the reference lists of previous meta-analyses that we found relevant.(16 17)

For peer review only - http://bmjopen.bmj.com/site/about/guidelines.xhtml

<*Selection of studies*>

We employed a systematic de-duplication algorithm in Endnote X9,(22) after which unique search hits were imported into Rayyan.(23) The publications were then assessed by two independent assessors in two phases. In phase 1, all titles and abstracts were screened for eligibility by two authors (of KK, AHB, EHL, and EA), and non-relevant publications were excluded. In phase 2, publications that had been included by at least one assessor in phase 1 were reviewed in full text. One assessor (KK) did this sequentially, i.e., from criterion a to h (see below), for all full texts. Each full text was also reviewed by a second assessor (either AHB, EHL, JH, or EA) who suggested a decision of exclusion based on the first exclusion criterion identified, i.e., in any order. Cases of disagreement were discussed in the research group and a final decision was reached either in consensus or, if necessary, by means of voting. If information about the eligibility criteria a-h was missing, authors were not contacted to provide additional information.

<*Eligibility criteria*>

(a) Studies had to be published in English in a peer-reviewed journal.

(b) Studies had to be randomized controlled trials where the study participant was the unit of randomization. This was because our focus was on conducting meta-analyses, for which cluster randomized trials require distinct analytical methods.(24) Only one publication was formally included for each randomized trial.

(c) Studies were required to evaluate the effect of an internet-delivered transdiagnostic psychological treatment for individuals with anxiety, depression, or both. "Internet-delivered" included treatments delivered via websites, mobile apps, or videoconferencing. Treatments where online components were a mere complement to a face-to-face treatment, or where participants were required to attend a particular

For peer review only - http://bmjopen.bmj.com/site/about/guidelines.xhtml

physical location, were excluded. “Transdiagnostic” was defined as intending to target depression as well as anxiety. “Psychological treatment” was defined as an intervention anchored in psychological theory with a clinical framing (i) where a clinician or facilitator had an active role in moderating the process, or (ii) which was delivered in a structured manner, for example in the form of modules with suggested homework, or with an interactive component such as automatic feedback. For example, a webpage with general information about depression and anxiety would not be considered a psychological treatment, due to the lack of structure and interactivity.

(d) Studies needed to recruit participants suffering from clinically significant depression, anxiety, or both. We considered this criterion to be met if one of the following was true: (i) all participants were required to meet full diagnostic criteria for either primary depression or a primary anxiety disorder and both populations were included in the trial, or (ii) all participants were required to score above a valid cut-off on an accepted screening measure for either depression or anxiety and both populations were included in the trial, or (iii) all participants were required to score above both a valid cut-off for depression and a valid cut-off for anxiety, or (iv) the baseline sample mean of at least one transdiagnostic treatment arm and at least one other arm were above both a valid cut-off for depression and a valid cut-off for anxiety. We excluded RCTs that either required all participants to meet full formal diagnostic criteria for depression, or required all participants to meet full formal diagnostic criteria for an anxiety disorder. This was to ensure a reasonably broad recruitment strategy, and to further reduce the risk that the treamtent had focused overwhealmingly on one specific psychiatric disorder. We also excluded RCTs that required all participants to meet full formal diagnostic criteria for both depression and an anxiety disorder at the same time. This is a very unusual design that requires all participants to have a very

For peer review only - http://bmjopen.bmj.com/site/about/guidelines.xhtml

substantial level of symptomatology that we suspected could be indicative of a highly specialized care setting unlike primary care which we were primarily interested in. Studies that subsumed obsessive-compulsive disorder or post-traumatic stress disorder under the umbrella of anxiety disorders, in accordance with earlier DSM versions, were included. Studies that included a substantial proportion of subclinical participants were included only if clinical participants were reported separately.

(e) Studies were required to evaluate treatments for samples that were representative for a general medical or mental health clinical setting in the sense that the sample had not been heavily selected on any particular clinical characteristic or medical condition other than that of primary interest. This led to the exclusion for example of treatments specifically aimed at cancer patients with depression or anxiety.

(f) Studies were required to have at least 10 participants in one arm enrolled in internet-delivered transdiagnostic psychological treatment. All control groups were included. Studies were required to have employed at least one valid self-report questionnaire for depression or anxiety. If severeral valid questionnaires were reported, the one described as primary was used. If no questionnaire was described as primary, we tabulated that which was reported first in the manuscript. Domain-specific anxiety scales such as those concerning social anxiety, health anxiety, or panic disorder anxiety were not considered adequate.

(g) Post-treatment assessment needed to be completed within three months after treatment termination, so as to not lie closer to a typical follow-up assessment.

(h) Studies were required to have reported means and standard deviations, or provided information that made it possible to derive such estimates. Typically, this was based on the original publication. In certain cases, conversions were applied, for example when the standard deviation could be derived from the sample size and the standard

For peer review only - http://bmjopen.bmj.com/site/about/guidelines.xhtml

error of the mean. Whenever necessary, the author was also contacted and encouraged to provide estimates via email.

<Data extraction>

Pre-specified variables were extracted by the first author (KK), including participant demographics (e.g., age and gender). Studies were considered to concern children or adolescents if the mean age was below 18, and to concern older adults if the mean age was above 65. The following study and sample characteristics were extracted: study setting (primary care, not primary care, or unclear), recruitment path (routine care or not routine care), main inclusion criterion (psychiatric diagnosis or "cut-off symptoms"), mean depression severity (mild, moderate, or severe), and mean anxiety severity (mild, moderate, or severe). Symptom severity ratings were based on published norm data.(25-28) Treatment characteristics extracted were: delivery format (website, mobile app, video, mixed or other), therapist support (guided or unguided), treatment type (CBT, mindfulness, mixed CBT and mindfulness, or other), specific treatment protocol, treatment length (≥6 weeks or <6 weeks; intended to contrast what are usually considered brief therapies with therapies of a more conventional length), component flexibility (standardized or tailored), and therapist time (minutes per participant in treatment). In line with recent findings demonstrating that many treatment-as-usual control groups are virtually indistinguishable from waitlist controls,(29) we did not lump all treatment-as-usual controls into the same category but rather made a distinction between (a) rudimentary passive controls that comprised waitlist controls and other controls without a structured intervention, (b) attention/engagement controls where the participants received some sort of standardized intervention that controlled for the attention from a caregiver or engagement in a study but without this being a conventional psychological treament, and (c) bona-fide treatments "delivered by trained therapists and [...]

For peer review only - http://bmjopen.bmj.com/site/about/guidelines.xhtml

based on psychological principles, [...] offered to the psychotherapy community as viable treatments".(30) For each trial, the primary outcome for depression and anxiety was tabulated. If no outcome was defined as primary, or the primary outcome measure was deemed not to be valid, we tabulated the first valid measure that was mentioned. KK contacted the corresponding author when main outcome data was missing. All authors were also asked to provide pre-post correlations for the calculation of within-group effects. Intention-to-treat estimates were tabulated whenever available.

<Assessment of risk of bias and quality of the evidence base>

Trial risk of bias was assessed using a modified version of the Cochrane risk-of-bias tool for randomized trials version 2 (RoB 2).(31) The RoB 2 covers five domains: (i) the randomization process, (ii) deviations from intended interventions, (iii) missing outcome data, (iv) measurement of the outcome, and (v) selection of the reported results. Because of the difficulty blinding therapists to the psychological treatment they are delivering, we omitted items related to blinding. Consistent with previous similar research, non-blinding did not therefore lead to an elevated risk of bias.(15 32) Each trial received one rating on each risk of bias domain, and this rating concerned both the depression and the anxiety outcome. Reporting one score was sufficient because all trials administered the depression and anxiety measures in parallel, which resulted in identical ratings. Authors KK and EL independently assessed the trials and discussed cases of disagreement to reach consensus. The decision was made that if five articles in a row were rated identically by both assessors, it would no longer be necessary with two assessments per article. This target was achieved after 25 articles, and KK assessed the remaining trials. Based partially on the risk of bias ratings, we assessed the overall quality of the evidence base according to GRADE.(33) We assigned separate ratings for the evidence base pertaining to (i) effects versus rudimentary passive controls for adults,

For peer review only

For peer review only - http://bmjopen.bmj.com/site/about/guidelines.xhtml

(ii) effects versus attention/engagement controls for adults, (iii) effects versus other bona-fide treatments for adults, (iv) effects for children and adolescents, and (v) effects in primary care. Similar to the risk of bias assessment, one common score was given for depression and anxiety.

<*Statistical analysis*>

We report studies focusing on children and adolescents, and studies focusing on older adults, separately due to their specific focus. Effects versus other bona fide treatments were too few to be included in meta-analysis. At the end of the tabulation phase, we also decided to report studies with a treatment length of two weeks or shorter separately, a duration which would not typically be regarded as bona fide in routine clinical care. Nine randomized factorial trials were also included in the meta-analysis of within-group effects, but their main results were summarized separately.

We proceeded with meta-analysis using random-effects models fitted with the restricted maximum likelihood estimator using the inverse variance method, in R 4.2.0(34) with the metafor 3.8-1 package.(35) Effects were quantified in terms of Hedges' *g*, i.e., the bias-corrected standardized mean difference.(36) Although not our primary focus, we first conducted a meta-analysis of the within-group change in treatment. The point of this was to ensure that change had been observed per se, to determine if heterogeneity was much reduced without the influence of variance due to different control groups, and to facilitate the interpretation of within-group effects in future studies. For this analysis, we standardized *g* on the pre-treatment standard deviation, and used the pre-post correlation (imputed pooled estimate if missing) to determine the sampling variance (37 38):

For peer review only - http://bmjopen.bmj.com/site/about/guidelines.xhtml

$$g = \frac{M_{pre} - M_{post}}{SD_{pre}} \times \sqrt{\frac{2}{(n-1)}} \left(\frac{\Gamma[(n-1)/2]}{\Gamma[(n-2)/2]} \right)$$

$$approx.\, variance(g) = \frac{2(1 - r_{prepost})}{n} + \frac{g^2}{2n}$$

We then conducted a meta-analysis of between-group effects versus rudimentary passive controls, and a meta-analysis of between-group effects versus attention/engagement controls. For these analyses, we standardized g on on the pooled post-treatment standard deviation, and employed the large-sample approximation of the sampling variance (36 39):

$$g = -\frac{M_{tx} - M_{control}}{SD_{pooled}} \times \sqrt{\frac{2}{(n_{tx} + n_{control} - 2)}} \left(\frac{\Gamma[(n_{tx} + n_{control} - 2)/2]}{\Gamma[(n_{tx} + n_{control} - 3)/2]} \right)$$

$$approx.\, variance(g) = \frac{n_{tx} + n_{control}}{n_{tx} n_{control}} + \frac{g^2}{2(n_{tx} + n_{control})}$$

Absolute values for g of 0.2 are usually regarded as small, 0.5 as moderate, and 0.8 as large.(40) We quantified absolute heterogeneity in terms of the τ^2, and also report the I^2 which stands for the proportion of the between-sample variance that is due to true study differences as opposed to sampling error. An I^2 of 25% is usually regarded as low, 50% as moderate, and 75% as high,(41) though the I^2 also increases with the precision of the original trials.(42) Pertaining to the meta-analysis of trials of transdiagnostic treatment vs. rudimentary passive controls, a series of planned moderator analyses were conducted on the basis of trial, sample, and treatment characteristics whenever strata included at least four studies. One exception was made to this rule where we analyzed primary care setting as a potential moderator based on four arms from three trials because we found this test important. In the analysis of trials with rudimentary controls, we also investigated publication bias based on the visual inspection of funnel plots in combination with Egger's test(43) and the Duval and Tweedie trim and fill procedure.(44) In line with recommendations,(45) the latter was repeated using the R0, L0, and Q0 estimators.

For peer review only

<*Patient and Public Involvement*>

None

<Results>

The inclusion process is presented in Fig. 1. Out of 435 RCTs reviewed in full text, 55 were suggested for inclusion by both assessors, 335 were immediate exclusions, and 45 were disagreements (90% agreement; κ = 0.65). The reasons for these disagreements are listed in Supplement B. Of the disagreements, five trials were included after discussion. Three trials were then excluded due to insufficient data. This resulted in a final sample of 57 RCTs. Full references to these trials are found in Supplement B.

INSERT FIGURE 1 ABOUT HERE

<*Study characteristics*>

In the 57 included trials, 21795 participants were randomized.(46-102)Arms were: 83 internet-delivered transdiagnostic psychological treatments, 36 rudimentary controls, 8 attention/engagement controls, and three other bona fide treatments. With regard to age groups, 53/57 trials (93%) recruited adults, 3/57 (5%) children or adolescents, and 1/57 (2%) specifically older adults. As to delivery formats, 37/57 (65%) of the internet-delivered transdiagnostic psychological treatments were delivered via a website mainly through text, 8/57 (14%) via a mobile app, 3/57 (5%) via video, and 9/57 (16%) were mixed or other. In terms of the therapeutic model, 37/57 (65%) evaluated some variation on cognitive behavioral therapy (CBT), 8/57 (14%) mindfulness-based therapy, 3/57 (5%) mixed CBT and mindfulness, and 11/57 (19%) other frameworks such as problem-solving therapy or affect-focused psychodynamic therapy. As for the recruitment strategy, 39/57 (68%) trials relied on

For peer review only - http://bmjopen.bmj.com/site/about/guidelines.xhtml

self-referrals, 9/57 (16%) recruited from routine care including student counselling, and 9/57 (16%) used a mixed strategy or other methods such as recruitment via a public health care website. Illustrating the lack of work in the routine clinical setting, 54/57 trials (95%) did not report having been conducted in primary health care, and 3/57 (5%) reported having been conducted in primary health care. The trials were based in Australia 15/57 (26%), Canada 12/57 (21%), United States 6/57 (11%,), United Kingdom 5/57 (9%), Sweden 4/57 (7%), the Netherlands 3/57 (5%), and other countries 12/57 (21%). The most common self-report questionnaire measuring depression symptoms was the Patient Health Questionnaire 9 (PHQ-9) 31/57 (54%), followed by the Depression, Anxiety and Depression Scale 21 (DASS-21) 11/57 (19%). The most common anxiety measure was the GAD-7 32/57 (56%), followed by the DASS-21 11/57 (19%), and the Hospital Anxiety and Depression Scale - Anxiety subscale (HADS-A) 6/57 (11%). More trial characteristics are tabulated in the online Supplement B.

<*Study risk of bias, and quality of the evidence base*>

The distribution of study risk-of-bias ratings is presented in Figure 2. Overall, 9/57 (16%) of the trials were rated as having a low risk of bias, 14/57 (25%) as raising some concerns, and 34/57 (60%) as having a high risk of bias. The most common reason for an overall high risk was missing outcome data, which was seen in 29/59 (51%) of the trials, followed by deviations from the intended interventions in 15/57 (26%). Pooled effect sizes did not differ between trials as a function of the overall risk of bias (see Table 1). In all outcome domains, the overall quality of the evidence base was very low (see Supplement C for details).

INSERT FIGURE 2 ABOUT HERE

For peer review only - http://bmjopen.bmj.com/site/about/guidelines.xhtml

<Effects on depression and anxiety>

For adults, the pooled within-group reduction in depression symptoms was large (g = 0.90; 95% CI 0.81-0.99; k = 78) and the heterogeneity was high (I^2 = 95%; τ^2 = 0.14; Q_{77} =1606, p = < 0.001). The pooled within-group reduction in anxiety was also large (g = 0.87; 95% CI 0.78-0.96; k = 78) and the heterogeneity was high (I^2 = 95%; τ^2 = 0.16; Q_{77} = 1882, p = < 0.001). The pooled between-group effect versus the rudimentary controls was moderate for depression symptoms (g = 0.52; 95% CI 0.42-0.63; k = 43) with high heterogeneity (I^2 = 80%; τ^2 = 0.08; Q_{42} = 162, p = < 0.001), and moderate for anxiety (g = 0.45; 95% CI 0.34-0.56; k = 43) with high heterogeneity (I^2 = 83%; τ^2 = 0.10; Q_{42}= 176, p = < 0.001). See Fig. 3. The pooled between-group effect versus the attention/engagement controls was also significant but small for depression symptoms (g = 0.30; 95% CI 0.07-0.53; k = 10; I^2 = 91%; τ^2 = 0.12; Q_9 = 48, p = < 0.001) and small for anxiety (g = 0.21; 95% CI 0.01-0.42; k = 10; I^2 = 88%; τ^2 = 0.09; Q_9 = 45, p = < 0.001). See Fig. 4.

INSERT FIGURE 3 AND 4 ABOUT HERE

<Risk of publication bias>

We explored indicators of publication bias in the meta-analysis of effects versus rudimentary passive controls. For depression, there was clear indication of small- to medium-sized trials with non-beneficial effects of internet-delivered psychological treatments being underreported, and with imputed studies the between-group effect dropped from 0.52 to 0.29 (95% CI 0.16-0.42). For anxiety, there were also indication of possible asymmetry in the same direction, but this was more ambiguous, and the trim-and-fill procedure did not result in any imputed trials. See tSupplement B for details.

For peer review only

For peer review only - http://bmjopen.bmj.com/site/about/guidelines.xhtml

<Moderators and subgroups>

The results of the planned moderator and subgroup analyses are presented in Table 1. With regard to between-group effects versus the rudimentary controls, one moderator was found to be significant for both depression and anxiety symptoms: trials that required all participants to meet full criteria for a psychiatric diagnosis reported a larger pooled between-group effect as compared to trials that did not have this requirement. We also identified 9 randomized factorial trials. These manipulated various aspects of the treatment format and reported mostly null results with small effect sizes. In single trials, small moderating effects were reported for therapist guidance, app recommendations, and pre-treatment motivational interviewing.(70 96) In other trials, null results were however also reported for guidance and motivational interviewing.(51 80)

Table 1. Moderator and subgroup analyses of the relative effect of Internet-delivered transdiagnostic psychological treatment versus rudimentary controls.

**DEPRESSION SYMPTOMS**								
**Potential moderator**	***Q (df)***	***p***	**Subgroup**	***k***	***g***	***95% CI***	***I^2***	***τ^2***
Pooled total				43	0.52	0.42-0.63	80%	0.08
Study and sample characteristics								
Study setting	2.14 (1)	0.144	Primary health care	4	0.30†	-0.03-0.62	79%	0.08
			Not or unclear	39	0.55	0.44-0.66		
Recruitment path	3.32 (1)	0.068	Routine care	8	0.32	0.07-0.57	81%	0.09
			Not routine care	31	0.58	0.45-0.71		
Main inclusion criterion	6.33 (1)	0.012*	Psychiatric diagnosis	12	0.74	0.54-0.94	78%	0.08
			Cut-off or symptoms	31	0.44	0.32-0.56		
Depression severity	2.31 (2)	0.315	Mild	5	0.46	0.18-0.74	68%	0.06
			Moderate	32	0.53	0.42-0.64		
			Severe	4	0.28†	-0.04-0.59		
Anxiety severity	1.29 (1)	0.255	Mild or moderate	30	0.46	0.35-0.56	74%	0.06

For peer review only - http://bmjopen.bmj.com/site/about/guidelines.xhtml

			Severe	10	0.59	0.38-0.79		
Treatment characteristics								
Delivery format	1.51 (2)	0.471	Website	29	0.50	0.38-0.63	79%	0.08
			Mobile app	6	0.43	0.09-0.77		
			Mixed or other	8	0.66	0.41-0.90		
Therapist support	0.91 (1)	0.341	Yes, guided	23	0.58	0.43-0.73	80%	0.08
			No, unguided	20	0.47	0.32-0.62		
Treatment type	4.67 (2)	0.097	CBT	29	0.53	0.41-0.66	77%	0.27
			Mindfulness	4	0.88	0.49-1.27		
			Other	10	0.40	0.21-0.60		
Specific protocols	0.06 (1)	0.812	Wellbeing course	6	0.49	0.21-0.78	81%	0.09
			Not wellbeing course	37	0.53	0.41-0.65		
Treatment length	0.02 (1)	0.901	≥6 weeks	28	0.53	0.40-0.66	79%	0.09
			<6 weeks	15	0.52	0.33-0.70		
Component flexibility	1.00 (1)	0.318	Standardized	36	0.56	0.44-0.68	81%	0.09
			Tailored	5	0.39	0.08-0.70		
Therapist time††	0.88 (1)	0.349	Per additional 10 min	28	0.00†	-0.01-0.01	63%	0.03
Overall study risk of bias								
Cochrane tool version 2	0.84 (2)	0.658	High	29	0.49	0.36-0.63	81%	0.09
			Some concerns	8	0.55	0.30-0.80		
			Low	6	0.63	0.35-0.91		
**ANXIETY**								
**Potential moderator**	***Q (df)***	***p***	**Subgroup**	***k***	***g***	***95% CI***	***I²***	**τ²**
Pooled total				43	0.45	0.34-0.56	83%	0.10
Study and sample characteristics								
Study setting	0.91 (1)	0.340	Primary health care	4	0.28†	-0.07-0.64	83%	0.10
			Not or unclear	39	0.47	0.35-0.59		
Recruitment path	2.19 (1)	0.139	Routine care	8	0.30	0.04-0.55	82%	0.10
			Not routine care	31	0.52	0.38-0.65		
Main inclusion criterion	7.01 (1)	0.008*	Psychiatric diagnosis	12	0.68	0.48-0.89	81%	0.09
			Cut-off or symptoms	31	0.36	0.23-0.48		
Depression severity	2.60 (2)	0.272	Mild	5	0.51	0.20-0.81	74%	0.08
			Moderate	32	0.44	0.32-0.56		
			Severe	4	0.16†	-0.18-0.50		
Anxiety severity	0.85 (1)	0.358	Mild or moderate	30	0.38	0.26-0.50	79%	0.07

For peer review only

			Severe	10	0.50	0.28-0.72		
Treatment characteristics								
Delivery format	0.02 (2)	0.988	Website	29	0.45	0.31-0.59	83%	0.11
			Mobile app	6	0.42	0.06-0.79		
			Mixed or other	8	0.46	0.19-0.73		
Therapist support	1.67 (1)	0.196	Yes, guided	23	0.52	0.36-0.68	82%	0.10
			No, unguided	20	0.38	0.22-0.53		
Treatment type	5.57 (2)	0.062	CBT	29	0.44	0.31-0.57	80%	0.29
			Mindfulness	4	0.89	0.48-1.30		
			Other	10	0.34	0.13-0.55		
Specific protocols	0.96 (1)	0.328	Wellbeing course	6	0.31	0.00-0.61	84%	0.10
			Not wellbeing course	37	0.47	0.35-0.60		
Treatment length	0.00 (1)	0.973	≥6 weeks	28	0.45	0.31-0.59	83%	0.11
			<6 weeks	15	0.45	0.25-0.66		
Component flexibility	2.22 (1)	0.136	Standardized	36	0.49	0.36-0.61	84%	0.11
			Tailored	5	0.22†	-0.11-0.55		
Therapist time††	0.47 (1)	0.494	Per additional 10 min	28	0.00†	-0.01-0.02	85%	0.09
Overall study risk of bias								
Cochrane tool version 2	0.01 (2)	0.995	High	29	0.45	0.31-0.60	84%	0.11
			Some concerns	8	0.44	0.17-0.71		
			Low	6	0.46	0.16-0.76		

In accordance with the study protocol, strata were collapsed or excluded from analysis if $k < 4$. For anxiety severity, "mild" ($k = 3$) and "moderate" were collapsed. For delivery formats, "video" ($k = 3$) was subsumed under "mixed or other". For treatment types, "CBT and mindfulness" ($k = 3$) was subsumed under "other". Q-tests listed in this table are tests of moderation, with the intercept included in the statistical model. Heterogeneity is reported as I^2 and τ^2. CBT, cognitive behavior therapy.

* Test of moderation significant at $\alpha = 0.05$.

† Pooled effect not significantly different from zero.

†† Therapist time per participant was tested as a continuous variable.

For peer review only

For peer review only - http://bmjopen.bmj.com/site/about/guidelines.xhtml

<Effects versus other bona fide treatments>

Three trials compared the effects of an internet-delivered transdiagnostic psychological treatment to those of a distinctly different bona fide treatment. These trials showed mixed results and are described in Supplement B.(49) (79) (91)

<Children, adolescents, and older adults>

Two trials recruited children or adolescents. One trial recruited ages 12-21 (M=16, SD=2; N=45) and reported null effects of 5 weeks of problem-solving therapy on depression (g = 0.04) and anxiety (g = -0.11) as compared to a waitlist.(62) The second trial recruited ages 13-18 (M=16, SD=1; N=103) in a school setting and found that a single session promoting growth, gratitude, and values had significant and moderate added effects on depression (g = 0.50) but not anxiety (g = 0.32) as compared to a control that focused on the promotion of study skills.(74) One trial exclusively recruited older adults, aged 60+ (M=66, SD=5; N=433), and found that 8 weeks of CBT in a structured text-based self-help format was associated with large within-group reductions in depression (gs = 1.19-1.30) and anxiety (gs = 1.21-1.31), but that there was no significant added effect of adding guidance or an initial interview.(85)

For peer review only

<Discussion>

In this systematic review with meta-analysis based on 57 randomized controlled trials we found that, for adults, internet-delivered transdiagnostic psychological treatments led to moderate between-group effects compared to rudimentary controls (depression: g = 0.52; anxiety: g = 0.45) and small between-group effects compared to attention/engagement controls (depression: g = 0.30; anxiety: g = 0.21). Heterogeneity was substantial. There were

For peer review only - http://bmjopen.bmj.com/site/about/guidelines.xhtml

also indications of possible publication bias, which probably inflated the pooled effect on depression versus rudimentary controls – perhaps as much as 0.23 units. One significant moderator was found: trials where all participants were required to meet full diagnostic criteria for depression or an anxiety disorder reported larger between-group effects versus rudimentary controls, than did trials without this requirement. Strengths of the present study include the systematic search strategy and the broad eligibility criteria, which allowed for an overview of treatment frameworks, formats, settings including primary health care, and also age groups. On the whole, the overall quality of the evidence base was very low, which implies that there is considerable uncertainty about true effects.

<Comparison with prior research>

Our final sample of 57 RCTs pertaining specifically to internet-delivered transdiagnostic psychological treatments suitable for both primary depression and primary anxiety disorders is large compared to previous systematic reviews.(15) This is probably explained both by the rapid growth of this research field, and our use of exhaustive search terms developed with the specific aim of identifying internet-delivered transdiagnostic psychological treatments. The between-group effect versus rudimentary controls was slightly smaller than expected, though still broadly in line with previous meta-analyses.(15 16) Our expectation, based on previous research,(17) that treatment length would be of little importance for the between-group effect was also supported in our data. Similarly, the finding that effects on depression are likely to be inflated by publication bias is also in line with previous research and therefore not unexpected.(103)

For peer review only - http://bmjopen.bmj.com/site/about/guidelines.xhtml

<Children, adolescents, and older adults>

This exhaustive literature search showed very clearly that the internet-delivered transdiagnostic treatments for children and adolescents, as well as specifically for older adults, have not come very far. Especially for children and adolescents, we found this rather surprising considering the relatively long tradition of treatments intended to suit a wide spectrum of anxiety-, but not necessarily mood, disorders.(104) Conceivably, this may have reduced the incentive to develop newer protocols that can accommodate patients with depression as well. For older adults, we identified a single study which presented promising results. It is widely acknowledged that older adults rarely receive psychological treatment(105 106). We wish to encourage further work in this area, especially with increased technical prowess in this population.

<Possible benefit of meeting a diagnosis>

In the exploratory moderator analyses, the only variable that was a significant moderator of the between-group effect of treatment was whether participants were required to meet full criteria for a psychiatric diagnosis. One interpretation is that of a “specificity effect”, where larger effects can be expected if patients suffer from clinical depression or an anxiety disorder, as opposed to symptoms caused for example by a somatoform disorder or a somatic disease.

<Treatment characteristics>

Except for the possible benefits of a diagnosis, a general pattern in both the planned tests of potential moderators and the original factorial trials was the lack of significant moderators, not least in terms of treatment characteristics. One interesting finding was for example that therapist-guidance was not found to be a significant moderator in our meta-analyses, and that

For peer review only

For peer review only - http://bmjopen.bmj.com/site/about/guidelines.xhtml

results from the factorial trials were inconsistent. We found that it is still unusual for trials to investigate the effect of treatments other than CBT or mindfulness-based interventions. There were, however, also examples of other treatments investigated in more than one trial, namely problem-solving therapies(62 65 89) and bias-modification training therapies.(61 97)

<New formats: Are we at a crossroads?>

For the past quarter of a century, internet-delivered psychological treatments have mostly been delivered via web platforms, with standardized linear text presentations intended to be accessed via a web browser. This systematic review illustrates that this treatment format is still most widely researched, and that newer formats remain understudied. We were able to pool the mean effects of 6 arms from 5 trials focusing on mobile apps, and 5 arms from 5 trials of tailored treatments, which showed no significant differences compared to the more common standardized web format. Notably, only three trials investigated videoconferencing interventions(86 93 100), and only one trial investigated the effect of an intervention using artificial intelligence(54). The vast majority of the literature still pertains to the standardized web-based text format, but the direction of the field appears to be towards automated, tailored, interventions.

<Limitations>

A clear limitation of the original studies was that the majority of trials recruited self-referred participants through advertisement and were not conducted in clinical setting. The fact that these samples might have been highly motivated, and not representative of patients in routine care, makes it more difficult to draw conclusions about effectiveness in routine care. Another limitation of this review is that our analyses of moderators were based exclusively on trial-level characteristics. This implies poor power, and often restriction of range, in many tests

For peer review only - http://bmjopen.bmj.com/site/about/guidelines.xhtml

such as that of whether pooled effects differ as a function of mean baseline symptom level. This review included studies published in English only, which means that RCTs published solely in other languages were systematically overlooked. Another aspect that warranted lowered GRADE ratings was the considerable heterogeneity in reported effects, which remained largely unexplained and often implied poor precision. Last, we remind the reader that being enrolled in a psychological treamtent implies engaging with the content of that treatment, which often includes its theoretical basis. Therefore, therapists and patients cannot be fully blinded to the intervention, and effects may depend partially on expectancy effects, including therapist's and patient's presumptions about "what ought to work".

<Clinical implications and recommendations for future research>

This meta-analysis contributes tentative evidence that transdiagnostic psychological treatments are effective for people with elevated depression symptoms or anxiety. However, even though a substantial proportion of patients are treated in routine care settings such as primary care,(6 107) only three (5%) of the 57 included trials were conducted in a primary care setting. Power was limited to quantify the pooled effect of this stratum, and *g* did not reach statistical significance. This means that there is an urgent need for RCTs evaluating internet-delivered transdiagnostic treatments based on a broad recruitment strategies in the routine clinical context. It is also important when planning for future RCTs to reduce currently high levels of missing data. More studies evaluating video-delivered interventions are needed. Adequately powered RCTs comparing internet-delivered transdiagnostic treatments with other bona fide treatments for depression and anxiety are also needed.

For peer review only - http://bmjopen.bmj.com/site/about/guidelines.xhtml

<Conclusion>

Internet-delivered transdiagnostic treatments suitable for both depression and anxiety appear to have moderate effects compared to rudimentary controls, and significant but small effects beyond those of attention/engagement controls. Evidence is lacking with regard to routine care, the video format, children and adolescents, and older adults.

For peer review only

For peer review only - http://bmjopen.bmj.com/site/about/guidelines.xhtml

Acknowledgements: We thank the Karolinska Institutet library service, and in particular Sabina Gillsund and Emma-Lotta Säätelä, for contributing to the systematic database search. We would also like to thank all study authors who responded to our queries and contributed with data on request.

Competing interests: KK, AHB, EHL, EL, JH, and EA have taken part in original research on internet-delivered treatment including transdiagnostic treatment.

Contributions of authors: KK and EA led the study design and data collection process, with input from all authors. KK and EA conducted the statistical analysis. KK wrote the initial draft in close collaboration with EA. All authors reviewed the manuscript for intellectual content, made contributions, and approved the final version. KK and EA had full access to all data and take responsibility for the integrity of the data and the accuracy of the presented analysis.

Funding: This work was supported by the Swedish Research Council (EA; identifier: 2021-06496) and the Krica Foundation. Neither of the funders had any role in the design, execution, or publication process.

Availability of data: Tabulated data is available from the authors on request.

Ethics Approval: Not applicable

Karoline Kolaas, PhD, Karolinska Institutet, Dept of Clin. Neuroscience, Stockholm, Sweden, **Anne H Berman,** PhD, Uppsala University, Dept of Psychology, Uppsala, Sweden,

For peer review only

For peer review only - http://bmjopen.bmj.com/site/about/guidelines.xhtml

Erik Hedman-Lagerlöf, PhD, Karolinska Institutet, Dept of Clin. Neuroscience, Stockholm, Sweden, **Elin Lindsäter**, PhD, Karolinska Institutet, Dept of Clin. Neuroscience, Stockholm, Sweden, **Jonna Hybelius**, MSc, Karolinska Institutet, Dept of Neurobiology, Care Sciences and Society, Stockholm, Sweden, & **Erland Axelsson**, PhD, Karolinska Institutet, Dept of Neurobiology, Care Sciences and Society, Stockholm, Sweden

For peer review only

For peer review only - http://bmjopen.bmj.com/site/about/guidelines.xhtml

Figure captions:

Figure 1 PRISMA flowchart of the study selection process. RCT, randomized controlled trial; SD, standard deviation.

Figure 2 Study risk of bias based on the Cochrane Risk of Bias Tool version 2. Note that blinding was not assessed, which means that the lack of blinding did not result in an elevated risk score.

Figure 3 Standardized effect on depression and anxiety symptoms vs rudimentary passive controls.

Figure 4 Standardized effect on depressioin and anxiety symptoms vs attention/engagement controls.

For peer review only

For peer review only - http://bmjopen.bmj.com/site/about/guidelines.xhtml

References

1. GBD 2019 Mental Disorders Collaborators. Global, regional, and national comparative risk assessment of 84 behavioural, environmental and occupational, and metabolic risks or clusters of risks for 195 countries and territories, 1990-2017: a systematic analysis for the Global Burden of Disease Study 2017. *Lancet* 2018;392(10159):1923-94. doi: 10.1016/s0140-6736(18)32225-6 [published Online First: 20181108]

2. Bandelow B, Michaelis S. Epidemiology of anxiety disorders in the 21st century. *Dialogues Clin Neurosci* 2015;17(3):327-35. doi: 10.31887/DCNS.2015.17.3/bbandelow

3. Cuijpers P, Cristea IA, Karyotaki E, et al. How effective are cognitive behavior therapies for major depression and anxiety disorders? A meta-analytic update of the evidence. *World Psychiatry* 2016;15(3):245-58. doi: 10.1002/wps.20346 [published Online First: 2016/10/08]

4. Santoft F, Axelsson E, Öst LG, et al. Cognitive behaviour therapy for depression in primary care: systematic review and meta-analysis. *Psychological medicine* 2019:1-9. doi: 10.1017/S0033291718004208 [published Online First: 2019/01/29]

5. Twomey C, O'Reilly G, Byrne M. Effectiveness of cognitive behavioural therapy for anxiety and depression in primary care: a meta-analysis. *Family practice* 2015;32(1):3-15. doi: 10.1093/fampra/cmu060 [published Online First: 20140922]

6. Alonso J, Angermeyer MC, Bernert S, et al. Use of mental health services in Europe: results from the European Study of the Epidemiology of Mental Disorders (ESEMeD) project. *Acta psychiatrica Scandinavica Supplementum* 2004(420):47-54. doi: 10.1111/j.1600-0047.2004.00330.x [published Online First: 2004/05/07]

For peer review only - http://bmjopen.bmj.com/site/about/guidelines.xhtml

7. Thornicroft G, Chatterji S, Evans-Lacko S, et al. Undertreatment of people with major depressive disorder in 21 countries. *Br J Psychiatry* 2017;210(2):119-24. doi: 10.1192/bjp.bp.116.188078 [published Online First: 20161201]

8. Alonso J, Liu Z, Evans-Lacko S, et al. Treatment gap for anxiety disorders is global: Results of the World Mental Health Surveys in 21 countries. *Depression and anxiety* 2018;35(3):195-208. doi: 10.1002/da.22711 [published Online First: 20180122]

9. McGrath JJ, Lim CCW, Plana-Ripoll O, et al. Comorbidity within mental disorders: a comprehensive analysis based on 145 990 survey respondents from 27 countries. *Epidemiol Psychiatr Sci* 2020;29:e153. doi: 10.1017/s2045796020000633 [published Online First: 20200812]

10. Jacobson NC, Newman MG. Anxiety and depression as bidirectional risk factors for one another: A meta-analysis of longitudinal studies. *Psychol Bull* 2017;143(11):1155-200. doi: 10.1037/bul0000111 [published Online First: 20170814]

11. Sloan E, Hall K, Moulding R, et al. Emotion regulation as a transdiagnostic treatment construct across anxiety, depression, substance, eating and borderline personality disorders: A systematic review. *Clinical psychology review* 2017;57:141-63. doi: 10.1016/j.cpr.2017.09.002 [published Online First: 20170911]

12. Wilamowska ZA, Thompson-Hollands J, Fairholme CP, et al. Conceptual background, development, and preliminary data from the unified protocol for transdiagnostic treatment of emotional disorders. *Depression and anxiety* 2010;27(10):882-90. doi: 10.1002/da.20735

13. Ringwald WR, Forbes MK, Wright AGC. Meta-analysis of structural evidence for the Hierarchical Taxonomy of Psychopathology (HiTOP) model. *Psychological medicine* 2023;53(2):533-46. doi: 10.1017/S0033291721001902 [published Online First: 2021/05/14]

For peer review only

For peer review only - http://bmjopen.bmj.com/site/about/guidelines.xhtml

14. Kolaas K, Berman AH, Hedman-Lagerlöf E, et al. Feasibility of a video-delivered mental health course for primary care patients: a single-group prospective cohort study. *BMC Prim Care* 2023;24(1):28. doi: 10.1186/s12875-023-01989-8 [published Online First: 20230123]

15. Cuijpers P, Miguel C, Ciharova M, et al. Transdiagnostic treatment of depression and anxiety: a meta-analysis. *Psychological medicine* 2023:1-12.

16. Newby JM, Twomey C, Yuan Li SS, et al. Transdiagnostic computerised cognitive behavioural therapy for depression and anxiety: A systematic review and meta-analysis. *Journal of affective disorders* 2016;199:30-41. doi: https://doi.org/10.1016/j.jad.2016.03.018

17. Păsărelu CR, Andersson G, Bergman Nordgren L, et al. Internet-delivered transdiagnostic and tailored cognitive behavioral therapy for anxiety and depression: a systematic review and meta-analysis of randomized controlled trials. *Cogn Behav Ther* 2017;46(1):1-28. doi: 10.1080/16506073.2016.1231219 [published Online First: 20161007]

18. Thompson EM, Destree L, Albertella L, et al. Internet-based acceptance and commitment therapy: a transdiagnostic systematic review and meta-analysis for mental health outcomes. *Behavior therapy* 2021;52(2):492-507.

19. Etzelmueller A, Vis C, Karyotaki E, et al. Effects of Internet-Based Cognitive Behavioral Therapy in Routine Care for Adults in Treatment for Depression and Anxiety: Systematic Review and Meta-Analysis. *Journal of medical Internet research* 2020;22(8):e18100. doi: 10.2196/18100 [published Online First: 20200831]

20. Page MJ, McKenzie JE, Bossuyt PM, et al. The PRISMA 2020 statement: an updated guideline for reporting systematic reviews. *BMJ (Clinical research ed)* 2021;372:n71. doi: 10.1136/bmj.n71 [published Online First: 20210329]

For peer review only - http://bmjopen.bmj.com/site/about/guidelines.xhtml

21. Bramer W, Bain P. Updating search strategies for systematic reviews using EndNote. *J Med Libr Assoc* 2017;105(3):285-89. doi: 10.5195/jmla.2017.183 [published Online First: 20170701]

22. Bramer WM, Giustini D, de Jonge GB, et al. De-duplication of database search results for systematic reviews in EndNote. *J Med Libr Assoc* 2016;104(3):240-3. doi: 10.3163/1536-5050.104.3.014

23. Ouzzani M, Hammady H, Fedorowicz Z, et al. Rayyan-a web and mobile app for systematic reviews. *Systematic reviews* 2016;5(1):210. doi: 10.1186/s13643-016-0384-4 [published Online First: 20161205]

24. Bolland MJ, Avenell A, Grey A. Analysis of cluster randomised trials as if they were individually randomised. *Lancet Diabetes Endocrinol* 2023;11(2):75. doi: 10.1016/s2213-8587(22)00363-1 [published Online First: 20221219]

25. Crawford J, Cayley C, Lovibond PF, et al. Percentile norms and accompanying interval estimates from an Australian general adult population sample for self‐report mood scales (BAI, BDI, CRSD, CES‐D, DASS, DASS‐21, STAI‐X, STAI‐Y, SRDS, and SRAS). *Australian Psychologist* 2011;46(1):3-14.

26. Crawford JR, Garthwaite PH, Lawrie CJ, et al. A convenient method of obtaining percentile norms and accompanying interval estimates for self‐report mood scales (DASS, DASS‐21, HADS, PANAS, and sAD). *British Journal of Clinical Psychology* 2009;48(2):163-80.

27. Schalet BD, Cook KF, Choi SW, et al. Establishing a common metric for self-reported anxiety: linking the MASQ, PANAS, and GAD-7 to PROMIS Anxiety. *Journal of anxiety disorders* 2014;28(1):88-96.

For peer review only

28. Wahl I, Löwe B, Bjorner JB, et al. Standardization of depression measurement: a common metric was developed for 11 self-report depression measures. *Journal of clinical epidemiology* 2014;67(1):73-86.

29. Munder T, Geisshüsler A, Krieger T, et al. Intensity of Treatment as Usual and Its Impact on the Effects of Face-to-Face and Internet-Based Psychotherapy for Depression: A Preregistered Meta-Analysis of Randomized Controlled Trials. *Psychother Psychosom* 2022;91(3):200-09. doi: 10.1159/000521951 [published Online First: 20220214]

30. Wampold BE, Mondin GW, Moody M, et al. A meta-analysis of outcome studies comparing bona fide psychotherapies: Empiricially," all must have prizes.". *Psychological bulletin* 1997;122(3):203.

31. Sterne JAC, Savović J, Page MJ, et al. RoB 2: a revised tool for assessing risk of bias in randomised trials. *BMJ (Clinical research ed)* 2019;366:l4898. doi: 10.1136/bmj.l4898 [published Online First: 20190828]

32. Bisby MA, Karin E, Hathway T, et al. A meta-analytic review of randomized clinical trials of online treatments for anxiety: Inclusion/exclusion criteria, uptake, adherence, dropout, and clinical outcomes. *Journal of anxiety disorders* 2022;92:102638. doi: 10.1016/j.janxdis.2022.102638 [published Online First: 20221007]

33. GRADE. Grading quality of evidence and strength of recommendations. *BMJ (Clinical research ed)* 2004;328(7454):1490.

34. R Core Team. R: A Language and Environment for Statistical Computing. R Foundation for Statistical Computing, Vienna, Austria. 2016

35. Viechtbauer W. Conducting meta-analyses in R with the metafor package. *Journal of statistical software* 2010;36(3):1-48.

For peer review only - http://bmjopen.bmj.com/site/about/guidelines.xhtml

36. Hedges LV. Distribution theory for Glass's estimator of effect size and related estimators. *journal of Educational Statistics* 1981;6(2):107-28.

37. Becker BJ. Synthesizing standardized mean ‐ change measures. *British Journal of Mathematical and Statistical Psychology* 1988;41(2):257-78.

38. Morris SB. Distribution of the standardized mean change effect size for meta ‐ analysis on repeated measures. *British Journal of Mathematical and Statistical Psychology* 2000;53(1):17-29.

39. Hedges LV. Estimation of effect size from a series of independent experiments. *Psychological bulletin* 1982;92(2):490.

40. Cohen J. Statistical power analysis for the behavioral science. 2 ed: Hillsdale, NJ: Lawrence Erlbaum Associates 1988:24-27.

41. Higgins JP, Thompson SG, Deeks JJ, et al. Measuring inconsistency in meta-analyses. *BMJ* 2003;327(7414):557-60. doi: 10.1136/bmj.327.7414.557 [published Online First: 2003/09/06]

42. Rücker G, Schwarzer G, Carpenter JR, et al. Undue reliance on I 2 in assessing heterogeneity may mislead. *BMC medical research methodology* 2008;8:1-9.

43. Egger M, Davey Smith G, Schneider M, et al. Bias in meta-analysis detected by a simple, graphical test. *BMJ (Clinical research ed)* 1997;315(7109):629-34. doi: 10.1136/bmj.315.7109.629

44. Duval S, Tweedie R. Trim and fill: A simple funnel-plot-based method of testing and adjusting for publication bias in meta-analysis. *Biometrics* 2000;56(2):455-63. doi: 10.1111/j.0006-341x.2000.00455.x

45. Shi L, Lin L. The trim-and-fill method for publication bias: practical guidelines and recommendations based on a large database of meta-analyses. *Medicine* 2019;98(23)

For peer review only

For peer review only - http://bmjopen.bmj.com/site/about/guidelines.xhtml

46. Al-Refae M, Al-Refae A, Munroe M, et al. A self-compassion and mindfulness-based cognitive mobile intervention (Serene) for depression, anxiety, and stress: promoting adaptive emotional regulation and wisdom. *Frontiers in psychology* 2021;12:648087.

47. Batterham PJ, Calear AL, Farrer L, et al. Efficacy of a transdiagnostic self-help internet intervention for reducing depression, anxiety, and suicidal ideation in adults: randomized controlled trial. *Journal of medical Internet research* 2021;23(1):e22698.

48. Beshai S, Bueno C, Yu M, et al. Examining the effectiveness of an online program to cultivate mindfulness and self-compassion skills (Mind-OP): Randomized controlled trial on Amazon's Mechanical Turk. *Behaviour research and therapy* 2020;134:103724.

49. Borjalilu S, Mazaheri MA, Talebpour A. Effectiveness of mindfulness-based stress management in the mental health of Iranian university students: a comparison of blended therapy, face-to-face sessions, and mHealth app (Aramgar). *Iranian Journal of Psychiatry and Behavioral Sciences* 2019;13(2)

50. Carolan S, Harris PR, Greenwood K, et al. Increasing engagement with an occupational digital stress management program through the use of an online facilitated discussion group: Results of a pilot randomised controlled trial. *Internet interventions* 2017;10:1-11.

51. Dear BF, Fogliati VJ, Fogliati R, et al. Treating anxiety and depression in young adults: A randomised controlled trial comparing clinician-guided versus self-guided Internet-delivered cognitive behavioural therapy. *Aust N Z J Psychiatry* 2018;52(7):668-79. doi: 10.1177/0004867417738055 [published Online First: 20171024]

52. Díaz-García A, González-Robles A, García-Palacios A, et al. Negative and positive affect regulation in a transdiagnostic Internet-based protocol for emotional disorders: randomized controlled trial. *Journal of medical Internet research* 2021;23(2):e21335.

For peer review only - http://bmjopen.bmj.com/site/about/guidelines.xhtml

53. Farrer LM, Gulliver A, Katruss N, et al. A novel multi-component online intervention to improve the mental health of university students: Randomised controlled trial of the Uni Virtual Clinic. *Internet Interv* 2019;18:100276. doi: 10.1016/j.invent.2019.100276 [published Online First: 20190828]

54. Fitzpatrick KK, Darcy A, Vierhile M. Delivering cognitive behavior therapy to young adults with symptoms of depression and anxiety using a fully automated conversational agent (Woebot): a randomized controlled trial. *JMIR mental health* 2017;4(2):e7785.

55. González-Robles A, Díaz-García A, García-Palacios A, et al. Effectiveness of a transdiagnostic guided internet-delivered protocol for emotional disorders versus treatment as usual in specialized care: randomized controlled trial. *Journal of medical Internet research* 2020;22(7):e18220.

56. Graham AK, Greene CJ, Kwasny MJ, et al. Coached Mobile App Platform for the Treatment of Depression and Anxiety Among Primary Care Patients: A Randomized Clinical Trial. *JAMA psychiatry* 2020;77(9):906-14. doi: 10.1001/jamapsychiatry.2020.1011

57. Hadjistavropoulos HD, Schneider LH, Edmonds M, et al. Randomized controlled trial of internet-delivered cognitive behaviour therapy comparing standard weekly versus optional weekly therapist support. *Journal of anxiety disorders* 2017;52:15-24. doi: 10.1016/j.janxdis.2017.09.006 [published Online First: 20170923]

58. Hadjistavropoulos HD, Peynenburg V, Nugent M, et al. Transdiagnostic Internet-delivered cognitive behaviour therapy with therapist support offered once-weekly or once-weekly supplemented with therapist support within one-business-day: Pragmatic randomized controlled trial. *Internet Interv* 2020;22:100347. doi: 10.1016/j.invent.2020.100347 [published Online First: 20200831]

For peer review only

59. Hadjistavropoulos HD, Peynenburg V, Thiessen DL, et al. A pragmatic factorial randomized controlled trial of transdiagnostic internet-delivered cognitive behavioural therapy: Exploring benefits of homework reflection questionnaires and twice-weekly therapist support. *Internet Interv* 2020;22:100357. doi: 10.1016/j.invent.2020.100357 [published Online First: 20201201]

60. Hensel JM, Shaw J, Ivers NM, et al. A web-based mental health platform for individuals seeking specialized mental health care services: multicenter pragmatic randomized controlled trial. *Journal of medical Internet research* 2019;21(6):e10838.

61. Hirsch CR, Krahé C, Whyte J, et al. Effects of modifying interpretation bias on transdiagnostic repetitive negative thinking. *Journal of consulting and clinical psychology* 2020;88(3):226.

62. Hoek W, Schuurmans J, Koot HM, et al. Effects of Internet-based guided self-help problem-solving therapy for adolescents with depression and anxiety: a randomized controlled trial. *PLoS One* 2012;7(8):e43485. doi: 10.1371/journal.pone.0043485 [published Online First: 20120831]

63. Johansson R, Björklund M, Hornborg C, et al. Affect-focused psychodynamic psychotherapy for depression and anxiety through the Internet: a randomized controlled trial. *PeerJ* 2013;1:e102.

64. Kladnitski N, Smith J, Uppal S, et al. Transdiagnostic internet-delivered CBT and mindfulness-based treatment for depression and anxiety: A randomised controlled trial. *Internet Interv* 2020;20:14. doi: 10.1016/j.invent.2020.100310

65. Kleiboer A, Donker T, Seekles W, et al. A randomized controlled trial on the role of support in Internet-based problem solving therapy for depression and anxiety. *Behaviour research and therapy* 2015;72:63-71.

For peer review only - http://bmjopen.bmj.com/site/about/guidelines.xhtml

66. Krafft J, Potts S, Schoendorff B, et al. A randomized controlled trial of multiple versions of an acceptance and commitment therapy matrix app for well-being. *Behavior Modification* 2019;43(2):246-72.

67. Levin ME, Hicks ET, Krafft J. Pilot evaluation of the stop, breathe & think mindfulness app for student clients on a college counseling center waitlist. *Journal of American college health* 2022;70(1):165-73.

68. Lindegaard T, Seaton F, Halaj A, et al. Internet-based cognitive behavioural therapy for depression and anxiety among Arabic-speaking individuals in Sweden: a pilot randomized controlled trial. *Cognitive Behaviour Therapy* 2021;50(1):47-66.

69. McCloud T, Jones R, Lewis G, et al. Effectiveness of a mobile app intervention for anxiety and depression symptoms in university students: randomized controlled trial. *JMIR mHealth and uHealth* 2020;8(7):e15418.

70. Mohr DC, Schueller SM, Tomasino KN, et al. Comparison of the Effects of Coaching and Receipt of App Recommendations on Depression, Anxiety, and Engagement in the IntelliCare Platform: Factorial Randomized Controlled Trial. *Journal of medical Internet research* 2019;21(8):e13609. doi: 10.2196/13609 [published Online First: 20190828]

71. Mullin A, Dear BF, Karin E, et al. The UniWellbeing course: A randomised controlled trial of a transdiagnostic internet-delivered cognitive behavioural therapy (CBT) programme for university students with symptoms of anxiety and depression. *Internet Interventions* 2015;2(2):128-36.

72. Newby JM, Mackenzie A, Williams AD, et al. Internet cognitive behavioural therapy for mixed anxiety and depression: a randomized controlled trial and evidence of effectiveness in primary care. *Psychological medicine* 2013;43(12):2635-48.

For peer review only - http://bmjopen.bmj.com/site/about/guidelines.xhtml

73. Nilsson A, Sörman K, Klingvall J, et al. MyCompass in a Swedish context–lessons learned from the transfer of a self-guided intervention targeting mental health problems. *BMC psychiatry* 2019;19:1-11.

74. Osborn TL, Rodriguez M, Wasil AR, et al. Single-session digital intervention for adolescent depression, anxiety, and well-being: Outcomes of a randomized controlled trial with Kenyan adolescents. *Journal of consulting and clinical psychology* 2020;88(7):657-68. doi: 10.1037/ccp0000505 [published Online First: 20200511]

75. Powell J, Hamborg T, Stallard N, et al. Effectiveness of a web-based cognitive-behavioral tool to improve mental well-being in the general population: randomized controlled trial. *Journal of medical Internet research* 2013;15(1):e2.

76. Proudfoot J, Clarke J, Birch M-R, et al. Impact of a mobile phone and web program on symptom and functional outcomes for people with mild-to-moderate depression, anxiety and stress: a randomised controlled trial. *BMC psychiatry* 2013;13(1):1-12.

77. Robichaud M, Talbot F, Titov N, et al. Facilitating access to iCBT: a randomized controlled trial assessing a translated version of an empirically validated program using a minimally monitored delivery model. *Behavioural and Cognitive Psychotherapy* 2020;48(2):185-202.

78. Rollman BL, Belnap BH, Abebe KZ, et al. Effectiveness of online collaborative care for treating mood and anxiety disorders in primary care: a randomized clinical trial. *JAMA psychiatry* 2018;75(1):56-64.

79. Sethi S. Treating Youth Depression and Anxiety: A Randomised Controlled Trial Examining the Efficacy of Computerised versus Face‐to‐face Cognitive Behaviour Therapy. *Australian Psychologist* 2013;48(4):249-57. doi: 10.1111/ap.12006

For peer review only - http://bmjopen.bmj.com/site/about/guidelines.xhtml

80. Soucy JN, Hadjistavropoulos HD, Karin E, et al. Brief online motivational interviewing pre-treatment intervention for enhancing internet-delivered cognitive behaviour therapy: A randomized controlled trial. *Internet Interv* 2021;25:100394. doi: 10.1016/j.invent.2021.100394 [published Online First: 20210420]

81. Southwell S, Gould E. A randomised wait list-controlled pre–post–follow-up trial of a gratitude diary with a distressed sample. *The Journal of Positive Psychology* 2017;12(6):579-93.

82. Terides MD, Dear BF, Fogliati VJ, et al. Increased skills usage statistically mediates symptom reduction in self-guided internet-delivered cognitive–behavioural therapy for depression and anxiety: a randomised controlled trial. *Cognitive behaviour therapy* 2018;47(1):43-61.

83. Titov N, Dear BF, Johnston L, et al. Improving adherence and clinical outcomes in self-guided internet treatment for anxiety and depression: randomised controlled trial. *PloS one* 2013;8(7):e62873.

84. Titov N, Dear BF, Schwencke G, et al. Transdiagnostic internet treatment for anxiety and depression: a randomised controlled trial. *Behaviour research and therapy* 2011;49(8):441-52.

85. Titov N, Fogliati VJ, Staples LG, et al. Treating anxiety and depression in older adults: randomised controlled trial comparing guided v. self-guided internet-delivered cognitive–behavioural therapy. *BJPsych open* 2016;2(1):50-58.

86. Tönnies J, Hartmann M, Wensing M, et al. Mental health specialist video consultations versus treatment-as-usual for patients with depression or anxiety disorders in primary care: randomized controlled feasibility trial. *JMIR mental health* 2021;8(3):e22569.

For peer review only - http://bmjopen.bmj.com/site/about/guidelines.xhtml

87. Tulbure BT, Rusu A, Sava FA, et al. A web-based transdiagnostic intervention for affective and mood disorders: randomized controlled trial. *JMIR mental health* 2018;5(2):e8901.

88. Twomey C, O'Reilly G, Byrne M, et al. A randomized controlled trial of the computerized CBT programme, MoodGYM, for public mental health service users waiting for interventions. *British Journal of Clinical Psychology* 2014;53(4):433-50.

89. van Straten A, Cuijpers P, Smits N. Effectiveness of a web-based self-help intervention for symptoms of depression, anxiety, and stress: randomized controlled trial. *Journal of medical Internet research* 2008;10(1):e7.

90. Viskovich S, Pakenham KI. Randomized controlled trial of a web - based Acceptance and Commitment Therapy (ACT) program to promote mental health in university students. *Journal of clinical psychology* 2020;76(6):929-51.

91. Villemaire-Krajden R, Myhr G. Evaluating the use of a computerized CBT program for outpatients on a waitlist in a university CBT unit. *Journal of Psychiatric Practice®* 2019;25(4):268-78.

92. Zetterqvist K, Maanmies J, Ström L, et al. Randomized controlled trial of internet-based stress management. *Cognitive Behaviour Therapy* 2003;32(3):151-60.

93. Grégoire S, Beaulieu F, Lachance L, et al. An online peer support program to improve mental health among university students: A randomized controlled trial. *Journal of American College Health* 2022:1-13.

94. Hadjistavropoulos HD, Peynenburg V, Thiessen DL, et al. A randomized factorial trial of internet-delivered cognitive behavioural therapy: An 8-week program with or without extended support and booster lesson. *Internet Interv* 2022;27:100499. doi: 10.1016/j.invent.2022.100499 [published Online First: 20220206]

For peer review only - http://bmjopen.bmj.com/site/about/guidelines.xhtml

95. Isbăşoiu AB, Tulbure BT, Rusu A, et al. Can We Boost Treatment Adherence to an Online Transdiagnostic Intervention by Adding Self-Enhancement Strategies? Results From a Randomized Controlled Non-inferiority Trial. *Frontiers in psychology* 2021;12:752249. doi: 10.3389/fpsyg.2021.752249 [published Online First: 20211202]

96. Peynenburg V, Hadjistavropoulos H, Thiessen D, et al. Internet-Delivered Cognitive Behavioral Therapy for Postsecondary Students: Randomized Factorial Trial for Examining Motivational Interviewing and Booster Lessons. *Journal of medical Internet research* 2022;24(9):e40637. doi: 10.2196/40637 [published Online First: 20220907]

97. Rogers J, Sicouri G. A Single-Session Online Cognitive Bias Modification of Interpretations Modified for Adults With Anxiety and Depressive Symptoms. *Behavior therapy* 2022;53(5):967-80. doi: 10.1016/j.beth.2022.04.006 [published Online First: 20220419]

98. Schaeuffele C, Homeyer S, Perea L, et al. The unified protocol as an internet-based intervention for emotional disorders: Randomized controlled trial. *PloS One* 2022;17(7):e0270178.

99. Taylor H, Cavanagh K, Field AP, et al. Health Care Workers' Need for Headspace: Findings From a Multisite Definitive Randomized Controlled Trial of an Unguided Digital Mindfulness-Based Self-help App to Reduce Healthcare Worker Stress. *JMIR mHealth and uHealth* 2022;10(8):e31744.

100. Hosseinzadeh Asl NR. A randomized controlled trial of a mindfulness-based intervention in social workers working during the COVID-19 crisis. *Current Psychology* 2022;41(11):8192-99.

101. Kang MY, Nan JK, Yuan Y. Effectiveness of an online short-term audio-based mindfulness program on negative emotions during the COVID-19 pandemic: Latent

For peer review only - http://bmjopen.bmj.com/site/about/guidelines.xhtml

growth curve analyses of anxiety and moderated mediation effects of anxiety between mindfulness and negative affect. *Current Psychology* 2022:1-13.

102. Viravan N, Atsariyasing W, Srifuengfung M, et al. Efficacy of Online Mindfulness Program'Mindful Senses' for Depression and Anxiety Reduction in Community Samples: A Randomized Controlled Trial. *Journal of the Medical Association of Thailand* 2022;105(11)

103. Cuijpers P, Miguel C, Harrer M, et al. Cognitive behavior therapy vs. control conditions, other psychotherapies, pharmacotherapies and combined treatment for depression: a comprehensive meta‐analysis including 409 trials with 52,702 patients. *World Psychiatry* 2023;22(1):105-15.

104. Ewing DL, Monsen JJ, Thompson EJ, et al. A meta-analysis of transdiagnostic cognitive behavioural therapy in the treatment of child and young person anxiety disorders. *Behavioural and cognitive psychotherapy* 2015;43(5):562-77.

105. Munasinghe S, Page A, Mannan H, et al. Referral patterns to primary mental health services in Western Sydney (Australia): an analysis of routinely collected data (2005-2018). *Int J Ment Health Syst* 2020;14:37. doi: 10.1186/s13033-020-00368-5 [published Online First: 20200526]

106. Prina AM, Marioni RE, Hammond GC, et al. Improving access to psychological therapies and older people: findings from the Eastern Region. *Behaviour research and therapy* 2014;56(100):75-81. doi: 10.1016/j.brat.2014.03.008 [published Online First: 20140328]

107. Wittchen H-U, Mühlig S, Beesdo K. Mental disorders in primary care. *Dialogues in Clinical Neuroscience* 2003;5(2):115-28. doi: 10.31887/DCNS.2003.5.2/huwittchen

For peer review only - http://bmjopen.bmj.com/site/about/guidelines.xhtml

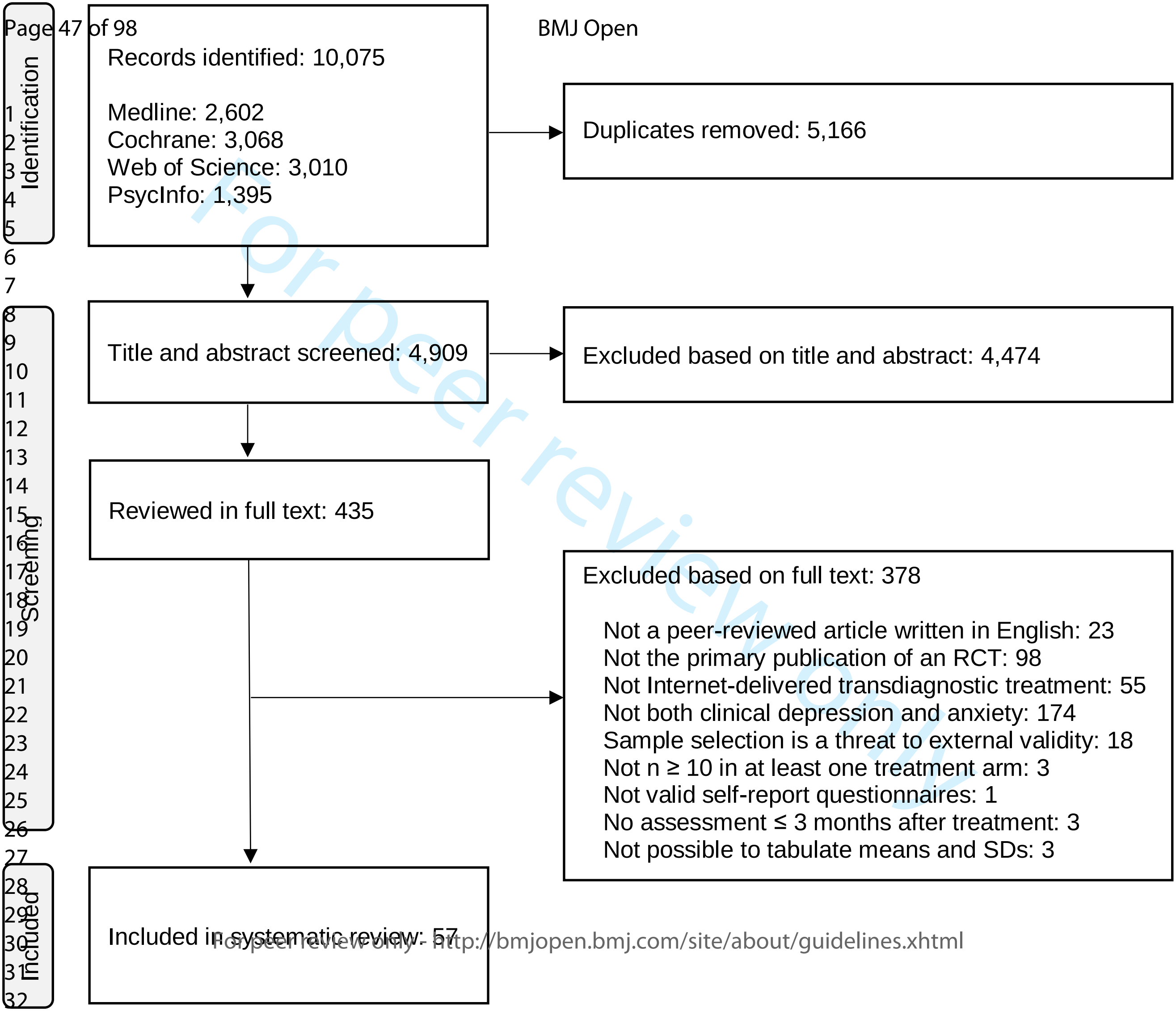

For peer review only - http://bmjopen.bmj.com/site/about/guidelines.xhtml

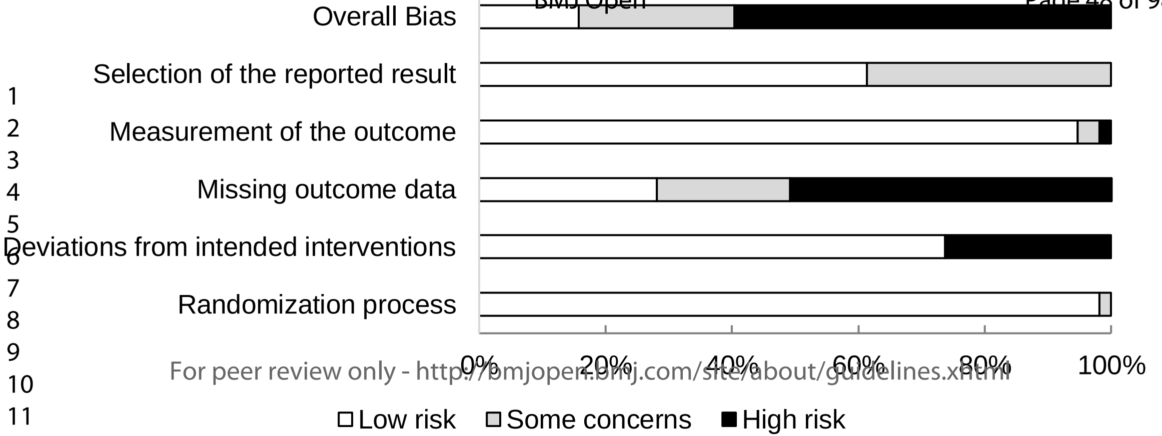
Overall Bias
Selection of the reported result
Measurement of the outcome
Missing outcome data
Deviations from intended interventions
Randomization process
0%
20%
40%
60%
80%
100%
Low risk
Some concerns
High risk

Standardized effect on depression symptoms

Study	Format	Type	g [95% CI]
Al−Refaee (2021)	Mobile app	CBT and mindfulness	0.30 [−0.03, 0.62]
Carolan (2017) I	Web, mainly text	CBT variant	0.19 [−0.35, 0.72]
Carolan (2017) II	Web, mainly text	CBT variant	0.27 [−0.25, 0.80]
Diaz−Garcia (2021) I	Mixed or other	CBT variant	1.21 [0.80, 1.61]
Diaz−Garcia (2021) II	Mixed or other	CBT variant	1.04 [0.63, 1.46]
Farrer (2019)	Mixed or other	CBT and mindfulness	0.15 [−0.12, 0.43]
Gonzalez−Robles (2020)	Web, mainly text	CBT variant	0.37 [0.09, 0.65]
Graham (2020)	Mobile app	CBT variant	0.71 [0.37, 1.04]
Grégoire (2022)	Video format	CBT variant	0.53 [0.14, 0.91]
Hensel (2019)	Web, mainly text	CBT variant	0.41 [0.22, 0.60]
Hosseinzadeh (2021)	Video format	Mindfulness	0.30 [−0.26, 0.87]
Kang (2022)	Mixed or other	Mindfulness	1.56 [1.11, 2.01]
Kladnitski (2020) I	Web, mainly text	CBT variant	0.89 [0.43, 1.36]
Kladnitski (2020) II	Web, mainly text	CBT and mindfulness	1.16 [0.68, 1.64]
Kladnitski (2020) III	Web, mainly text	Mindfulness	0.82 [0.36, 1.28]
Kleiboer (2015) I	Web, mainly text	Problem−solving therapy	0.26 [−0.01, 0.53]
Kleiboer (2015) II	Web, mainly text	Problem−solving therapy	0.29 [0.02, 0.56]
Kleiboer (2015) III	Web, mainly text	Problem−solving therapy	0.40 [0.13, 0.68]
Krafft (2019) I	Mobile app	CBT variant	0.57 [−0.44, 1.58]
Krafft (2019) II	Mobile app	CBT variant	0.51 [−0.49, 1.51]
Levin (2020)	Mobile app	Mindfulness	0.41 [−0.62, 1.43]
Lindegaard (2020)	Web, mainly text	CBT variant	0.29 [−0.22, 0.81]
McCloud (2020)	Mobile app	CBT variant	0.20 [−0.20, 0.60]
Mullin (2015)	Web, mainly text	CBT variant	0.39 [−0.16, 0.94]
Newby (2013)	Web, mainly text	CBT variant	0.99 [0.59, 1.39]
Powell (2013)	Web, mainly text	CBT variant	0.21 [0.14, 0.28]
Proudfoot (2013)	Web, mainly text	Hybrid or other	0.51 [0.32, 0.69]
Robichaud (2020)	Web, mainly text	CBT variant	0.35 [−0.14, 0.84]
Rollman (2018) I	Web, mainly text	CBT variant	0.15 [−0.08, 0.37]
Rollman (2018) II	Web, mainly text	CBT variant	0.15 [−0.07, 0.38]
Schaeuffele (2022)	Web, mainly text	CBT variant	0.82 [0.47, 1.18]
Sethi (2013)	Web, mainly text	CBT variant	1.93 [1.23, 2.63]
Southwell (2017)	Web, mainly text	Gratitude diary	0.37 [−0.01, 0.75]
Terides (2018)	Web, mainly text	CBT variant	0.38 [0.04, 0.71]
Titov (2011)	Web, mainly text	CBT variant	0.57 [0.10, 1.03]
Titov (2013) I	Web, mainly text	CBT variant	0.67 [0.39, 0.96]
Titov (2013) II	Web, mainly text	CBT variant	0.54 [0.27, 0.81]
Twomey (2014)	Web, mainly text	CBT variant	0.19 [−0.30, 0.68]
Tönnies (2021)	Video format	Hybrid or other	0.19 [−0.39, 0.78]
Tullbure (2018)	Web, mainly text	CBT variant	0.96 [0.53, 1.38]
van Staten (2008)	Web, mainly text	Problem−solving therapy	0.50 [0.22, 0.77]
Viskovich (2019)	Mixed or other	CBT variant	0.36 [0.25, 0.48]
Zetterqvist (2003)	Web, mainly text	CBT variant	0.61 [0.08, 1.13]
Pooled total			0.52 [0.42, 0.63]

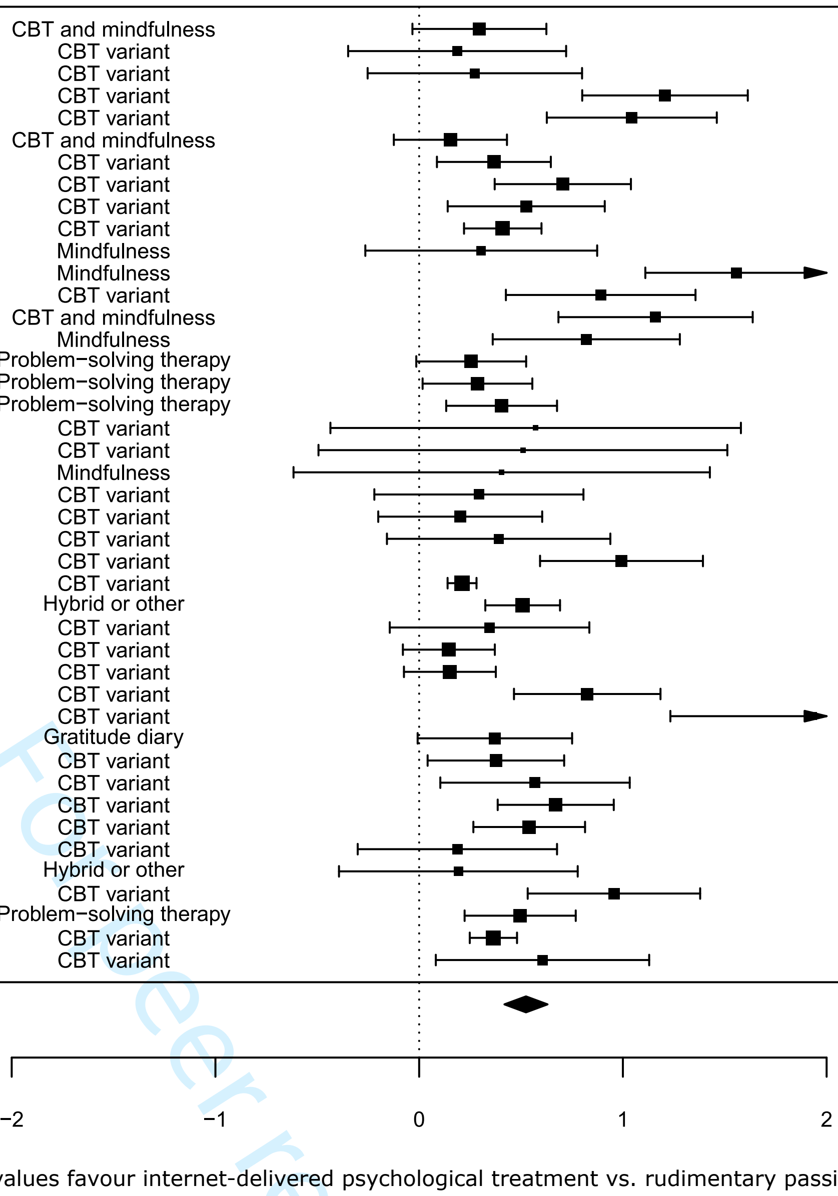

Standardized effect on anxiety

Study	Format	Type	g [95% CI]
Al−Refaee (2021)	Mobile app	CBT and mindfulness	0.19 [−0.13, 0.52]
Carolan (2017) I	Web, mainly text	CBT variant	0.30 [−0.24, 0.84]
Carolan (2017) II	Web, mainly text	CBT variant	0.44 [−0.09, 0.97]
Diaz−Garcia (2021) I	Mixed or other	CBT variant	0.64 [0.24, 1.04]
Diaz−Garcia (2021) II	Mixed or other	CBT variant	0.68 [0.28, 1.08]
Farrer (2019)	Mixed or other	CBT and mindfulness	0.13 [−0.14, 0.41]
Gonzalez−Robles (2020)	Web, mainly text	CBT variant	0.35 [0.07, 0.63]
Graham (2020)	Mobile app	CBT variant	0.60 [0.27, 0.93]
Grégoire (2022)	Video format	CBT variant	0.51 [0.13, 0.90]
Hensel (2019)	Web, mainly text	CBT variant	0.42 [0.23, 0.61]
Hosseinzadeh (2021)	Video format	Mindfulness	0.28 [−0.29, 0.85]
Kang (2022)	Mixed or other	Mindfulness	1.41 [0.97, 1.84]
Kladnitski (2020) I	Web, mainly text	CBT variant	1.06 [0.59, 1.54]
Kladnitski (2020) II	Web, mainly text	CBT and mindfulness	1.22 [0.74, 1.70]
Kladnitski (2020) III	Web, mainly text	Mindfulness	0.99 [0.52, 1.46]
Kleiboer (2015) I	Web, mainly text	Problem−solving therapy	0.30 [0.03, 0.57]
Kleiboer (2015) II	Web, mainly text	Problem−solving therapy	0.27 [0.00, 0.54]
Kleiboer (2015) III	Web, mainly text	Problem−solving therapy	0.33 [0.06, 0.60]
Krafft (2019) I	Mobile app	CBT variant	0.96 [−0.08, 2.00]
Krafft (2019) II	Mobile app	CBT variant	0.49 [−0.51, 1.49]
Levin (2020)	Mobile app	Mindfulness	0.56 [−0.47, 1.59]
Lindegaard (2020)	Web, mainly text	CBT variant	−0.16 [−0.67, 0.35]
McCloud (2020)	Mobile app	CBT variant	0.20 [−0.20, 0.60]
Mullin (2015)	Web, mainly text	CBT variant	0.59 [0.04, 1.15]
Newby (2013)	Web, mainly text	CBT variant	0.96 [0.56, 1.36]
Powell (2013)	Web, mainly text	CBT variant	0.19 [0.12, 0.27]
Proudfoot (2013)	Web, mainly text	Hybrid or other	0.27 [0.09, 0.45]
Robichaud (2020)	Web, mainly text	CBT variant	0.35 [−0.14, 0.84]
Rollman (2018) I	Web, mainly text	CBT variant	0.19 [−0.04, 0.41]
Rollman (2018) II	Web, mainly text	CBT variant	0.20 [−0.03, 0.42]
Schaeuffele (2022)	Web, mainly text	CBT variant	0.75 [0.39, 1.10]
Sethi (2013)	Web, mainly text	CBT variant	1.96 [1.26, 2.66]
Southwell (2017)	Web, mainly text	Gratitude diary	0.39 [0.01, 0.77]
Terides (2018)	Web, mainly text	CBT variant	0.40 [0.06, 0.73]
Titov (2011)	Web, mainly text	CBT variant	−0.51 [−0.97, −0.05]
Titov (2013) I	Web, mainly text	CBT variant	0.56 [0.28, 0.85]
Titov (2013) II	Web, mainly text	CBT variant	0.39 [0.11, 0.66]
Twomey (2014)	Web, mainly text	CBT variant	0.01 [−0.48, 0.50]
Tönnies (2021)	Video format	Hybrid or other	0.13 [−0.46, 0.71]
Tullbure (2018)	Web, mainly text	CBT variant	0.93 [0.51, 1.35]
van Staten (2008)	Web, mainly text	Problem−solving therapy	0.33 [0.06, 0.60]
Viskovich (2019)	Mixed or other	CBT variant	0.02 [−0.10, 0.13]
Zetterqvist (2003)	Web, mainly text	CBT variant	0.56 [0.11, 1.00]
Pooled total			0.45 [0.34, 0.56]

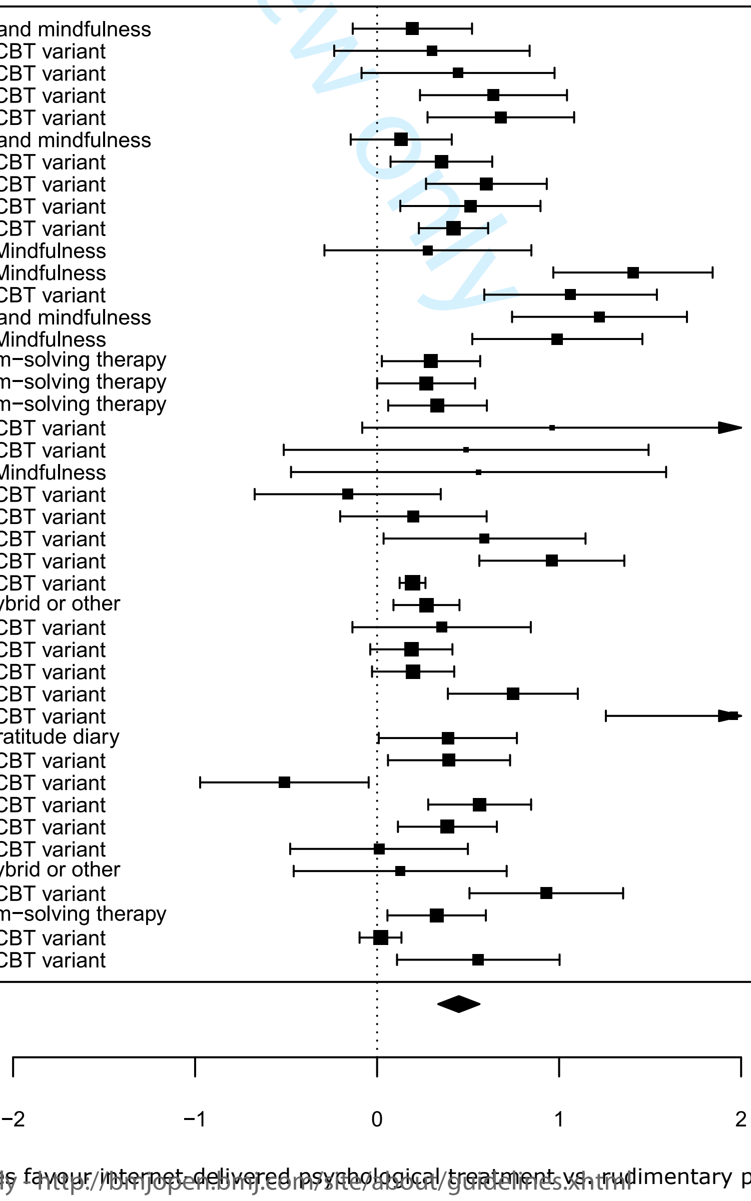

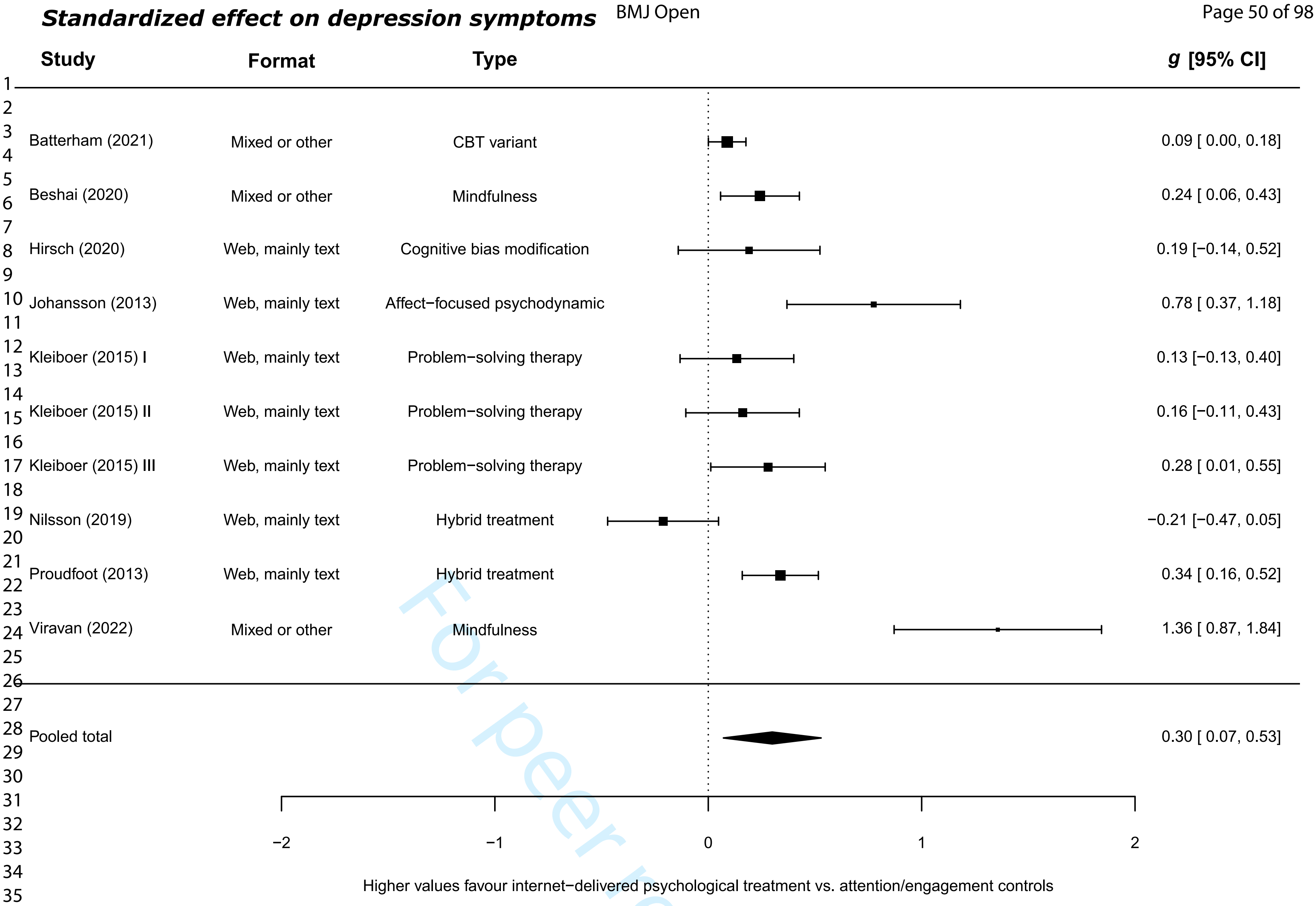

For peer review only

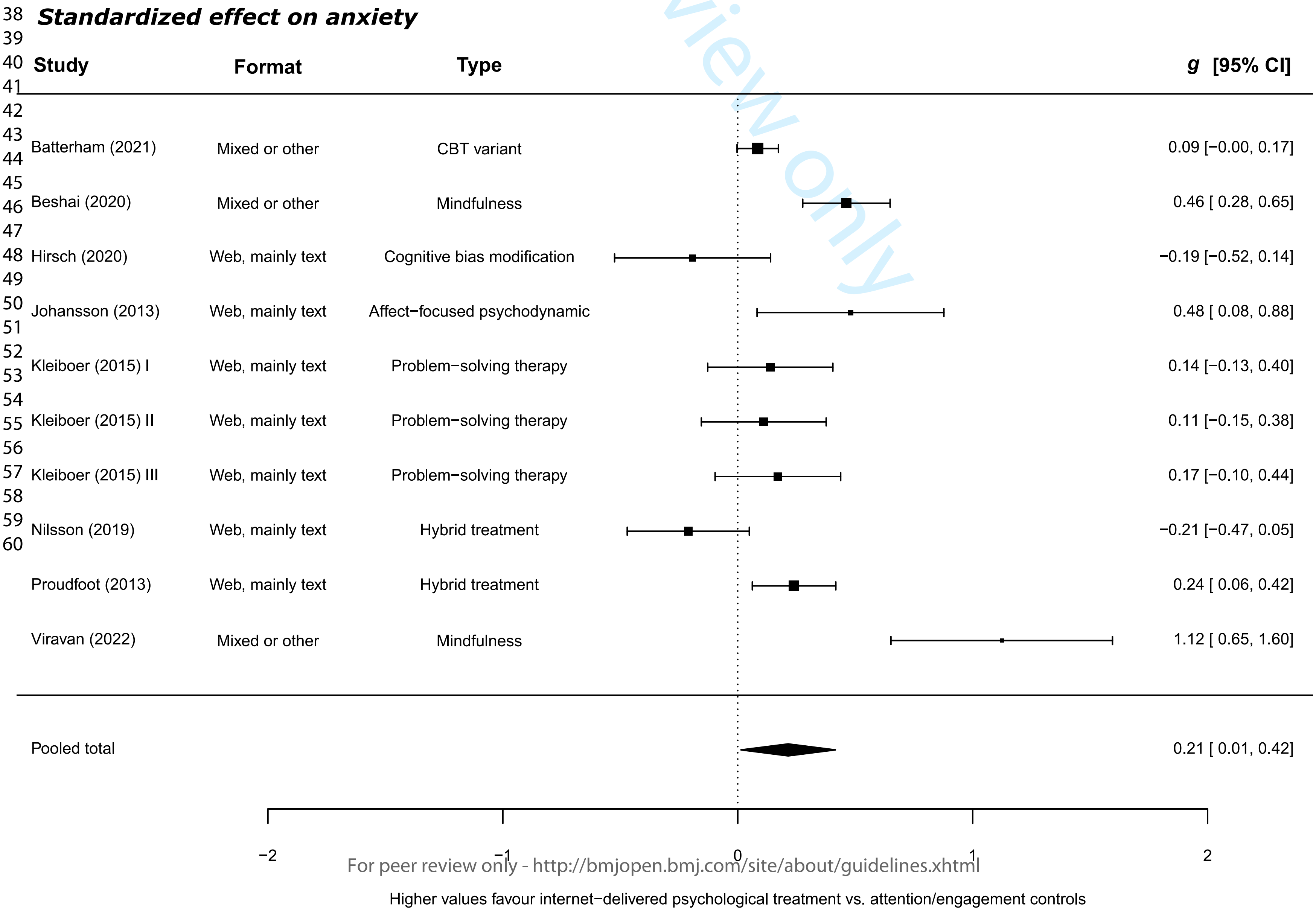

Internet-delivered transdiagnostic psychological treatments for individuals with depression, anxiety, or both

Systematic search: 2021 main search

For peer review only

Karolinska Institutet

Documentation of search strategies
University Library search consultation group

Date: May 24, 2021

Topic/research question: Systematic review and meta-analysis of transdiagnostic psychological interventions delivered via the internet for individuals suffering from common mental disorders (PROSPERO: CRD42021243172)

Name of researcher(s): Erland Axelsson

Librarian(s): Sabina Gillsund & Emma-Lotta Säätelä

Databases:

1. Medline (Ovid)
2. Cochrane Library (Wiley)
3. Web of Science Core Collection (Clarivate Analytics)
4. PsycInfo (Ovid)

Total number of hits:

- Before deduplication: 7,366
- After deduplication: 3,519

Comments:

For peer review only - http://bmjopen.bmj.com/site/about/guidelines.xhtml

Karolinska Institutet

1. Medline

Interface: Ovid MEDLINE(R) and Epub Ahead of Print, In-Process & Other Non-Indexed Citations and Daily

Date of Search: 24 May 2021

Number of hits: 1,898

Comment: In Ovid, two or more words are automatically searched as phrases; i.e. no quotation marks are needed

Field labels

- exp/ = exploded MeSH term
- / = non exploded MeSH term
- .ti,ab,kf. = title, abstract and author keywords
- adjx = within x words, regardless of order
- * = truncation of word for alternate endings

#	Searches	Results
1	exp Psychotherapy/	202021
2	(("acceptance and commitment" or affect-focused or behavio?r* or biofeedback or computeri* or cognit* or digital or "emotional awareness and expression" or educat* or exposure or guided or internet* or interpersonal or mobile or online or positive psychology or psychodynamic or psychologic* or self-guided or self-help or transdiagnost* or web*) adj2 (intervention* or therap* or treat* or program*)).ti,ab,kf.	188596
3	(behavio?ral activation or bias modification or cognitive restructuring or hypno* or meditation or mindfulness or problem solving or psychoeducation* or psychotherap* or relaxation or self-management or unified protocol).ti,ab,kf.	251506
4	or/1-3	538226
5	Anxiety Disorders/	35452
6	Agoraphobia/	2617
7	Neurotic Disorders/	17987
8	Obsessive-Compulsive Disorder/	14943
9	Panic Disorder/	7019
10	exp Phobic Disorders/	11590
11	Stress Disorders, Post-Traumatic/	34732
12	Anxiety/	87458
13	(agoraphobi* or anxi* or obsessive-compulsive or panic or phobi* or post-traumatic stress or posttraumatic stress).ti,ab,kf.	281431
14	or/5-13	336088

Karolinska Institutet

15	Mood Disorders/	14821
16	Depressive Disorder/	73869
17	Depressive Disorder, Major/	32106
18	Affective Symptoms/	13300
19	Depression/	127756
20	depress*.ti,ab,kf.	491024
21	((affective or mood) adj2 disorder*).ti,ab,kf.	37052
22	or/15-21	562251
23	14 and 22	140055
24	(emotional adj2 disorder*).ti,ab,kf.	3677
25	(transdiagnost* or unified protocol).ti,ab,kf.	2780
26	24 or 25	6275
27	23 or 26	144265
28	Internet/	75778
29	Internet-Based Intervention/	544
30	Therapy, Computer-Assisted/	6898
31	Telemedicine/	28197
32	Telerehabilitation/	548
33	exp Remote Consultation/	5247
34	Mobile Applications/	7799
35	Videoconferencing/	1919
36	(app or apps or cellphone* or computer* or digital or e mail or email or internet* or mobile application* or online or phone* or smartphone* or technolog* or telephone* or text message* or video* or virtual or web based or web site or website).ti,ab,kf.	1396114
37	(distance counsel?ing or distance consultation* or e consultation* or econsultation* or e counsel?ing or ecounsel?ing or e health* or ehealth* or e therapies or e therapy or etherap* or e visit* or evisit* or m health or mhealth or mobile counsel?ing or mobile consultation* or remote consultation* or remote counsel?ing or tele health* or telehealth* or tele consultation* or teleconsultation* or tele medicine or telemedicine or tele rehabilitation or telerehabilitation).ti,ab,kf.	36032
38	or/28-37	1441976
39	randomized controlled trial.pt.	531357
40	controlled clinical trial.pt.	94168
41	randomi?ed.ab.	622540

For peer review only - http://bmjopen.bmj.com/site/about/guidelines.xhtml

Karolinska Institutet

42	placebo.ab.	218143
43	clinical trials as topic.sh.	195916
44	randomly.ab.	357785
45	trial.ti.	240304
46	or/39-45	1407342
47	(systematic review or meta-analy*).ti.	219916
48	46 not 47	1340792
49	exp animals/ not humans.sh.	4832148
50	48 not 49	1230987
51	4 and 27 and 38 and 50	1921
52	limit 51 to english language	1905
53	limit 52 to yr="1995 -Current"	1898

For peer review only

For peer review only - http://bmjopen.bmj.com/site/about/guidelines.xhtml

Karolinska Institutet

2. Cochrane Library

Interface: Wiley<br><br>Date of Search: 24 May 2021<br><br>Number of hits: 2,337	Field labels<br><br>• ti,ab,kw = title, abstract and author keywords<br>• NEAR/x = within x words, regardless of order<br>• * = truncation of word for alternate endings

ID	Search	Hits
#1	MeSH descriptor: [Psychotherapy] explode all trees	24621
#2	(("acceptance and commitment" or "affect-focused" or behavio?r* or "biofeedback" or computeri* or cognit* or "digital" or "emotional awareness and expression" or educat* or "exposure" or "guided" or internet* or "interpersonal" or "mobile" or "online" or "positive psychology" or "psychodynamic" or psychologic* or "self-guided" or "self-help" or transdiagnost* or web*) NEAR/2 (intervention* or therap* or treat* or program*)):ti,ab,kw	81443
#3	((behavio?ral NEXT activation) or "bias modification" or "cognitive restructuring" or hypno* or "meditation" or "mindfulness" or "problem solving" or psychoeducation* or psychotherap* or "relaxation" or "self-management" or "unified protocol"):ti,ab,kw	54905
#4	#1 or #2 or #3	121709
#5	MeSH descriptor: [Anxiety Disorders] this term only	3927
#6	MeSH descriptor: [Agoraphobia] this term only	436
#7	MeSH descriptor: [Neurotic Disorders] this term only	300
#8	MeSH descriptor: [Obsessive-Compulsive Disorder] this term only	1050
#9	MeSH descriptor: [Panic Disorder] this term only	955
#10	MeSH descriptor: [Phobic Disorders] explode all trees	1382
#11	MeSH descriptor: [Stress Disorders, Post-Traumatic] this term only	2707
#12	MeSH descriptor: [Anxiety] this term only	7681
#13	(agoraphobi* or anxi* or "obsessive compulsive" or "obsessive-compulsive" or "panic" or phobi* or "post-traumatic stress" or "posttraumatic stress"):ti,ab,kw	63654
#14	#5 or #6 or #7 or #8 or #9 or #10 or #11 or #12 or #13	64273
#15	MeSH descriptor: [Mood Disorders] this term only	839
#16	MeSH descriptor: [Depressive Disorder] this term only	8011
#17	MeSH descriptor: [Depressive Disorder, Major] this term only	5111
#18	MeSH descriptor: [Affective Symptoms] this term only	459
#19	MeSH descriptor: [Depression] this term only	12730
#20	depress*:ti,ab,kw	89514
#21	(("affective" or "mood") NEAR/3 disorder*):ti,ab,kw	5404
#22	#15 or #16 or #17 or #18 or #19 or #20 or #21	91976
#23	#14 and #22	30987
#24	("emotional" NEAR/2 disorder*):ti,ab,kw	862
#25	(transdiagnost* or "unified protocol"):ti,ab,kw	730
#26	#24 or #25	1468
#27	#23 or #26	31780
#28	MeSH descriptor: [Internet] this term only	3893
#29	MeSH descriptor: [Internet-Based Intervention] this term only	182

For peer review only

#30	MeSH descriptor: [Therapy, Computer-Assisted] this term only	1342
#31	MeSH descriptor: [Telemedicine] this term only	2293
#32	MeSH descriptor: [Telerehabilitation] this term only	126
#33	MeSH descriptor: [Remote Consultation] explode all trees	398
#34	MeSH descriptor: [Mobile Applications] this term only	765
#35	MeSH descriptor: [Videoconferencing] this term only	190
#36	("app" or "apps" or cellphone* or computer* or "digital" or "e mail" or "email" or internet* or ("mobile" NEXT application*) or "online" or phone* or smartphone* or technolog* or telephone* or ("text" NEXT message*) or video* or "virtual" or "web based" or "web site" or "website"):ti,ab,kw	150142
#37	(("distance" NEXT "counsel?ing") or ("distance" NEXT consultation*) or ("e" NEXT consultation*) or econsultation* or ("e" NEXT "counsel?ing") or "ecounsel?ing" or ("e" NEXT health*) or ehealth* or "e therapies" or "e therapy" or etherap* or ("e" NEXT visit*) or evisit* or "m health" or "mhealth" or ("mobile" NEXT "counsel?ing") or ("mobile" NEXT consultation*) or ("remote" NEXT consultation*) or ("remote" NEXT "counsel?ing") or ("tele" NEXT health*) or telehealth* or ("tele" NEXT consultation*) or teleconsultation* or "tele medicine" or "telemedicine" or "tele rehabilitation" or "telerehabilitation"):ti,ab,kw	9310
#38	#28 or #29 or #30 or #31 or #32 or #33 or #34 or #35 or #36 or #37	152807
#39	#4 and #27 and #38	4365
#40	(clinicaltrials or trialsearch):so	364015
#41	#39 NOT #40	2416
	Limit to publication date 1995-2021 in Trials	2337

For peer review only - http://bmjopen.bmj.com/site/about/guidelines.xhtml

Karolinska Institutet

3. Web of Science Core Collection

Interface: Clarivate Analytics<br><br>Date of Search: 24 May 2021<br><br>Number of hits: 2,233	Field labels<br><br>• TS/Topic = title, abstract, author keywords and Keywords Plus<br>• NEAR/x = within x words, regardless of order<br>• * = truncation of word for alternate endings<br><br>Note: sometimes “quotation marks” are needed for single search terms to avoid automatic term mapping (lemmatization).

18 2,233

#14 AND #11 AND #8 AND #3

Refined by: LANGUAGES: (ENGLISH) AND [excluding] PUBLICATION YEARS: (1994 OR 1993) AND DOCUMENT TYPES: (ARTICLE OR CORRECTION OR EARLY ACCESS)

17 2,366

#14 AND #11 AND #8 AND #3

Refined by: LANGUAGES: (ENGLISH) AND [excluding] PUBLICATION YEARS: (1994 OR 1993)

16 2,370

#14 AND #11 AND #8 AND #3

Refined by: LANGUAGES: (ENGLISH)

15 2,395

#14 AND #11 AND #8 AND #3

14 916,173

#12 NOT #13

13 259,971

TI=(“systematic review” or “meta-analy*”)

12 982,134

TI=(“randomi$ed” OR “randomi$ed” OR “randomi$ation” OR “randomi$ation” OR placebo* OR (random* AND (allocat* OR assign*)) OR (blind* AND (“single” OR “double” OR “treble” OR “triple”))) OR AB=(“randomi$ed” OR “randomi$ed” OR “randomi$ation” OR “randomi$ation” OR placebo* OR (random* AND (allocat* OR assign*)) OR (blind* AND (“single” OR “double” OR “treble” OR “triple”)))

For peer review only - http://bmjopen.bmj.com/site/about/guidelines.xhtml

Karolinska Institutet

11 4,745,692

#10 OR #9

10 51,707

TS=(“distance counsel$ing” or “distance consultation*” or “e consultation*” or econsultation* or “e counsel$ing” or “ecounsel$ing” or “e health*” or ehealth* or “e therapies” or “e therapy” or etherap* or “e visit*” or evisit* or “m health” or “mhealth” or “mobile counsel$ing” or “mobile consultation*” or “remote consultation*” or “remote counsel$ing” or “tele health*” or telehealth* or “tele consultation*” or teleconsultation* or “tele medicine” or “telemedicine” or “tele rehabilitation” or “telerehabilitation")

9 4,726,717

TS=(“app” or “apps” or cellphone* or computer* or “digital” or “e mail” or “email” or internet* or “mobile application*” or “online” or phone* or smartphone* or technolog* or telephone* or “text message*” or video* or “virtual” or “web based” or “web site” or “website”)

8 176,232

#7 OR #6

7 7,752

TS=((”emotional” NEAR/1 disorder*) or transdiagnost* or “unified protocol”)

6 171,279

#5 AND #4

5 691,430

TS=(depress* or ((“affective” or “mood”) NEAR/1 disorder*))

4 401,339

TS=(agoraphobi* or anxi* or “obsessive-compulsive” or “panic” or phobi* or “post-traumatic stress” or “posttraumatic stress”)

3 871,462

#2 OR #1

2 644,089

For peer review only - http://bmjopen.bmj.com/site/about/guidelines.xhtml

Karolinska Institutet

TS=((“behavio$ral activation” or “bias modification” or “cognitive restructuring” or hypno* or “meditation” or “mindfulness” or “problem solving” or psychoeducation* or psychotherap* or “relaxation” or “self-management” or “unified protocol”))

1 263,093

TS=((("acceptance and commitment" or “affect-focused” or behavio$r* or “biofeedback” or computeri* or cognit* or “digital” or "emotional awareness and expression" or educat* or “exposure” or “guided” or internet* or “interpersonal” or “mobile” or “online” or “positive psychology” or “psychodynamic” or psychologic* or “self-guided” or “self-help” or transdiagnost* or web*) NEAR/1 (intervention* or therap* or treat* or program*)))

For peer review only

For peer review only - http://bmjopen.bmj.com/site/about/guidelines.xhtml

Karolinska Institutet

4. Psycinfo

Interface: Ovid<br><br>Date of Search: 24 May 2021<br><br>Number of hits: 898<br><br>Comment: In Ovid, two or more words are automatically searched as phrases; i.e. no quotation marks are needed	Field labels<br><br>• exp/ = exploded controlled term<br>• / = non exploded controlled term<br>• .ti,ab,id. = title, abstract and author keywords<br>• adjx = within x words, regardless of order<br>• * = truncation of word for alternate endings

#	Searches	Results
1	exp Psychotherapy/	207730
2	exp Cognitive Therapy/	13639
3	exp Behavior Therapy/	21289
4	exp Cognitive Behavior Therapy/	23144
5	(("acceptance and commitment" or affect-focused or behavio?r* or biofeedback or computeri* or cognit* or digital or "emotional awareness and expression" or educat* or exposure or guided or internet* or interpersonal or mobile or online or positive psychology or psychodynamic or psychologic* or self-guided or self-help or transdiagnost* or web*) adj2 (intervention* or therap* or treat* or program*)).ti,ab,id.	175860
6	(behavio?ral activation or bias modification or cognitive restructuring or hypno* or meditation or mindfulness or problem solving or psychoeducation* or psychotherap* or relaxation or self-management or unified protocol).ti,ab,id.	240370
7	or/1-6	495594
8	Anxiety Disorders/	19140
9	exp Agoraphobia/	2916
10	Neurosis/	7043
11	Obsessive Compulsive Disorder/	14683
12	Panic Disorder/	7760
13	exp Phobias/	13434
14	Posttraumatic Stress Disorder/	34688
15	Anxiety/	65640
16	(agoraphobi* or anxi* or obsessive-compulsive or panic or phobi* or post-traumatic stress or posttraumatic stress).ti,ab,id.	283726

For peer review only - http://bmjopen.bmj.com/site/about/guidelines.xhtml

Karolinska Institutet

17	or/8-16	296637
18	Affective Disorders/	14669
19	exp Major Depression/	138828
20	"Depression (Emotion)"/	26043
21	depress*.ti,ab,id.	321600
22	((affective or mood) adj2 disorder*).ti,ab,id.	35856
23	or/18-22	343842
24	17 and 23	109245
25	(emotional adj2 disorder*).ti,ab,id.	5905
26	(transdiagnost* or unified protocol).ti,ab,id.	3036
27	25 or 26	8661
28	24 or 27	115823
29	Internet/	29614
30	Digital Interventions/	593
31	Computer Assisted Therapy/	1140
32	exp Telemedicine/	9675
33	Digital Technology/	719
34	Videoconferencing/	645
35	Mobile Applications/	1193
36	(app or apps or cellphone* or computer* or digital or e mail or email or internet* or mobile application* or online or phone* or smartphone* or technolog* or telephone* or text message* or video* or virtual or web based or web site or website).ti,ab,id.	436545
37	(distance counsel?ing or distance consultation* or e consultation* or econsultation* or e counsel?ing or ecounsel?ing or e health* or ehealth* or e therapies or e therapy or etherap* or e visit* or evisit* or m health or mhealth or mobile counsel?ing or mobile consultation* or remote consultation* or remote counsel?ing or tele health* or telehealth* or tele consultation* or teleconsultation* or tele medicine or telemedicine or tele rehabilitation or telerehabilitation).ti,ab,id.	7477
38	or/29-37	440321
39	exp Randomized Controlled Trials/	954
40	randomi?ed.ab.	83970
41	placebo.ab.	40594
42	randomly.ab.	76127
43	trial.ti.	32809

Karolinska Institutet

44	or/39-43	183930
45	(systematic review or meta-analy*).ti.	40744
46	44 not 45	176278
47	7 and 28 and 38 and 46	1037
48	limit 47 to english language	983
49	limit 48 to yr="1995 -Current"	981
50	limit 49 to "0100 journal"	898

For peer review only

For peer review only - http://bmjopen.bmj.com/site/about/guidelines.xhtml

Systematic search: 2023 updated main search

For peer review only

Documentation of search strategies
University Library search consultation group

Date: February 2023

Topic/research question: Systematic review and meta-analysis of transdiagnostic psychological interventions delivered via the internet for individuals suffering from common mental disorders (PROSPERO: CRD42021243172)

Name of researcher(s): Erland Axelsson

Librarian(s): Sabina Gillsund & Emma-Lotta Säätelä

Databases:

1. Medline (Ovid)
2. Cochrane Library (Wiley)
3. Web of Science Core Collection (Clarivate Analytics)
4. PsycInfo (EBSCO)

Total number of hits:

- Before deduplication: 10,075
- After deduplication: 4,909

1. Medline

Interface: Ovid MEDLINE(R) ALL<br><br>Date of Search: 6 February 2023<br><br>Number of hits: 2,602<br><br>Comment: In Ovid, two or more words are automatically searched as phrases; i.e. no quotation marks are needed	Field labels<br><br>• exp/ = exploded MeSH term<br>• / = non exploded MeSH term<br>• .ti,ab,kf. = title, abstract and author keywords<br>• adjx = within x words, regardless of order<br>• * = truncation of word for alternate endings

For peer review only - http://bmjopen.bmj.com/site/about/guidelines.xhtml

Karolinska Institutet

Database(s): **Ovid MEDLINE(R) ALL** 1946 to February 03, 2023
Search Strategy:

#	Searches	Results
1	exp Psychotherapy/	215915
2	(("acceptance and commitment" or affect-focused or behavio?r* or biofeedback or computeri* or cognit* or digital or "emotional awareness and expression" or educat* or exposure or guided or internet* or interpersonal or mobile or online or positive psychology or psychodynamic or psychologic* or self-guided or self-help or transdiagnost* or web*) adj2 (intervention* or therap* or treat* or program*)).ti,ab,kf.	220048
3	(behavio?ral activation or bias modification or cognitive restructuring or hypno* or meditation or mindfulness or problem solving or psychoeducation* or psychotherap* or relaxation or self-management or unified protocol).ti,ab,kf.	277967
4	or/1-3	597258
5	Anxiety Disorders/	40399
6	Agoraphobia/	2669
7	Neurotic Disorders/	18000
8	Obsessive-Compulsive Disorder/	16224
9	Panic Disorder/	7250
10	exp Phobic Disorders/	12293
11	Stress Disorders, Post-Traumatic/	40198
12	Anxiety/	103449
13	(agoraphobi* or anxi* or obsessive-compulsive or panic or phobi* or post-traumatic stress or posttraumatic stress).ti,ab,kf.	332361
14	or/5-13	389293
15	Mood Disorders/	15751
16	Depressive Disorder/	74889
17	Depressive Disorder, Major/	37299
18	Affective Symptoms/	13816
19	Depression/	146908
20	depress*.ti,ab,kf.	552903
21	((affective or mood) adj2 disorder*).ti,ab,kf.	40988
22	or/15-21	627024
23	14 and 22	168605

For peer review only - http://bmjopen.bmj.com/site/about/guidelines.xhtml

Karolinska Institutet

24	(emotional adj2 disorder*).ti,ab,kf.	4243
25	(transdiagnost* or unified protocol).ti,ab,kf.	4104
26	24 or 25	8093
27	23 or 26	174000
28	Internet/	80600
29	Internet-Based Intervention/	1071
30	Therapy, Computer-Assisted/	6968
31	Telemedicine/	36073
32	Telerehabilitation/	900
33	exp Remote Consultation/	5715
34	Mobile Applications/	10971
35	Videoconferencing/	2295
36	(app or apps or cellphone* or computer* or digital or e mail or email or internet* or mobile application* or online or phone* or smartphone* or technolog* or telephone* or text message* or video* or virtual or web based or web site or website).ti,ab,kf.	1678151
37	(distance counsel?ing or distance consultation* or e consultation* or econsultation* or e counsel?ing or ecounsel?ing or e health* or ehealth* or e therapies or e therapy or etherap* or e visit* or evisit* or m health or mhealth or mobile counsel?ing or mobile consultation* or remote consultation* or remote counsel?ing or tele health* or telehealth* or tele consultation* or teleconsultation* or tele medicine or telemedicine or tele rehabilitation or telerehabilitation).ti,ab,kf.	51873
38	or/28-37	1730206
39	randomized controlled trial.pt.	585953
40	controlled clinical trial.pt.	95177
41	randomi?ed.ab.	708985
42	placebo.ab.	235960
43	clinical trials as topic.sh.	200855
44	randomly.ab.	402423
45	trial.ti.	279911
46	or/39-45	1550232
47	(systematic review or meta-analy*).ti.	290755
48	46 not 47	1464360
49	exp animals/ not humans.sh.	5089811
50	48 not 49	1343292

51	4 and 27 and 38 and 50	2627
52	limit 51 to english language	2609
53	limit 52 to yr="1995 -Current"	2602

For peer review only

Karolinska Institutet

2. Cochrane Library

Interface: Wiley<br><br>Date of Search: 3 February 2023<br><br>Number of hits: 3,068	Field labels<br><br>• ti,ab,kw = title, abstract and author keywords<br>• NEAR/x = within x words, regardless of order<br>• * = truncation of word for alternate endings

ID	Search	Hits
#1	MeSH descriptor: [Psychotherapy] explode all trees	30769
#2	(("acceptance and commitment" or "affect-focused" or behavio?r* or "biofeedback" or computeri* or cognit* or "digital" or "emotional awareness and expression" or educat* or "exposure" or "guided" or internet* or "interpersonal" or "mobile" or "online" or "positive psychology" or "psychodynamic" or psychologic* or "self-guided" or "self-help" or transdiagnost* or web*) NEAR/2 (intervention* or therap* or treat* or program*)):ti,ab,kw	95851
#3	((behavio?ral NEXT activation) or "bias modification" or "cognitive restructuring" or hypno* or "meditation" or mindfulness or "problem solving" or psychoeducation* or psychotherap* or "relaxation" or "self-management" or "unified protocol"):ti,ab,kw	64668
#4	#1 or #2 or #3	143208
#5	MeSH descriptor: [Anxiety Disorders] this term only	5112
#6	MeSH descriptor: [Agoraphobia] this term only	472
#7	MeSH descriptor: [Neurotic Disorders] this term only	316
#8	MeSH descriptor: [Obsessive-Compulsive Disorder] this term only	1250
#9	MeSH descriptor: [Panic Disorder] this term only	1053
#10	MeSH descriptor: [Phobic Disorders] explode all trees	1942
#11	MeSH descriptor: [Stress Disorders, Post-Traumatic] this term only	3561
#12	MeSH descriptor: [Anxiety] this term only	9845
#13	(agoraphobi* or anxi* or "obsessive compulsive" or "obsessive-compulsive" or "panic" or phobi* or "post-traumatic stress" or "posttraumatic stress"):ti,ab,kw	76993
#14	#5 or #6 or #7 or #8 or #9 or #10 or #11 or #12 or #13	77700
#15	MeSH descriptor: [Mood Disorders] this term only	1004
#16	MeSH descriptor: [Depressive Disorder] this term only	9053
#17	MeSH descriptor: [Depressive Disorder, Major] this term only	6360
#18	MeSH descriptor: [Affective Symptoms] this term only	514
#19	MeSH descriptor: [Depression] this term only	15880
#20	depress*:ti,ab,kw	103690
#21	(("affective" or "mood") NEAR/3 disorder*):ti,ab,kw	6072
#22	#15 or #16 or #17 or #18 or #19 or #20 or #21	106436
#23	#14 and #22	38854
#24	("emotional" NEAR/2 disorder*):ti,ab,kw	1098
#25	(transdiagnost* or "unified protocol"):ti,ab,kw	1061
#26	#24 or #25	1974
#27	#23 or #26	39878

For peer review only - http://bmjopen.bmj.com/site/about/guidelines.xhtml

Karolinska Institutet

#28	MeSH descriptor: [Internet] this term only	4780
#29	MeSH descriptor: [Internet-Based Intervention] this term only	527
#30	MeSH descriptor: [Therapy, Computer-Assisted] this term only	1476
#31	MeSH descriptor: [Telemedicine] this term only	3338
#32	MeSH descriptor: [Telerehabilitation] this term only	229
#33	MeSH descriptor: [Remote Consultation] 3 tree(s) exploded	440
#34	MeSH descriptor: [Mobile Applications] this term only	1476
#35	MeSH descriptor: [Videoconferencing] this term only	259
#36	("app" or "apps" or cellphone* or computer* or "digital" or "e mail" or "email" or internet* or (mobile NEXT application*) or "online" or phone* or smartphone* or technolog* or telephone* or (text NEXT message*) or video* or "virtual" or "web based" or "web site" or "website"):ti,ab,kw	184801
#37	(("distance" NEXT counsel?ing) or ("distance" NEXT consultation*) or ("e" NEXT consultation*) or econsultation* or ("e" NEXT counsel?ing) or ecounsel?ing or ("e" NEXT health*) or ehealth* or "e therapies" or "e therapy" or etherap* or ("e" NEXT visit*) or evisit* or "m health" or "mhealth" or ("mobile" NEXT counsel?ing) or ("mobile" NEXT consultation*) or ("remote" NEXT consultation*) or ("remote" NEXT counsel?ing) or ("tele" NEXT health*) or telehealth* or ("tele" NEXT consultation*) or teleconsultation* or "tele medicine" or "telemedicine" or "tele rehabilitation" or "telerehabilitation"):ti,ab,kw	12716
#38	#28 or #29 or #30 or #31 or #32 or #33 or #34 or #35 or #36 or #37	188389
#39	#4 and #27 and #38	6085
#40	(clinicaltrials or trialsearch):so	444610
#41	#39 NOT #40	3156
	Limit to publication date 1995-2023 in Trials	3068

For peer review only

3. Web of Science Core Collection

Interface: Clarivate Analytics<br><br>Date of Search: 3 February 2023<br><br>Number of hits: 3,010	Field labels<br><br>• TS/Topic = title, abstract, author keywords and Keywords Plus<br>• NEAR/x = within x words, regardless of order<br>• * = truncation of word for alternate endings<br><br>Note: sometimes "quotation marks" are needed for single search terms to avoid automatic term mapping (lemmatization).

18 #14 AND #11 AND #8 AND #3
Refined by: LANGUAGES: (ENGLISH) AND [excluding] PUBLICATION YEARS: (1994 OR 1993) AND DOCUMENT TYPES: (ARTICLE OR CORRECTION OR EARLY ACCESS)

17 #14 AND #11 AND #8 AND #3
Refined by: LANGUAGES: (ENGLISH) AND [excluding] PUBLICATION YEARS: (1994 OR 1993)

16 #14 AND #11 AND #8 AND #3
Refined by: LANGUAGES: (ENGLISH)

15 #14 AND #11 AND #8 AND #3

14 #12 NOT #13

13 TI=("systematic review" or "meta-analy*")

12 TI=("randomi$ed" OR "randomi$ed" OR "randomi$ation" OR "randomi$ation" OR placebo* OR (random* AND (allocat* OR assign*)) OR (blind* AND ("single" OR "double" OR "treble" OR "triple"))) OR AB=("randomi$ed" OR "randomi$ed" OR "randomi$ation" OR "randomi$ation" OR placebo* OR (random* AND (allocat* OR assign*)) OR (blind* AND ("single" OR "double" OR "treble" OR "triple")))

11 #10 OR #9

10 TS=("distance counsel$ing" or "distance consultation*" or "e consultation*" or econsultation* or "e counsel$ing" or "ecounsel$ing" or "e health*" or ehealth* or "e therapies" or "e therapy" or etherap* or "e visit*" or evisit* or "m health" or "mhealth" or "mobile counsel$ing" or "mobile consultation*" or "remote consultation*" or "remote counsel$ing" or "tele health*" or telehealth* or "tele consultation*" or teleconsultation* or "tele medicine" or "telemedicine" or "tele rehabilitation" or "telerehabilitation")

For peer review only - http://bmjopen.bmj.com/site/about/guidelines.xhtml

9 TS=(“app” or “apps” or cellphone* or computer* or “digital” or “e mail” or “email” or internet* or “mobile application*” or “online” or phone* or smartphone* or technolog* or telephone* or “text message*” or video* or “virtual” or “web based” or “web site” or “website”)

8 #7 OR #6

7 TS=((”emotional” NEAR/1 disorder*) or transdiagnost* or “unified protocol”)

6 #5 AND #4

5 TS=(depress* or ((“affective” or “mood”) NEAR/1 disorder*))

4 TS=(agoraphobi* or anxi* or “obsessive-compulsive” or “panic” or phobi* or “post-traumatic stress” or “posttraumatic stress”)

3 #2 OR #1

2 TS=((“behavio$ral activation” or “bias modification” or “cognitive restructuring” or hypno* or “meditation” or “mindfulness” or “problem solving” or psychoeducation* or psychotherap* or “relaxation” or “self-management” or “unified protocol”))

1 TS=((("acceptance and commitment" or “affect-focused” or behavio$r* or “biofeedback” or computeri* or cognit* or “digital” or "emotional awareness and expression" or educat* or “exposure” or “guided” or internet* or “interpersonal” or “mobile” or “online” or “positive psychology” or “psychodynamic” or psychologic* or “self-guided” or “self-help” or transdiagnost* or web*) NEAR/1 (intervention* or therap* or treat* or program*)))

For peer review only - http://bmjopen.bmj.com/site/about/guidelines.xhtml

Karolinska Institutet

4. Psycinfo

Interface: EBSCO

Date of Search: 6 february 2023

Number of hits: 1,395

Field labels

- DE = subject heading
- TI = title
- AB = abstract
- KW = author keywords
- Nx = within x words, regardless of order
- * = truncation of word for alternate endings

S47	S45 NOT S36<br><br>Publication year: 1995-2023<br>Language: - english	1,395
S45	S44 NOT S36	1,418
S44	S7 AND S32 AND S35 AND S43	1,572
S43	S39 OR S42	135,063
S42	S40 AND S41	125,758
S41	S17 OR S18 OR S19 OR S20 OR S21	375,079
S40	S8 OR S9 OR S10 OR S11 OR S12 OR S13 OR S14 OR S15 OR S16	332,986
S39	S37 OR S38	12,089
S38	TI (transdiagnost* or "unified protocol") OR AB (transdiagnost* or "unified protocol") OR KW (transdiagnost* or "unified protocol")	4,261
S37	TI emotional N2 disorder* OR AB emotional N2 disorder* OR KW emotional N2 disorder*	8,225
S36	TI "systematic review" or meta-analy*	50,479
S35	S33 OR S34	206,233
S34	AB randomi?ed OR AB placebo OR AB randomly OR TI trial	206,178
S33	DE "Randomized Controlled Trials" OR DE "Randomized Clinical Trials"	1,387
S32	S23 OR S24 OR S25 OR S26 OR S27 OR S28 OR S29 OR S30 OR S31	504,430
S31	TI (“distance counsel#ing” or “distance consultation*” or “e consultation*” or econsultation* or “e counsel#ing” or “ecounsel#ing” or “e health*” or ehealth* or “e therapies” or “e therapy” or etherap* or “e visit*” or evisit* or “m health” or “mhealth” or “mobile counsel#ing” or “mobile consultation*” or “remote consultation*” or “remote counsel#ing” or “tele health*” or telehealth* or “tele consultation*” or teleconsultation* or “tele medicine” or “telemedicine” or “tele rehabilitation” or “telerehabilitation") OR AB (“distance counsel#ing” or “distance consultation*” or “e consultation*” or econsultation* or “e counsel#ing” or “ecounsel#ing” or “e health*” or ehealth* or “e therapies” or “e therapy” or etherap* or “e visit*” or evisit* or “m health” or “mhealth” or “mobile counsel#ing” or “mobile consultation*” or “remote consultation*” or “remote counsel#ing” or “tele health*” or telehealth* or “tele consultation*” or teleconsultation* or “tele medicine” or “telemedicine” or “tele rehabilitation” or “telerehabilitation") OR KW (“distance counsel#ing” or “distance consultation*” or “e consultation*” or econsultation* or “e counsel#ing” or “ecounsel#ing” or “e health*” or ehealth* or “e therapies” or “e therapy” or etherap* or “e visit*” or evisit* or “m health” or “mhealth” or “mobile counsel#ing” or “mobile consultation*” or “remote consultation*” or “remote counsel#ing” or “tele health*” or telehealth* or “tele consultation*” or teleconsultation* or “tele medicine” or “telemedicine” or “tele rehabilitation” or “telerehabilitation")	10,426
S30	TI (app or apps or cellphone* or computer* or digital or “e mail” or email or internet* or “mobile application*” or online or phone* or smartphone* or technolog* or telephone* or “text message*” or video* or virtual or “web based” or “web site” or website) OR AB (app or apps	498,437

For peer review only - http://bmjopen.bmj.com/site/about/guidelines.xhtml

Karolinska Institutet

	or cellphone* or computer* or digital or “e mail” or email or internet* or “mobile application*” or online or phone* or smartphone* or technolog* or telephone* or “text message*” or video* or virtual or “web based” or “web site” or website) OR KW (app or apps or cellphone* or computer* or digital or “e mail” or email or internet* or “mobile application*” or online or phone* or smartphone* or technolog* or telephone* or “text message*” or video* or virtual or “web based” or “web site” or website)	
S29	DE "Mobile Applications"	2,881
S28	DE "Videoconferencing"	1,138
S27	DE "Digital Technology"	1,488
S26	DE "Telemedicine" OR DE "Online Therapy" OR DE "Teleconferencing" OR DE "Teleconsultation" OR DE "Telepsychiatry" OR DE "Telepsychology" OR DE "Telerehabilitation" OR DE "Teleconferencing" OR DE "Videoconferencing"	13,791
S25	DE "Computer Assisted Therapy"	1,211
S24	DE "Digital Interventions"	1,160
S23	DE "Internet"	38,434
S22	S17 OR S18 OR S19 OR S20 OR S21	375,079
S21	TI (((affective or mood) N2 disorder*)) OR AB (((affective or mood) N2 disorder*)) OR KW (((affective or mood) N2 disorder*))	41,246
S20	TI depress* OR AB depress* OR KW depress*	349,996
S19	DE "Depression (Emotion)"	26,821
S18	DE "Major Depression" OR DE "Anaclitic Depression" OR DE "Dysthymic Disorder" OR DE "Endogenous Depression" OR DE "Late Life Depression" OR DE "Postpartum Depression" OR DE "Reactive Depression" OR DE "Recurrent Depression" OR DE "Treatment Resistant Depression"	154,908
S17	DE "Affective Disorders"	15,668
S16	TI (agoraphobi* or anxi* or obsessive-compulsive or panic or phobi* or “post-traumatic stress” or “posttraumatic stress”)) OR AB (agoraphobi* or anxi* or obsessive-compulsive or panic or phobi* or “post-traumatic stress” or “posttraumatic stress”)) OR KW (agoraphobi* or anxi* or obsessive-compulsive or panic or phobi* or “post-traumatic stress” or “posttraumatic stress”))	310,917
S15	DE "Anxiety"	94,782
S14	DE "Posttraumatic Stress Disorder"	38,806
S13	DE "Phobias" OR DE "Acrophobia" OR DE "Agoraphobia" OR DE "Claustrophobia" OR DE "Ophidiophobia" OR DE "School Phobia" OR DE "Social Phobia"	14,824
S12	DE "Panic Disorder"	9,312
S11	DE "Obsessive Compulsive Disorder"	15,724
S10	DE "Neurosis"	7,143
S9	DE "Agoraphobia"	3,756
S8	DE "Anxiety Disorders"	34,816
S7	S1 OR S2 OR S3 OR S4 OR S5 OR S6	579,216
S6	TI (“behavio#ral activation” or “bias modification” or “cognitive restructuring” or hypno* or meditation or mindfulness or “problem solving” or psychoeducation* or psychotherap* or relaxation or self-management or “unified protocol”) OR AB (“behavio#ral activation” or “bias modification” or “cognitive restructuring” or hypno* or meditation or mindfulness or “problem solving” or psychoeducation* or psychotherap* or relaxation or self-management or “unified protocol”) OR KW (“behavio#ral activation” or “bias modification” or “cognitive restructuring” or hypno* or meditation or mindfulness or “problem solving” or psychoeducation* or psychotherap* or relaxation or self-management or “unified protocol”)	256,300
S5	TI ((("acceptance and commitment" or affect-focused or behavio#r* or biofeedback or computeri* or cognit* or digital or "emotional awareness and expression" or educat* or exposure or guided or internet* or interpersonal or mobile or online or positive psychology or psychodynamic or psychologic* or self-guided or self-help or transdiagnost* or web*) N2	229,986

For peer review only - http://bmjopen.bmj.com/site/about/guidelines.xhtml

Karolinska Institutet

	(intervention* or therap* or treat* or program*))) OR AB ((("acceptance and commitment" or affect-focused or behavio#r* or biofeedback or computeri* or cognit* or digital or "emotional awareness and expression" or educat* or exposure or guided or internet* or interpersonal or mobile or online or positive psychology or psychodynamic or psychologic* or self-guided or self-help or transdiagnost* or web*) N2 (intervention* or therap* or treat* or program*))) OR KW ((("acceptance and commitment" or affect-focused or behavio#r* or biofeedback or computeri* or cognit* or digital or "emotional awareness and expression" or educat* or exposure or guided or internet* or interpersonal or mobile or online or positive psychology or psychodynamic or psychologic* or self-guided or self-help or transdiagnost* or web*) N2 (intervention* or therap* or treat* or program*)))	
S4	DE "Cognitive Behavior Therapy" OR DE "Acceptance and Commitment Therapy" OR DE "Cognitive Processing Therapy" OR DE "Prolonged Exposure Therapy"	26,182
S3	DE "Behavior Therapy" OR DE "Aversion Therapy" OR DE "Conversion Therapy" OR DE "Dialectical Behavior Therapy" OR DE "Exposure Therapy" OR DE "Implosive Therapy" OR DE "Reciprocal Inhibition Therapy" OR DE "Response Cost" OR DE "Systematic Desensitization Therapy" OR DE "Aversion Therapy" OR DE "Covert Sensitization" OR DE "Exposure Therapy" OR DE "Imaginal Exposure" OR DE "Implosive Therapy" OR DE "In Vivo Exposure" OR DE "Prolonged Exposure Therapy" OR DE "Systematic Desensitization Therapy" OR DE "Virtual Reality Exposure Therapy"	34,636
S2	DE "Cognitive Therapy"	24,241
S1	DE "Psychotherapy" OR DE "Adlerian Psychotherapy" OR DE "Adolescent Psychotherapy" OR DE "Affirmative Therapy" OR DE "Analytical Psychotherapy" OR DE "Autogenic Training" OR DE "Brief Psychotherapy" OR DE "Brief Relational Therapy" OR DE "Child Psychotherapy" OR DE "Client Centered Therapy" OR DE "Conversion Therapy" OR DE "Couples Therapy" OR DE "Eclectic Psychotherapy" OR DE "Emotion Focused Therapy" OR DE "Existential Therapy" OR DE "Experiential Psychotherapy" OR DE "Expressive Psychotherapy" OR DE "Eye Movement Desensitization Therapy" OR DE "Feminist Therapy" OR DE "Geriatric Psychotherapy" OR DE "Gestalt Therapy" OR DE "Group Psychotherapy" OR DE "Guided Imagery" OR DE "Humanistic Psychotherapy" OR DE "Hypnotherapy" OR DE "Individual Psychotherapy" OR DE "Insight Therapy" OR DE "Integrative Psychotherapy" OR DE "Interpersonal Psychotherapy" OR DE "Logotherapy" OR DE "Narrative Therapy" OR DE "Network Therapy" OR DE "Persuasion Therapy" OR DE "Primal Therapy" OR DE "Psychoanalysis" OR DE "Psychodrama" OR DE "Psychodynamic Psychotherapy" OR DE "Psychotherapeutic Counseling" OR DE "Psychotherapeutic Techniques" OR DE "Rational Emotive Behavior Therapy" OR DE "Reality Therapy" OR DE "Relationship Therapy" OR DE "Solution Focused Therapy" OR DE "Strategic Therapy" OR DE "Supportive Psychotherapy" OR DE "Transactional Analysis" OR DE "Adolescent Psychotherapy" OR DE "Multisystemic Therapy" OR DE "Child Psychotherapy" OR DE "Play Therapy" OR DE "Gestalt Therapy" OR DE "Empty Chair Technique" OR DE "Group Psychotherapy" OR DE "Encounter Group Therapy" OR DE "Therapeutic Community" OR DE "Humanistic Psychotherapy" OR DE "Client Centered Therapy" OR DE "Hypnotherapy" OR DE "Age Regression (Hypnotic)" OR DE "Ericksonian Psychotherapy" OR DE "Posthypnotic Suggestions" OR DE "Integrative Psychotherapy" OR DE "Schema Therapy" OR DE "Psychoanalysis" OR DE "Adlerian Psychotherapy" OR DE "Brief Relational Therapy" OR DE "Dream Analysis" OR DE "Self-Analysis" OR DE "Psychotherapeutic Counseling" OR DE "Family Therapy" OR DE "Psychotherapeutic Techniques" OR DE "Active Listening" OR DE "Animal Assisted Therapy" OR DE "Autogenic Training" OR DE "Brief Relational Therapy" OR DE "Centering" OR DE "Cotherapy" OR DE "Dream Analysis" OR DE "Empty Chair Technique" OR DE "Ericksonian Psychotherapy" OR DE "Free Association" OR DE "Guided Imagery" OR DE "Life Review" OR DE "Mirroring" OR DE "Morita Therapy" OR DE "Motivational Interviewing" OR DE "Mutual Storytelling Technique" OR DE "Network Therapy" OR DE "Paradoxical Techniques" OR DE "Psychodrama" OR DE "Strategic Therapy" OR DE "Strategic Family Therapy"	237,062

For peer review only - http://bmjopen.bmj.com/site/about/guidelines.xhtml

Internet-delivered transdiagnostic psychological treatments for individuals with depression, anxiety, or both: a systematic review with meta-analysis of randomized controlled trials

Randomized controlled trials per year of publication

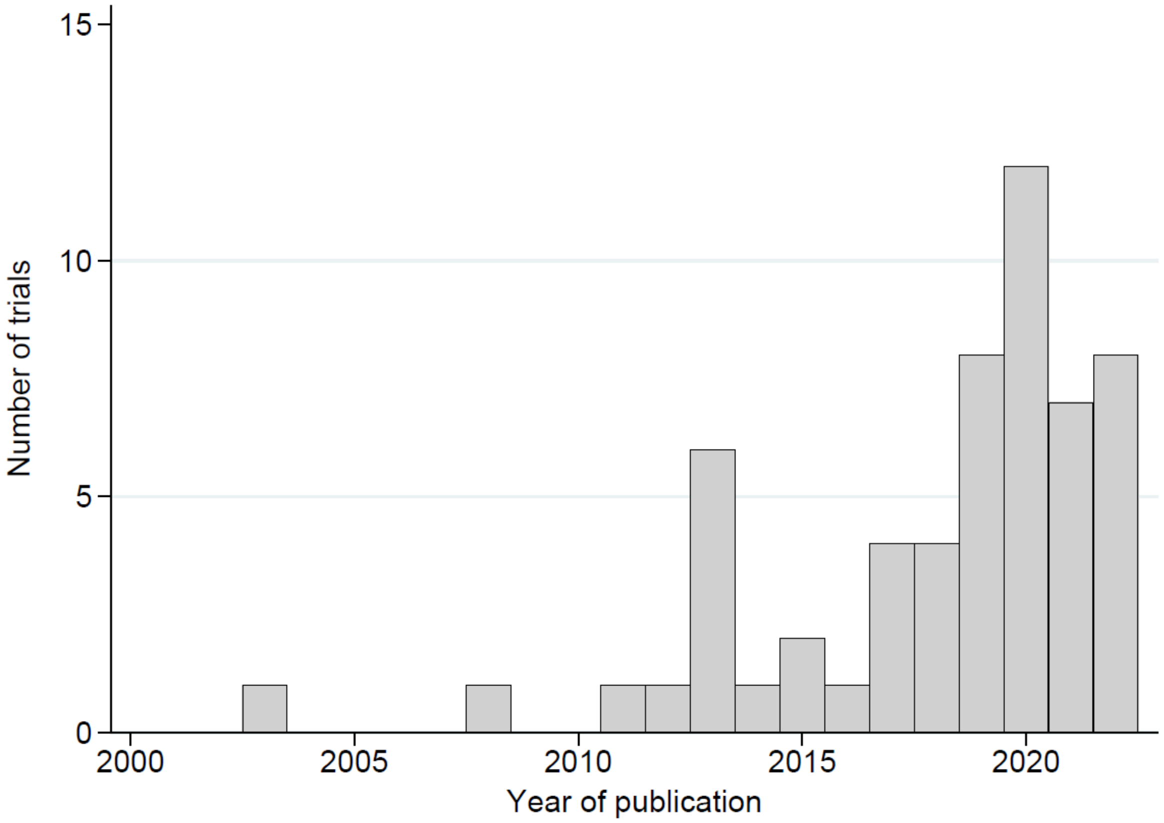

Internet-delivered transdiagnostic psychological treatments for individuals with depression, anxiety, or both: a systematic review with meta-analysis of randomized controlled trials

Exclusion criteria discussed with regard to disagreements

There were 45 disagreements:

- 18 concerned criterion d, i.e., whether the study recruited a mix of participants suffering from clinically significant depression and anxiety
- 15 concerned criterion c, i.e., whether the study evaluated the efficacy of an internet-delivered transdiagnostic psychological treatment for anxiety and depression
- 6 concerned criterion e, i.e., whether the sample composition was such that it was unreasonable to generalize to typical patients with depression and anxiety disorders
- 4 concerned criterion b, i.e., whether the text under consideration was a primary publication from a randomized controlled trial
- 1 concerned criterion a, i.e., whether the text under consideration was a peer reviewed article written in English
- 1 concerned criterion f, i.e., whether there was at least 10 participants in at least one internet-delivered transdiagnostic psychological treatment arm

For peer review only

Internet-delivered transdiagnostic psychological treatments for individuals with depression, anxiety, or both: a systematic review with meta-analysis of randomized controlled trials

Trial characteristics

Table S.B.1. Trial characteristics including references

Reference	Year	Country	Age, M	Women, %	N	Duration	Format	Treatment type/school	Missing, %
Al-Refae, et al. [1]	2021	Canada	25	79	245	< 6 weeks	app	mixed CBT/mindfulness	41
Batterham, et al. [2]	2021	Australia		85	1986	< 6 weeks	mixed/other	CBT	66
Beshai, et al. [3]	2020	Canada	35	44	456	< 6 weeks	mixed/other	mindfulness based	65
Borjalilu, et al. [4]	2019	Iran	24	71	68	≥ 6 weeks	app	mindfulness based	
Carolan, et al. [5]	2017	United Kingdom	41	85	84	≥ 6 weeks	website	CBT	26
Dear, et al. [6]	2018	Australia	21	82	217	< 6 weeks	website	CBT	28
Diaz-Garcia, et al. [7]	2021	Spain	34	72	216	≥ 6 weeks	mixed/other	CBT	32
Farrer, et al. [8]	2019	Australia	22	78	200	≥ 6 weeks	mixed/other	mixed CBT/mindfulness	28
Fitzpatrick, et al. [9]	2017	United States	22	67	70	< 6 weeks	mixed/other	CBT	20
Gonzalez-Robles, et al. [10]	2020	Spain	38	69	214	≥ 6 weeks	website	CBT	39
Graham, et al. [11]	2020	United States	42	82	146	≥ 6 weeks	app	CBT	4
Gregoire, et al. [12]	2022	Canada	26	77	107	< 6 weeks	video	CBT	31
Hadjistavropoulos, et al. [13]	2017	Canada	38	79	182	≥ 6 weeks	website	CBT	25
Hadjistavropoulos, et al. [14]	2020a	Canada	37	76	744	≥ 6 weeks	website	CBT	31
Hadjistavropoulos, et al. [15]	2020b	Canada	37	72	631	≥ 6 weeks	website	CBT	35
Hadjistavropoulos, et al. [16]	2022	Canada	36	76	469	≥ 6 weeks	website	CBT	27
Hensel, et al. [17]	2019	Canada	41	73, 78	812	≥ 6 weeks	website	CBT	45
Hirsch, et al. [18]	2020	United Kingdom	29	83	178	< 6 weeks	website	other	12
Hoek, et al. [19]	2012	The Netherlands	16	76	45	< 6 weeks	website	other	38
Hosseinzadeh Asl [20]	2022	Turkey	33	55	59	< 6 weeks	video	mindfulness based	17
Isbăşoiu, et al. [21]	2021	Romania	33	85	284	≥ 6 weeks	website	CBT	45

For peer review only - http://bmjopen.bmj.com/site/about/guidelines.xhtml

Internet-delivered transdiagnostic psychological treatments for individuals with depression, anxiety, or both: a systematic review with meta-analysis of randomized controlled trials

Johansson, et al. [22]	2013	Sweden	45	82	100	≥ 6 weeks	website	other	0
Kang, et al. [23]	2022	China	37	53	100	< 6 weeks	mixed/other	mindfulness based	0
Kladnitski, et al. [24]	2020	Australia	39	86	158	≥ 6 weeks	website	mixed CBT/mindfulness	27
Kleiboer, et al. [25]	2015	The Netherlands	45	65	537	< 6 weeks	website	other	22
Krafft, et al. [26]	2019	United States	25	66	35	< 6 weeks	app	CBT	60
Levin, et al. [27]	2022	United States	20	100	23	< 6 weeks	app	mindfulness based	30
Lindegaard, et al. [28]	2021	Sweden	38	42	59	≥ 6 weeks	website	CBT	39
McCloud, et al. [29]	2020	United Kingdom	24	83	168	≥ 6 weeks	app	CBT	42
Mohr, et al. [30]	2019	United States	36	76	301	≥ 6 weeks	app	CBT	3
Mullin, et al. [31]	2015	Australia	28	64	55	≥ 6 weeks	website	CBT	24
Newby, et al. [32]	2013	Australia	44	78	109	≥ 6 weeks	website	CBT	12
Nilsson, et al. [33]	2019	Sweden			837	≥ 6 weeks	website	other	67
Osborn, et al. [34]	2020	Kenya	15	64	103	< 6 weeks	website	other	0
Peynenburg, et al. [35]	2022	Canada	24	81	308	< 6 weeks	website	CBT	29
Powell, et al. [36]	2012	United Kingdom	41	78	3070	≥ 6 weeks	website	CBT	42
Proudfoot, et al. [37]	2013	Australia	39	70	720	≥ 6 weeks	website	other	28
Robichaud, et al. [38]	2020	Canada	35	87	63	≥ 6 weeks	website	CBT	32
Rogers and Sicouri [39]	2022	Australia	20	55	45	< 6 weeks	website	other	3
Rollman, et al. [40]	2018	United States	43	80	704	≥ 6 weeks	website	CBT	14
Schaeuffele, et al. [41]	2022	Germany	37	68	132	≥ 6 weeks	website	CBT	25
Sethi [42]	2013	Australia	20	58	89	< 6 weeks	website	CBT	0
Soucy, et al. [43]	2021	Canada	38	75	480	≥ 6 weeks	website	CBT	30
Southwell and Gould [44]	2016	Australia	34	88	120	< 6 weeks	website	other	52
Taylor, et al. [45]	2022	United Kingdom	41	83	2182	< 6 weeks	app	mindfulness based	35
Terides, et al. [46]	2018	Australia	45	88, 75	148	≥ 6 weeks	website	CBT	18
Titov, et al. [47]	2011	Australia	44	73	77	≥ 6 weeks	website	CBT	10

For peer review only

Internet-delivered transdiagnostic psychological treatments for individuals with depression, anxiety, or both: a systematic review with meta-analysis of randomized controlled trials

Titov, et al. [48]	2013	Australia	41	74	274	≥ 6 weeks	website	CBT	20
Titov, et al. [49]	2016	Australia	66	64	459	≥ 6 weeks	website	CBT	9
Tönnies, et al. [50]	2021	Germany	49	70	50	≥ 6 weeks	video	other	10
Tulbure, et al. [51]	2018	Romania	34	81	105	≥ 6 weeks	website	CBT	8
Twomey, et al. [52]	2014	Ireland	35	74	201	< 6 weeks	website	CBT	67
van Straten, et al. [53]	2008	The Netherlands	45	71	213	< 6 weeks	website	other	17
Villemaire-Krajden and Myhr [54]	2019	Canada	38	67	67	≥ 6 weeks	mixed/other	CBT	31
Viravan, et al. [55]	2022	Thailand	34	32	80	< 6 weeks	mixed/other	mindfulness based	4
Viskovich and Pakenham [56]	2020	Australia	27	68	2110	< 6 weeks	mixed/other	CBT	68
Zetterqvist, et al. [57]	2003	Sweden	39	57, 65	100	≥ 6 weeks	website	CBT	37

For peer review only

Internet-delivered transdiagnostic psychological treatments for individuals with depression, anxiety, or both: a systematic review with meta-analysis of randomized controlled trials

Publication bias

We explored indicators of publication bias in the meta-analysis of effects versus rudimentary passive controls. For depression, the funnel plot (see below) appeared to indicate that small- to medium-sized trials with non-beneficial effects of internet-delivered psychological treatments were underreported. This asymmetry was confirmed by Egger's test (z = 2.24, p = 0.025). The trim-and-fill procedure resulted in different outcomes depending on the choice of estimator. With the R0 estimator ($p < 0.001$), 19 studies were imputed, and the between-group effect dropped from 0.52 to 0.29 (95% CI 0.16-0.42). In contrast, the L0 and Q0 estimators resulted in no imputation. Visual inspection of the funnel plot for anxiety (see below) was also indicative of possible asymmetry in the same direction, but this was more ambiguous. Egger's test was significant (z = 2.64, p = 0.008) but the trim-and-fill procedure did not result in imputation, regardless of estimator. As is recommended in high heterogeneity situations,[58] we repeated all tests of publication bias in a manner informed by the moderator analyses and results were similar (see below).

For peer review only

Internet-delivered transdiagnostic psychological treatments for individuals with depression, anxiety, or both: a systematic review with meta-analysis of randomized controlled trials

Meta-analysis versus rudimentary passive controls, with depression symptoms as outcome

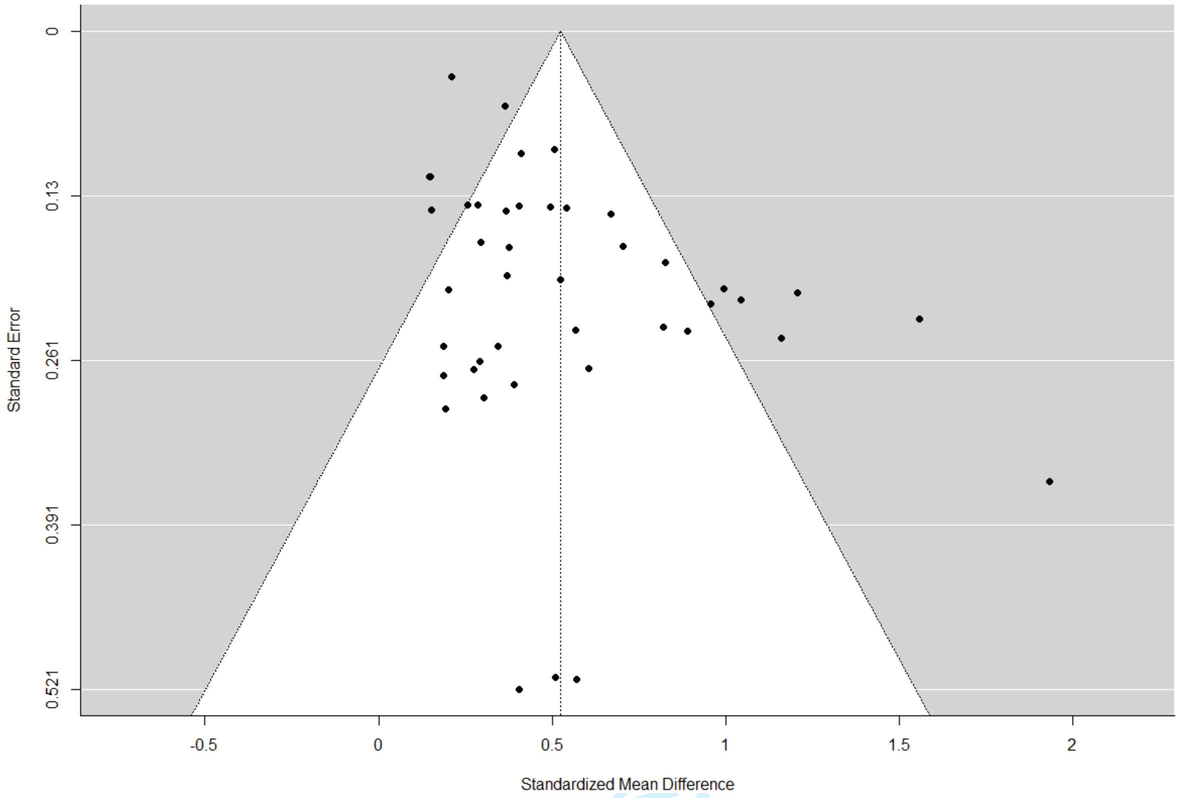

For peer review only - http://bmjopen.bmj.com/site/about/guidelines.xhtml

Internet-delivered transdiagnostic psychological treatments for individuals with depression, anxiety, or both: a systematic review with meta-analysis of randomized controlled trials

Meta-analysis versus rudimentary passive controls, with depression symptoms as outcome, with missing trials imputed based on the trim-and-fill procedure and the R0 estimator

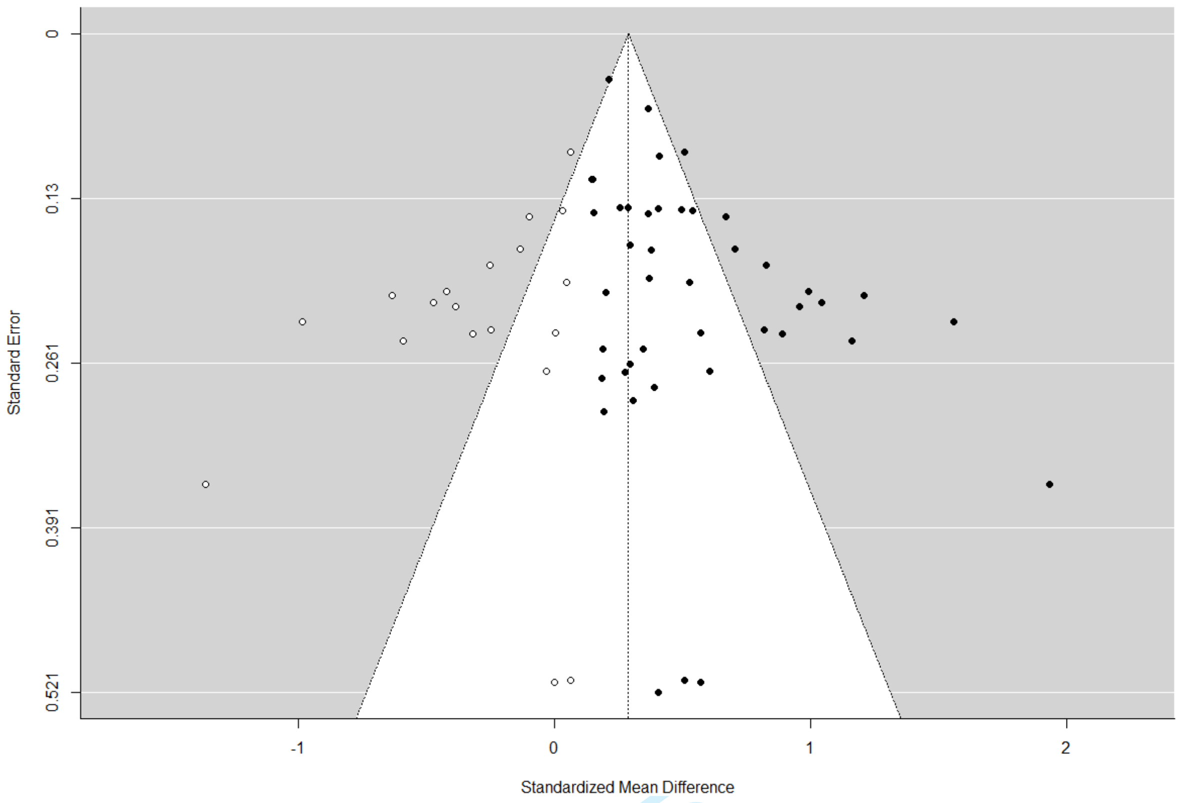

Internet-delivered transdiagnostic psychological treatments for individuals with depression, anxiety, or both: a systematic review with meta-analysis of randomized controlled trials

Meta-analysis versus rudimentary passive controls, with anxiety as outcome

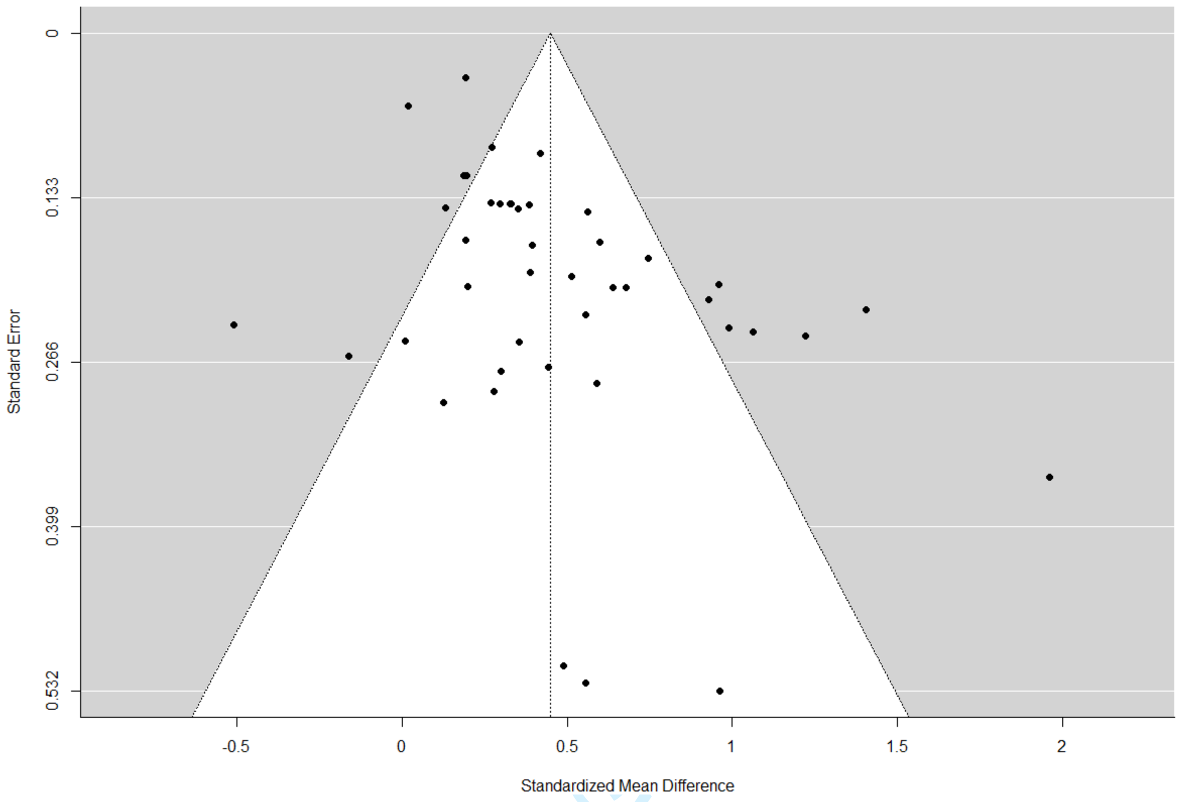

Internet-delivered transdiagnostic psychological treatments for individuals with depression, anxiety, or both: a systematic review with meta-analysis of randomized controlled trials

Risk of bias re-assessed separately based on whether trials required participants to meet full criteria for formal psychiatric diagnosis or not

Asymmetry of the funnel plot may result from several mechanisms. Because heterogeneity in the main analyses was substantial, and the moderator analyses indicated that the only significant moderator of the between-group effect versus rudimentary passive controls was whether trials required all participants to meet full criteria for a formal diagnosis or not, we repeated our investigation of the funnel plot for these substrata of the trials. That is, we first assessed whether publication bias appeared to be present in trials that employed psychiatric diagnoses, and then assessed whether publication bias appeared to be present in trials that did not employ psychiatric diagnoses. The results are tabulated in the table below. Overall, even when the sample of trials was split along this only significant effect moderator (i.e., psychiatric diagnoses or not), the outcome of the analyses of publication bias was still contradictory and similar to the equivalent tests of all trials.

Table S.B.2. Sensitivity analyses pertaining to publication bias

**Subset of trials**	**Outcome**	**Visual inspection**	**Egger's test**	**Trim-and-fill**
Psychiatric diagnosis (k = 12)	Depression	Possibly slight bias, but not clear	z = 3.67, p < 0.001	R0: p = 0.250, but 1 trial imputed<br>L0: no imputation<br>Q0: no imputation
Psychiatric diagnosis (k = 12)	Anxiety	No clear sign of bias	z = 2.08, p = 0.038	R0: p = 0.500, no imputation<br>L0: no imputation<br>Q0: no imputation
Cut-off or symptoms (k = 31)	Depression	Possibly slight bias, but not clear	z = 0.82, p = 0.410	R0: p = 0.004, and 7 trials imputed<br>L0: no imputation<br>Q0: no imputation
Cut-off or symptoms (k = 31)	Anxiety	Possibly slight bias, but not clear	z = 2.31, p = 0.021	R0: p = 0.500, no imputation<br>L0: 9 trials imputed<br>Q0: 12 trials imputed

For peer review only

Internet-delivered transdiagnostic psychological treatments for individuals with depression, anxiety, or both: a systematic review with meta-analysis of randomized controlled trials

Non-planned sensitivity analyses of psychiatric diagnosis as an effect moderator

One moderator was found to be significant for both depression and anxiety symptoms: trials that required all participants to meet full criteria for a psychiatric diagnosis reported a larger pooled between-group effects versus rudimentary passive controls, as compared to trials that did not have this requirement. We conducted a non-planned sensitivity analysis to ensure that this effect was not driven merely by participants in control groups of the routine clinic being more likely to be enrolled other treatments, by testing the moderating effect of diagnosis on the pooled within-group effect. This test illustrated that psychiatric diagnosis remained a significant effect moderator also with regard to within-group effects (depression: 1.10 vs. 0.75, $p = 0.001$; anxiety: 1.14 vs. 0.63, $p < 0.001$). We also reviewed studies that used the most common depression and anxiety outcome respectively (the PHQ-9 and GAD-7) to determine if the effect of diagnosis was driven primarily by a difference in the mean symptom reduction, or by a smaller standard deviation. Though the average standard deviation was slightly smaller in studies that required participants to meet full criteria for a diagnosis, the reduction in the mean was also larger. In summary, requiring participants to meet full criteria for psychiatric diagnosis appeared to be robust effect moderator.

For peer review only

Internet-delivered transdiagnostic psychological treatments for individuals with depression, anxiety, or both: a systematic review with meta-analysis of randomized controlled trials

Randomized controlled trials versus other bona fide treatments

Two trials compared the effects of an internet-delivered transdiagnostic psychological treatment to those of a distinctly different bona fide treatment. One trial compared mindfulness-based stress reduction delivered via a mobile app (n = 28) to a face-to-face equivalent (n = 20) for university students and reported non-significant between-group effects (depression: g = -0.49; anxiety: g = -0.13).[4] One trial compared web-based CBT (n = 23) to a face-to-face equivalent (n = 21) for university students and reported a significant and large between-group effect on depression in favor of the face-to-face treatment (g = -1.39) and a non-significant and small between-group effect on anxiety (g = -0.21).[42] Last, we also identified one trial that was on the verge of being a bona-fide comparison. In this trial, web-based CBT (n = 33) was compared to a condition which was described as a waitlist, but where participants had access to an unguided CBT bibliotherapy which we deemed to be relatively credible (n = 34). There were no significant between-group effects on depression (g = 0.33) or anxiety (g = -0.13).[54]

For peer review only

Internet-delivered transdiagnostic psychological treatments for individuals with depression, anxiety, or both: a systematic review with meta-analysis of randomized controlled trials

Randomized factorial trials

Nine randomized factorial trials explored moderators of the overall effect of internet-delivered transdiagnostic psychological treatments. Seven of these trials evaluated the same treatment protocol: the Wellbeing Course. The factors investigated, and the main outcome of each of the nine randomized factorial trials is tabulated below:

Table S.B.3. Randomized factorial trials of internet-delivered transdiagnostic psychological treatments suitable for both depression and anxiety

Reference	N	Protocol	Factors	Summary of main outcome
Dear *et al.* (2018)[6]	217	Wellbeing Course	Therapist-guided vs. unguided	Mostly no significant mean differences in the clinical outcome, with small between-group effects on depression and anxiety at post-treatment.
Hadjistavropoulos *et al.* (2017)[13]	182	Wellbeing Course	Weekly support vs. optional support	No significant mean differences in the clinical outcome, with small between-group effects on depression and anxiety at post-treatment. Rates of treatment completion were significantly higher with weekly support.
Hadjistavropoulos *et al.* (2020a)[14]	744	Wellbeing Course	Weekly support by ICBT specialist vs. weekly support plus on-demand support by ICBT specialist vs. weekly support by other clinician	Mostly no significant mean differences in the clinical outcome, with small between-group effects on depression and anxiety at post-treatment. Rates of response in depression were lower with support from other clinicians.
Hadjistavropoulos *et al.* (2020b)[15]	631	Wellbeing Course	Use of homework reflection vs. no use of homework reflection<br><br>Weekly support vs. twice-weekly support<br><br>ICBT clinic vs. community mental health clinic	No significant mean differences in the clinical outcome, with small between-group effects on depression and anxiety at post-treatment.
Hadjistavropoulos *et al.* (2022)[16]	469	Wellbeing Course	Optional 4-week extension of support vs. no such option<br><br>Optional booster lesson vs. no such option	No significant mean differences in the clinical outcome, with small between-group effects on depression and anxiety at post-treatment. About 52% opted to make use of the booster lesson.

For peer review only

Internet-delivered transdiagnostic psychological treatments for individuals with depression, anxiety, or both: a systematic review with meta-analysis of randomized controlled trials

Isbăşoiu *et al.* (2021)[21]	284	Unified protocol	Addition of self-enhancement and emotion regulation components vs. no such components	No significant mean differences in the clinical outcome, with small between-group effects on depression and anxiety at post-treatment.
Mohr *et al.* (2019)[30]	301	IntelliCare	Therapist-guided vs. unguided *<br><br>Weekly app recommendations vs. no such recommendations *	Guidance led to a significantly larger mean effect on anxiety (but not depression). Recommendations led to a significantly larger mean effect on depression (but not anxiety). Between-group effects were small.
Peynenburg *et al.* (2022)[35]	308	Wellbeing Course	Internet-delivered exercises based on motivational interviewing before treatment vs. no such exercises *<br><br>Optional booster lesson vs. no such option	Internet-delivered exercises based on motivational interviewing before treatment led to a significantly larger mean effect on depression and anxiety. Having the option of a booster lesson did not lead to significantly larger mean effects. Between-group effects were small. About 32% opted to make use of the booster lesson.
Soucy *et al.* (2021)[43]	480	Wellbeing Course	Internet-delivered exercises based on motivational interviewing before treatment vs. no such exercises	No significant mean differences in the clinical outcome, with small between-group effects on depression and anxiety at post-treatment.

* This factor was a significant moderator of the effect on depression symptoms or anxiety.

For peer review only

Internet-delivered transdiagnostic psychological treatments for individuals with depression, anxiety, or both: a systematic review with meta-analysis of randomized controlled trials

Brief interventions

Two trials evaluated brief interventions (≤2 weeks) for adults. One trial (*N*=70) found that 2 weeks of communication with an automated chatbot trained in active listening and CBT strategies was associated with a small to moderate between-group effect on depression (g = 0.37) but no significant between-group effect on anxiety as compared to a waitlist.[9] The second trial (*N*=45) reported null effects of a single session of cognitive bias modification on depression (g = -0.04) and anxiety (g = 0.42), compared to a psychological placebo.[39]

For peer review only

Internet-delivered transdiagnostic psychological treatments for individuals with depression, anxiety, or both: a systematic review with meta-analysis of randomized controlled trials

References

1. Al-Refae M, Al-Refae A, Munroe M, et al. A Self-Compassion and Mindfulness-Based Cognitive Mobile Intervention (Serene) for Depression, Anxiety, and Stress: Promoting Adaptive Emotional Regulation and Wisdom. *Front Psychol* 2021;12:648087. doi: 10.3389/fpsyg.2021.648087 [published Online First: 2021/04/09]
2. Batterham PJ, Calear AL, Farrer L, et al. Efficacy of a Transdiagnostic Self-Help Internet Intervention for Reducing Depression, Anxiety, and Suicidal Ideation in Adults: Randomized Controlled Trial. *J Med Internet Res* 2021;23(1):e22698. doi: 10.2196/22698 [published Online First: 2021/01/23]
3. Beshai S, Bueno C, Yu M, et al. Examining the effectiveness of an online program to cultivate mindfulness and self-compassion skills (Mind-OP): Randomized controlled trial on Amazon's Mechanical Turk. *Behav Res Ther* 2020;134:103724. doi: 10.1016/j.brat.2020.103724 [published Online First: 2020/09/18]
4. Borjalilu S, Mazaheri MA, Talebpour A. Effectiveness of Mindfulness-Based Stress Management in The Mental Health of Iranian University Students: A Comparison of Blended Therapy, Face-to-Face Sessions, and mHealth App (Aramgar). *Iranian Journal of Psychiatry and Behavioral Sciences* 2019;13(2) doi: 10.5812/ijpbs.84726
5. Carolan S, Harris PR, Greenwood K, et al. Increasing engagement with an occupational digital stress management program through the use of an online facilitated discussion group: Results of a pilot randomised controlled trial. *Internet Interv* 2017;10:1-11. doi: 10.1016/j.invent.2017.08.001 [published Online First: 2018/08/24]
6. Dear BF, Fogliati VJ, Fogliati R, et al. Treating anxiety and depression in young adults: A randomised controlled trial comparing clinician-guided versus self-guided Internet-delivered cognitive behavioural therapy. *Aust N Z J Psychiatry* 2018;52(7):668-79. doi: 10.1177/0004867417738055 [published Online First: 2017/10/25]
7. Diaz-Garcia A, Gonzalez-Robles A, Garcia-Palacios A, et al. Negative and Positive Affect Regulation in a Transdiagnostic Internet-Based Protocol for Emotional Disorders: Randomized Controlled Trial. *J Med Internet Res* 2021;23(2):e21335. doi: 10.2196/21335 [published Online First: 2021/02/02]
8. Farrer LM, Gulliver A, Katruss N, et al. A novel multi-component online intervention to improve the mental health of university students: Randomised controlled trial of the Uni Virtual Clinic. *Internet Interv* 2019;18:100276. doi: 10.1016/j.invent.2019.100276 [published Online First: 2020/01/01]
9. Fitzpatrick KK, Darcy A, Vierhile M. Delivering Cognitive Behavior Therapy to Young Adults With Symptoms of Depression and Anxiety Using a Fully Automated Conversational Agent (Woebot): A Randomized Controlled Trial. *JMIR Ment Health* 2017;4(2):e19. doi: 10.2196/mental.7785 [published Online First: 2017/06/08]
10. Gonzalez-Robles A, Diaz-Garcia A, Garcia-Palacios A, et al. Effectiveness of a Transdiagnostic Guided Internet-Delivered Protocol for Emotional Disorders Versus Treatment as Usual in Specialized Care: Randomized Controlled Trial. *J Med Internet Res* 2020;22(7):e18220. doi: 10.2196/18220 [published Online First: 2020/07/17]
11. Graham AK, Greene CJ, Kwasny MJ, et al. Coached Mobile App Platform for the Treatment of Depression and Anxiety Among Primary Care Patients: A Randomized Clinical Trial. *JAMA Psychiatry* 2020;77(9):906-14. doi: 10.1001/jamapsychiatry.2020.1011 [published Online First: 2020/05/21]
12. Gregoire S, Beaulieu F, Lachance L, et al. An online peer support program to improve mental health among university students: A randomized controlled trial. *J Am Coll*

For peer review only

Health 2022:1-13. doi: 10.1080/07448481.2022.2099224 [published Online First: 2022/08/10]
13. Hadjistavropoulos HD, Schneider LH, Edmonds M, et al. Randomized controlled trial of internet-delivered cognitive behaviour therapy comparing standard weekly versus optional weekly therapist support. *J Anxiety Disord* 2017;52:15-24. doi: 10.1016/j.janxdis.2017.09.006 [published Online First: 2017/10/02]
14. Hadjistavropoulos HD, Peynenburg V, Nugent M, et al. Transdiagnostic Internet-delivered cognitive behaviour therapy with therapist support offered once-weekly or once-weekly supplemented with therapist support within one-business-day: Pragmatic randomized controlled trial. *Internet Interv* 2020;22:100347. doi: 10.1016/j.invent.2020.100347 [published Online First: 20200831]
15. Hadjistavropoulos HD, Peynenburg V, Thiessen DL, et al. A pragmatic factorial randomized controlled trial of transdiagnostic internet-delivered cognitive behavioural therapy: Exploring benefits of homework reflection questionnaires and twice-weekly therapist support. *Internet Interv* 2020;22:100357. doi: 10.1016/j.invent.2020.100357 [published Online First: 20201201]
16. Hadjistavropoulos HD, Peynenburg V, Thiessen DL, et al. A randomized factorial trial of internet-delivered cognitive behavioural therapy: An 8-week program with or without extended support and booster lesson. *Internet Interv* 2022;27:100499. doi: 10.1016/j.invent.2022.100499 [published Online First: 20220206]
17. Hensel JM, Shaw J, Ivers NM, et al. A Web-Based Mental Health Platform for Individuals Seeking Specialized Mental Health Care Services: Multicenter Pragmatic Randomized Controlled Trial. *J Med Internet Res* 2019;21(6):e10838. doi: 10.2196/10838 [published Online First: 2019/06/06]
18. Hirsch CR, Krahe C, Whyte J, et al. Effects of modifying interpretation bias on transdiagnostic repetitive negative thinking. *J Consult Clin Psychol* 2020;88(3):226-39. doi: 10.1037/ccp0000455 [published Online First: 2020/02/19]
19. Hoek W, Schuurmans J, Koot HM, et al. Effects of Internet-based guided self-help problem-solving therapy for adolescents with depression and anxiety: a randomized controlled trial. *PLoS One* 2012;7(8):e43485. doi: 10.1371/journal.pone.0043485 [published Online First: 2012/09/07]
20. Hosseinzadeh Asl NR. A randomized controlled trial of a mindfulness-based intervention in social workers working during the COVID-19 crisis. *Curr Psychol* 2022;41(11):8192-99. doi: 10.1007/s12144-021-02150-3 [published Online First: 2021/08/17]
21. Isbăşoiu AB, Tulbure BT, Rusu A, et al. Can We Boost Treatment Adherence to an Online Transdiagnostic Intervention by Adding Self-Enhancement Strategies? Results From a Randomized Controlled Non-inferiority Trial. *Frontiers in psychology* 2021;12:752249. doi: 10.3389/fpsyg.2021.752249 [published Online First: 20211202]
22. Johansson R, Bjorklund M, Hornborg C, et al. Affect-focused psychodynamic psychotherapy for depression and anxiety through the Internet: a randomized controlled trial. *PeerJ* 2013;1:e102. doi: 10.7717/peerj.102 [published Online First: 2013/07/19]
23. Kang MY, Nan JKM, Yuan Y. Effectiveness of an online short-term audio-based mindfulness program on negative emotions during the COVID-19 pandemic: Latent growth curve analyses of anxiety and moderated mediation effects of anxiety between mindfulness and negative affect. *Curr Psychol* 2022:1-13. doi: 10.1007/s12144-022-03902-5 [published Online First: 2022/12/06]

For peer review only

Internet-delivered transdiagnostic psychological treatments for individuals with depression, anxiety, or both: a systematic review with meta-analysis of randomized controlled trials

24. Kladnitski N, Smith J, Uppal S, et al. Transdiagnostic internet-delivered CBT and mindfulness-based treatment for depression and anxiety: A randomised controlled trial. *Internet Interv* 2020;20:100310. doi: 10.1016/j.invent.2020.100310 [published Online First: 2020/03/07]
25. Kleiboer A, Donker T, Seekles W, et al. A randomized controlled trial on the role of support in Internet-based problem solving therapy for depression and anxiety. *Behav Res Ther* 2015;72:63-71. doi: 10.1016/j.brat.2015.06.013 [published Online First: 2015/07/21]
26. Krafft J, Potts S, Schoendorff B, et al. A Randomized Controlled Trial of Multiple Versions of an Acceptance and Commitment Therapy Matrix App for Well-Being. *Behav Modif* 2019;43(2):246-72. doi: 10.1177/0145445517748561 [published Online First: 2017/12/22]
27. Levin ME, Hicks ET, Krafft J. Pilot evaluation of the stop, breathe & think mindfulness app for student clients on a college counseling center waitlist. *J Am Coll Health* 2022;70(1):165-73. doi: 10.1080/07448481.2020.1728281 [published Online First: 2020/03/10]
28. Lindegaard T, Seaton F, Halaj A, et al. Internet-based cognitive behavioural therapy for depression and anxiety among Arabic-speaking individuals in Sweden: a pilot randomized controlled trial. *Cogn Behav Ther* 2021;50(1):47-66. doi: 10.1080/16506073.2020.1771414 [published Online First: 2020/07/01]
29. McCloud T, Jones R, Lewis G, et al. Effectiveness of a Mobile App Intervention for Anxiety and Depression Symptoms in University Students: Randomized Controlled Trial. *JMIR Mhealth Uhealth* 2020;8(7):e15418. doi: 10.2196/15418 [published Online First: 2020/08/01]
30. Mohr DC, Schueller SM, Tomasino KN, et al. Comparison of the Effects of Coaching and Receipt of App Recommendations on Depression, Anxiety, and Engagement in the IntelliCare Platform: Factorial Randomized Controlled Trial. *Journal of medical Internet research* 2019;21(8):e13609. doi: 10.2196/13609 [published Online First: 20190828]
31. Mullin A, Dear BF, Karin E, et al. The UniWellbeing course: A randomised controlled trial of a transdiagnostic internet-delivered cognitive behavioural therapy (CBT) programme for university students with symptoms of anxiety and depression. *Internet Interventions* 2015;2(2):128-36. doi: 10.1016/j.invent.2015.02.002
32. Newby JM, Mackenzie A, Williams AD, et al. Internet cognitive behavioural therapy for mixed anxiety and depression: a randomized controlled trial and evidence of effectiveness in primary care. *Psychol Med* 2013;43(12):2635. doi: 10.1017/S0033291713000111
33. Nilsson A, Sorman K, Klingvall J, et al. MyCompass in a Swedish context - lessons learned from the transfer of a self-guided intervention targeting mental health problems. *BMC Psychiatry* 2019;19(1):51. doi: 10.1186/s12888-019-2039-1 [published Online First: 2019/02/02]
34. Osborn TL, Rodriguez M, Wasil AR, et al. Single-session digital intervention for adolescent depression, anxiety, and well-being: Outcomes of a randomized controlled trial with Kenyan adolescents. *J Consult Clin Psychol* 2020;88(7):657-68. doi: 10.1037/ccp0000505 [published Online First: 2020/05/12]
35. Peynenburg V, Hadjistavropoulos H, Thiessen D, et al. Internet-Delivered Cognitive Behavioral Therapy for Postsecondary Students: Randomized Factorial Trial for Examining Motivational Interviewing and Booster Lessons. *Journal of medical*

For peer review only

Internet-delivered transdiagnostic psychological treatments for individuals with depression, anxiety, or both: a systematic review with meta-analysis of randomized controlled trials

Internet research 2022;24(9):e40637. doi: 10.2196/40637 [published Online First: 20220907]

36. Powell J, Hamborg T, Stallard N, et al. Effectiveness of a web-based cognitive-behavioral tool to improve mental well-being in the general population: randomized controlled trial. *J Med Internet Res* 2012;15(1):e2. doi: 10.2196/jmir.2240 [published Online First: 2013/01/11]
37. Proudfoot J, Clarke J, Birch MR, et al. Impact of a mobile phone and web program on symptom and functional outcomes for people with mild-to-moderate depression, anxiety and stress: a randomised controlled trial. *BMC Psychiatry* 2013;13:312. doi: 10.1186/1471-244X-13-312 [published Online First: 2013/11/19]
38. Robichaud M, Talbot F, Titov N, et al. Facilitating access to iCBT: a randomized controlled trial assessing a translated version of an empirically validated program using a minimally monitored delivery model. *Behav Cogn Psychother* 2020;48(2):185-202. doi: 10.1017/S135246581900047X [published Online First: 2019/08/17]
39. Rogers J, Sicouri G. A Single-Session Online Cognitive Bias Modification of Interpretations Modified for Adults With Anxiety and Depressive Symptoms. *Behav Ther* 2022;53(5):967-80. doi: 10.1016/j.beth.2022.04.006 [published Online First: 2022/08/21]
40. Rollman BL, Herbeck Belnap B, Abebe KZ, et al. Effectiveness of Online Collaborative Care for Treating Mood and Anxiety Disorders in Primary Care: A Randomized Clinical Trial. *JAMA Psychiatry* 2018;75(1):56-64. doi: 10.1001/jamapsychiatry.2017.3379 [published Online First: 2017/11/09]
41. Schaeuffele C, Homeyer S, Perea L, et al. The unified protocol as an internet-based intervention for emotional disorders: Randomized controlled trial. *PLoS One* 2022;17(7):e0270178. doi: 10.1371/journal.pone.0270178 [published Online First: 2022/07/12]
42. Sethi S. Treating Youth Depression and Anxiety: A Randomised Controlled Trial Examining the Efficacy of Computerised versus Face-to-face Cognitive Behaviour Therapy. *Australian Psychologist* 2013;48(4):249-57. doi: 10.1111/ap.12006
43. Soucy JN, Hadjistavropoulos HD, Karin E, et al. Brief online motivational interviewing pre-treatment intervention for enhancing internet-delivered cognitive behaviour therapy: A randomized controlled trial. *Internet Interv* 2021;25:100394. doi: 10.1016/j.invent.2021.100394 [published Online First: 20210420]
44. Southwell S, Gould E. A randomised wait list-controlled pre–post–follow-up trial of a gratitude diary with a distressed sample. *The Journal of Positive Psychology* 2016;12(6):579-93. doi: 10.1080/17439760.2016.1221127
45. Taylor H, Cavanagh K, Field AP, et al. Health Care Workers' Need for Headspace: Findings From a Multisite Definitive Randomized Controlled Trial of an Unguided Digital Mindfulness-Based Self-help App to Reduce Healthcare Worker Stress. *JMIR Mhealth Uhealth* 2022;10(8):e31744. doi: 10.2196/31744 [published Online First: 2022/08/26]
46. Terides MD, Dear BF, Fogliati VJ, et al. Increased skills usage statistically mediates symptom reduction in self-guided internet-delivered cognitive-behavioural therapy for depression and anxiety: a randomised controlled trial. *Cogn Behav Ther* 2018;47(1):43-61. doi: 10.1080/16506073.2017.1347195 [published Online First: 2017/07/21]

For peer review only

47. Titov N, Dear BF, Schwencke G, et al. Transdiagnostic internet treatment for anxiety and depression: a randomised controlled trial. *Behav Res Ther* 2011;49(8):441-52. doi: 10.1016/j.brat.2011.03.007 [published Online First: 2011/06/18]
48. Titov N, Dear BF, Johnston L, et al. Improving adherence and clinical outcomes in self-guided internet treatment for anxiety and depression: randomised controlled trial. *PLoS One* 2013;8(7):e62873. doi: 10.1371/journal.pone.0062873 [published Online First: 2013/07/12]
49. Titov N, Fogliati VJ, Staples LG, et al. Treating anxiety and depression in older adults: randomised controlled trial comparing guided v. self-guided internet-delivered cognitive-behavioural therapy. *BJPsych Open* 2016;2(1):50-58. doi: 10.1192/bjpo.bp.115.002139 [published Online First: 2016/10/06]
50. Tönnies J, Hartmann M, Wensing M, et al. Mental Health Specialist Video Consultations Versus Treatment-as-Usual for Patients With Depression or Anxiety Disorders in Primary Care: Randomized Controlled Feasibility Trial. *JMIR Ment Health* 2021;8(3):e22569. doi: 10.2196/22569 [published Online First: 2021/03/13]
51. Tulbure BT, Rusu A, Sava FA, et al. A Web-Based Transdiagnostic Intervention for Affective and Mood Disorders: Randomized Controlled Trial. *JMIR Ment Health* 2018;5(2):e36. doi: 10.2196/mental.8901 [published Online First: 2018/05/26]
52. Twomey C, O'Reilly G, Byrne M, et al. A randomized controlled trial of the computerized CBT programme, MoodGYM, for public mental health service users waiting for interventions. *Br J Clin Psychol* 2014;53(4):433-50. doi: 10.1111/bjc.12055 [published Online First: 2014/05/17]
53. van Straten A, Cuijpers P, Smits N. Effectiveness of a web-based self-help intervention for symptoms of depression, anxiety, and stress: randomized controlled trial. *J Med Internet Res* 2008;10(1):e7. doi: 10.2196/jmir.954 [published Online First: 2008/03/28]
54. Villemaire-Krajden R, Myhr G. Evaluating the Use of a Computerized CBT Program for Outpatients on a Waitlist in a University CBT Unit. *J Psychiatr Pract* 2019;25(4):268-78. doi: 10.1097/PRA.0000000000000396 [published Online First: 2019/07/11]
55. Viravan N, Atsariyasing W, Srifuengfung M, et al. Efficacy of Online Mindfulness Program 'Mindful Senses' for Depression and Anxiety Reduction in Community Samples: A Randomized Controlled Trial. *J Med Assoc Thai* 2022;105(11):1108-19. doi: 10.35755/jmedassocthai.2022.11.13700
56. Viskovich S, Pakenham KI. Randomized controlled trial of a web-based Acceptance and Commitment Therapy (ACT) program to promote mental health in university students. *J Clin Psychol* 2020;76(6):929-51. doi: 10.1002/jclp.22848 [published Online First: 2019/08/31]
57. Zetterqvist K, Maanmies J, Strom L, et al. Randomized controlled trial of internet-based stress management. *Cogn Behav Ther* 2003;32(3):151-60. doi: 10.1080/16506070302316 [published Online First: 2005/11/18]
58. Shi L, Lin L. The trim-and-fill method for publication bias: practical guidelines and recommendations based on a large database of meta-analyses. *Medicine* 2019;98(23)

Outcome / reserch question	RCT design	1. Risk of B	2. Inconsis	3. Indirectn	4. Impreci
Adults vs. TAU/WLC (34 RCTs)	4	-2	-1	0	0
Adults vs. attention/engagement (8 RCTs)	4	-2	-2	0	-1
Adults vs. bona fide (3 RCTs)	4	-2	-2	0	-2
Children/adolescents (2 RCTs)	4	-1	-2	0	-2
Primary care (3 RCTs)	4	0	-2	0	-2

For peer review only

5. Publicat	6*. Large r	7*. Dose R	8*. Effect (	GRADE	Quality
-1	0	0	0	0	Very low
-1	0	0	0	-2	Very low
-1	0	0	0	-3	Very low
-1	0	0	0	-2	Very low
-1	0	0	0	-1	Very low

For peer review only

PRISMA 2020 Checklist

Section and Topic	Item #	Checklist item	Location where item is reported
**TITLE**			Page:
Title	1	Identify the report as a systematic review.	1
**ABSTRACT**			
Abstract	2	See the PRISMA 2020 for Abstracts checklist.	3
**INTRODUCTION**			
Rationale	3	Describe the rationale for the review in the context of existing knowledge.	4
Objectives	4	Provide an explicit statement of the objective(s) or question(s) the review addresses.	5
**METHODS**			
Eligibility criteria	5	Specify the inclusion and exclusion criteria for the review and how studies were grouped for the syntheses.	6,7,8
Information sources	6	Specify all databases, registers, websites, organisations, reference lists and other sources searched or consulted to identify studies. Specify the date when each source was last searched or consulted.	5
Search strategy	7	Present the full search strategies for all databases, registers and websites, including any filters and limits used.	5 + Supplement A
Selection process	8	Specify the methods used to decide whether a study met the inclusion criteria of the review, including how many reviewers screened each record and each report retrieved, whether they worked independently, and if applicable, details of automation tools used in the process.	6
Data collection process	9	Specify the methods used to collect data from reports, including how many reviewers collected data from each report, whether they worked independently, any processes for obtaining or confirming data from study investigators, and if applicable, details of automation tools used in the process.	8,9
Data items	10a	List and define all outcomes for which data were sought. Specify whether all results that were compatible with each outcome domain in each study were sought (e.g. for all measures, time points, analyses), and if not, the methods used to decide which results to collect.	8,9
	10b	List and define all other variables for which data were sought (e.g. participant and intervention characteristics, funding sources). Describe any assumptions made about any missing or unclear information.	8,9
Study risk of bias assessment	11	Specify the methods used to assess risk of bias in the included studies, including details of the tool(s) used, how many reviewers assessed each study and whether they worked independently, and if applicable, details of automation tools used in the process.	10
Effect measures	12	Specify for each outcome the effect measure(s) (e.g. risk ratio, mean difference) used in the synthesis or presentation of results.	10, 11
Synthesis methods	13a	Describe the processes used to decide which studies were eligible for each synthesis (e.g. tabulating the study intervention characteristics and comparing against the planned groups for each synthesis (item #5)).	9 - 11
	13b	Describe any methods required to prepare the data for presentation or synthesis, such as handling of missing summary statistics, or data conversions.	10, 11
	13c	Describe any methods used to tabulate or visually display results of individual studies and syntheses.	10, 11
	13d	Describe any methods used to synthesize results and provide a rationale for the choice(s). If meta-analysis was performed, describe the model(s), method(s) to identify the presence and extent of statistical heterogeneity, and software package(s) used.	10-11
	13e	Describe any methods used to explore possible causes of heterogeneity among study results (e.g. subgroup analysis, meta-regression).	11
	13f	Describe any sensitivity analyses conducted to assess robustness of the synthesized results.	11
Reporting bias assessment	14	Describe any methods used to assess risk of bias due to missing results in a synthesis (arising from reporting biases).	11

For peer review only - http://bmjopen.bmj.com/site/about/guidelines.xhtml

PRISMA 2020 Checklist

Section and Topic	Item #	Checklist item	Location where item is reported
Certainty assessment	15	Describe any methods used to assess certainty (or confidence) in the body of evidence for an outcome.	11
**RESULTS**			
Study selection	16a	Describe the results of the search and selection process, from the number of records identified in the search to the number of studies included in the review, ideally using a flow diagram.	Fig 1
	16b	Cite studies that might appear to meet the inclusion criteria, but which were excluded, and explain why they were excluded.	11
Study characteristics	17	Cite each included study and present its characteristics.	
Risk of bias in studies	18	Present assessments of risk of bias for each included study.	11 + Fig 2
Results of individual studies	19	For all outcomes, present, for each study: (a) summary statistics for each group (where appropriate) and (b) an effect estimate and its precision (e.g. confidence/credible interval), ideally using structured tables or plots.	12, Fig 3, Fig 4
Results of syntheses	20a	For each synthesis, briefly summarise the characteristics and risk of bias among contributing studies.	13
	20b	Present results of all statistical syntheses conducted. If meta-analysis was done, present for each the summary estimate and its precision (e.g. confidence/credible interval) and measures of statistical heterogeneity. If comparing groups, describe the direction of the effect.	13
	20c	Present results of all investigations of possible causes of heterogeneity among study results.	13
	20d	Present results of all sensitivity analyses conducted to assess the robustness of the synthesized results.	14
Reporting biases	21	Present assessments of risk of bias due to missing results (arising from reporting biases) for each synthesis assessed.	14, Supplement 2
Certainty of evidence	22	Present assessments of certainty (or confidence) in the body of evidence for each outcome assessed.	13, 14, Figure 3, Figure 4,
**DISCUSSION**			
Discussion	23a	Provide a general interpretation of the results in the context of other evidence.	16, 17, 18
	23b	Discuss any limitations of the evidence included in the review.	19
	23c	Discuss any limitations of the review processes used.	19
	23d	Discuss implications of the results for practice, policy, and future research.	19
**OTHER INFORMATION**			
Registration and protocol	24a	Provide registration information for the review, including register name and registration number, or state that the review was not registered.	5
	24b	Indicate where the review protocol can be accessed, or state that a protocol was not prepared.	5
	24c	Describe and explain any amendments to information provided at registration or in the protocol.	10
Support	25	Describe sources of financial or non-financial support for the review, and the role of the funders or sponsors in the review.	21
Competing interests	26	Declare any competing interests of review authors.	3
Availability of	27	Report which of the following are publicly available and where they can be found: template data collection forms; data extracted from included	21

For peer review only

PRISMA 2020 Checklist

Section and Topic	Item #	Checklist item	Location where item is reported
data, code and other materials		studies; data used for all analyses; analytic code; any other materials used in the review.	

From: Page MJ, McKenzie JE, Bossuyt PM, Boutron I, Hoffmann TC, Mulrow CD, et al. The PRISMA 2020 statement: an updated guideline for reporting systematic reviews. BMJ 2021;372:n71. doi: 10.1136/bmj.n71

For more information, visit: http://www.prisma-statement.org/

For peer review only
